# Time Trends in Patient Characteristics, Anticoagulation Treatment, and Prognosis of Incident Nonvalvular Atrial Fibrillation in the Netherlands

**DOI:** 10.1001/jamanetworkopen.2023.9973

**Published:** 2023-04-25

**Authors:** Qingui Chen, Myrthe M. A. Toorop, Laurens F. Tops, Willem M. Lijfering, Suzanne C. Cannegieter

**Affiliations:** 1 Department of Clinical Epidemiology, Leiden University Medical Center, Leiden, the Netherlands; 2 Department of Cardiology, Leiden University Medical Center, Leiden, the Netherlands; 3 The Knowledge Institute of the Federation of Medical Specialists, Utrecht, the Netherlands; 4 Department of Medicine, Thrombosis and Hemostasis, Leiden University Medical Center, Leiden, the Netherlands

## Abstract

**Importance:**

The temporal trend in adverse events regarding stroke prevention for nonvalvular atrial fibrillation (NVAF) in the direct oral anticoagulant (DOAC) era was rarely investigated comprehensively, especially taking into account potential changes in patient characteristics and anticoagulation treatment.

**Objective:**

To investigate time trends in patient characteristics, anticoagulation treatment, and prognosis of patients with incident NVAF in the Netherlands.

**Design, Setting, and Participants:**

This retrospective cohort study assessed patients with incident NVAF initially recognized within a hospitalization between 2014 and 2018, using data from Statistics Netherlands. Participants were followed-up for 1 year from the hospital admission at which the incident NVAF diagnosis was made or until death, whichever occurred first. Data were analyzed from January 15, 2021, to March 8, 2023.

**Exposure:**

Calendar year of the incident NVAF diagnosis, according to which the participants were categorized into 5 cohorts.

**Main Outcomes and Measures:**

Outcomes of interest were baseline patient characteristics, anticoagulation treatment, and occurrence of ischemic stroke or major bleeding within the 1-year follow-up after incident NVAF.

**Results:**

Between 2014 and 2018, 301 301 patients (mean [SD] age, 74.2 [11.9] years; 169 748 [56.3%] male patients) experienced incident NVAF in the Netherlands, each of whom was categorized into 1 of 5 cohorts by calendar year. Baseline patient characteristics were broadly the same between cohorts with a mean (SD) CHA_2_DS_2_-VASc (congestive heart failure, hypertension, age ≥75 years [doubled], diabetes, stroke [doubled], vascular disease, age 65 to 74 years, and sex category [female]) score of 2.9 (1.7). The median (IQR) proportion of days covered by OACs (ie, vitamin K antagonists or DOACs) within the 1-year follow-up increased from 56.99% (0%-86.30%) to 75.62% (0%-94.52%), and DOACs increased from 5102 patients (13.5%) to 32 314 patients (72.0%) among those who received OACs, gradually replacing VKAs as the first choice of OACs. Over the course of the study, there were statistically significant decreases in the 1-year cumulative incidence of ischemic stroke (from 1.63% [95% CI, 1.52%-1.73%] to 1.39% [95% CI, 1.30%-1.48%) and major bleeding (from 2.50% [95% CI, 2.37%-2.63%] to 2.07% [95% CI, 1.96%-2.19%]), and the association was consistent after adjusting for baseline patient characteristics and excluding those with preexisting chronic anticoagulation.

**Conclusions and Relevance:**

This cohort study of patients with incident NVAF diagnosed between 2014 and 2018 in the Netherlands found similar baseline characteristics, increased OAC use with DOACs being favored over time, and improved 1-year prognosis. Comorbidity burden, potential underuse of anticoagulation medications, and specific subgroups of patients with NVAF remain directions for future investigations and further improvement.

## Key Points

**Question:**

What trends in patient characteristics, anticoagulation treatment, and prognosis were associated with patients with incident nonvalvular atrial fibrillation (NVAF) in the Netherlands in 2014 to 2018?

**Findings:**

In this cohort study that included 301 301 patients with incident NVAF, the patient population showed similar baseline characteristics between 2014 and 2018 but increasingly received oral anticoagulation (OAC) with direct OAC being favored and had a parallel decreasing 1-year cumulative incidence of both ischemic stroke and major bleeding.

**Meaning:**

These findings suggest that increasing use of OAC treatment, with direct OAC becoming the first-line anticoagulant, may partly explain the improved prognosis among patients with incident NVAF in the Netherlands.

## Teaser

This cohort study investigates time trends in patient characteristics, anticoagulation treatment, and prognosis of patients with incident nonvalvular atrial fibrillation in the Netherlands.

## Introduction

Atrial fibrillation (AF), a global health problem with increasing prevalence and incidence,^1,2^ is associated with an overall 5-fold increased risk of thromboembolic stroke and contributes to up to 20% of all strokes.^3,4^ This makes stroke prevention one of the cornerstones of AF management,^5,6,7^ with oral anticoagulation medication (OAC) being the preferred approach. For nonvalvular AF (NVAF), advances have been made in the past decade.^8^ The CHA_2_DS_2_-VASc (congestive heart failure, hypertension, age ≥75 years [doubled], diabetes, stroke [doubled], vascular disease, age 65 to 74 years, and sex category [female]) score^9^ has been recommended since 2010^10^ to assess stroke risk, and now plays a central role in determining whether OACs are indicated.^5,6,7^ Meanwhile, direct OACs (DOACs) have been replacing vitamin K antagonists (VKAs) as the first choice of OACs for stroke prevention,^11,12,13^ as noninferiority but superior safety for stroke prevention was demonstrated for DOACs compared with VKAs.^14,15,16,17^

However, it has not been thoroughly investigated whether stroke prevention has improved along with these advances in anticoagulation at the population level. Since evidence from clinical trials may lack generalizability^18,19^ and adherence to guidelines could be suboptimal,^20^ an update obtained from population-level data can be highly relevant to both clinical practice and research. To fill this knowledge gap, we investigated time trends in patient characteristics, anticoagulation treatment, and prognosis of patients with incident AF between 2014 and 2018 in the Netherlands.

## Methods

This cohort study adhered to the Declaration of Helsinki and received an ethical approval from the Scientific Committee of the Department of Clinical Epidemiology of the Leiden University Medical Center with a waiver of participant consent due to the use of preexisting, deidentified data only. We followed the Strengthening the Reporting of Observational Studies in Epidemiology (STROBE) reporting guideline for cohort studies.

### Data Sources

Nationwide data were accessed from Statistics Netherlands (Centraal Bureau voor de Statistiek), including data on diagnoses registered within hospitalizations, dates and causes of death, personal characteristics, and outpatient medication prescriptions. A detailed description of the data sources and codes for variable identification are provided in the eMethods and eTable 1 in Supplement 1.

### Study Population

The study population included patients with incident NVAF (including atrial flutter) initially recognized within a hospitalization who were not primarily admitted for ischemic stroke in the Netherlands between 2014 and 2018 (Figure). Detailed inclusion criteria are presented in the eMethods in Supplement 1. The admission date of the hospitalization in which the incident AF diagnosis was made (ie, the index hospitalization) was considered as the index date, and patients were further categorized into 5 cohorts by calendar year of the index date, namely 2014, 2015, 2016, 2017, and 2018.

**Figure.**
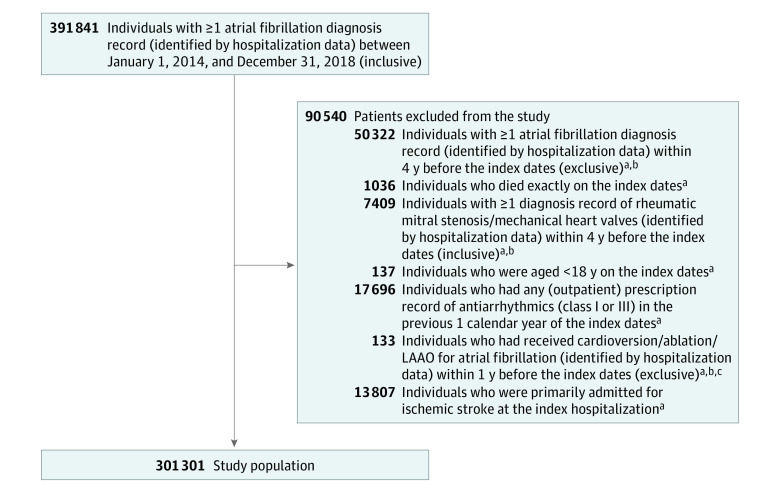
Flowchart for the Inclusion of the Study Population LAAO indicates left atrial appendage occlusion. ^a^The index date refers to the admission date of the hospitalization in which a diagnosis record of atrial fibrillation was registered for the first time between January 1, 2014, and December 31, 2018. ^b^One year was counted as 365.25 days. ^c^There were 12 patients who received cardioversion and 121 patients who received ablation, but no patients received LAAO.

### Baseline Patient Characteristics

The following baseline patient characteristics were identified on the index dates: age, sex, immigration background, standardized household income, marital status, type of AF, CHA_2_DS_2_-VASc score,^9^ HAS-BLED (hypertension, abnormal renal and liver function, stroke, bleeding, elderly, and drugs or alcohol abuse) score,^21^ preexisting chronic use of antithrombotic agents, and various comorbidities (or medical history). Immigration status data were collected directly from Statistics Netherlands. Details about all the investigated baseline patient characteristics are presented in the eMethods in Supplement 1.

### Follow-up, Anticoagulation Treatment, and Prognosis

All the included patients were followed from the index date up to 1 year or date of death, whichever came first. We examined data on outpatient prescriptions of antithrombotic agents during the follow-up to determine: (1) whether a patient received at least 1 prescription of any OAC (ie, VKA or DOAC), VKA, DOAC, heparin group, or antiplatelet agent; (2) type of first-received OAC among those who received OACs; and (3) proportion of days covered (PDC) by OACs or heparin group medications, OACs only, or antiplatelet agents. Details about the calculation of PDC are presented in the eMethods and eFigure 1 in Supplement 1. The following adverse events were included as clinical outcomes: ischemic stroke, major bleeding, intracranial hemorrhage (ICH), gastrointestinal bleeding, and all-cause mortality, which were identified through data on diagnoses and primary cause of death (eMethods in Supplement 1).

### Statistical Analysis

Baseline patient characteristics and anticoagulation treatment received during the follow-up by cohorts are described as mean and SD, median and IQR, or numbers and percentages. The description of anticoagulation treatment was further stratified by baseline CHA_2_DS_2_-VASc score (ie, 0, 1, and ≥2); the description of baseline patient characteristics was further stratified by the received anticoagulation treatment (ie, no OAC, ≥1 OAC, and receiving VKA or DOAC as the first-received OAC). Incidence rates and cumulative incidences of the clinical outcomes were estimated within each cohort, where cumulative incidences were estimated by the cumulative incidence competing risk method, except for all-cause mortality, which was estimated by the Kaplan-Meier estimator. Patients who experienced a specific outcome event exactly on the index dates were excluded from analyses into this outcome. To further compare the clinical outcomes among cohorts, the cohort of patients diagnosed in 2018 was used as the reference group, and hazard ratios (HRs) of the clinical outcomes in the other cohorts (vs the reference group) were estimated using Cox regression models. Besides a crude model, several adjustment models were prespecified (eMethods in Supplement 1). As sensitivity analyses, all analyses were repeated after excluding patients who had at least 2 prescription records of VKAs or DOACs within 6 months before the index date (exclusive).

Subgroup analyses were performed by estimating incidence rates and cumulative incidences of the clinical outcomes for each cohort according to several prespecified baseline patient characteristics. We also described the individual items of the CHA_2_DS_2_-VASc score and the HAS-BLED score of each cohort overall and by sex. As a check on data quality, we estimated the association of baseline CHA_2_DS_2_-VASc score with the 1-year risk of ischemic stroke in the complete study population (for comparison with literature^9^). In addition, we determined numbers and proportions of several prespecified diagnoses made in the complete nationwide data to identify a potential change in data quality over the years.

Since the study used nationwide data, loss to follow-up was assumed as rare and ignored; absence of records on medication prescription or diagnoses in the data sources were assumed as absence of these medications or diseases; only the variable standardized household income had missing values (<0.2%) in the original data, and a complete-case analysis was used when this variable was involved.

All statistical analyses were performed with SPSS Statistics version 25.0 (IBM) and R version 4.1.3 (R Project for Statistical Computing). Data were analyzed from January 15, 2021, to March 8, 2023.

## Results

### Time Trends in Patient Characteristics of Incident AF

A total of 301 301 patients (mean [SD] age, 74.2 [11.9] years; 169 748 [56.3%] male patients) with incident AF were identified (Figure). The sizes of the cohorts were largely similar, with approximately 61 000 patients per year (Table 1), except for 2014, which included 55 880 patients. For index hospitalizations, the median length of hospital stay was 3 days, with IQRs ranging from 0 to 8, and all years had in-hospital mortality rates of approximately 4% (eg, 2412 patients [4.3%] in 2014 and 2865 patients [4.6%] in 2018) (Table 1). According to the recorded main admission reason for the index hospitalizations, 112 459 patients (37.3%) were primarily admitted for AF, followed by heart failure (24 016 patients [8.0%]), respiratory diseases (23 666 patients [7.9%]), and ischemic heart diseases (23 062 patients [7.7%]) (eTable 2 in Supplement 1).

**Table 1.** Time Trends in Patient Characteristics of Incident Nonvalvular Atrial Fibrillation

Characteristic	Patients by year of diagnosis, No. (%)				
	2014 (n = 55 880)	2015 (n = 61 317)	2016 (n = 60 018)	2017 (n = 62 390)	2018 (n = 61 696)
Age, mean (SD), years	74.5 (11.9)	74.3 (12.0)	74.1 (12.0)	74.1 (11.9)	74.1 (11.9)
Sex					
Female	24 489 (43.8)	27 271 (44.5)	26 257 (43.7)	27 086 (43.4)	26 450 (42.9)
Male	31 391 (56.2)	34 046 (55.5)	33 761 (56.3)	35 304 (56.6)	35 246 (57.1)
Immigration background^a^					
Native Dutch	49 197 (88.0)	53 983 (88.0)	52 449 (87.4)	54 543 (87.4)	53 914 (87.4)
First-generation immigrants	3741 (6.7)	4022 (6.6)	4166 (6.9)	4357 (7.0)	4423 (7.2)
Second-generation immigrants	2942 (5.3)	3312 (5.4)	3403 (5.7)	3490 (5.6)	3359 (5.4)
Standardized household income, quintile^b^					
First (lowest)	9531 (17.1)	10 451 (17.1)	10 600 (17.7)	11 261 (18.1)	11 176 (18.2)
Second	15 462 (27.7)	17 181 (28.1)	16 757 (28.0)	17 577 (28.2)	17 258 (28.0)
Third	12 970 (23.2)	13 978 (22.8)	13 779 (23.0)	14 496 (23.3)	14 578 (23.7)
Fourth	8426 (15.1)	9452 (15.4)	9262 (15.5)	9443 (15.2)	9339 (15.2)
Fifth (highest)	6744 (12.1)	7598 (12.4)	7324 (12.2)	7551 (12.1)	7383 (12.0)
Private household with an unknown income	31 (0.1)	46 (0.1)	49 (0.1)	41 (0.1)	38 (0.1)
Institutional household	2629 (4.7)	2501 (4.1)	2145 (3.6)	1884 (3.0)	1799 (2.9)
Type of atrial fibrillation					
Paroxysmal atrial fibrillation	NA	9756 (15.9)	9478 (15.8)	9683 (15.5)	9068 (14.7)
Persistent atrial fibrillation	NA	1897 (3.1)	2007 (3.3)	2131 (3.4)	2101 (3.4)
Chronic atrial fibrillation	NA	3240 (5.3)	3210 (5.3)	3306 (5.3)	3281 (5.3)
Type I atrial flutter	NA	384 (0.6)	437 (0.7)	570 (0.9)	688 (1.1)
Type II atrial flutter	NA	173 (0.3)	146 (0.2)	133 (0.2)	130 (0.2)
Unspecified atrial fibrillation	NA	45 867 (74.8)	44 740 (74.5)	46 567 (74.6)	46 428 (75.3)
Diagnosis made before 2015	55 880 (100)	0	0	0	0
CHA_2_DS_2_-VASc score					
Mean (SD)	2.95 (1.67)	2.91 (1.68)	2.89 (1.67)	2.90 (1.67)	2.90 (1.66)
≥2	44 420 (79.5)	48 221 (78.6)	47 068 (78.4)	49 053 (78.6)	48 542 (78.7)
HAS-BLED score, mean (SD)^c^					
Mean (SD)	1.79 (1.02)	1.73 (1.02)	1.71 (1.02)	1.70 (1.02)	1.69 (1.01)
≥3^c^	12 912 (23.1)	13 052 (21.3)	12 547 (20.9)	12 968 (20.8)	12 416 (20.1)
Preexisting chronic use of antithrombotic agents^d^					
Oral anticoagulant					
Any	15 217 (27.2)	17 168 (28.0)	16 665 (27.8)	17 973 (28.8)	19 110 (31.0)
Vitamin K antagonist	13 335 (23.9)	13 121 (21.4)	10 721 (17.9)	9211 (14.8)	7668 (12.4)
Direct oral anticoagulant	1941 (3.5)	4186 (6.8)	6107 (10.2)	8916 (14.3)	11 563 (18.7)
Heparin group	910 (1.6)	897 (1.5)	830 (1.4)	670 (1.1)	533 (0.9)
Antiplatelet agent	16 060 (28.7)	16 304 (26.6)	15 790 (26.3)	16 683 (26.7)	16 264 (26.4)
≥1 Hospitalization within the prior 4 y	37 004 (66.2)	39 243 (64.0)	38 189 (63.6)	39 582 (63.4)	38 138 (61.8)
Information about the index hospitalization					
Length of hospital stay, median (IQR), d	3 (1-8)	3 (0-8)	3 (0-8)	3 (0-7)	3 (0-7)
In-hospital mortality	2412 (4.3)	2622 (4.3)	2557 (4.3)	2709 (4.3)	2865 (4.6)
Primarily admitted for atrial fibrillation	19 496 (34.9)	23 257 (37.9)	23 047 (38.4)	23 782 (38.1)	22 877 (37.1)

Abbreviations: CHA_2_DS_2_-VASc, congestive heart failure, hypertension, age ≥75 years (doubled), diabetes, stroke (doubled), vascular disease, age 65 to 74 years, and sex category (female); HAS-BLED, hypertension, abnormal renal and liver function, stroke, bleeding, elderly, and drugs or alcohol abuse; NA, not available.

^a^ Immigration data were collected from Statistics Netherlands. First-generation immigrants refers to persons who were born abroad with at least 1 parent who was born abroad; second-generation immigrants refers to persons who was born in the Netherlands with at least 1 parent who was born abroad.

^b^ Percentile groups were determined based on disposable income of private households of the complete target population in the database (instead of the study population only).

^c^ Labile international normalized ratio was not included for calculation.

^d^ Preexisting chronic use was defined as at least 2 (outpatient) prescription records of the same type of antithrombotic agents within 6 months (ie, 183 days) before the index dates (exclusive). Preexisting chronic use of oral anticoagulant refers to persons with preexisting chronic use of vitamin K antagonist or direct oral anticoagulant.

The baseline patient characteristics were similar between cohorts (Table 1; eTable 2 in Supplement 1). The overall mean (SD) CHA_2_DS_2_-VASc score was 2.9 (1.7), and the mean (SD) HAS-BLED score was 1.7 (1.0), although the patients diagnosed in 2014 were older (mean [SD] age, 74.5 [11.9] years) and had a higher baseline CHA_2_DS_2_-VASc score (mean [SD] score, 3.0 [1.7]) and HAS-BLED score (mean [SD] score, 1.8 [1.0]). Information about types of AF was available from 2015 on and was unspecified in 183 602 patients (74.8%), paroxysmal in 37 985 patients (15.5%), and persistent in 8136 patients (3.3%) (Table 1). The most prevalent comorbidities were hypertension, heart failure, and diabetes (eTable 2 in Supplement 1).

The proportion of preexisting chronic use of OACs increased over time, from 15 217 patients (27.2%) in 2014 to 19 110 patients (31.0%) in 2018, with an increasing proportion of DOACs vs VKAs, but the proportion of preexisting chronic antiplatelet agents use remained fairly constant, at between 26% and 29% (Table 1). After excluding patients with preexisting chronic use of OACs, the time trends in baseline patient characteristics were consistent with those in the complete study population (eTable 3 in Supplement 1).

### Time Trends in Anticoagulation Treatment Within 1 Year After Incident AF Diagnosis

The proportion of patients receiving at least 1 prescription of OAC within 1 year after incident AF diagnosis was approximately 72% in each cohort (Table 2), but it was lower in 2014 (37 905 patients [67.8%]). Among those who received at least 1 prescription of OAC, DOAC gradually replaced VKA as the first option, increasing from 5102 patients (13.5%) in 2014 to 32 314 patients (72.0%) in 2018. When evaluated by PDCs, the median (IQR) PDC by OACs or heparin group increased from 63.01% (0%-89.04%) to 77.53% (0%-95.07%), and this trend was similar to that observed in median PDC by OACs only (from 56.99% [0%-86.30%] to 75.62% [0%-94.52%]). Use of antiplatelet agents remained stable, although in 2014, there was a higher proportion of patients receiving at least 1 antiplatelet agent (Table 2). After excluding patients with preexisting chronic use of OACs, these time trends remained the same (eTable 4 in Supplement 1).

**Table 2.** Time Trends in Anticoagulation Treatment Within 1 Year After Incident Nonvalvular Atrial Fibrillation Diagnosis

Treatment	Patients by year of diagnosis, No. (%)				
	2014 (n = 55 880)	2015 (n = 61 317)	2016 (n = 60 018)	2017 (n = 62 390)	2018 (n = 61 696)
Received ≥1 antithrombotic agent prescription					
Oral anticoagulant	37 905 (67.8)	44 426 (72.5)	43 767 (72.9)	45 144 (72.4)	44 881 (72.7)
Vitamin K antagonist	33 179 (59.4)	32 078 (52.3)	23 980 (40.0)	17 774 (28.5)	13 283 (21.5)
Direct oral anticoagulant	6429 (11.5)	14 808 (24.1)	22 304 (37.2)	29 579 (47.4)	33 555 (54.4)
Heparin group	6982 (12.5)	6810 (11.1)	5464 (9.1)	4401 (7.1)	3680 (6.0)
Antiplatelet agent	16 218 (29.0)	15 574 (25.4)	14 966 (24.9)	16 270 (26.1)	15 794 (25.6)
Type of first-received oral anticoagulant					
Vitamin K antagonist	32 803 (58.7)	31 387 (51.2)	23 152 (38.6)	16 979 (27.2)	12 567 (20.4)
Direct oral anticoagulant	5102 (9.1)	13 039 (21.3)	20 615 (34.3)	28 165 (45.1)	32 314 (52.4)
Without receiving oral anticoagulant	17 975 (32.2)	16 891 (27.5)	16 251 (27.1)	17 246 (27.6)	16 815 (27.3)
Proportion of days covered, median (IQR), %^a^					
Oral anticoagulant or heparin group	63.01 (0-89.04)	70.68 (0-92.05)	72.88 (0-93.42)	75.34 (0-94.52)	77.53 (0-95.07)
Oral anticoagulant	56.99 (0-86.30)	66.30 (0-89.86)	69.04 (0-92.05)	73.42 (0-93.75)	75.62 (0-94.52)
Antiplatelet agent	0 (0-26.58)	0 (0-14.29)	0 (0-0)	0 (0-24.66)	0 (0-21.92)

^a^ When calculating proportion of days covered by oral anticoagulant or heparin group, each patient was followed for 1 year (or until death if it occurred within 1 year), and each prescription of vitamin K antagonist, direct oral anticoagulant, or heparin group was assumed to be prescribed for 90 days unless there was a refill within 90 days, regardless of types of anticoagulants. The proportion of days covered by antiplatelet agents was calculated in the same way, but only prescriptions of antiplatelet agents were examined.

Increasing PDC by OACs and use of DOACs were observed as well when the cohorts were stratified by baseline CHA_2_DS_2_-VASc score (eTable 5 in Supplement 1), except that in the stratum with a baseline CHA_2_DS_2_-VASc score of 0, the PDC by OACs decreases over time. The absolute PDC by OACs in this stratum was also much lower compared with that of patients from the same cohort but with higher baseline CHA_2_DS_2_-VASc scores, which was consistent after excluding patients with preexisting chronic use of OACs (eTable 6 in Supplement 1).

Compared with patients who did not receive OACs within the 1-year follow-up, patients who received at least 1 OAC were older, had higher CHA_2_DS_2_-VASc and HAS-BLED scores, and had a higher proportion of patients with preexisting chronic use of OACs but a lower proportion of patients receiving antiplatelet agents (eTable 7 and eTable 8 in Supplement 1). Compared with patients who received VKA as the first OAC, those who received DOAC as the first OAC were younger, had lower baseline CHA_2_DS_2_-VASc and HAS-BLED scores, and had a higher proportion of preexisting chronic use of DOACs but a lower proportion of patients with preexisting chronic VKAs use (eTable 9 and eTable 10 in Supplement 1). Although the patients using VKAs generally had a higher prevalence of the most investigated comorbidities than those using DOACs, this was particularly noticeable for patients with comorbidities, such as heart failure, other valvular heart disease, and chronic kidney diseases. Results were consistent after excluding patients with preexisting chronic use of OACs (eTables 11-14 in Supplement 1).

### Time Trends in Prognosis Within 1 Year After Incident AF Diagnosis

Except for the cumulative incidence of gastrointestinal bleeding, which remined generally consistent (Table 3), the 1-year cumulative incidences of the investigated clinical events all showed a decreasing trend from 2014 to 2018, including ischemic stroke (from 1.63% [95% CI, 1.52%-1.73%] to 1.39% [95% CI, 1.30%-1.48%]), major bleeding (from 2.50% [95% CI, 2.37%-2.63%] to 2.07% [95% CI, 1.96%-2.19%]), ICH (from 0.60% [95% CI, 0.54%-0.67%] to 0.40% [95% CI, 0.36%-0.46%]), and all-cause mortality (from 19.13% [95% CI, 18.80%-19.45%] to 17.67% [95% CI, 17.37%-17.97%]) (Table 3). The risk of these events was higher within the first month after the AF diagnosis than in the subsequent period (eTable 15 and eFigures 2-6 in Supplement 1). After excluding patients with preexisting chronic use of OACs, the trends were consistent (eTable 16, eTable 17, and eFigures 7-11 in Supplement 1).

**Table 3.** Time Trends in Prognosis Within 1 Year After Incident Nonvalvular Atrial Fibrillation Diagnosis

Year of diagnosis^a^	No. at risk^b^	PYs	Events, No.	Cumulative incidence, % (95% CI)^c^	Incidence rate (95% CI), per 100 PYs	HR (95% CI)	
						Crude	Adjusted^d^
**Ischemic stroke**								
2014	55 880	47 930	909	1.63 (1.52-1.73)	1.90 (1.78-2.02)	1.18 (1.07-1.29)	1.26 (1.11-1.42)
2015	61 317	52 877	1003	1.64 (1.54-1.74)	1.90 (1.78-2.02)	1.18 (1.08-1.29)	1.18 (1.08-1.29)
2016	60 018	51 971	912	1.52 (1.42-1.62)	1.75 (1.64-1.87)	1.09 (0.99-1.20)	1.11 (1.01-1.22)
2017	62 390	54 099	917	1.47 (1.38-1.57)	1.70 (1.59-1.81)	1.06 (0.96-1.16)	1.07 (0.98-1.18)
2018	61 696	53 363	857	1.39 (1.30-1.48)	1.61 (1.50-1.72)	1 [Reference]	1 [Reference]
**Major bleeding**								
2014	54 773	46 860	1368	2.50 (2.37-2.63)	2.92 (2.77-3.08)	1.21 (1.12-1.31)	1.19 (1.08-1.32)
2015	60 221	51 862	1336	2.22 (2.10-2.34)	2.58 (2.44-2.72)	1.07 (0.99-1.16)	1.10 (1.02-1.19)
2016	59 078	51 108	1266	2.14 (2.03-2.26)	2.48 (2.34-2.62)	1.03 (0.95-1.11)	1.06 (0.98-1.14)
2017	61 372	53 134	1246	2.03 (1.92-2.14)	2.35 (2.22-2.48)	0.98 (0.90-1.06)	0.99 (0.91-1.07)
2018	60 701	52 412	1259	2.07 (1.96-2.19)	2.40 (2.27-2.54)	1 [Reference]	1 [Reference]
**ICH**								
2014	55 468	47 915	332	0.60 (0.54-0.67)	0.69 (0.62-0.77)	1.50 (1.27-1.76)	1.66 (1.32-2.08)
2015	60 907	52 912	313	0.51 (0.46-0.57)	0.59 (0.53-0.66)	1.28 (1.08-1.51)	1.29 (1.09-1.52)
2016	59 655	52 047	259	0.43 (0.38-0.49)	0.50 (0.44-0.56)	1.08 (0.90-1.28)	1.10 (0.93-1.31)
2017	62 005	54 138	223	0.36 (0.32-0.41)	0.41 (0.36-0.47)	0.89 (0.74-1.07)	0.90 (0.75-1.08)
2018	61 339	53 408	247	0.40 (0.36-0.46)	0.46 (0.41-0.52)	1 [Reference]	1 [Reference]
**GI bleeding**								
2014	55 522	47 701	548	0.99 (0.91-1.07)	1.15 (1.05-1.25)	0.99 (0.88-1.11)	0.94 (0.81-1.09)
2015	60 982	52 710	571	0.94 (0.86-1.02)	1.08 (1.00-1.18)	0.94 (0.83-1.05)	0.96 (0.86-1.07)
2016	59 716	51 826	566	0.95 (0.87-1.03)	1.09 (1.00-1.19)	0.94 (0.84-1.06)	0.97 (0.86-1.08)
2017	62 071	53 883	615	0.99 (0.92-1.07)	1.14 (1.05-1.24)	0.99 (0.88-1.10)	1.00 (0.89-1.11)
2018	61 388	53 169	615	1.00 (0.93-1.08)	1.16 (1.07-1.25)	1 [Reference]	1 [Reference]
**All-cause mortality**								
2014	55 880	48 254	10 689	19.13 (18.80-19.45)	22.15 (21.73-22.58)	1.09 (1.06-1.12)	1.28 (1.24-1.33)
2015	61 317	53 256	11 194	18.26 (17.95-18.56)	21.02 (20.63-21.41)	1.03 (1.01-1.06)	1.04 (1.01-1.07)
2016	60 018	52 337	10 675	17.79 (17.48-18.09)	20.40 (20.01-20.79)	1.00 (0.98-1.03)	1.01 (0.99-1.04)
2017	62 390	54 443	11 062	17.73 (17.43-18.03)	20.32 (19.94-20.70)	1.00 (0.97-1.03)	1.00 (0.98-1.03)
2018	61 696	53 709	10 900	17.67 (17.37-17.97)	20.29 (19.92-20.68)	1 [Reference]	1 [Reference]

Abbreviations: GI, gastrointestinal; HR, hazard ratio; ICH, intracranial hemorrhage; PY, person-year.

^a^ Including death caused by the clinical event.

^b^ Individuals who were primarily admitted to the index hospitalization for the clinical event were excluded.

^c^ Estimated by the cumulative incidence competing risk method, except for all-cause mortality, which was estimated by the Kaplan-Meier estimator.

^d^ Adjusted for age, sex, immigration background, standard household income, type of atrial fibrillation, and comorbidities, including asthma, chronic obstructive pulmonary disease, other chronic lung diseases, heart failure, myocardial infarction (history), hypertension, other valvular heart disease (except for rheumatic mitral stenosis and mechanical heart valves), peripheral artery disease, liver diseases, gastroesophageal reflux disease, peptic ulcer disease, chronic kidney diseases, anemia, coagulopathy, diabetes, thyroid disease, ischemic stroke (history), transient ischemic attack, other arterial thromboembolism, Parkinson disease, Alzheimer disease, autoimmune disease, systemic connective tissue disorders, venous thromboembolism, major bleeding, and malignant tumor. Comorbidities were identified within the prior 4 years and within index hospitalization separately, except for venous thromboembolism, which was identified between the prior 4 years and prior 6 months and within the prior 6 months separately.

After adjustment for patient characteristics and compared with the 2018 cohort, 1-year hazards in the 2014 cohort were significant higher for ischemic stroke (adjusted HR 1.26 [95% CI, 1.11-1.42]), major bleeding (adjusted HR, 1.19 [95% CI, 1.08-1.32]), and all-cause mortality (adjusted HR, 1.28 [95% CI, 1.24-1.33]) (Table 3). This risk of ICH was also higher in the 2014 cohort than the 2018 cohort (adjusted HR, 1.66 [95% CI, 1.32-2.08]), while for gastrointestinal bleeding, there was no statistically significant difference (adjusted HR, 0.94 [95% CI, 0.81-1.09]). The results were consistent after excluding patients with preexisting chronic use of OACs (eTable 16 in Supplement 1), and when using different adjustment models (eTable 18 and eTable 19 in Supplement 1).

The trends in prognosis within the investigated subgroups were similar to those in the complete study population, especially for subgroups with relatively large sizes (eTables 20-24 in Supplement 1). Specifically, although the risk of both adverse events showed a decreasing trend, female patients experienced a higher risk of ischemic stroke but a lower risk of major bleeding compared with male patients diagnosed in the same calendar years.

### Sensitivity Analyses

Among the individual items of the CHA_2_DS_2_-VASc and HAS-BLED scores, the prevalence of the item stroke, transient ischemic attack, or thromboembolism in the CHA_2_DS_2_-VASc score decreased, and uncontrolled hypertension, ischemic stroke or transient ischemic attack (history), and antiplatelet agents or nonsteroidal anti-inflammatory drugs all decreased in the HAS-BLED score (eTable 25 in Supplement 1). When comparing between the sexes, female patients had higher total scores than male patients, and a clear sex difference in items composing the 2 scores could be observed for the CHA_2_DS_2_-VASc score: female patients had higher prevalence of the items heart failure, hypertension, and (older) age but a lower prevalence of vascular disease; for the HAS-BLED score, female patients had higher prevalence of uncontrolled hypertension and being elderly but lower prevalence of alcohol abuse (eTable 26 and eTable 27 in Supplement 1). Results were similar when excluding patients with preexisting chronic use of OACs (eTables 28-30 in Supplement 1).

The baseline CHA_2_DS_2_-VASc score was associated with the 1-year risk of ischemic stroke following a dose-response pattern (eTable 31 in Supplement 1). eTable 32 in Supplement 1 presents the numbers and proportions of several diagnoses in the complete data source, indicating that the proportions of AF, rheumatic mitral stenosis or mechanical heart valves, ischemic stroke, major bleeding, and gastrointestinal bleeding to the total number of diagnoses made in 2014 were all lower than that in the subsequent years.

## Discussion

This cohort study investigated time trends in patient characteristics, anticoagulation treatment, and prognosis of patients with incident AF initially identified within a hospitalization between 2014 and 2018 in the Netherlands. The main findings were that the baseline characteristics of patients with incident AF remained broadly the same among calendar years, OACs were increasingly prescribed within 1 year after incident AF diagnosis, DOACs replaced VKA as the first option of OAC, and the 1-year risks of both ischemic stroke and major bleeding decreased. This study provides a comprehensive overview of population-level anticoagulation practice in patients with NVAF in the Netherlands in recent years, giving insights into characteristics of patients with newly diagnosed AF, their (subsequent) anticoagulation management, their prognosis, and whether these changed over time. These findings might benefit further improvement in AF management by raising awareness on the comorbidity burden in this patient population, revealing potential problems of current anticoagulation practice, and indicating research directions.

### Time Trends in Patient Characteristics of Incident AF

Studies that investigated time trends in characteristics of patients with AF using updated and large-scale data are rare. Compared with studies that investigated patients with incident AF in European countries,^22,23,24,25^ the baseline characteristics of our study population are consistent: most were aged 55 years or older, and men accounted for a higher proportion. Similarly, a high prevalence of comorbidities, such as hypertension, heart failure, and diabetes, was also reported by studies that presented this information.^22,25^ What our study adds to this topic is that the time trends in the prevalence of most comorbidities remained constant between 2014 and 2018, which might be a direction for further improvement in care of these patients.

### Time Trends in Anticoagulation Treatment for AF

Our findings are consistent with most studies that investigated anticoagulation in patients with AF,^11,12,13,25,26,27,28,29,30,31,32,33,34,35^ although they generally only reported crude proportions instead of PDCs to evaluate OAC use. As PDC itself is a metric for evaluating medication adherence and the increasing PDC with time was only observed in patients with a high baseline CHA_2_DS_2_-VASc score, our results suggest that OACs were increasingly prescribed to patients for whom this was indeed indicated. However, there was still room for further improvement, as approximately 30% of the patients with a CHA_2_DS_2_-VASc score of 2 or greater did not receive OACs. It should be noted that this crude proportion might be overestimated, as data on medications prescribed during hospitalizations were unavailable in our study. Still, several other studies reported a comparable proportion of patients with AF who had an indication to receive OAC but did not do so.^36,37^ Studies with more granular data are suggested to investigate whether anticoagulation treatment is actually appropriate for these patients.

We found that patients who received VKA were generally older and had more comorbidities than those who received DOAC. This may suggest there are still safety or efficacy concerns about the use of DOACs in patients with some specific characteristics, such as older age, heart failure, other valvular heart disease, and chronic kidney diseases. Some of these concerns about DOACs may not be well supported by evidence^38,39,40,41^; therefore, investigations are needed to examine the benefits and risks of DOAC use in these subgroups. Interestingly, we found clear sex differences in the distribution of components of CHA_2_DS_2_-VASc score and HAS-BLED score, and correspondingly, there was a higher risk of ischemic stroke but a lower risk of major bleeding among female patients compared with male patients. A further initial analysis found there was no significant sex difference in OAC use, while the proportion of male patients receiving antiplatelet agents was higher than that of female patients.

### Time Trends in Prognosis of Incident AF

Findings about time trends in AF prognosis from most currently available large-scale studies are inconsistent and difficult to interpret. Most of these studies were actually answering different research questions than ours, as they investigated time trends in prevalence of AF in patients with ischemic stroke,^31,42,43,44,45,46,47,48,49,50^ rather than time trends in incidence of ischemic stroke among a complete population of patients with AF. The few studies that used an appropriate study design^25,51^ are rather outdated, and potential changes in patient characteristics or anticoagulation treatment over time were not well considered.^51^ A 2019 study by Hohnloser et al^25^ analyzed whether the introduction of DOACs benefited ischemic stroke prevention in patients with AF and found that the risk of ischemic stroke decreased but bleeding complications did not. However, Hohnloser et al^25^ only compared patients with prevalent AF in 2016 vs those in 2011, while the 2 cohorts had significant difference in anticoagulation use. A 2021 study by Wilton et al^51^ used a similar design to ours in which they included patients with incident AF in Canada between 2006 and 2015 and investigated the 1-year risk of ischemic stroke and major bleeding, but they did not investigate OAC use at the same time. Compared with these studies, our study was strengthened by the nationwide (complete and unselected) population of patients with incident AF, the updated data, the well-defined cohort study design, and the adjustment for many patient characteristics.

In addition, since we presented time trends in various patient characteristics, anticoagulation treatment, and prognosis, our study might provide insights into factors associated with changes in prognosis; however, it should also be noted that our study is of a descriptive nature, and therefore the findings cannot be interpreted in a causal way. Although it seems intuitively reasonable to attribute the observed improvement in prognosis to the observed change in anticoagulation treatment, there are still many other variables that might have changed over time and at the same time have affected prognosis, such as other (unmeasured) patient characteristics, dosage of anticoagulant,^52^ medication persistence,^53^ and subtypes of DOACs.^54^

### Limitations

This study has several limitations. First, as registries from various sources were used and most variables were identified by codes, information from our study is at risk of misclassification. However, the patient characteristics and the absolute rates of the clinical outcomes in our study are comparable to similar investigations,^14,15,16,17,55,56,57^ and a fair association between the CHA_2_DS_2_-VASc score and stroke risk was observed, which suggests that the impact of any misclassification may have been low. Some important information, such as indications and amounts of OACs prescriptions, was unavailable in our study, and assumptions were introduced. It should be kept in mind that it is informative and important to focus on the relative changes between the calendar years when interpreting results from our study, since the possible misclassifications are likely to be constant over the years. The patient cohort in 2014 was an outlier in our study, which might be due to variation in data quality. However, as the observed trends in patient characteristics, anticoagulation treatment, and prognosis remained consistent even after excluding patients from 2014, our findings should be considered robust. Second, due to lack of outpatient diagnoses data, the study population was all initially hospitalized, which may have led to exclusion of some healthier patients with AF. However, it should be noted that all-cause inpatient contact is rather common in patients with incident AF^58,59^; therefore, the 4-year time window we used to identify AF using hospitalization data should capture most patients with AF. Furthermore, in the Netherlands, most patients with AF first seen by general practitioners are referred to a specialist,^60^ and there is no reason to assume a specialist’s anticoagulation strategy would differ greatly between outpatients and inpatients. Additionally, the patient characteristics and anticoagulant use reported by a recent Dutch study^37^ in community settings are very similar to ours. Third, we only investigated anticoagulation treatment and clinical outcomes within 1 year after incident AF diagnosis, and thus the trends in a longer period remain underreported.

## Conclusions

In this cohort study of patients with incident NVAF diagnosed between 2014 and 2018 in the Netherlands, there were similar baseline characteristics, increased OAC use with DOACs being favored, and an improved 1-year prognosis. Comorbidity burden, potential underuse of anticoagulation medications, and specific subgroups of patients with NVAF remain directions for future investigations and further improvement.

## Notes

Supplement 1.
eMethods.
**eTable 1.** Codes Used for Variable Identification **eTable 2.** Time Trends in Other Patient Characteristics of Incident Nonvalvular Atrial Fibrillation **eTable 3.** Time Trends in Patient Characteristics of Incident Nonvalvular Atrial Fibrillation, Excluding Patients With Preexisting Chronic Oral Anticoagulant Treatment **eTable 4.** Time Trends in Anticoagulation Treatment Within 1 Year After Incident Nonvalvular Atrial Fibrillation Diagnosis, Excluding Patients With Preexisting Chronic Oral Anticoagulant Treatment **eTable 5.** Time Trends in Anticoagulation Treatment Within 1 Year After Incident Nonvalvular Atrial Fibrillation, Stratified by Baseline CHA_2_DS_2_-VASc Score **eTable 6.** Time Trends in Anticoagulation Treatment Within 1 Year After Incident Nonvalvular Atrial Fibrillation Diagnosis, Stratified by Baseline CHA_2_DS_2_-VASc Score Excluding Patients With Preexisting Chronic Oral Anticoagulant Treatment **eTable 7.** Time Trends in Patient Characteristics of Incident Nonvalvular Atrial Fibrillation Among Patients Who Did Not Receive Any Oral Anticoagulants Within the 1-Year Follow-up **eTable 8.** Time Trends in Patient Characteristics of Incident Nonvalvular Atrial Fibrillation Among Patients Who Received at Least 1 Oral Anticoagulant Within the 1-Year Follow-up **eTable 9.** Time Trends in Patient Characteristics of Incident Nonvalvular Atrial Fibrillation Among Patients Who First Received Vitamin K Antagonist as Oral Anticoagulant Within the 1-Year Follow-up **eTable 10.** Time Trends in Patient Characteristics of Incident Nonvalvular Atrial Fibrillation Among Patients Who First Received Direct Oral Anticoagulant as Oral Anticoagulant Within the 1-Year Follow-up **eTable 11.** Time Trends in Patient Characteristics of Incident Nonvalvular Atrial Fibrillation Among Patients Who Did Not Receive Any Oral Anticoagulants Within the 1-Year Follow-up, Excluding Patients With Preexisting Chronic Oral Anticoagulant Treatment **eTable 12.** Time Trends in Patient Characteristics of Incident Nonvalvular Atrial Fibrillation Among Patients Who Received at Least 1 Oral Anticoagulant Within the 1-Year Follow-up, Excluding Patients With Preexisting Chronic Oral Anticoagulant Treatment **eTable 13.** Time Trends in Patient Characteristics of Incident Nonvalvular Atrial Fibrillation Among Patients Who First Received Vitamin K Antagonist as Oral Anticoagulant Within the 1-Year Follow-up, Excluding Patients With Preexisting Chronic Oral Anticoagulant Treatment **eTable 14.** Time Trends in Patient Characteristics of Incident Nonvalvular Atrial Fibrillation Among Patients Who First Received Direct Oral Anticoagulant as Oral Anticoagulant Within the 1-Year Follow-up, Excluding Patients With Preexisting Chronic Oral Anticoagulant Treatment **eTable 15.** Risk of Clinical Events Within the 1-Year Follow-up After Incident Nonvalvular Atrial Fibrillation Diagnosis **eTable 16.** Time Trends in Prognosis Within 1 Year After Incident Nonvalvular Atrial Fibrillation Diagnosis, Excluding Patients With Preexisting Chronic Use of Oral Anticoagulants **eTable 17.** Risk of Clinical Events Within the 1-Year Follow-up After Incident Nonvalvular Atrial Fibrillation Diagnosis, Excluding Patients With Preexisting Chronic Oral Anticoagulant Treatment **eTable 18.** Hazard Ratios of Prognosis Within 1 Year After Incident Nonvalvular Atrial Fibrillation Diagnosis Between Cohorts According to Other Adjustment Models **eTable 19.** Hazard Ratios of Prognosis Within 1 Year After Incident Nonvalvular Atrial Fibrillation Diagnosis Between Cohorts According to Other Adjustment Models, Excluding Patients With Preexisting Chronic Oral Anticoagulant Treatment **eTable 20.** Subgroup Analysis of Risk of Ischemic Stroke Within the 1-Year Follow-up After Incident Nonvalvular Atrial Fibrillation Diagnosis **eTable 21.** Subgroup Analysis of Risk of Major Bleeding Within the 1-Year Follow-up After Incident Nonvalvular Atrial Fibrillation Diagnosis **eTable 22.** Subgroup Analysis of Risk of Intracranial Hemorrhage Within the 1-Year Follow-up After Incident Nonvalvular Atrial Fibrillation Diagnosis **eTable 23.** Subgroup Analysis of Risk of Gastrointestinal Bleeding Within the 1-Year Follow-up After Incident Nonvalvular Atrial Fibrillation Diagnosis **eTable 24.** Subgroup Analysis of Risk of All-Cause Mortality Within the 1-Year Follow-up After Incident Nonvalvular Atrial Fibrillation Diagnosis **eTable 25.** Overall Time Trends in Composition of CHA_2_DS_2_-VASc Score and HAS-BLED Score of Patients With Incident Nonvalvular Atrial Fibrillation in the Netherlands **eTable 26.** Time Trends in Composition of CHA_2_DS_2_-VASc Score and HAS-BLED Score of Male Patients With Incident Nonvalvular Atrial Fibrillation in the Netherlands **eTable 27.** Time Trends in Composition of CHA_2_DS_2_-VASc Score and HAS-BLED Score of Female Patients With Incident Nonvalvular Atrial Fibrillation in the Netherlands **eTable 28.** Time Trends in Composition of CHA_2_DS_2_-VASc Score and HAS-BLED Score of Incident Nonvalvular Atrial Fibrillation Patients in the Netherlands, Excluding Patients With Preexisting Chronic Oral Anticoagulant Treatment **eTable 29.** Time Trends in Composition of CHA_2_DS_2_-VASc Score and HAS-BLED Score of Male Patients With Incident Nonvalvular Atrial Fibrillation in the Netherlands, Excluding Patients With Preexisting Chronic Oral Anticoagulant Treatment **eTable 30.** Time Trends in Composition of CHA_2_DS_2_-VASc Score and HAS-BLED Score of Female Patients With Incident Nonvalvular Atrial Fibrillation in the Netherlands, Excluding Patients With Preexisting Chronic Oral Anticoagulant Treatment **eTable 31.** Association Between Baseline CHA_2_DS_2_-VASc Score and 1-Year Risk of Ischemic Stroke **eTable 32.** Total Numbers of Different Diagnosis Records in the Data Source by Calendar Years **eFigure 1.** Illustration of the Calculation of Proportion of Days Covered by Antithrombotic Agents **eFigure 2.** Cumulative Incidence of Ischemic Stroke Within 1 Year After Incident Nonvalvular Atrial Fibrillation Diagnosis **eFigure 3.** Cumulative Incidence of Major Bleeding Within 1 Year After Incident Nonvalvular Atrial Fibrillation Diagnosis **eFigure 4.** Cumulative Incidence of Intracranial Hemorrhage Within 1 Year After Incident Nonvalvular Atrial Fibrillation Diagnosis **eFigure 5.** Cumulative Incidence of Gastrointestinal Bleeding Within 1 Year After Incident Nonvalvular Atrial Fibrillation Diagnosis **eFigure 6.** Survival Probability Within 1 Year After Incident Nonvalvular Atrial Fibrillation Diagnosis **eFigure 7.** Cumulative Incidence of Ischemic Stroke Within 1 Year After Incident Nonvalvular Atrial Fibrillation Diagnosis, Excluding Patients With Preexisting Chronic Oral Anticoagulant Treatment **eFigure 8.** Cumulative Incidence of Major Bleeding Within 1 Year After Incident Nonvalvular Atrial Fibrillation Diagnosis, Excluding Patients With Preexisting Chronic Oral Anticoagulant Treatment **eFigure 9.** Cumulative Incidence of Intracranial Hemorrhage Within 1 Year After Incident Nonvalvular Atrial Fibrillation Diagnosis, Excluding Patients With Preexisting Chronic Oral Anticoagulant Treatment **eFigure 10.** Cumulative Incidence of Gastrointestinal Bleeding Within 1 Year After Incident Nonvalvular Atrial Fibrillation Diagnosis, Excluding Patients With Preexisting Chronic Oral Anticoagulant Treatment **eFigure 11.** Survival Probability Within 1 Year After Incident Nonvalvular Atrial Fibrillation Diagnosis, Excluding Patients With Preexisting Chronic Oral Anticoagulant Treatment

Supplement 2.
Data Sharing Statement
