# Supplementary material for: Time Trends in Patient Characteristics, Anticoagulation Treatment, and Prognosis of Incident Nonvalvular Atrial Fibrillation in the Netherlands
Source: JAMA Netw Open. 2023 Apr 25;6(4):e239973. doi: 10.1001/jamanetworkopen.2023.9973 (PMC10130953; doi:10.1001/jamanetworkopen.2023.9973)
Supplement: Supplement 1. — eMethods. eTable 1. Codes Used for Variable Identification eTable 2. Time Trends in Other Patient Characteristics of Incident Nonvalvular Atrial Fibrillation eTable 3. Time Trends in Patient Characteristics of Incident Nonvalvular Atrial Fibrillation, Excluding Patients With Preexisting Chronic Oral Anticoagulant Treatment eTable 4. Time Trends in Anticoagulation Treatment Within 1 Year After Incident Nonvalvular Atrial Fibrillation Diagnosis, Excluding Patients With Preexisting Chronic Oral Anticoagulant Treatment eTable 5. Time Trends in Anticoagulation Treatment Within 1 Year After Incident Nonvalvular Atrial Fibrillation, Stratified by Baseline CHA2DS2-VASc Score eTable 6. Time Trends in Anticoagulation Treatment Within 1 Year After Incident Nonvalvular Atrial Fibrillation Diagnosis, Stratified by Baseline CHA2DS2-VASc Score Excluding Patients With Preexisting Chronic Oral Anticoagulant Treatment eTable 7. Time Trends in Patient Characteristics of Incident Nonvalvular Atrial Fibrillation Among Patients Who Did Not Receive Any Oral Anticoagulants Within the 1-Year Follow-up eTable 8. Time Trends in Patient Characteristics of Incident Nonvalvular Atrial Fibrillation Among Patients Who Received at Least 1 Oral Anticoagulant Within the 1-Year Follow-up eTable 9. Time Trends in Patient Characteristics of Incident Nonvalvular Atrial Fibrillation Among Patients Who First Received Vitamin K Antagonist as Oral Anticoagulant Within the 1-Year Follow-up eTable 10. Time Trends in Patient Characteristics of Incident Nonvalvular Atrial Fibrillation Among Patients Who First Received Direct Oral Anticoagulant as Oral Anticoagulant Within the 1-Year Follow-up eTable 11. Time Trends in Patient Characteristics of Incident Nonvalvular Atrial Fibrillation Among Patients Who Did Not Receive Any Oral Anticoagulants Within the 1-Year Follow-up, Excluding Patients With Preexisting Chronic Oral Anticoagulant Treatment eTable 12. Time Trends in Patient Characteristics of Incident Nonvalvu [file jamanetwopen-e239973-s001.pdf]

Supplemental Online Content

Chen Q, Toorop MMA, Tops LF, Lijfering WM, Cannegieter SC. Time trends in patient characteristics, anticoagulation treatment, and prognosis of incident nonvalvular atrial fibrillation in the Netherlands. *JAMA Netw Open*. 2023;6(4):e239973. doi:10.1001/jamanetworkopen.2023.9973

eMethods.
eTable 1. Codes Used for Variable Identification
eTable 2. Time Trends in Other Patient Characteristics of Incident Nonvalvular Atrial Fibrillation
eTable 3. Time Trends in Patient Characteristics of Incident Nonvalvular Atrial Fibrillation, Excluding Patients With Preexisting Chronic Oral Anticoagulant Treatment
eTable 4. Time Trends in Anticoagulation Treatment Within 1 Year After Incident Nonvalvular Atrial Fibrillation Diagnosis, Excluding Patients With Preexisting Chronic Oral Anticoagulant Treatment
eTable 5. Time Trends in Anticoagulation Treatment Within 1 Year After Incident Nonvalvular Atrial Fibrillation, Stratified by Baseline CHA_2DS_2-VASc Score
eTable 6. Time Trends in Anticoagulation Treatment Within 1 Year After Incident Nonvalvular Atrial Fibrillation Diagnosis, Stratified by Baseline CHA_2DS_2-VASc Score Excluding Patients With Preexisting Chronic Oral Anticoagulant Treatment
eTable 7. Time Trends in Patient Characteristics of Incident Nonvalvular Atrial Fibrillation Among Patients Who Did Not Receive Any Oral Anticoagulants Within the 1-Year Follow-up
eTable 8. Time Trends in Patient Characteristics of Incident Nonvalvular Atrial Fibrillation Among Patients Who Received at Least 1 Oral Anticoagulant Within the 1-Year Follow-up
eTable 9. Time Trends in Patient Characteristics of Incident Nonvalvular Atrial Fibrillation Among Patients Who First Received Vitamin K Antagonist as Oral Anticoagulant Within the 1-Year Follow-up
eTable 10. Time Trends in Patient Characteristics of Incident Nonvalvular Atrial Fibrillation Among Patients Who First Received Direct Oral Anticoagulant as Oral Anticoagulant Within the 1-Year Follow-up
eTable 11. Time Trends in Patient Characteristics of Incident Nonvalvular Atrial Fibrillation Among Patients Who Did Not Receive Any Oral Anticoagulants Within the 1-Year Follow-up, Excluding Patients With Preexisting Chronic Oral Anticoagulant Treatment
eTable 12. Time Trends in Patient Characteristics of Incident Nonvalvular Atrial Fibrillation Among Patients Who Received at Least 1 Oral Anticoagulant Within the 1-Year Follow-up, Excluding Patients With Preexisting Chronic Oral Anticoagulant Treatment
eTable 13. Time Trends in Patient Characteristics of Incident Nonvalvular Atrial Fibrillation Among Patients Who First Received Vitamin K Antagonist as Oral Anticoagulant Within the 1-Year Follow-up, Excluding Patients With Preexisting Chronic Oral Anticoagulant Treatment
eTable 14. Time Trends in Patient Characteristics of Incident Nonvalvular Atrial Fibrillation Among Patients Who First Received Direct Oral Anticoagulant as Oral Anticoagulant Within the 1-Year Follow-up, Excluding Patients With Preexisting Chronic Oral Anticoagulant Treatment
eTable 15. Risk of Clinical Events Within the 1-Year Follow-up After Incident Nonvalvular Atrial Fibrillation Diagnosis
eTable 16. Time Trends in Prognosis Within 1 Year After Incident Nonvalvular Atrial Fibrillation Diagnosis, Excluding Patients With Preexisting Chronic Use of Oral Anticoagulants
eTable 17. Risk of Clinical Events Within the 1-Year Follow-up After Incident Nonvalvular Atrial Fibrillation Diagnosis, Excluding Patients With Preexisting Chronic Oral Anticoagulant Treatment

© 2023 Chen Q et al. *JAMA Network Open.*

eTable 18. Hazard Ratios of Prognosis Within 1 Year After Incident Nonvalvular Atrial Fibrillation Diagnosis Between Cohorts According to Other Adjustment Models
eTable 19. Hazard Ratios of Prognosis Within 1 Year After Incident Nonvalvular Atrial Fibrillation Diagnosis Between Cohorts According to Other Adjustment Models, Excluding Patients With Preexisting Chronic Oral Anticoagulant Treatment
eTable 20. Subgroup Analysis of Risk of Ischemic Stroke Within the 1-Year Follow-up After Incident Nonvalvular Atrial Fibrillation Diagnosis
eTable 21. Subgroup Analysis of Risk of Major Bleeding Within the 1-Year Follow-up After Incident Nonvalvular Atrial Fibrillation Diagnosis
eTable 22. Subgroup Analysis of Risk of Intracranial Hemorrhage Within the 1-Year Follow-up After Incident Nonvalvular Atrial Fibrillation Diagnosis
eTable 23. Subgroup Analysis of Risk of Gastrointestinal Bleeding Within the 1-Year Follow-up After Incident Nonvalvular Atrial Fibrillation Diagnosis
eTable 24. Subgroup Analysis of Risk of All-Cause Mortality Within the 1-Year Follow-up After Incident Nonvalvular Atrial Fibrillation Diagnosis
eTable 25. Overall Time Trends in Composition of CHA_2DS_2-VASc Score and HAS-BLED Score of Patients With Incident Nonvalvular Atrial Fibrillation in the Netherlands
eTable 26. Time Trends in Composition of CHA_2DS_2-VASc Score and HAS-BLED Score of Male Patients With Incident Nonvalvular Atrial Fibrillation in the Netherlands
eTable 27. Time Trends in Composition of CHA_2DS_2-VASc Score and HAS-BLED Score of Female Patients With Incident Nonvalvular Atrial Fibrillation in the Netherlands
eTable 28. Time Trends in Composition of CHA_2DS_2-VASc Score and HAS-BLED Score of Incident Nonvalvular Atrial Fibrillation Patients in the Netherlands, Excluding Patients With Preexisting Chronic Oral Anticoagulant Treatment
eTable 29. Time Trends in Composition of CHA_2DS_2-VASc Score and HAS-BLED Score of Male Patients With Incident Nonvalvular Atrial Fibrillation in the Netherlands, Excluding Patients With Preexisting Chronic Oral Anticoagulant Treatment
eTable 30. Time Trends in Composition of CHA_2DS_2-VASc Score and HAS-BLED Score of Female Patients With Incident Nonvalvular Atrial Fibrillation in the Netherlands, Excluding Patients With Preexisting Chronic Oral Anticoagulant Treatment
eTable 31. Association Between Baseline CHA_2DS_2-VASc Score and 1-Year Risk of Ischemic Stroke
eTable 32. Total Numbers of Different Diagnosis Records in the Data Source by Calendar Years
eFigure 1. Illustration of the Calculation of Proportion of Days Covered by Antithrombotic Agents
eFigure 2. Cumulative Incidence of Ischemic Stroke Within 1 Year After Incident Nonvalvular Atrial Fibrillation Diagnosis
eFigure 3. Cumulative Incidence of Major Bleeding Within 1 Year After Incident Nonvalvular Atrial Fibrillation Diagnosis
eFigure 4. Cumulative Incidence of Intracranial Hemorrhage Within 1 Year After Incident Nonvalvular Atrial Fibrillation Diagnosis
eFigure 5. Cumulative Incidence of Gastrointestinal Bleeding Within 1 Year After Incident Nonvalvular Atrial Fibrillation Diagnosis
eFigure 6. Survival Probability Within 1 Year After Incident Nonvalvular Atrial Fibrillation Diagnosis
eFigure 7. Cumulative Incidence of Ischemic Stroke Within 1 Year After Incident Nonvalvular Atrial Fibrillation Diagnosis, Excluding Patients With Preexisting Chronic Oral Anticoagulant Treatment
eFigure 8. Cumulative Incidence of Major Bleeding Within 1 Year After Incident Nonvalvular Atrial Fibrillation Diagnosis, Excluding Patients With Preexisting Chronic Oral Anticoagulant Treatment
eFigure 9. Cumulative Incidence of Intracranial Hemorrhage Within 1 Year After Incident Nonvalvular Atrial Fibrillation Diagnosis, Excluding Patients With Preexisting Chronic Oral Anticoagulant Treatment

© 2023 Chen Q et al. *JAMA Network Open.*

eFigure 10. Cumulative Incidence of Gastrointestinal Bleeding Within 1 Year After Incident Nonvalvular Atrial Fibrillation Diagnosis, Excluding Patients With Preexisting Chronic Oral Anticoagulant Treatment
eFigure 11. Survival Probability Within 1 Year After Incident Nonvalvular Atrial Fibrillation Diagnosis, Excluding Patients With Preexisting Chronic Oral Anticoagulant Treatment

This supplemental material has been provided by the authors to give readers additional information about their work.

eMethods.

Details about the data sources

The study used data accessed from Statistics Netherlands ("Centraal Bureau voor de Statistiek", CBS), which is a Dutch governmental institution that gathers and links de-identified individual data from various nationwide data sources. In the study we used the following data between 2010 and 2019: 1) data on diagnoses registered within hospitalizations, which came from the Dutch Hospital Data (DHD) registry, covering all general and academic Dutch hospitals and two short-stay categorical hospitals (*i.e.*, a cancer clinic and an eye hospital); 2) data on mortality (including date of death and primary cause of death), which came from the Personal Records Database ("Basisregistratie Personen", BRP) and the nationwide Dutch Registry of Causes of Death statistics, covering all deceased Dutch inhabitants; 3) data on personal characteristics (including standardized household income), which came from the BRP and the Tax and Customs administration and student grant registrations of the Education Executive Agency ("Dienst Uitvoering Onderwijs", DUO), covering all Dutch inhabitants; 4) data on outpatient medication prescriptions, which came from the Dutch Healthcare Insurance Board ("College voor Zorgverzekeringen", CVZ), covering all individuals under the Basic Dutch Health Insurance (which is compulsory for all Dutch inhabitants).

Among the data we obtained, the data on diagnoses registered within hospitalizations only provided the diagnoses extracted from discharge letters, but there was a variable (referred to as "main diagnosis") indicating which diagnosis was the main reason for the corresponding hospital admission, and another variable (referred to as "primary diagnosis") indicating whether a diagnosis was the main reason for providing the corresponding care. One of the "primary diagnoses" would be the "main diagnosis", and the remaining ones could be seen as ancillary (but relevant) diagnoses. In addition, the data also provided the main procedure (if any) a patient received during a hospitalization. A low proportion of the diagnoses were imputed by CBS, and in the study we did not distinguish whether a diagnosis was imputed or not.

There were some changes in the collection of the data on diagnoses registered within hospitalizations, generally leading to an improvement in data quality[1]. Up to and including the registration year 2013, the data was collected by an external party on behalf of DHD and from January 1, 2015, DHD has taken over the collection in-house. In 2013, the data source started to be switched from the National Medical Registration ("Landelijke Medische Registratie", LMR) to the National Basic Register Hospital Care ("Landelijke Basisregistratie Ziekenhuiszorg", LBZ), which had been finished since 2014. The transition from the International Classification of Diseases codes [9th Revision (ICD-9)] to the 10th Revision (ICD-10) started from registration year 2011. In the data we obtained, ICD-10 was completely available in data registered from 2013. In addition, the definitions of "admission" were modified several times. With effect from registration year 2012, the definition of clinical admissions and day admissions according to the Dutch Healthcare Authority ("Nederlandse Zorgautoriteit", NZa) was followed in the LMR. Some of the hospitals delivered data directly in accordance with the new definition, and from 2014 all hospitals did. Due to the change in definition, certain recordings that were previously registered as clinical admissions were no longer included, leading to a decrease in the total number of clinical admissions. In 2014, the NZa further clarified the definition of a day admission. As a result, many day admissions were no longer registered as such, resulting in a decrease in the total number of day admissions. With effect from the registration year 2015, long-term observation was added as a type of care.

The data on outpatient medication prescriptions we obtained included all medications covered by the Basic Dutch Health Insurance and dispensed by outpatient hospital pharmacies (including those dispensed to the elderly in residential homes), but did not include medications dispensed within hospitals or nursing homes.

In data on prescription of antithrombotic agents, detailed date and the general type (*i.e.*, vitamin K antagonist (VKA), direct oral anticoagulant (DOAC), heparin group, and antiplatelet agent) of each prescription were provided, but the detailed amount and more specific type of the medication were unavailable. In data on prescription of other medications, we only knew whether an individual received ≥1 prescription within a calendar year without knowing the detailed date or amount of the prescription.

Unless otherwise stated, in the study we identified diseases by ICD-10 (or ICD-9 when ICD-10 was unavailable, *i.e.*, for diagnoses made before 2013), medications by the codes from the Anatomic-Therapeutic-Chemical (ATC) system of the World Health Organization, and procedures by the specific coding system used in the data. Detailed codes are presented in eTable 1.

Detailed inclusion criteria of the study population

The study population consisted of incident non-valvular atrial fibrillation (NVAF) patients initially recognised within a hospitalization between 2014 and 2018 who were not primarily admitted for ischemic stroke. In detail, the study population was identified according to the below criteria: 1) ≥1 diagnosis record of atrial fibrillation between 1/1/2014 and 31/12/2018 (inclusive, based on dates of admission, and the admission date of the hospitalization in which the incident atrial fibrillation diagnosis was made was referred to as the index date); 2) without any diagnosis record of atrial fibrillation within four years (*i.e.*, 1,461 days, where one year was counted as 365.25 days) before the index dates (exclusive); 3) survived at least until the index dates (inclusive); 4)

without any diagnosis record of valvular heart disease (*i.e.*, rheumatic mitral stenosis, or mechanical heart valves) within four years before the index dates (inclusive); 5) aged ≥18 years on the index dates; 6) without any outpatient prescription record of antiarrhythmics (class I or III) in the previous one calendar year of the index dates; 7) without any atrial fibrillation relevant procedure record (*i.e.*, cardioversion, ablation, and left atrial appendage occlusion) within one year before the index dates (exclusive); 8) the main reason for the admission to the index hospitalization (*i.e.*, in which the incident NVAF diagnosis was made) was not ischemic stroke (according to the variable "main diagnosis").

Details about the investigated baseline patient characteristics

The study investigated the following patient characteristics at baseline (*i.e.*, on the index dates): age, sex, immigration background, standardized household income, marital status, type of atrial fibrillation (only available for diagnosis made after 2015), the CHA_2DS_2-VASc score[2], the HAS-BLED score (modified, without including the item labile international normalized ratio)[3], pre-existing chronic use of antithrombotic agents (*i.e.*, defined as ≥2 outpatient prescription records of the same type of antithrombotic agent within six months (*i.e.*, 183 days) before the index dates (exclusive)), and various comorbidities (or medical history), including asthma, chronic obstructive pulmonary disease, other chronic lung diseases, heart failure, myocardial infarction (history), hypertension, other valvular heart disease, peripheral artery disease, liver diseases, gastroesophageal reflux disease, peptic ulcer disease, chronic kidney diseases, anaemia, coagulopathy, diabetes, thyroid disease, ischemic stroke (history), transient ischemic attack (TIA), other arterial thromboembolism, Parkinson's disease, Alzheimer's disease, autoimmune disease, systemic connective tissue disorders, venous thromboembolism, deep vein thrombosis, pulmonary embolism, major bleeding, intracranial haemorrhage (ICH), gastrointestinal bleeding, and malignant tumour.

The CHA_2DS_2-VASc score was calculated using relevant diagnoses data within four years before the index dates (inclusive), while the HAS-BLED score was calculated using relevant data on diagnoses and medication within one year before index dates (inclusive). The various comorbidities were identified using diagnosis data within four years before the index dates (inclusive), but we also specially identified these conditions without including the index hospitalizations, or only within the index hospitalizations, respectively; for venous thromboembolism, deep vein thrombosis, and pulmonary embolism, we identified them using diagnoses data between four years and six months (exclusive) before the index dates, and within six months before the index dates (inclusive), respectively.

In addition, we identified the below variables: 1) ≥1 hospitalization within the prior four years; 2) length of hospital stay and in-hospital mortality of the index hospitalization; 3) the main admission reason for the index hospitalization (according to the variable "main diagnosis"), including atrial fibrillation, heart failure, ischemic heart diseases, respiratory diseases, injury/poisoning, symptoms/signs/abnormal laboratory findings (except for abnormalities of heart beat, or cardiac murmurs and other cardiac sounds), neoplasms, digestive diseases, other heart valve disorders (except for endocarditis or unspecified valve diseases), genitourinary diseases, infectious/parasitic diseases, endocrine/nutritional/metabolic diseases, diseases of blood/blood-forming organs, and neurological disorders (except for TIA or vascular syndromes of brain in cerebrovascular diseases).

Details about the calculation of proportion of days covered (PDC) by antithrombotic agents

Since the amount of each prescription was unknown, the calculation of PDCs was under the assumption that each antithrombotic agent was prescribed for 90 days (i.e., the maximum amount of chronically used medication allowed to be prescribed in The Netherlands) unless there was a refill, a prescription of another type of antithrombotic agents, death, or end of follow-up within 90 days. As illustrated in eFigure 1, with this assumption we calculated PDCs by dividing the length of periods covered by the studied antithrombotic agents by the total length of follow-up. Antiplatelet agent prescriptions were not taken into account when determining PDCs by OACs/heparin group or OACs only, and vice versa.

Details about the identification of the investigated clinical outcomes

Except for all-cause mortality, the other outcome events were identified by examining data on diagnoses (restricting to diagnoses indicated as a main or primary diagnosis) and data on primary cause of death. The admission date of the first outcome event (when it was identified by data on diagnoses), or the date of death (when it was identified by data on primary cause of death) would be considered as the date when the outcome event occurred. However, for an outcome event that occurred exactly within the index hospitalization, we used half of the length of hospital stay as the time to event.

Prespecified adjustment models

When estimating the hazard ratios (HRs) of the studied clinical outcomes between cohorts, besides a crude model, the below adjustment models were prespecified: 1) Model 1: adjusting for age and sex; 2) Model 2: adjusting for model 1, immigration background, standard household income, and type of atrial fibrillation; 3) Model 3: adjusting for model 2, CHA_2DS_2-VASc score, and HAS-BLED score; 4) Model 4: adjusting for model 2, and pre-existing chronic use of antithrombotic agents; 5) Model 5: adjusting for model 2, and the investigated comorbidities (except for deep vein thrombosis, pulmonary embolism, ICH, and gastrointestinal bleeding) identified within four years before the index dates (inclusive); 6) Model 6: the same as Model 5, but the

comorbidities were identified within for years before the index dates (exclusive) and within the index hospitalization, respectively (for venous thromboembolism, identified between four years and six months (exclusive) before the index dates, and within six months before the index dates (inclusive), respectively).

References for the eMethods

[1] Data DH. [DE LMR/LBZ DOOR DE JAREN HEEN]. https://www.dhd.nl/over-ons/10jaardhd/Paginas/10-jaar-LBZ.aspx (April 24 2022)

[2] Lip GY, Nieuwlaat R, Pisters R, Lane DA, Crijns HJ. Refining clinical risk stratification for predicting stroke and thromboembolism in atrial fibrillation using a novel risk factor-based approach: the euro heart survey on atrial fibrillation. Chest 2010;137:263-272. doi: 10.1378/chest.09-1584

[3] Pisters R, Lane DA, Nieuwlaat R, et al. A novel user-friendly score (HAS-BLED) to assess 1-year risk of major bleeding in patients with atrial fibrillation: the Euro Heart Survey. Chest 2010;138:1093-1100. doi: 10.1378/chest.10-0134

© 2023 Chen Q et al. *JAMA Network Open.*

eTable 1. Codes Used for Variable Identification

Disease/Medication/Procedure	Type of code	Code(s)
**For identification of the study population**		
Atrial fibrillation (including atrial and flutter)	ICD-10	I48
	ICD-9	4273
Rheumatic mitral stenosis or mechanical heart valves	ICD-10	I050, I052, I059, Z952
	ICD-9	3940, 3942, 3949, V433
Antiarrhythmics (class I or III)	ATC	C01B
**Atrial fibrillation relevant procedures**		
Cardioversion	CBV	333280A, 333280B, 333289B
	ZA	333280A, 333280B, 333289B
Ablation	CBV	333298F, 333292L, 333292N, 333292K, 333298G, 333298H, 333297U, 333298L, 333298M, 332910A, 333298U, 333298W, 333298S, 333298T, 333298N, 333298P, 333297Z, 333298J, 333298K, 333298Q, 333298R, 333298V, 333297Y, 333297X, 333297W, 333298I, 333298X, 333298Z, 333298Y, 333298E, 333298A, 333298B, 333298D, 333298C, 333292P, 333292F, 333297G, 192305, 192306, 192315, 192316, 192301, 192302, 192013, 192313, 192314, 192309, 192310, 192015, 192303, 192304, 192311, 192312
	ZA	033240, 032940, 032942, 032946, 032944, 032941, 032945, 032987, 333292K, 333298G, 333298H, 333297U, 333298L, 332910A, 333298U, 333298S, 333298N, 333297Z, 333298J, 333298Q, 333297Y, 333297X, 333297W, 333298X, 333298E, 333298A, 333298B, 333298C, 192305, 192306, 192315, 192316, 192301, 192302, 192013, 192313, 192314, 192309, 192310, 192015, 192303, 192304, 192311, 192312
Left atrial appendage occlusion	CBV	033049, 333049, 333137
	ZA	033049, 333049
Ischemic stroke	ICD-10	I63, I64, H341 (Restricted to main diagnosis[†])
**Patient characteristics (at baseline)**		
**Type of atrial fibrillation[‡]**		
Paroxysmal atrial fibrillation	ICD-10	I480
Persistent atrial fibrillation	ICD-10	I481
Chronic atrial fibrillation	ICD-10	I482
Type I atrial flutter	ICD-10	I483
Type II atrial flutter	ICD-10	I484
Unspecified atrial fibrillation	ICD-10	I489
**CHA_2DS_2-VASc score**		
Heart failure	ICD-10	I50
	ICD-9	428
Hypertension	ICD-10	I10, I11, I12, I13, I15

© 2023 Chen Q et al. *JAMA Network Open.*

	ICD-9	401, 402, 403, 404, 405
Diabetes	ICD-10	E10, E11, E12, E13, E14
	ICD-9	250
Stroke/TIA/thromboembolism		
Ischemic stroke (including history)	ICD-10	I63, I64, H341, I693, I694
	ICD-9	434, 436, 3623, 438
TIA	ICD-10	G450, G451, G452, G453, G458, G459
	ICD-9	435
Thromboembolism (Other arterial thromboembolism)	ICD-10	I74
	ICD-9	444
Vascular disease		
Myocardial infarction (including history)	ICD-10	I21, I22, I252
	ICD-9	410, 412
Peripheral artery disease	ICD-10	I739
	ICD-9	4439
Aortic plaque	ICD-10	I70, I250, I251
	ICD-9	440, 4292, 4140
**HAS-BLED score**		
Hypertension (uncontrolled)	ATC	C02, C03, C07, C08, C09 (At least 3 classes of antihypertensive drugs)
Abnormal renal function (Chronic kidney diseases)	ICD-10	E102, E112, E132, E142, I120, M300, M313, M319, M321, N02, N03, N04, N05, N06, N07, N08, N11, N12, N14, N158, N159, N160, N162, N163, N164, N168, N18, N19, N26, Q612, Q613, Q615, Q619
	ICD-9	403, 404, 581, 585, 586, V420, V451, V56
Abnormal liver function (Liver diseases)	ICD-10	B15, B16, B17, B18, B19, C22, D684, I982, K70, K71, K72, K73, K74, K75, K76, K77, Z944
	ICD-9	0702, 0703, 0704, 0705, 0706, 0709, 1550, 4560, 4561, 4562, 5710, 5712, 5713, 5714, 5715, 5718, 5722, 5723, 5724, 5731, 5733, 7824, 7895, V427
Ischemic stroke/TIA (including history)	ICD-10	G450, G451, G452, G453, G458, G459, I63, I64, H341, I693, I694
	ICD-9	435, 434, 436, 3623, 438
Major Bleeding	ICD-10	D62, D683, H313, H356, H431, H450, I312, I60, I61, I62, I850, I9820, I983, J942, K228, K250, K252, K254, K256, K260, K262, K264, K266, K270, K272, K274, K276, K280, K282, K284, K286, K290, K625, K661, K920, K921, K922, N02, R04, R31, R58, S064, S065, S066 (Restricted to primary/main diagnosis[†])
	ICD-9	430, 431, 4320, 4321, 4329, 5310, 5312, 5314, 5316, 5320, 5322, 5324, 5326, 5330, 5332, 5334, 5336, 5340, 5342, 5344, 5346, 5780, 5781, 5789, 5693, 2878, 2879, 5967, 7848, 5997, 6271, 4590, 7191, 7863 (Restricted to primary/main diagnosis[†])
Alcohol abuse	ICD-10	F10, K70, E52, T51, K860, E244, G312, I426, O354, Z714, Z721, G621, G721, K292, Q860, P043
	ICD-9	291, 3050, 3030, 3039, 5710, 5711, 5712, 5713, 9800, 9801, E8600, E8601, E8602, E8609, 4255, 3575, 5353, 6554, 76071

© 2023 Chen Q et al. *JAMA Network Open.*

Antiplatelet agents or NSAIDs	ATC	B01AC, M01A
**Antithrombotic agents**		
Vitamin K antagonist	ATC	B01AA
Direct oral anticoagulant	ATC	B01AE (including B01AF)
Heparin group	ATC	B01AB
Antiplatelet agent	ATC	B01AC
**Comorbidities (or medical history)**		
Asthma	ICD-10	J45, J46
	ICD-9	493
Chronic obstructive pulmonary disease	ICD-10	J44
	ICD-9	496
Other chronic lung diseases	ICD-10	J41, J42, J43, J47, J6, J7
	ICD-9	491, 492, 494, 495, 50
Heart failure	ICD-10	I50
	ICD-9	428
Myocardial infarction (including history)	ICD-10	I21, I22, I252
	ICD-9	410, 412
Hypertension	ICD-10	I10, I11, I12, I13, I15
	ICD-9	401, 402, 403, 404, 405
Other valvular heart disease*	ICD-10	I051, I058, I06, I07, I08, I34, I35, I36, I37, I38, I39
	ICD-9	3941, 395, 396, 397, 424
Peripheral artery disease	ICD-10	I739
	ICD-9	4439
Liver diseases	ICD-10	B15, B16, B17, B18, B19, C22, D684, I982, K70, K71, K72, K73, K74, K75, K76, K77, Z944
	ICD-9	0702, 0703, 0704, 0705, 0706, 0709, 1550, 4560, 4561, 4562, 5710, 5712, 5713, 5714, 5715, 5718, 5722, 5723, 5724, 5731, 5733, 7824, 7895, V427
Gastroesophageal reflux disease	ICD-10	K21
	ICD-9	5305
Peptic ulcer disease	ICD-10	K25, K26, K27
	ICD-9	533, 532, 531
Chronic kidney diseases	ICD-10	E102, E112, E132, E142, I120, M300, M313, M319, M321, N02, N03, N04, N05, N06, N07, N08, N11, N12, N14, N158, N159, N160, N162, N163, N164, N168, N18, N19, N26, Q612, Q613, Q615, Q619
	ICD-9	403, 404, 581, 585, 586, V420, V451, V56
Anaemia	ICD-10	D5, D60, D61, D62, D63, D64
	ICD-9	280, 281, 282, 283, 284, 285
Coagulopathy	ICD-10	D65, D66, D67, D68, D69
	ICD-9	286

© 2023 Chen Q et al. *JAMA Network Open.*

Diabetes	ICD-10	E10, E11, E12, E13, E14
	ICD-9	250
Thyroid disease	ICD-10	E00, E01, E02, E03, E04, E05, E06, E07
	ICD-9	240, 241, 242, 243, 244, 245, 246
Ischemic stroke (including history)	ICD-10	I63, I64, H341, I693, I694
	ICD-9	434, 436, 3623, 438
TIA	ICD-10	G450, G451, G452, G453, G458, G459
	ICD-9	435
Other arterial thromboembolism	ICD-10	I74
	ICD-9	444
Parkinson's disease	ICD-10	G20
	ICD-9	3320
Alzheimer's disease	ICD-10	G30
	ICD-9	3310
Autoimmune disease	ICD-10	D8
	ICD-9	279
Systemic connective tissue disorders	ICD-10	M3
	ICD-9	710
Venous thromboembolism	ICD-10	I801, I802, I803, I808, I809, I821, I822, I823, I828, I829, I26
	ICD-9	4511, 45111, 45119, 4512, 4518, 45181, 45189, 4519, 452, 4531, 4532, 4533, 4538, 4539, 4151
Deep vein thrombosis	ICD-10	I801, I802, I803, I808, I809, I821, I822, I823, I828, I829
	ICD-9	4511, 45111, 45119, 4512, 4518, 45181, 45189, 4519, 452, 4531, 4532, 4533, 4538, 4539
Pulmonary embolism	ICD-10	I26
	ICD-9	4151
Major bleeding	ICD-10	D62, D683, H313, H356, H431, H450, I312, I60, I61, I62, I850, I9820, I983, J942, K228, K250, K252, K254, K256, K260, K262, K264, K266, K270, K272, K274, K276, K280, K282, K284, K286, K290, K625, K661, K920, K921, K922, N02, R04, R31, R58, S064, S065, S066
	ICD-9	430, 431, 4320, 4321, 4329, 5310, 5312, 5314, 5316, 5320, 5322, 5324, 5326, 5330, 5332, 5334, 5336, 5340, 5342, 5344, 5346, 5780, 5781, 5789, 5693, 2878, 2879, 5967, 7848, 5997, 6271, 4590, 7191, 7863
Intracranial haemorrhage	ICD-10	I60, I61, I62
	ICD-9	430, 431, 4320, 4321, 4329
Gastrointestinal bleeding	ICD-10	I850, K250, K252, K254, K256, K260, K262, K264, K266, K270, K272, K274, K276, K280, K282, K284, K286, K625, K661, K920, K921, K922, K228, I983, K290
	ICD-9	5310, 5312, 5314, 5316, 5320, 5322, 5324, 5326, 5330, 5332, 5334, 5336, 5340, 5342, 5344, 5346, 5780, 5781, 5789, 5693
Malignant tumour	ICD-10	C
	ICD-9	14, 15, 16, 17, 18, 19, 20

© 2023 Chen Q et al. *JAMA Network Open.*

**Main admission reason for the index hospitalization**		
Atrial fibrillation	ICD-10	I48 (Restricted to main diagnosis[†])
Heart failure	ICD-10	150 (Restricted to main diagnosis[†])
Ischemic heart diseases	ICD-10	I20, I21, I22, I23, I24, I25 (Restricted to main diagnosis[†])
Respiratory diseases	ICD-10	J (Restricted to main diagnosis[†])
Injury/poisoning	ICD-10	S, T (Restricted to main diagnosis[†])
Symptoms/signs/abnormal laboratory findings	ICD-10	R (except for R00 and R01[§]; restricted to main diagnosis[†])
Neoplasms	ICD-10	C, D0, D1, D2, D3, D4 (Restricted to main diagnosis[†])
Digestive diseases	ICD-10	K (Restricted to main diagnosis[‡])
Other heart valve disorders	ICD-10	I34, I35, I36, I37, I39 (except for I398[ǁ]; restricted to main diagnosis[†])
Genitourinary diseases	ICD-10	N (Restricted to main diagnosis[†])
Infectious/parasitic diseases	ICD-10	A, B (Restricted to main diagnosis[†])
Endocrine/nutritional/metabolic diseases	ICD-10	E (Restricted to main diagnosis[†])
Diseases of blood/blood-forming organs	ICD-10	D5, D6, D7, D8 (Restricted to main diagnosis[†])
Neurological disorders	ICD-10	G (except for G45, G46[#]; restricted to main diagnosis[†])
**Treatment during the follow-up**		
**Antithrombotic agents**		
Vitamin K antagonist	ATC	B01AA
Direct oral anticoagulant	ATC	B01AE (including B01AF)
Heparin group	ATC	B01AB
Antiplatelet agent	ATC	B01AC
**Clinical outcomes (prognosis)**		
Ischemic stroke	ICD-10	I63, I64, H341 (Restricted to primary/main diagnosis[†])
Ischemic stroke caused death	ICD-10	I63, I64, H341
Major bleeding	ICD-10	D62, D683, H313, H356, H431, H450, I312, I60, I61, I62, I850, I9820, I983, J942, K228, K250, K252, K254, K256, K260, K262, K264, K266, K270, K272, K274, K276, K280, K282, K284, K286, K290, K625, K661, K920, K921, K922, N02, R04, R31, R58, S064, S065, S066 (Restricted to primary/main diagnosis[†])
Major bleeding caused death	ICD-10	D62, D683, H313, H356, H431, H450, I312, I60, I61, I62, I850, I9820, I983, J942, K228, K250, K252, K254, K256, K260, K262, K264, K266, K270, K272, K274, K276, K280, K282, K284, K286, K290, K625, K661, K920, K921, K922, N02, R04, R31, R58, S064, S065, S066
Intracranial haemorrhage	ICD-10	I60, I61, I62 (Restricted to primary/main diagnosis[†])
Intracranial haemorrhage caused death	ICD-10	I60, I61, I62
Gastrointestinal bleeding	ICD-10	I850, K250, K252, K254, K256, K260, K262, K264, K266, K270, K272, K274, K276, K280, K282, K284, K286, K625, K661, K920, K921, K922, K228, I983, K290 (Restricted to primary/main diagnosis[†])
Gastrointestinal bleeding caused death	ICD-10	I850, K250, K252, K254, K256, K260, K262, K264, K266, K270, K272, K274, K276, K280, K282, K284, K286, K625, K661, K920, K921, K922, K228, I983, K290

© 2023 Chen Q et al. *JAMA Network Open.*

† A main diagnosis refers to the diagnosis which at the end of the hospitalization was considered as the main reason for admission to the hospital, while a primary diagnosis refers to the diagnosis that was considered as the main reason for providing the care by the specialist (who made the diagnosis).
‡ The version of ICD-10 coding system used before 2015 did not include an additional digit to identify (sub)types of atrial fibrillation (*i.e.*, I48 only).
* Except for rheumatic mitral stenosis and mechanical heart valves
§ R00 refers to abnormalities of heart beat, and R01 refers to cardiac murmurs and other cardiac sounds.
|| I398 refers to endocarditis, valve unspecified, in diseases classified elsewhere.
G45 refers to transient cerebral ischaemic attacks and related syndromes, and G46 refers to vascular syndromes of brain in cerebrovascular diseases.
Abbreviations: ICD, International Classification of Diseases; ATC, Anatomic-Therapeutic-Chemical (codes); CBV, Central Management Transaction File (in Dutch “Centraal Beheer Verrichtingenbestand”); ZA, Healthcare Activities (in Dutch “ZorgActiviteiten”); TIA, transient ischemic attack; NSAID, nonsteroidal anti-inflammatory drugs.

© 2023 Chen Q et al. *JAMA Network Open.*

eTable 2. Time Trends in Other Patient Characteristics of Incident Nonvalvular Atrial Fibrillation

Cohort	2014 (N=55,880)	2015 (N=61,317)	2016 (N=60,018)	2017 (N=62,390)	2018 (N=61,696)
Age group, years, n (%)					
18-24	75 (0.1)	76 (0.1)	78 (0.1)	89 (0.1)	92 (0.1)
25-34	217 (0.4)	257 (0.4)	265 (0.4)	269 (0.4)	283 (0.5)
35-44	641 (1.1)	802 (1.3)	725 (1.2)	732 (1.2)	697 (1.1)
45-54	2,655 (4.8)	3,045 (5.0)	2,966 (4.9)	2,995 (4.8)	2,909 (4.7)
55-64	7,425 (13.3)	8,088 (13.2)	8,301 (13.8)	8,610 (13.8)	8,507 (13.8)
65-74	15,590 (27.9)	17,403 (28.4)	17,326 (28.9)	18,286 (29.3)	18,357 (29.8)
75-84	18,257 (32.7)	19,771 (32.2)	19,123 (31.9)	19,709 (31.6)	19,517 (31.6)
≥85	11,020 (19.7)	11,875 (19.4)	11,234 (18.7)	11,700 (18.8)	11,334 (18.4)
Marital status, n (%)					
Married or in partnership	30,975 (55.4)	34,359 (56.0)	33,562 (55.9)	34,781 (55.7)	34,578 (56.0)
Unmarried or single	3,869 (6.9)	4,402 (7.2)	4,617 (7.7)	4,996 (8.0)	4,969 (8.1)
Divorced	5,481 (9.8)	6,100 (9.9)	6,185 (10.3)	6,678 (10.7)	6,745 (10.9)
Widowed	15,555 (27.8)	16,456 (26.8)	15,654 (26.1)	15,935 (25.5)	15,404 (25.0)
CHA_2DS_2-VASc score, n (%)					
0	4,038 (7.2)	4,787 (7.8)	4,790 (8.0)	4,760 (7.6)	4,589 (7.4)
1	7,422 (13.3)	8,309 (13.6)	8,160 (13.6)	8,577 (13.7)	8,565 (13.9)
2	11,044 (19.8)	12,171 (19.8)	12,185 (20.3)	12,485 (20.0)	12,365 (20.0)
3	13,112 (23.5)	14,253 (23.2)	14,005 (23.3)	14,547 (23.3)	14,380 (23.3)
4	10,513 (18.8)	11,398 (18.6)	10,955 (18.3)	11,605 (18.6)	11,562 (18.7)
5	6,023 (10.8)	6,454 (10.5)	6,140 (10.2)	6,457 (10.3)	6,421 (10.4)
6	2,587 (4.6)	2,708 (4.4)	2,645 (4.4)	2,733 (4.4)	2,716 (4.4)
≥7	1,141 (2.0)	1,237 (2.0)	1,138 (1.9)	1,226 (2.0)	1,098 (1.8)
HAS-BLED score[†], n (%)					
0	5,034 (9.0)	6,083 (9.9)	6,227 (10.4)	6,506 (10.4)	6,531 (10.6)
1	17,923 (32.1)	20,476 (33.4)	20,433 (34.0)	21,426 (34.3)	21,440 (34.8)
2	20,011 (35.8)	21,706 (35.4)	20,811 (34.7)	21,490 (34.4)	21,309 (34.5)
≥3	12,912 (23.1)	13,052 (21.3)	12,547 (20.9)	12,968 (20.8)	12,416 (20.1)
Comorbidities (or medical history)[‡], n (%)					
**Asthma**	**1,074 (1.9)**	**1,308 (2.1)**	**1,315 (2.2)**	**1,402 (2.2)**	**1,617 (2.6)**
Prior 4 years	514 (0.9)	663 (1.1)	727 (1.2)	718 (1.2)	866 (1.4)
Index hospitalization	687 (1.2)	837 (1.4)	791 (1.3)	911 (1.5)	1,046 (1.7)
**Chronic obstructive pulmonary disease**	**7,208 (12.9)**	**7,472 (12.2)**	**7,143 (11.9)**	**7,250 (11.6)**	**7,062 (11.4)**
Prior 4 years	3,469 (6.2)	3,844 (6.3)	3,849 (6.4)	4,022 (6.4)	3,861 (6.3)

© 2023 Chen Q et al. *JAMA Network Open.*

Index hospitalization	5,837 (10.4)	6,073 (9.9)	5,713 (9.5)	5,718 (9.2)	5,684 (9.2)
**Other chronic lung diseases**	**1,379 (2.5)**	**1,359 (2.2)**	**1,341 (2.2)**	**1,465 (2.3)**	**1,622 (2.6)**
Prior 4 years	707 (1.3)	640 (1.0)	643 (1.1)	647 (1.0)	676 (1.1)
Index hospitalization	748 (1.3)	835 (1.4)	811 (1.4)	973 (1.6)	1,116 (1.8)
**Heart failure**	**11,741 (21.0)**	**12,504 (20.4)**	**12,133 (20.2)**	**12,651 (20.3)**	**12,749 (20.7)**
Prior 4 years	3,196 (5.7)	3,329 (5.4)	3,311 (5.5)	3,338 (5.4)	3,307 (5.4)
Index hospitalization	9,984 (17.9)	10,705 (17.5)	10,407 (17.3)	10,882 (17.4)	11,043 (17.9)
**Myocardial infarction (history)**	**6,667 (11.9)**	**7,184 (11.7)**	**7,353 (12.3)**	**7,811 (12.5)**	**7,546 (12.2)**
Prior 4 years	4,544 (8.1)	4,845 (7.9)	4,972 (8.3)	5,361 (8.6)	5,078 (8.2)
Index hospitalization	2,552 (4.6)	2,899 (4.7)	2,997 (5.0)	3,190 (5.1)	3,216 (5.2)
**Hypertension**	**19,963 (35.7)**	**22,062 (36.0)**	**21,706 (36.2)**	**23,432 (37.6)**	**23,792 (38.6)**
Prior 4 years	7,638 (13.7)	9,102 (14.8)	9,424 (15.7)	10,387 (16.6)	10,528 (17.1)
Index hospitalization	16,265 (29.1)	17,925 (29.2)	17,379 (29.0)	18,579 (29.8)	19,113 (31.0)
**Other valvular heart disease**[*]	**8,006 (14.3)**	**8,427 (13.7)**	**8,002 (13.3)**	**8,458 (13.6)**	**8,276 (13.4)**
Prior 4 years	3,015 (5.4)	3,328 (5.4)	3,389 (5.6)	3,654 (5.9)	3,605 (5.8)
Index hospitalization	6,639 (11.9)	7,057 (11.5)	6,653 (11.1)	6,875 (11.0)	6,721 (10.9)
**Peripheral artery disease**	**1,617 (2.9)**	**1,878 (3.1)**	**1,722 (2.9)**	**1,816 (2.9)**	**1,761 (2.9)**
Prior 4 years	810 (1.4)	981 (1.6)	1,037 (1.7)	1,088 (1.7)	1,038 (1.7)
Index hospitalization	1,025 (1.8)	1,212 (2.0)	994 (1.7)	1,023 (1.6)	986 (1.6)
**Liver diseases**	**1,545 (2.8)**	**1,613 (2.6)**	**1,625 (2.7)**	**1,645 (2.6)**	**1,627 (2.6)**
Prior 4 years	644 (1.2)	701 (1.1)	758 (1.3)	773 (1.2)	808 (1.3)
Index hospitalization	1,052 (1.9)	1,058 (1.7)	1,037 (1.7)	1,051 (1.7)	1,036 (1.7)
**Gastroesophageal reflux disease**	**262 (0.5)**	**305 (0.5)**	**360 (0.6)**	**426 (0.7)**	**394 (0.6)**
Prior 4 years	151 (0.3)	219 (0.4)	256 (0.4)	309 (0.5)	289 (0.5)
Index hospitalization	117 (0.2)	92 (0.2)	114 (0.2)	127 (0.2)	111 (0.2)
**Peptic ulcer disease**	**473 (0.8)**	**456 (0.7)**	**448 (0.7)**	**452 (0.7)**	**444 (0.7)**
Prior 4 years	245 (0.4)	222 (0.4)	241 (0.4)	240 (0.4)	221 (0.4)
Index hospitalization	239 (0.4)	244 (0.4)	212 (0.4)	226 (0.4)	235 (0.4)
**Chronic kidney diseases**	**7,398 (13.2)**	**8,059 (13.1)**	**8,422 (14.0)**	**8,886 (14.2)**	**8,421 (13.6)**
Prior 4 years	3,014 (5.4)	3,456 (5.6)	3,717 (6.2)	3,965 (6.4)	3,904 (6.3)
Index hospitalization	5,808 (10.4)	6,304 (10.3)	6,516 (10.9)	6,896 (11.1)	6,585 (10.7)
**Anaemia**	**6,191 (11.1)**	**6,764 (11.0)**	**6,915 (11.5)**	**7,403 (11.9)**	**7,577 (12.3)**
Prior 4 years	3,355 (6.0)	3,688 (6.0)	3,709 (6.2)	3,949 (6.3)	4,013 (6.5)
Index hospitalization	3,527 (6.3)	3,868 (6.3)	4,015 (6.7)	4,390 (7.0)	4,546 (7.4)
**Coagulopathy**	**1,047 (1.9)**	**1,486 (2.4)**	**1,480 (2.5)**	**1,602 (2.6)**	**1,744 (2.8)**
Prior 4 years	345 (0.6)	511 (0.8)	574 (1.0)	630 (1.0)	653 (1.1)
Index hospitalization	749 (1.3)	1,036 (1.7)	982 (1.6)	1,044 (1.7)	1,179 (1.9)

© 2023 Chen Q et al. *JAMA Network Open.*

**Diabetes**	**11,112 (19.9)**	**11,866 (19.4)**	**11,710 (19.5)**	**12,241 (19.6)**	**11,992 (19.4)**
Prior 4 years	5,348 (9.6)	6,064 (9.9)	6,174 (10.3)	6,665 (10.7)	6,392 (10.4)
Index hospitalization	9,242 (16.5)	10,024 (16.3)	9,944 (16.6)	10,308 (16.5)	10,341 (16.8)
**Thyroid disease**	**1,861 (3.3)**	**1,978 (3.2)**	**1,963 (3.3)**	**2,156 (3.5)**	**2,083 (3.4)**
Prior 4 years	590 (1.1)	648 (1.1)	730 (1.2)	805 (1.3)	757 (1.2)
Index hospitalization	1,408 (2.5)	1,504 (2.5)	1,409 (2.3)	1,580 (2.5)	1,570 (2.5)
**Ischemic stroke (history)**	**2,549 (4.6)**	**2,595 (4.2)**	**2,415 (4.0)**	**2,496 (4.0)**	**2,411 (3.9)**
Prior 4 years	1,613 (2.9)	1,759 (2.9)	1,687 (2.8)	1,792 (2.9)	1,685 (2.7)
Index hospitalization	1,255 (2.2)	1,143 (1.9)	972 (1.6)	990 (1.6)	1,047 (1.7)
**TIA**	**1,411 (2.5)**	**1,463 (2.4)**	**1,469 (2.4)**	**1,453 (2.3)**	**1,360 (2.2)**
Prior 4 years	793 (1.4)	832 (1.4)	804 (1.3)	860 (1.4)	778 (1.3)
Index hospitalization	650 (1.2)	671 (1.1)	716 (1.2)	636 (1.0)	627 (1.0)
**Other arterial thromboembolism**	**788 (1.4)**	**768 (1.3)**	**716 (1.2)**	**714 (1.1)**	**607 (1.0)**
Prior 4 years	593 (1.1)	541 (0.9)	509 (0.8)	474 (0.8)	398 (0.6)
Index hospitalization	232 (0.4)	275 (0.4)	237 (0.4)	268 (0.4)	222 (0.4)
**Parkinson's disease**	**557 (1.0)**	**656 (1.1)**	**605 (1.0)**	**685 (1.1)**	**624 (1.0)**
Prior 4 years	263 (0.5)	323 (0.5)	281 (0.5)	350 (0.6)	285 (0.5)
Index hospitalization	429 (0.8)	514 (0.8)	496 (0.8)	557 (0.9)	528 (0.9)
**Alzheimer's disease**	**610 (1.1)**	**664 (1.1)**	**640 (1.1)**	**649 (1.0)**	**664 (1.1)**
Prior 4 years	200 (0.4)	201 (0.3)	186 (0.3)	183 (0.3)	150 (0.2)
Index hospitalization	506 (0.9)	573 (0.9)	559 (0.9)	575 (0.9)	616 (1.0)
**Autoimmune disease**	**241 (0.4)**	**227 (0.4)**	**303 (0.5)**	**345 (0.6)**	**342 (0.6)**
Prior 4 years	158 (0.3)	149 (0.2)	229 (0.4)	241 (0.4)	248 (0.4)
Index hospitalization	106 (0.2)	105 (0.2)	117 (0.2)	154 (0.2)	139 (0.2)
**Systemic connective tissue disorders**	**686 (1.2)**	**843 (1.4)**	**832 (1.4)**	**868 (1.4)**	**924 (1.5)**
Prior 4 years	312 (0.6)	394 (0.6)	481 (0.8)	515 (0.8)	469 (0.8)
Index hospitalization	466 (0.8)	601 (1.0)	538 (0.9)	528 (0.8)	640 (1.0)
**Venous thromboembolism**	**1,186 (2.1)**	**1,213 (2.0)**	**1,268 (2.1)**	**1,386 (2.2)**	**1,293 (2.1)**
Prior 4 years to prior 6 months	376 (0.7)	417 (0.7)	454 (0.8)	427 (0.7)	405 (0.7)
Prior 6 months	839 (1.5)	833 (1.4)	854 (1.4)	998 (1.6)	918 (1.5)
**Deep vein thrombosis**	**496 (0.9)**	**504 (0.8)**	**491 (0.8)**	**548 (0.9)**	**495 (0.8)**
Prior 4 years to prior 6 months	165 (0.3)	183 (0.3)	169 (0.3)	178 (0.3)	149 (0.2)
Prior 6 months	338 (0.6)	330 (0.5)	330 (0.5)	380 (0.6)	350 (0.6)
**Pulmonary embolism**	**811 (1.5)**	**820 (1.3)**	**912 (1.5)**	**958 (1.5)**	**903 (1.5)**
Prior 4 years to prior 6 months	252 (0.5)	276 (0.5)	330 (0.5)	303 (0.5)	295 (0.5)
Prior 6 months	579 (1.0)	563 (0.9)	609 (1.0)	676 (1.1)	632 (1.0)
**Major bleeding**	**4,259 (7.6)**	**4,614 (7.5)**	**4,417 (7.4)**	**4,777 (7.7)**	**4,756 (7.7)**

© 2023 Chen Q et al. *JAMA Network Open.*

Prior 4 years	2,287 (4.1)	2,454 (4.0)	2,444 (4.1)	2,617 (4.2)	2,545 (4.1)
Index hospitalization	2,235 (4.0)	2,450 (4.0)	2,248 (3.7)	2,446 (3.9)	2,510 (4.1)
**Intracranial haemorrhage**	**712 (1.3)**	**695 (1.1)**	**602 (1.0)**	**673 (1.1)**	**606 (1.0)**
Prior 4 years	263 (0.5)	262 (0.4)	203 (0.3)	266 (0.4)	224 (0.4)
Index hospitalization	487 (0.9)	481 (0.8)	427 (0.7)	450 (0.7)	420 (0.7)
**Gastrointestinal bleeding**	**1,810 (3.2)**	**1,898 (3.1)**	**1,873 (3.1)**	**2,006 (3.2)**	**2,008 (3.3)**
Prior 4 years	1,095 (2.0)	1,131 (1.8)	1,159 (1.9)	1,222 (2.0)	1,194 (1.9)
Index hospitalization	796 (1.4)	850 (1.4)	802 (1.3)	882 (1.4)	913 (1.5)
**Malignant tumour**	**7,842 (14.0)**	**8,091 (13.2)**	**8,091 (13.5)**	**8,457 (13.6)**	**8,371 (13.6)**
Prior 4 years	5,948 (10.6)	6,165 (10.1)	6,174 (10.3)	6,560 (10.5)	6,423 (10.4)
Index hospitalization	4,364 (7.8)	4,664 (7.6)	4,682 (7.8)	4,830 (7.7)	4,979 (8.1)
Main reason for admission to the index hospitalization					
Heart failure	4,566 (8.2)	4,906 (8.0)	4,767 (7.9)	4,872 (7.8)	4,905 (8.0)
Ischemic heart diseases	4,504 (8.1)	4,544 (7.4)	4,469 (7.4)	4,714 (7.6)	4,831 (7.8)
Respiratory diseases	3,880 (6.9)	4,863 (7.9)	4,641 (7.7)	5,038 (8.1)	5,244 (8.5)
Injury/poisoning	2,972 (5.3)	3,120 (5.1)	3,138 (5.2)	3,209 (5.1)	3,464 (5.6)
Symptoms/signs/abnormal laboratory findings[§]	2,922 (5.2)	2,997 (4.9)	2,761 (4.6)	2,655 (4.3)	2,483 (4.0)
Neoplasms	2,749 (4.9)	2,794 (4.6)	2,722 (4.5)	2,869 (4.6)	2,929 (4.7)
Digestive diseases	2,477 (4.4)	2,470 (4.0)	2,297 (3.8)	2,515 (4.0)	2,396 (3.9)
Other heart valve disorders[‖]	1,477 (2.6)	1,498 (2.4)	1,630 (2.7)	1,623 (2.6)	1,534 (2.5)
Genitourinary diseases	1,469 (2.6)	1,531 (2.5)	1,570 (2.6)	1,672 (2.7)	1,707 (2.8)
Infectious/parasitic diseases	1,059 (1.9)	1,069 (1.7)	1,037 (1.7)	1,204 (1.9)	1,189 (1.9)
Endocrine/nutritional/metabolic diseases	680 (1.2)	670 (1.1)	616 (1.0)	634 (1.0)	721 (1.2)
Diseases of blood/blood-forming organs	492 (0.9)	503 (0.8)	465 (0.8)	514 (0.8)	483 (0.8)
Neurological disorders[#]	491 (0.9)	455 (0.7)	402 (0.7)	441 (0.7)	405 (0.7)

† Labile INR (international normalized ratio) was not included for calculation.
‡ Unless otherwise stated, for each comorbidity (or medical history), prevalence calculated by screening all hospitalizations within the prior four years (including the index hospitalization), all hospitalizations within the prior four years (excluding the index hospitalization), and only the index hospitalization is presented, respectively.
* Except for rheumatic mitral stenosis and mechanical heart valves
§ Except for abnormalities of heart beat (ICD-10 code: R00) and cardiac murmurs and other cardiac sounds (ICD-10 code: R01)
‖ Except for endocarditis, valve unspecified, in diseases classified elsewhere (ICD-10 code: I398)
Except for transient cerebral ischaemic attacks and related syndromes (ICD-10 code: G45) and vascular syndromes of brain in cerebrovascular diseases (ICD-10 code: G46)
Abbreviations: TIA, transient ischemic attack; ICD, International Classification of Diseases.

© 2023 Chen Q et al. *JAMA Network Open.*

eTable 3. Time Trends in Patient Characteristics of Incident Nonvalvular Atrial Fibrillation, Excluding Patients With Preexisting Chronic Oral Anticoagulant Treatment

Cohort	2014 (N=40,663)	2015 (N=44,149)	2016 (N=43,353)	2017 (N=44,417)	2018 (N=42,586)
Age, years, mean ± SD	74.2 ± 12.3	74.0 ± 12.5	73.8 ± 12.4	73.8 ± 12.4	73.6 ± 12.4
Age group, years, n (%)					
18-24	69 (0.2)	71 (0.2)	75 (0.2)	84 (0.2)	85 (0.2)
25-34	195 (0.5)	227 (0.5)	241 (0.6)	252 (0.6)	253 (0.6)
35-44	546 (1.3)	676 (1.5)	616 (1.4)	626 (1.4)	591 (1.4)
45-54	2,090 (5.1)	2,415 (5.5)	2,368 (5.5)	2,359 (5.3)	2,276 (5.3)
55-64	5,642 (13.9)	6,076 (13.8)	6,218 (14.3)	6,430 (14.5)	6,223 (14.6)
65-74	11,120 (27.3)	12,100 (27.4)	12,144 (28.0)	12,680 (28.5)	12,439 (29.2)
75-84	12,859 (31.6)	13,844 (31.4)	13,344 (30.8)	13,529 (30.5)	12,861 (30.2)
≥85	8,142 (20.0)	8,740 (19.8)	8,347 (19.3)	8,457 (19.0)	7,858 (18.5)
Sex, n (%)					
Male	22,407 (55.1)	23,997 (54.4)	23,838 (55.0)	24,609 (55.4)	23,775 (55.8)
Female	18,256 (44.9)	20,152 (45.6)	19,515 (45.0)	19,808 (44.6)	18,811 (44.2)
Immigration background[†], n (%)					
Native Dutch	35,696 (87.8)	38,620 (87.5)	37,686 (86.9)	38,583 (86.9)	36,908 (86.7)
First-generation immigrants	2,848 (7.0)	3,111 (7.0)	3,190 (7.4)	3,320 (7.5)	3,369 (7.9)
Second-generation immigrants	2,119 (5.2)	2,418 (5.5)	2,477 (5.7)	2,514 (5.7)	2,309 (5.4)
Standardized household income[‡], n (%)					
First quintile (0-20%)	7,050 (17.4)	7,740 (17.6)	7,935 (18.3)	8,245 (18.6)	8,095 (19.1)
Second quintile (20-40%)	11,124 (27.4)	12,254 (27.8)	12,021 (27.8)	12,468 (28.1)	11,774 (27.7)
Third quintile (40-60%)	9,392 (23.1)	9,855 (22.4)	9,696 (22.4)	10,095 (22.8)	9,761 (23.0)
Fourth quintile (60-80%)	6,136 (15.1)	6,813 (15.5)	6,603 (15.3)	6,609 (14.9)	6,339 (14.9)
Fifth quintile (80-100%)	4,927 (12.1)	5,464 (12.4)	5,269 (12.2)	5,396 (12.2)	5,091 (12.0)
Private household with an unknown income	18 (0.0)	39 (0.1)	42 (0.1)	32 (0.1)	31 (0.1)
Institutional household	1,937 (4.8)	1,886 (4.3)	1,694 (3.9)	1,452 (3.3)	1,387 (3.3)
Marital status, n (%)					
Married or in partnership	22,303 (54.8)	24,404 (55.3)	23,820 (54.9)	24,366 (54.9)	23,436 (55.0)
Unmarried or single	2,931 (7.2)	3,346 (7.6)	3,547 (8.2)	3,803 (8.6)	3,706 (8.7)
Divorced	4,050 (10.0)	4,479 (10.1)	4,635 (10.7)	4,863 (10.9)	4,845 (11.4)
Widowed	11,379 (28.0)	11,920 (27.0)	11,351 (26.2)	11,385 (25.6)	10,599 (24.9)
Type of atrial fibrillation, n (%)					
Paroxysmal atrial fibrillation (I48.0)	-	7,133 (16.2)	7,005 (16.2)	6,860 (15.4)	6,276 (14.7)
Persistent atrial fibrillation (I48.1)	-	965 (2.2)	1,005 (2.3)	1,047 (2.4)	962 (2.3)

© 2023 Chen Q et al. *JAMA Network Open.*

Chronic atrial fibrillation (I48.2)	-	1,571 (3.6)	1,511 (3.5)	1,489 (3.4)	1,337 (3.1)
Type I atrial flutter (I48.3)	-	275 (0.6)	322 (0.7)	416 (0.9)	464 (1.1)
Type II atrial flutter (I48.4)	-	128 (0.3)	103 (0.2)	101 (0.2)	93 (0.2)
Unspecified atrial fibrillation (I48.9)	-	34,077 (77.2)	33,407 (77.1)	34,504 (77.7)	33,454 (78.6)
Diagnosis made before 2015	40,663 (100.0)	-	-	-	-
CHA_2DS_2-VASc score, mean ± SD	2.95 ± 1.67	2.92 ± 1.68	2.90 ± 1.68	2.92 ± 1.68	2.91 ± 1.67
CHA_2DS_2-VASc score, n (%)					
0	2,925 (7.2)	3,475 (7.9)	3,460 (8.0)	3,420 (7.7)	3,191 (7.5)
1	5,424 (13.3)	5,925 (13.4)	5,860 (13.5)	6,048 (13.6)	5,904 (13.9)
**≥2**	**32,314 (79.5)**	**34,749 (78.7)**	**34,033 (78.5)**	**34,949 (78.7)**	**33,491 (78.6)**
2	8,057 (19.8)	8,591 (19.5)	8,605 (19.8)	8,762 (19.7)	8,417 (19.8)
3	9,533 (23.4)	10,270 (23.3)	10,127 (23.4)	10,380 (23.4)	9,960 (23.4)
4	7,651 (18.8)	8,314 (18.8)	7,991 (18.4)	8,301 (18.7)	7,955 (18.7)
5	4,390 (10.8)	4,695 (10.6)	4,481 (10.3)	4,645 (10.5)	4,450 (10.4)
6	1,865 (4.6)	1,968 (4.5)	1,962 (4.5)	1,944 (4.4)	1,918 (4.5)
≥7	818 (2.0)	911 (2.1)	867 (2.0)	917 (2.1)	791 (1.9)
HAS-BLED score*, mean ± SD	1.79 ± 1.04	1.73 ± 1.04	1.72 ± 1.05	1.71 ± 1.04	1.69 ± 1.04
HAS-BLED score*, n (%)					
0	3,930 (9.7)	4,729 (10.7)	4,835 (11.2)	4,996 (11.2)	4,913 (11.5)
1	12,830 (31.6)	14,438 (32.7)	14,389 (33.2)	14,962 (33.7)	14,413 (33.8)
2	14,359 (35.3)	15,335 (34.7)	14,809 (34.2)	14,990 (33.7)	14,394 (33.8)
≥3	9,544 (23.5)	9,647 (21.9)	9,320 (21.5)	9,469 (21.3)	8,866 (20.8)
Pre-existing chronic use of antithrombotic agents§					
**Oral anticoagulant, n (%)**	**-**	**-**	**-**	**-**	**-**
Vitamin K antagonist, n (%)	-	-	-	-	-
Direct oral anticoagulant, n (%)	-	-	-	-	-
Heparin group, n (%)	472 (1.2)	483 (1.1)	495 (1.1)	424 (1.0)	355 (0.8)
Antiplatelet agent, n (%)	14,543 (35.8)	14,645 (33.2)	14,265 (32.9)	15,076 (33.9)	14,560 (34.2)
Comorbidities (or medical history)‖, n (%)					
**Asthma**	**798 (2.0)**	**959 (2.2)**	**1,001 (2.3)**	**1,037 (2.3)**	**1,158 (2.7)**
Prior 4 years	380 (0.9)	494 (1.1)	550 (1.3)	524 (1.2)	632 (1.5)
Index hospitalization	517 (1.3)	620 (1.4)	612 (1.4)	685 (1.5)	756 (1.8)
**Chronic obstructive pulmonary disease**	**5,304 (13.0)**	**5,467 (12.4)**	**5,308 (12.2)**	**5,337 (12.0)**	**5,128 (12.0)**
Prior 4 years	2,575 (6.3)	2,845 (6.4)	2,874 (6.6)	2,977 (6.7)	2,798 (6.6)
Index hospitalization	4,324 (10.6)	4,478 (10.1)	4,271 (9.9)	4,230 (9.5)	4,133 (9.7)
**Other chronic lung diseases**	**1,082 (2.7)**	**1,028 (2.3)**	**1,055 (2.4)**	**1,190 (2.7)**	**1,267 (3.0)**
Prior 4 years	550 (1.4)	478 (1.1)	483 (1.1)	510 (1.1)	528 (1.2)

© 2023 Chen Q et al. *JAMA Network Open.*

Index hospitalization	594 (1.5)	644 (1.5)	666 (1.5)	803 (1.8)	877 (2.1)
**Heart failure**	**8,501 (20.9)**	**9,206 (20.9)**	**8,945 (20.6)**	**9,090 (20.5)**	**8,986 (21.1)**
Prior 4 years	2,049 (5.0)	2,201 (5.0)	2,247 (5.2)	2,212 (5.0)	2,091 (4.9)
Index hospitalization	7,380 (18.1)	8,026 (18.2)	7,815 (18.0)	7,913 (17.8)	7,921 (18.6)
**Myocardial infarction (history)**	**5,241 (12.9)**	**5,620 (12.7)**	**5,771 (13.3)**	**6,106 (13.7)**	**5,851 (13.7)**
Prior 4 years	3,372 (8.3)	3,546 (8.0)	3,723 (8.6)	3,976 (9.0)	3,727 (8.8)
Index hospitalization	2,250 (5.5)	2,554 (5.8)	2,601 (6.0)	2,780 (6.3)	2,783 (6.5)
**Hypertension**	**14,480 (35.6)**	**16,066 (36.4)**	**15,840 (36.5)**	**16,764 (37.7)**	**16,408 (38.5)**
Prior 4 years	5,551 (13.7)	6,759 (15.3)	7,018 (16.2)	7,558 (17.0)	7,473 (17.5)
Index hospitalization	11,805 (29.0)	13,070 (29.6)	12,673 (29.2)	13,246 (29.8)	13,112 (30.8)
**Other valvular heart disease[#]**	**5,730 (14.1)**	**6,033 (13.7)**	**5,751 (13.3)**	**6,009 (13.5)**	**5,718 (13.4)**
Prior 4 years	2,216 (5.4)	2,465 (5.6)	2,508 (5.8)	2,646 (6.0)	2,545 (6.0)
Index hospitalization	4,764 (11.7)	5,105 (11.6)	4,810 (11.1)	4,921 (11.1)	4,687 (11.0)
**Peripheral artery disease**	**1,171 (2.9)**	**1,402 (3.2)**	**1,268 (2.9)**	**1,305 (2.9)**	**1,261 (3.0)**
Prior 4 years	596 (1.5)	739 (1.7)	752 (1.7)	787 (1.8)	737 (1.7)
Index hospitalization	736 (1.8)	898 (2.0)	749 (1.7)	730 (1.6)	710 (1.7)
**Liver diseases**	**1,215 (3.0)**	**1,251 (2.8)**	**1,308 (3.0)**	**1,302 (2.9)**	**1,259 (3.0)**
Prior 4 years	500 (1.2)	524 (1.2)	585 (1.3)	590 (1.3)	609 (1.4)
Index hospitalization	844 (2.1)	846 (1.9)	868 (2.0)	861 (1.9)	820 (1.9)
**Gastroesophageal reflux disease**	**199 (0.5)**	**237 (0.5)**	**270 (0.6)**	**312 (0.7)**	**287 (0.7)**
Prior 4 years	115 (0.3)	169 (0.4)	180 (0.4)	227 (0.5)	209 (0.5)
Index hospitalization	90 (0.2)	72 (0.2)	96 (0.2)	94 (0.2)	84 (0.2)
**Peptic ulcer disease**	**364 (0.9)**	**350 (0.8)**	**348 (0.8)**	**337 (0.8)**	**329 (0.8)**
Prior 4 years	185 (0.5)	160 (0.4)	176 (0.4)	179 (0.4)	155 (0.4)
Index hospitalization	188 (0.5)	197 (0.4)	177 (0.4)	171 (0.4)	185 (0.4)
**Chronic kidney diseases**	**5,437 (13.4)**	**6,025 (13.6)**	**6,252 (14.4)**	**6,478 (14.6)**	**6,035 (14.2)**
Prior 4 years	2,223 (5.5)	2,565 (5.8)	2,724 (6.3)	2,870 (6.5)	2,813 (6.6)
Index hospitalization	4,264 (10.5)	4,748 (10.8)	4,896 (11.3)	5,057 (11.4)	4,771 (11.2)
**Anaemia**	**4,615 (11.3)**	**5,106 (11.6)**	**5,265 (12.1)**	**5,510 (12.4)**	**5,566 (13.1)**
Prior 4 years	2,495 (6.1)	2,770 (6.3)	2,784 (6.4)	2,877 (6.5)	2,926 (6.9)
Index hospitalization	2,631 (6.5)	2,937 (6.7)	3,099 (7.1)	3,316 (7.5)	3,358 (7.9)
**Coagulopathy**	**728 (1.8)**	**944 (2.1)**	**1,023 (2.4)**	**1,091 (2.5)**	**1,133 (2.7)**
Prior 4 years	233 (0.6)	340 (0.8)	396 (0.9)	423 (1.0)	422 (1.0)
Index hospitalization	529 (1.3)	642 (1.5)	686 (1.6)	720 (1.6)	769 (1.8)
**Diabetes**	**7,886 (19.4)**	**8,374 (19.0)**	**8,456 (19.5)**	**8,680 (19.5)**	**8,335 (19.6)**
Prior 4 years	3,819 (9.4)	4,313 (9.8)	4,487 (10.3)	4,774 (10.7)	4,492 (10.5)
Index hospitalization	6,560 (16.1)	7,125 (16.1)	7,238 (16.7)	7,322 (16.5)	7,200 (16.9)

© 2023 Chen Q et al. *JAMA Network Open.*

**Thyroid disease**	**1,420 (3.5)**	**1,468 (3.3)**	**1,509 (3.5)**	**1,646 (3.7)**	**1,532 (3.6)**
Prior 4 years	440 (1.1)	460 (1.0)	549 (1.3)	599 (1.3)	541 (1.3)
Index hospitalization	1,079 (2.7)	1,140 (2.6)	1,089 (2.5)	1,219 (2.7)	1,167 (2.7)
**Ischemic stroke (history)**	**1,871 (4.6)**	**1,907 (4.3)**	**1,831 (4.2)**	**1,888 (4.3)**	**1,726 (4.1)**
Prior 4 years	1,160 (2.9)	1,282 (2.9)	1,254 (2.9)	1,340 (3.0)	1,176 (2.8)
Index hospitalization	941 (2.3)	854 (1.9)	772 (1.8)	762 (1.7)	790 (1.9)
**TIA**	**1,093 (2.7)**	**1,084 (2.5)**	**1,076 (2.5)**	**1,067 (2.4)**	**946 (2.2)**
Prior 4 years	597 (1.5)	596 (1.3)	568 (1.3)	636 (1.4)	532 (1.2)
Index hospitalization	524 (1.3)	517 (1.2)	545 (1.3)	460 (1.0)	438 (1.0)
**Other arterial thromboembolism**	**571 (1.4)**	**569 (1.3)**	**523 (1.2)**	**527 (1.2)**	**431 (1.0)**
Prior 4 years	413 (1.0)	391 (0.9)	356 (0.8)	334 (0.8)	265 (0.6)
Index hospitalization	186 (0.5)	214 (0.5)	190 (0.4)	213 (0.5)	174 (0.4)
**Parkinson's disease**	**408 (1.0)**	**481 (1.1)**	**467 (1.1)**	**492 (1.1)**	**451 (1.1)**
Prior 4 years	186 (0.5)	234 (0.5)	224 (0.5)	257 (0.6)	217 (0.5)
Index hospitalization	321 (0.8)	383 (0.9)	382 (0.9)	399 (0.9)	375 (0.9)
**Alzheimer's disease**	**481 (1.2)**	**505 (1.1)**	**509 (1.2)**	**497 (1.1)**	**475 (1.1)**
Prior 4 years	160 (0.4)	154 (0.3)	146 (0.3)	148 (0.3)	117 (0.3)
Index hospitalization	400 (1.0)	437 (1.0)	452 (1.0)	441 (1.0)	441 (1.0)
**Autoimmune disease**	**189 (0.5)**	**176 (0.4)**	**230 (0.5)**	**266 (0.6)**	**270 (0.6)**
Prior 4 years	123 (0.3)	115 (0.3)	177 (0.4)	188 (0.4)	203 (0.5)
Index hospitalization	85 (0.2)	81 (0.2)	83 (0.2)	113 (0.3)	105 (0.2)
**Systemic connective tissue disorders**	**516 (1.3)**	**646 (1.5)**	**623 (1.4)**	**645 (1.5)**	**645 (1.5)**
Prior 4 years	230 (0.6)	301 (0.7)	360 (0.8)	384 (0.9)	331 (0.8)
Index hospitalization	356 (0.9)	462 (1.0)	403 (0.9)	390 (0.9)	451 (1.1)
**Venous thromboembolism**	**924 (2.3)**	**930 (2.1)**	**1,008 (2.3)**	**1,088 (2.4)**	**960 (2.3)**
Prior 4 years to prior 6 months	219 (0.5)	247 (0.6)	298 (0.7)	257 (0.6)	203 (0.5)
Prior 6 months	722 (1.8)	707 (1.6)	736 (1.7)	851 (1.9)	768 (1.8)
**Deep vein thrombosis**	**372 (0.9)**	**369 (0.8)**	**395 (0.9)**	**442 (1.0)**	**363 (0.9)**
Prior 4 years to prior 6 months	96 (0.2)	114 (0.3)	123 (0.3)	126 (0.3)	89 (0.2)
Prior 6 months	280 (0.7)	261 (0.6)	275 (0.6)	323 (0.7)	274 (0.6)
**Pulmonary embolism**	**645 (1.6)**	**637 (1.4)**	**722 (1.7)**	**735 (1.7)**	**668 (1.6)**
Prior 4 years to prior 6 months	146 (0.4)	153 (0.3)	202 (0.5)	163 (0.4)	131 (0.3)
Prior 6 months	511 (1.3)	497 (1.1)	538 (1.2)	581 (1.3)	546 (1.3)
**Major bleeding**	**2,923 (7.2)**	**3,179 (7.2)**	**3,089 (7.1)**	**3,298 (7.4)**	**3,188 (7.5)**
Prior 4 years	1,622 (4.0)	1,715 (3.9)	1,725 (4.0)	1,821 (4.1)	1,696 (4.0)
Index hospitalization	1,477 (3.6)	1,658 (3.8)	1,548 (3.6)	1,678 (3.8)	1,680 (3.9)
**Intracranial haemorrhage**	**471 (1.2)**	**487 (1.1)**	**409 (0.9)**	**457 (1.0)**	**374 (0.9)**

© 2023 Chen Q et al. *JAMA Network Open.*

Prior 4 years	192 (0.5)	209 (0.5)	159 (0.4)	196 (0.4)	160 (0.4)
Index hospitalization	299 (0.7)	309 (0.7)	268 (0.6)	291 (0.7)	241 (0.6)
**Gastrointestinal bleeding**	**1,299 (3.2)**	**1,362 (3.1)**	**1,345 (3.1)**	**1,396 (3.1)**	**1,368 (3.2)**
Prior 4 years	801 (2.0)	819 (1.9)	844 (1.9)	853 (1.9)	792 (1.9)
Index hospitalization	551 (1.4)	600 (1.4)	561 (1.3)	613 (1.4)	633 (1.5)
**Malignant tumour**	**5,989 (14.7)**	**6,092 (13.8)**	**6,165 (14.2)**	**6,356 (14.3)**	**6,178 (14.5)**
Prior 4 years	4,534 (11.2)	4,661 (10.6)	4,682 (10.8)	4,943 (11.1)	4,765 (11.2)
Index hospitalization	3,486 (8.6)	3,669 (8.3)	3,767 (8.7)	3,794 (8.5)	3,854 (9.0)
≥1 hospitalization within the prior 4 years, n (%)	26,643 (65.5)	28,059 (63.6)	27,387 (63.2)	28,183 (63.5)	26,302 (61.8)
Information about the index hospitalization					
Length of hospital stay, days, median (IQR)	4 (1-9)	4 (1-8)	4 (1-8)	3 (1-8)	3 (1-8)
In-hospital mortality, n (%)	1,848 (4.5)	2,033 (4.6)	2,029 (4.7)	2,122 (4.8)	2,187 (5.1)
**Main reason for admission to the index hospitalization**					
Atrial fibrillation	13,287 (32.7)	15,636 (35.4)	15,466 (35.7)	15,844 (35.7)	14,512 (34.1)
Heart failure	3,260 (8.0)	3,596 (8.1)	3,521 (8.1)	3,422 (7.7)	3,388 (8.0)
Ischemic heart diseases	3,790 (9.3)	3,825 (8.7)	3,754 (8.7)	3,906 (8.8)	3,981 (9.3)
Respiratory diseases	3,034 (7.5)	3,774 (8.5)	3,657 (8.4)	3,867 (8.7)	3,979 (9.3)
Injury/poisoning	2,076 (5.1)	2,148 (4.9)	2,150 (5.0)	2,195 (4.9)	2,310 (5.4)
Symptoms/signs/abnormal laboratory findings**	2,023 (5.0)	2,055 (4.7)	1,905 (4.4)	1,763 (4.0)	1,589 (3.7)
Neoplasms	2,163 (5.3)	2,120 (4.8)	2,153 (5.0)	2,218 (5.0)	2,203 (5.2)
Digestive diseases	1,849 (4.5)	1,854 (4.2)	1,740 (4.0)	1,866 (4.2)	1,728 (4.1)
Other heart valve disorders§§	1,158 (2.8)	1,188 (2.7)	1,233 (2.8)	1,266 (2.9)	1,139 (2.7)
Genitourinary diseases	1,103 (2.7)	1,161 (2.6)	1,180 (2.7)	1,259 (2.8)	1,254 (2.9)
Infectious/parasitic diseases	821 (2.0)	843 (1.9)	838 (1.9)	926 (2.1)	901 (2.1)
Endocrine/nutritional/metabolic diseases	488 (1.2)	491 (1.1)	464 (1.1)	443 (1.0)	538 (1.3)
Diseases of blood/blood-forming organs	313 (0.8)	310 (0.7)	288 (0.7)	332 (0.7)	290 (0.7)
Neurological disorders##	351 (0.9)	326 (0.7)	279 (0.6)	327 (0.7)	273 (0.6)

† First-generation immigrants refer to persons who were born abroad with at least one parent who was born abroad; second-generation immigrants refer to persons who was born in the Netherlands with at least one parent who was born abroad.

‡ Percentile groups were determined based on disposable income of private households of the complete target population in the database (instead of the study population only).

* Labile INR (international normalized ratio) was not included for calculation.

§ Pre-existing chronic use was defined as ≥2 (outpatient) prescription records of the same type of antithrombotic agents within six months (*i.e.*, 183 days) before the index dates (exclusive). Pre-existing chronic use of oral anticoagulant refers to persons with pre-existing chronic use of vitamin K antagonist or direct oral anticoagulant.

|| Unless otherwise stated, for each comorbidity (or medical history), prevalence calculated by screening all hospitalizations within the prior four years (including the index hospitalization), all hospitalizations within the prior four years (excluding the index hospitalization), and only the index hospitalization is presented, respectively.

Except for rheumatic mitral stenosis and mechanical heart valves

** Except for abnormalities of heart beat (ICD-10 code: R00) and cardiac murmurs and other cardiac sounds (ICD-10 code: R01)

§§ Except for endocarditis, valve unspecified, in diseases classified elsewhere (ICD-10 code: I398)

Except for transient cerebral ischaemic attacks and related syndromes (ICD-10 code: G45) and vascular syndromes of brain in cerebrovascular diseases (ICD-10 code: G46) Abbreviations: SD, standard deviation; TIA, transient ischemic attack; IQR, interquartile range; ICD, International Classification of Diseases.

© 2023 Chen Q et al. *JAMA Network Open.*

eTable 4. Time Trends in Anticoagulation Treatment Within 1 Year After Incident Nonvalvular Atrial Fibrillation Diagnosis, Excluding Patients With Preexisting Chronic Oral Anticoagulant Treatment†

Cohort	2014 (N=40,663)	2015 (N=44,149)	2016 (N=43,353)	2017 (N=44,417)	2018 (N=42,586)
Received ≥1 antithrombotic agent prescription					
Oral anticoagulant, n (%)	24,415 (60.0)	29,122 (66.0)	28,791 (66.4)	28,860 (65.0)	27,558 (64.7)
Vitamin K antagonist, n (%)	21,478 (52.8)	20,734 (47.0)	14,823 (34.2)	10,076 (22.7)	7,019 (16.5)
Direct oral anticoagulant, n (%)	4,110 (10.1)	10,148 (23.0)	15,713 (36.2)	20,200 (45.5)	21,736 (51.0)
Heparin group, n (%)	5,016 (12.3)	4,910 (11.1)	3,929 (9.1)	3,074 (6.9)	2,544 (6.0)
Antiplatelet agent, n (%)	14,353 (35.3)	13,601 (30.8)	13,115 (30.3)	14,275 (32.1)	13,626 (32.0)
Type of first received oral anticoagulant, n (%)					
Vitamin K antagonist	21,227 (52.2)	20,209 (45.8)	14,203 (32.8)	9,518 (21.4)	6,545 (15.4)
Direct oral anticoagulant	3,188 (7.8)	8,913 (20.2)	14,588 (33.6)	19,342 (43.5)	21,013 (49.3)
Without receiving oral anticoagulant	16,248 (40.0)	15,027 (34.0)	14,562 (33.6)	15,557 (35.0)	15,028 (35.3)
Proportion of days covered‡					
Oral anticoagulant or heparin group, %, median (IQR)	49.32 (0-88.22)	61.37 (0-93.15)	64.38 (0-94.79)	66.30 (0-95.65)	68.18 (0-96.16)
Oral anticoagulant, %, median (IQR)	44.93 (0-84.40)	55.34 (0-90.82)	58.44 (0-93.70)	61.64 (0-95.07)	63.84 (0-95.62)
Antiplatelet agent, %, median (IQR)	0 (0-53.56)	0 (0-28.77)	0 (0-28.49)	0 (0-37.81)	0 (0-36.71)

† Pre-existing chronic oral anticoagulant treatment was defined as ≥2 (outpatient) prescription records of the same type of oral anticoagulant (*i.e.*, vitamin K antagonist or direct oral anticoagulant) within six months (*i.e.*, 183 days) before the index dates (exclusive).

‡ When calculating proportion of days covered by oral anticoagulant or heparin group, each patient was followed for one year (or until death if it occurred within one year), and each prescription of vitamin K antagonist, direct oral anticoagulant, or heparin group was assumed to be prescribed for 90 days unless there was a refill within 90 days, regardless of types of anticoagulants. The proportion of days covered by antiplatelet agent was calculated in the same way, but only prescriptions of antiplatelet agent were examined.

Abbreviation: IQR, interquartile range.

© 2023 Chen Q et al. *JAMA Network Open.*

eTable 5. Time Trends in Anticoagulation Treatment Within 1 Year After Incident Nonvalvular Atrial Fibrillation, Stratified by Baseline CHA_2DS_2-VASc Score

Cohort	2014	2015	2016	2017	2018
CHA_2DS_2-VASc =0	(N=4,038)	(N=4,787)	(N=4,790)	(N=4,760)	(N=4,589)
Received ≥1 antithrombotic agent prescription					
Oral anticoagulant, n (%)	2,311 (57.2)	2,880 (60.2)	2,880 (60.1)	2,795 (58.7)	2,780 (60.6)
Vitamin K antagonist, n (%)	1,677 (41.5)	1,369 (28.6)	907 (18.9)	535 (11.2)	366 (8.0)
Direct oral anticoagulant, n (%)	832 (20.6)	1,740 (36.3)	2,156 (45.0)	2,389 (50.2)	2,492 (54.3)
Heparin group, n (%)	415 (10.3)	355 (7.4)	302 (6.3)	267 (5.6)	207 (4.5)
Antiplatelet agent, n (%)	835 (20.7)	658 (13.7)	580 (12.1)	702 (14.7)	617 (13.4)
Type of first received oral anticoagulant, n (%)					
Vitamin K antagonist	1,639 (40.6)	1,304 (27.2)	840 (17.5)	479 (10.1)	328 (7.1)
Direct oral anticoagulant	672 (16.6)	1,576 (32.9)	2,040 (42.6)	2,316 (48.7)	2,452 (53.4)
Without receiving oral anticoagulant	1,727 (42.8)	1,907 (39.8)	1,910 (39.9)	1,965 (41.3)	1,809 (39.4)
Proportion of days covered[†]					
Oral anticoagulant or heparin group, %, median (IQR)	33.15 (0-84.38)	36.99 (0-85.48)	34.79 (0-84.86)	30.96 (0-83.84)	31.23 (0-84.38)
Oral anticoagulant, %, median (IQR)	27.12 (0-81.64)	33.70 (0-84.11)	32.47 (0-83.29)	28.77 (0-82.47)	29.86 (0-83.01)
Antiplatelet agent, %, median (IQR)	0 (0-0)	0 (0-0)	0 (0-0)	0 (0-0)	0 (0-0)
CHA_2DS_2-VASc =1	(N=7,422)	(N=8,309)	(N=8,160)	(N=8,577)	(N=8,565)
Received ≥1 antithrombotic agent prescription					
Oral anticoagulant, n (%)	4,839 (65.2)	5,861 (70.5)	5,745 (70.4)	5,956 (69.4)	5,989 (69.9)
Vitamin K antagonist, n (%)	3,789 (51.1)	3,296 (39.7)	2,251 (27.6)	1,555 (18.1)	1,116 (13.0)
Direct oral anticoagulant, n (%)	1,415 (19.1)	3,042 (36.6)	3,882 (47.6)	4,715 (55.0)	5,102 (59.6)
Heparin group, n (%)	934 (12.6)	911 (11.0)	739 (9.1)	612 (7.1)	531 (6.2)
Antiplatelet agent, n (%)	1,914 (25.8)	1,637 (19.7)	1,491 (18.3)	1,771 (20.6)	1,704 (19.9)
Type of first received oral anticoagulant, n (%)					
Vitamin K antagonist	3,718 (50.1)	3,171 (38.2)	2,130 (26.1)	1,450 (16.9)	1,011 (11.8)
Direct oral anticoagulant	1,121 (15.1)	2,690 (32.4)	3,615 (44.3)	4,506 (52.5)	4,978 (58.1)
Without receiving oral anticoagulant	2,583 (34.8)	2,448 (29.5)	2,415 (29.6)	2,621 (30.6)	2,576 (30.1)
Proportion of days covered[†]					
Oral anticoagulant or heparin group, %, median (IQR)	63.84 (0-90.68)	73.97 (0-93.42)	73.97 (0-93.42)	75.62 (0-93.70)	75.89 (0-93.42)
Oral anticoagulant, %, median (IQR)	57.53 (0-88.22)	70.14 (0-91.51)	70.96 (0-92.33)	73.42 (0-93.15)	73.97 (0-92.88)
Antiplatelet agent, %, median (IQR)	0 (0-24.66)	0 (0-0)	0 (0-0)	0 (0-0)	0 (0-0)
CHA_2DS_2-VASc ≥2	(N=44,420)	(N=48,221)	(N=47,068)	(N=49,053)	(N=48,542)
Received ≥1 antithrombotic agent prescription					
Oral anticoagulant, n (%)	30,755 (69.2)	35,685 (74.0)	35,142 (74.7)	36,393 (74.2)	36,112 (74.4)
Vitamin K antagonist, n (%)	27,713 (62.4)	27,413 (56.8)	20,822 (44.2)	15,684 (32.0)	11,801 (24.3)
Direct oral anticoagulant, n (%)	4,182 (9.4)	10,026 (20.8)	16,266 (34.6)	22,475 (45.8)	25,961 (53.5)

© 2023 Chen Q et al. *JAMA Network Open.*

Heparin group, n (%)	5,633 (12.7)	5,544 (11.5)	4,423 (9.4)	3,522 (7.2)	2,942 (6.1)
Antiplatelet agent, n (%)	13,469 (30.3)	13,279 (27.5)	12,895 (27.4)	13,797 (28.1)	13,473 (27.8)
Type of first received oral anticoagulant, n (%)					
Vitamin K antagonist	27,446 (61.8)	26,912 (55.8)	20,182 (42.9)	15,050 (30.7)	11,228 (23.1)
Direct oral anticoagulant	3,309 (7.4)	8,773 (18.2)	14,960 (31.8)	21,343 (43.5)	24,884 (51.3)
Without receiving oral anticoagulant	13,665 (30.8)	12,536 (26.0)	11,926 (25.3)	12,660 (25.8)	12,430 (25.6)
Proportion of days covered†					
Oral anticoagulant or heparin group, %, median (IQR)	64.38 (0-89.04)	71.78 (15.74-92.33)	73.97 (24.66-93.97)	77.49 (15.34-95.34)	79.73 (15.89-95.62)
Oral anticoagulant, %, median (IQR)	58.63 (0-86.03)	67.40 (0-90.14)	70.68 (0-92.60)	74.79 (0-94.52)	77.81 (0-95.34)
Antiplatelet agent, %, median (IQR)	0 (0-30.14)	0 (0-24.66)	0 (0-24.66)	0 (0-24.66)	0 (0-24.66)

† When calculating proportion of days covered by oral anticoagulant or heparin group, each patient was followed for one year (or until death if it occurred within one year), and each prescription of vitamin K antagonist, direct oral anticoagulant, or heparin group was assumed to be prescribed for 90 days unless there was a refill within 90 days, regardless of types of anticoagulants. The proportion of days covered by antiplatelet agent was calculated in the same way, but only prescriptions of antiplatelet agent were examined.

Abbreviation: IQR, interquartile range.

© 2023 Chen Q et al. *JAMA Network Open.*

eTable 6. Time Trends in Anticoagulation Treatment Within 1 Year After Incident Nonvalvular Atrial Fibrillation Diagnosis, Stratified by Baseline CHA_2DS_2-VASc Score Excluding Patients With Preexisting Chronic Oral Anticoagulant Treatment[†]

Cohort	2014	2015	2016	2017	2018
CHA_2DS_2-VASc =0	(N=2,925)	(N=3,475)	(N=3,460)	(N=3,420)	(N=3,191)
Received ≥1 antithrombotic agent prescription					
Oral anticoagulant, n (%)	1,259 (43.0)	1,641 (47.2)	1,644 (47.5)	1,543 (45.1)	1,463 (45.8)
Vitamin K antagonist, n (%)	927 (31.7)	754 (21.7)	461 (13.3)	270 (7.9)	199 (6.2)
Direct oral anticoagulant, n (%)	433 (14.8)	1,024 (29.5)	1,276 (36.9)	1,337 (39.1)	1,299 (40.7)
Heparin group, n (%)	328 (11.2)	277 (8.0)	234 (6.8)	213 (6.2)	162 (5.1)
Antiplatelet agent, n (%)	750 (25.6)	583 (16.8)	510 (14.7)	640 (18.7)	557 (17.5)
Type of first received oral anticoagulant, n (%)					
Vitamin K antagonist	906 (31.0)	709 (20.4)	414 (12.0)	244 (7.1)	180 (5.6)
Direct oral anticoagulant	353 (12.1)	932 (26.8)	1,230 (35.5)	1,299 (38.0)	1,283 (40.2)
Without receiving oral anticoagulant	1,666 (57.0)	1,834 (52.8)	1,816 (52.5)	1,877 (54.9)	1,728 (54.2)
Proportion of days covered[‡]					
Oral anticoagulant or heparin group, %, median (IQR)	0 (0-68.49)	0 (0-72.05)	0 (0-72.67)	0 (0-68.82)	0 (0-62.90)
Oral anticoagulant, %, median (IQR)	0 (0-61.92)	0 (0-67.26)	0 (0-68.77)	0 (0-63.63)	0 (0-58.08)
Antiplatelet agent, %, median (IQR)	0 (0-16.16)	0 (0-0)	0 (0-0)	0 (0-0)	0 (0-0)
CHA_2DS_2-VASc =1	(N=5,424)	(N=5,925)	(N=5,860)	(N=6,048)	(N=5,904)
Received ≥1 antithrombotic agent prescription					
Oral anticoagulant, n (%)	2,910 (53.7)	3,554 (60.0)	3,519 (60.1)	3,506 (58.0)	3,415 (57.8)
Vitamin K antagonist, n (%)	2,296 (42.3)	1,935 (32.7)	1,264 (21.6)	801 (13.2)	584 (9.9)
Direct oral anticoagulant, n (%)	853 (15.7)	1,943 (32.8)	2,509 (42.8)	2,875 (47.5)	2,958 (50.1)
Heparin group, n (%)	717 (13.2)	664 (11.2)	549 (9.4)	455 (7.5)	396 (6.7)
Antiplatelet agent, n (%)	1,730 (31.9)	1,428 (24.1)	1,341 (22.9)	1,615 (26.7)	1,545 (26.2)
Type of first received oral anticoagulant, n (%)					
Vitamin K antagonist	2,257 (41.6)	1,839 (31.0)	1,177 (20.1)	741 (12.3)	529 (9.0)
Direct oral anticoagulant	653 (12.0)	1,715 (28.9)	2,342 (40.0)	2,765 (45.7)	2,886 (48.9)
Without receiving oral anticoagulant	2,514 (46.3)	2,371 (40.0)	2,341 (39.9)	2,542 (42.0)	2,489 (42.2)
Proportion of days covered[‡]					
Oral anticoagulant or heparin group, %, median (IQR)	32.60 (0-89.04)	49.32 (0-93.97)	51.64 (0-94.30)	48.35 (0-94.52)	45.62 (0-94.52)
Oral anticoagulant, %, median (IQR)	24.66 (0-84.66)	46.58 (0-92.05)	47.26 (0-93.15)	40.82 (0-93.70)	38.77 (0-93.70)
Antiplatelet agent, %, median (IQR)	0 (0-43.84)	0 (0-0)	0 (0-0)	0 (0-24.66)	0 (0-24.66)
CHA_2DS_2-VASc ≥2	(N=32,314)	(N=34,749)	(N=34,033)	(N=34,949)	(N=33,491)
Received ≥1 antithrombotic agent prescription					
Oral anticoagulant, n (%)	20,246 (62.7)	23,927 (68.9)	23,628 (69.4)	23,811 (68.1)	22,680 (67.7)
Vitamin K antagonist, n (%)	18,255 (56.5)	18,045 (51.9)	13,098 (38.5)	9,005 (25.8)	6,236 (18.6)

© 2023 Chen Q et al. *JAMA Network Open.*

Direct oral anticoagulant, n (%)	2,824 (8.7)	7,181 (20.7)	11,928 (35.0)	15,988 (45.7)	17,479 (52.2)
Heparin group, n (%)	3,971 (12.3)	3,969 (11.4)	3,146 (9.2)	2,406 (6.9)	1,986 (5.9)
Antiplatelet agent, n (%)	11,873 (36.7)	11,590 (33.4)	11,264 (33.1)	12,020 (34.4)	11,524 (34.4)
Type of first received oral anticoagulant, n (%)					
Vitamin K antagonist	18,064 (55.9)	17,661 (50.8)	12,612 (37.1)	8,533 (24.4)	5,836 (17.4)
Direct oral anticoagulant	2,182 (6.8)	6,266 (18.0)	11,016 (32.4)	15,278 (43.7)	16,844 (50.3)
Without receiving oral anticoagulant	12,068 (37.3)	10,822 (31.1)	10,405 (30.6)	11,138 (31.9)	10,811 (32.3)
Proportion of days covered‡					
Oral anticoagulant or heparin group, %, median (IQR)	53.66 (0-88.97)	66.85 (0-93.70)	70.41 (0-95.34)	73.70 (0-96.44)	75.69 (0-96.71)
Oral anticoagulant, %, median (IQR)	49.32 (0-85.48)	61.02 (0-91.51)	64.93 (0-94.52)	69.86 (0-95.89)	73.17 (0-96.44)
Antiplatelet agent, %, median (IQR)	0 (0-60)	0 (0-38.90)	0 (0-40)	0 (0-49.32)	0 (0-49.32)

† Pre-existing chronic oral anticoagulant treatment was defined as ≥2 (outpatient) prescription records of the same type of oral anticoagulant (*i.e.*, vitamin K antagonist or direct oral anticoagulant) within six months (*i.e.*, 183 days) before the index dates (exclusive).

‡ When calculating proportion of days covered by oral anticoagulant or heparin group, each patient was followed for one year (or until death if it occurred within one year), and each prescription of vitamin K antagonist, direct oral anticoagulant, or heparin group was assumed to be prescribed for 90 days unless there was a refill within 90 days, regardless of types of anticoagulants. The proportion of days covered by antiplatelet agent was calculated in the same way, but only prescriptions of antiplatelet agent were examined.

Abbreviation: IQR, interquartile range.

© 2023 Chen Q et al. *JAMA Network Open.*

eTable 7. Time Trends in Patient Characteristics of Incident Nonvalvular Atrial Fibrillation Among Patients Who Did Not Receive Any Oral Anticoagulants Within the 1-Year Follow-up

Cohort	2014 (N=17,975)	2015 (N=16,891)	2016 (N=16,251)	2017 (N=17,246)	2018 (N=16,815)
Age, years, mean ± SD	73.7 ± 13.9	73.6 ± 14.6	73.1 ± 14.5	72.9 ± 14.3	72.9 ± 14.2
Age group, years, n (%)					
18-24	60 (0.3)	59 (0.3)	60 (0.4)	65 (0.4)	66 (0.4)
25-34	154 (0.9)	175 (1.0)	180 (1.1)	198 (1.1)	177 (1.1)
35-44	363 (2.0)	415 (2.5)	397 (2.4)	396 (2.3)	369 (2.2)
45-54	1,184 (6.6)	1,275 (7.5)	1,236 (7.6)	1,238 (7.2)	1,174 (7.0)
55-64	2,741 (15.2)	2,509 (14.9)	2,637 (16.2)	2,845 (16.5)	2,805 (16.7)
65-74	4,288 (23.9)	3,730 (22.1)	3,657 (22.5)	4,207 (24.4)	4,179 (24.9)
75-84	4,985 (27.7)	4,452 (26.4)	4,208 (25.9)	4,426 (25.7)	4,337 (25.8)
≥85	4,200 (23.4)	4,276 (25.3)	3,876 (23.9)	3,871 (22.4)	3,708 (22.1)
Sex, n (%)					
Male	10,134 (56.4)	9,575 (56.7)	9,352 (57.5)	9,891 (57.4)	9,652 (57.4)
Female	7,841 (43.6)	7,316 (43.3)	6,899 (42.5)	7,355 (42.6)	7,163 (42.6)
Immigration background[†], n (%)					
Native Dutch	15,754 (87.6)	14,804 (87.6)	14,067 (86.6)	14,884 (86.3)	14,497 (86.2)
First-generation immigrants	1,236 (6.9)	1,159 (6.9)	1,274 (7.8)	1,388 (8.0)	1,377 (8.2)
Second-generation immigrants	985 (5.5)	928 (5.5)	910 (5.6)	974 (5.6)	941 (5.6)
Standardized household income[‡], n (%)					
First quintile (0-20%)	3,568 (19.9)	3,683 (21.9)	3,571 (22.0)	3,896 (22.7)	3,961 (23.6)
Second quintile (20-40%)	4,379 (24.4)	3,977 (23.6)	3,886 (24.0)	4,228 (24.6)	3,991 (23.8)
Third quintile (40-60%)	3,775 (21.0)	3,217 (19.1)	3,223 (19.9)	3,425 (19.9)	3,312 (19.7)
Fourth quintile (60-80%)	2,608 (14.5)	2,441 (14.5)	2,271 (14.0)	2,439 (14.2)	2,389 (14.2)
Fifth quintile (80-100%)	2,264 (12.6)	2,225 (13.2)	2,088 (12.9)	2,194 (12.8)	2,151 (12.8)
Private household with an unknown income	14 (0.1)	17 (0.1)	26 (0.2)	18 (0.1)	22 (0.1)
Institutional household	1,330 (7.4)	1,289 (7.7)	1,144 (7.1)	995 (5.8)	947 (5.6)
Marital status, n (%)					
Married or in partnership	9,494 (52.8)	8,685 (51.4)	8,290 (51.0)	8,925 (51.8)	8,799 (52.3)
Unmarried or single	1,475 (8.2)	1,562 (9.2)	1,715 (10.6)	1,796 (10.4)	1,738 (10.3)
Divorced	1,821 (10.1)	1,776 (10.5)	1,771 (10.9)	1,977 (11.5)	1,933 (11.5)
Widowed	5,185 (28.8)	4,868 (28.8)	4,475 (27.5)	4,548 (26.4)	4,345 (25.8)
Type of atrial fibrillation, n (%)					
Paroxysmal atrial fibrillation (I48.0)	-	2,651 (15.7)	2,440 (15.0)	2,466 (14.3)	2,222 (13.2)
Persistent atrial fibrillation (I48.1)	-	356 (2.1)	363 (2.2)	461 (2.7)	433 (2.6)

© 2023 Chen Q et al. *JAMA Network Open.*

Chronic atrial fibrillation (I48.2)	-	707 (4.2)	638 (3.9)	675 (3.9)	677 (4.0)
Type I atrial flutter (I48.3)	-	93 (0.6)	88 (0.5)	136 (0.8)	162 (1.0)
Type II atrial flutter (I48.4)	-	34 (0.2)	35 (0.2)	28 (0.2)	26 (0.2)
Unspecified atrial fibrillation (I48.9)	-	13,050 (77.3)	12,687 (78.1)	13,480 (78.2)	13,295 (79.1)
Diagnosis made before 2015	17,975 (100.0)	-	-	-	-
CHA_2DS_2-VASc score, mean ± SD	2.88 ± 1.75	2.85 ± 1.80	2.79 ± 1.80	2.80 ± 1.79	2.81 ± 1.78
CHA_2DS_2-VASc score, n (%)					
0	1,727 (9.6)	1,907 (11.3)	1,910 (11.8)	1,965 (11.4)	1,809 (10.8)
1	2,583 (14.4)	2,448 (14.5)	2,415 (14.9)	2,621 (15.2)	2,576 (15.3)
**≥2**	**13,665 (76.0)**	**12,536 (74.2)**	**11,926 (73.4)**	**12,660 (73.4)**	**12,430 (73.9)**
2	3,234 (18.0)	2,757 (16.3)	2,830 (17.4)	3,031 (17.6)	3,036 (18.1)
3	4,019 (22.4)	3,691 (21.9)	3,499 (21.5)	3,611 (20.9)	3,524 (21.0)
4	3,180 (17.7)	3,073 (18.2)	2,797 (17.2)	3,010 (17.5)	2,944 (17.5)
5	1,963 (10.9)	1,766 (10.5)	1,640 (10.1)	1,811 (10.5)	1,775 (10.6)
6	871 (4.8)	837 (5.0)	795 (4.9)	796 (4.6)	794 (4.7)
≥7	398 (2.2)	412 (2.4)	365 (2.2)	401 (2.3)	357 (2.1)
HAS-BLED score*, mean ± SD	1.77 ± 1.09	1.71 ± 1.11	1.68 ± 1.12	1.67 ± 1.10	1.66 ± 1.09
HAS-BLED score*, n (%)					
0	2,109 (11.7)	2,295 (13.6)	2,395 (14.7)	2,514 (14.6)	2,472 (14.7)
1	5,440 (30.3)	5,283 (31.3)	5,145 (31.7)	5,482 (31.8)	5,367 (31.9)
2	6,099 (33.9)	5,444 (32.2)	5,102 (31.4)	5,441 (31.5)	5,449 (32.4)
≥3	4,327 (24.1)	3,869 (22.9)	3,609 (22.2)	3,809 (22.1)	3,527 (21.0)
Pre-existing chronic use of antithrombotic agents§					
**Oral anticoagulant, n (%)**	**1,727 (9.6)**	**1,864 (11.0)**	**1,689 (10.4)**	**1,689 (9.8)**	**1,787 (10.6)**
Vitamin K antagonist, n (%)	1,637 (9.1)	1,705 (10.1)	1,402 (8.6)	1,282 (7.4)	1,181 (7.0)
Direct oral anticoagulant, n (%)	92 (0.5)	166 (1.0)	293 (1.8)	413 (2.4)	613 (3.6)
Heparin group, n (%)	259 (1.4)	241 (1.4)	276 (1.7)	216 (1.3)	156 (0.9)
Antiplatelet agent, n (%)	6,337 (35.3)	5,282 (31.3)	5,069 (31.2)	5,786 (33.5)	5,589 (33.2)
Comorbidities (or medical history)‖, n (%)					
**Asthma**	**354 (2.0)**	**379 (2.2)**	**361 (2.2)**	**389 (2.3)**	**426 (2.5)**
Prior 4 years	185 (1.0)	212 (1.3)	221 (1.4)	212 (1.2)	244 (1.5)
Index hospitalization	198 (1.1)	236 (1.4)	198 (1.2)	239 (1.4)	237 (1.4)
**Chronic obstructive pulmonary disease**	**2,602 (14.5)**	**2,377 (14.1)**	**2,196 (13.5)**	**2,263 (13.1)**	**2,163 (12.9)**
Prior 4 years	1,315 (7.3)	1,343 (8.0)	1,251 (7.7)	1,399 (8.1)	1,265 (7.5)
Index hospitalization	2,051 (11.4)	1,929 (11.4)	1,734 (10.7)	1,720 (10.0)	1,670 (9.9)
**Other chronic lung diseases**	**671 (3.7)**	**656 (3.9)**	**609 (3.7)**	**670 (3.9)**	**726 (4.3)**
Prior 4 years	293 (1.6)	265 (1.6)	239 (1.5)	250 (1.4)	250 (1.5)

© 2023 Chen Q et al. *JAMA Network Open.*

Index hospitalization	412 (2.3)	447 (2.6)	419 (2.6)	476 (2.8)	538 (3.2)
**Heart failure**	**3,456 (19.2)**	**3,341 (19.8)**	**3,202 (19.7)**	**3,243 (18.8)**	**3,242 (19.3)**
Prior 4 years	1,031 (5.7)	1,044 (6.2)	1,000 (6.2)	985 (5.7)	1,005 (6.0)
Index hospitalization	2,909 (16.2)	2,818 (16.7)	2,700 (16.6)	2,753 (16.0)	2,741 (16.3)
**Myocardial infarction (history)**	**2,435 (13.5)**	**2,251 (13.3)**	**2,258 (13.9)**	**2,474 (14.3)**	**2,391 (14.2)**
Prior 4 years	1,638 (9.1)	1,524 (9.0)	1,514 (9.3)	1,723 (10.0)	1,659 (9.9)
Index hospitalization	971 (5.4)	928 (5.5)	975 (6.0)	1,006 (5.8)	1,005 (6.0)
**Hypertension**	**6,121 (34.1)**	**5,754 (34.1)**	**5,438 (33.5)**	**6,046 (35.1)**	**6,014 (35.8)**
Prior 4 years	2,620 (14.6)	2,734 (16.2)	2,783 (17.1)	3,095 (17.9)	3,127 (18.6)
Index hospitalization	4,766 (26.5)	4,532 (26.8)	4,106 (25.3)	4,447 (25.8)	4,436 (26.4)
**Other valvular heart disease[#]**	**2,078 (11.6)**	**2,012 (11.9)**	**1,826 (11.2)**	**2,043 (11.8)**	**1,970 (11.7)**
Prior 4 years	811 (4.5)	846 (5.0)	829 (5.1)	975 (5.7)	949 (5.6)
Index hospitalization	1,673 (9.3)	1,660 (9.8)	1,471 (9.1)	1,557 (9.0)	1,489 (8.9)
**Peripheral artery disease**	**597 (3.3)**	**643 (3.8)**	**525 (3.2)**	**574 (3.3)**	**527 (3.1)**
Prior 4 years	309 (1.7)	339 (2.0)	328 (2.0)	340 (2.0)	312 (1.9)
Index hospitalization	358 (2.0)	420 (2.5)	297 (1.8)	325 (1.9)	287 (1.7)
**Liver diseases**	**723 (4.0)**	**662 (3.9)**	**702 (4.3)**	**681 (3.9)**	**677 (4.0)**
Prior 4 years	315 (1.8)	284 (1.7)	300 (1.8)	302 (1.8)	314 (1.9)
Index hospitalization	500 (2.8)	465 (2.8)	490 (3.0)	467 (2.7)	475 (2.8)
**Gastroesophageal reflux disease**	**87 (0.5)**	**115 (0.7)**	**130 (0.8)**	**151 (0.9)**	**131 (0.8)**
Prior 4 years	46 (0.3)	79 (0.5)	89 (0.5)	110 (0.6)	94 (0.6)
Index hospitalization	45 (0.3)	38 (0.2)	45 (0.3)	45 (0.3)	39 (0.2)
**Peptic ulcer disease**	**212 (1.2)**	**196 (1.2)**	**203 (1.2)**	**177 (1.0)**	**184 (1.1)**
Prior 4 years	112 (0.6)	89 (0.5)	87 (0.5)	91 (0.5)	80 (0.5)
Index hospitalization	106 (0.6)	109 (0.6)	118 (0.7)	94 (0.5)	109 (0.6)
**Chronic kidney diseases**	**2,798 (15.6)**	**2,884 (17.1)**	**2,782 (17.1)**	**2,908 (16.9)**	**2,791 (16.6)**
Prior 4 years	1,163 (6.5)	1,294 (7.7)	1,278 (7.9)	1,362 (7.9)	1,348 (8.0)
Index hospitalization	2,200 (12.2)	2,254 (13.3)	2,158 (13.3)	2,249 (13.0)	2,185 (13.0)
**Anaemia**	**2,530 (14.1)**	**2,695 (16.0)**	**2,628 (16.2)**	**2,673 (15.5)**	**2,730 (16.2)**
Prior 4 years	1,422 (7.9)	1,516 (9.0)	1,421 (8.7)	1,457 (8.4)	1,486 (8.8)
Index hospitalization	1,424 (7.9)	1,554 (9.2)	1,571 (9.7)	1,602 (9.3)	1,664 (9.9)
**Coagulopathy**	**467 (2.6)**	**595 (3.5)**	**622 (3.8)**	**639 (3.7)**	**708 (4.2)**
Prior 4 years	161 (0.9)	202 (1.2)	239 (1.5)	237 (1.4)	261 (1.6)
Index hospitalization	335 (1.9)	422 (2.5)	421 (2.6)	443 (2.6)	487 (2.9)
**Diabetes**	**3,671 (20.4)**	**3,385 (20.0)**	**3,276 (20.2)**	**3,509 (20.3)**	**3,346 (19.9)**
Prior 4 years	1,919 (10.7)	1,893 (11.2)	1,881 (11.6)	2,131 (12.4)	2,010 (12.0)
Index hospitalization	2,914 (16.2)	2,827 (16.7)	2,735 (16.8)	2,813 (16.3)	2,703 (16.1)

© 2023 Chen Q et al. *JAMA Network Open.*

**Thyroid disease**	**631 (3.5)**	**572 (3.4)**	**532 (3.3)**	**620 (3.6)**	**620 (3.7)**
Prior 4 years	215 (1.2)	213 (1.3)	235 (1.4)	272 (1.6)	263 (1.6)
Index hospitalization	463 (2.6)	418 (2.5)	345 (2.1)	421 (2.4)	430 (2.6)
**Ischemic stroke (history)**	**977 (5.4)**	**886 (5.2)**	**846 (5.2)**	**838 (4.9)**	**808 (4.8)**
Prior 4 years	545 (3.0)	538 (3.2)	537 (3.3)	536 (3.1)	521 (3.1)
Index hospitalization	552 (3.1)	476 (2.8)	417 (2.6)	395 (2.3)	411 (2.4)
**TIA**	**330 (1.8)**	**289 (1.7)**	**255 (1.6)**	**270 (1.6)**	**252 (1.5)**
Prior 4 years	233 (1.3)	209 (1.2)	180 (1.1)	195 (1.1)	194 (1.2)
Index hospitalization	105 (0.6)	92 (0.5)	79 (0.5)	86 (0.5)	63 (0.4)
**Other arterial thromboembolism**	**278 (1.5)**	**266 (1.6)**	**232 (1.4)**	**225 (1.3)**	**202 (1.2)**
Prior 4 years	204 (1.1)	177 (1.0)	157 (1.0)	152 (0.9)	122 (0.7)
Index hospitalization	87 (0.5)	106 (0.6)	86 (0.5)	83 (0.5)	83 (0.5)
**Parkinson's disease**	**279 (1.6)**	**244 (1.4)**	**221 (1.4)**	**250 (1.4)**	**220 (1.3)**
Prior 4 years	126 (0.7)	127 (0.8)	108 (0.7)	143 (0.8)	115 (0.7)
Index hospitalization	219 (1.2)	191 (1.1)	186 (1.1)	205 (1.2)	179 (1.1)
**Alzheimer's disease**	**350 (1.9)**	**374 (2.2)**	**343 (2.1)**	**349 (2.0)**	**345 (2.1)**
Prior 4 years	118 (0.7)	98 (0.6)	86 (0.5)	101 (0.6)	82 (0.5)
Index hospitalization	290 (1.6)	335 (2.0)	310 (1.9)	315 (1.8)	326 (1.9)
**Autoimmune disease**	**124 (0.7)**	**88 (0.5)**	**120 (0.7)**	**142 (0.8)**	**138 (0.8)**
Prior 4 years	84 (0.5)	57 (0.3)	93 (0.6)	104 (0.6)	106 (0.6)
Index hospitalization	49 (0.3)	40 (0.2)	44 (0.3)	55 (0.3)	48 (0.3)
**Systemic connective tissue disorders**	**245 (1.4)**	**303 (1.8)**	**249 (1.5)**	**283 (1.6)**	**269 (1.6)**
Prior 4 years	126 (0.7)	151 (0.9)	156 (1.0)	181 (1.0)	148 (0.9)
Index hospitalization	158 (0.9)	223 (1.3)	161 (1.0)	162 (0.9)	183 (1.1)
**Venous thromboembolism**	**427 (2.4)**	**381 (2.3)**	**446 (2.7)**	**455 (2.6)**	**375 (2.2)**
Prior 4 years to prior 6 months	113 (0.6)	108 (0.6)	153 (0.9)	128 (0.7)	102 (0.6)
Prior 6 months	321 (1.8)	282 (1.7)	304 (1.9)	335 (1.9)	282 (1.7)
**Deep vein thrombosis**	**189 (1.1)**	**163 (1.0)**	**185 (1.1)**	**184 (1.1)**	**170 (1.0)**
Prior 4 years to prior 6 months	54 (0.3)	54 (0.3)	63 (0.4)	60 (0.3)	45 (0.3)
Prior 6 months	138 (0.8)	110 (0.7)	125 (0.8)	128 (0.7)	126 (0.7)
**Pulmonary embolism**	**266 (1.5)**	**239 (1.4)**	**300 (1.8)**	**303 (1.8)**	**232 (1.4)**
Prior 4 years to prior 6 months	69 (0.4)	60 (0.4)	102 (0.6)	82 (0.5)	63 (0.4)
Prior 6 months	202 (1.1)	182 (1.1)	204 (1.3)	224 (1.3)	176 (1.0)
**Major bleeding**	**1,861 (10.4)**	**1,911 (11.3)**	**1,891 (11.6)**	**1,864 (10.8)**	**1,891 (11.2)**
Prior 4 years	889 (4.9)	881 (5.2)	924 (5.7)	916 (5.3)	889 (5.3)
Index hospitalization	1,103 (6.1)	1,168 (6.9)	1,107 (6.8)	1,092 (6.3)	1,150 (6.8)
**Intracranial haemorrhage**	**445 (2.5)**	**423 (2.5)**	**385 (2.4)**	**403 (2.3)**	**336 (2.0)**

© 2023 Chen Q et al. *JAMA Network Open.*

Prior 4 years	130 (0.7)	133 (0.8)	97 (0.6)	118 (0.7)	101 (0.6)
Index hospitalization	341 (1.9)	319 (1.9)	308 (1.9)	312 (1.8)	256 (1.5)
**Gastrointestinal bleeding**	**725 (4.0)**	**777 (4.6)**	**773 (4.8)**	**724 (4.2)**	**794 (4.7)**
Prior 4 years	431 (2.4)	415 (2.5)	433 (2.7)	426 (2.5)	421 (2.5)
Index hospitalization	332 (1.8)	398 (2.4)	376 (2.3)	343 (2.0)	415 (2.5)
**Malignant tumour**	**3,592 (20.0)**	**3,235 (19.2)**	**3,243 (20.0)**	**3,305 (19.2)**	**3,240 (19.3)**
Prior 4 years	2,588 (14.4)	2,339 (13.8)	2,329 (14.3)	2,451 (14.2)	2,393 (14.2)
Index hospitalization	2,391 (13.3)	2,282 (13.5)	2,309 (14.2)	2,284 (13.2)	2,304 (13.7)
≥1 hospitalization within the prior 4 years, n (%)	12,380 (68.9)	11,234 (66.5)	10,757 (66.2)	11,554 (67.0)	10,992 (65.4)
Information about the index hospitalization					
Length of hospital stay, days, median (IQR)	4 (1-10)	5 (1-10)	5 (1-10)	4 (0-10)	4 (0-10)
In-hospital mortality, n (%)	2,378 (13.2)	2,577 (15.3)	2,529 (15.6)	2,656 (15.4)	2,787 (16.6)
**Main reason for admission to the index hospitalization**					
Atrial fibrillation	4,732 (26.3)	4,239 (25.1)	4,190 (25.8)	5,111 (29.6)	4,785 (28.5)
Heart failure	1,056 (5.9)	985 (5.8)	975 (6.0)	977 (5.7)	980 (5.8)
Ischemic heart diseases	1,630 (9.1)	1,413 (8.4)	1,399 (8.6)	1,472 (8.5)	1,540 (9.2)
Respiratory diseases	1,493 (8.3)	1,842 (10.9)	1,709 (10.5)	1,798 (10.4)	1,820 (10.8)
Injury/poisoning	1,370 (7.6)	1,353 (8.0)	1,297 (8.0)	1,228 (7.1)	1,394 (8.3)
Symptoms/signs/abnormal laboratory findings[**]	919 (5.1)	809 (4.8)	697 (4.3)	598 (3.5)	510 (3.0)
Neoplasms	1,534 (8.5)	1,319 (7.8)	1,331 (8.2)	1,380 (8.0)	1,351 (8.0)
Digestive diseases	1,044 (5.8)	927 (5.5)	931 (5.7)	899 (5.2)	852 (5.1)
Other heart valve disorders[§§]	303 (1.7)	332 (2.0)	335 (2.1)	349 (2.0)	292 (1.7)
Genitourinary diseases	559 (3.1)	614 (3.6)	579 (3.6)	586 (3.4)	545 (3.2)
Infectious/parasitic diseases	536 (3.0)	503 (3.0)	458 (2.8)	490 (2.8)	477 (2.8)
Endocrine/nutritional/metabolic diseases	278 (1.5)	252 (1.5)	227 (1.4)	206 (1.2)	238 (1.4)
Diseases of blood/blood-forming organs	211 (1.2)	231 (1.4)	179 (1.1)	186 (1.1)	173 (1.0)
Neurological disorders[##]	205 (1.1)	187 (1.1)	168 (1.0)	160 (0.9)	160 (1.0)

† First-generation immigrants refer to persons who were born abroad with at least one parent who was born abroad; second-generation immigrants refer to persons who was born in the Netherlands with at least one parent who was born abroad.

‡ Percentile groups were determined based on disposable income of private households of the complete target population in the database (instead of the study population only).

* Labile INR (international normalized ratio) was not included for calculation.

§ Pre-existing chronic use was defined as ≥2 (outpatient) prescription records of the same type of antithrombotic agents within six months (*i.e.*, 183 days) before the index dates (exclusive). Pre-existing chronic use of oral anticoagulant refers to persons with pre-existing chronic use of vitamin K antagonist or direct oral anticoagulant.

|| Unless otherwise stated, for each comorbidity (or medical history), prevalence calculated by screening all hospitalizations within the prior four years (including the index hospitalization), all hospitalizations within the prior four years (excluding the index hospitalization), and only the index hospitalization is presented, respectively.

Except for rheumatic mitral stenosis and mechanical heart valves

** Except for abnormalities of heart beat (ICD-10 code: R00) and cardiac murmurs and other cardiac sounds (ICD-10 code: R01)

§§ Except for endocarditis, valve unspecified, in diseases classified elsewhere (ICD-10 code: I398)

Except for transient cerebral ischaemic attacks and related syndromes (ICD-10 code: G45) and vascular syndromes of brain in cerebrovascular diseases (ICD-10 code: G46) Abbreviations: SD, standard deviation; TIA, transient ischemic attack; IQR, interquartile range; ICD, International Classification of Diseases.

© 2023 Chen Q et al. *JAMA Network Open.*

eTable 8. Time Trends in Patient Characteristics of Incident Nonvalvular Atrial Fibrillation Among Patients Who Received at Least 1 Oral Anticoagulant Within the 1-Year Follow-up

Cohort	2014 (N=37,905)	2015 (N=44,426)	2016 (N=43,767)	2017 (N=45,144)	2018 (N=44,881)
Age, years, mean ± SD	74.8 ± 10.9	74.5 ± 10.9	74.4 ± 10.9	74.6 ± 10.8	74.5 ± 10.9
Age group, years, n (%)					
18-24	15 (0.0)	17 (0.0)	18 (0.0)	24 (0.1)	26 (0.1)
25-34	63 (0.2)	82 (0.2)	85 (0.2)	71 (0.2)	106 (0.2)
35-44	278 (0.7)	387 (0.9)	328 (0.7)	336 (0.7)	328 (0.7)
45-54	1,471 (3.9)	1,770 (4.0)	1,730 (4.0)	1,757 (3.9)	1,735 (3.9)
55-64	4,684 (12.4)	5,579 (12.6)	5,664 (12.9)	5,765 (12.8)	5,702 (12.7)
65-74	11,302 (29.8)	13,673 (30.8)	13,669 (31.2)	14,079 (31.2)	14,178 (31.6)
75-84	13,272 (35.0)	15,319 (34.5)	14,915 (34.1)	15,283 (33.9)	15,180 (33.8)
≥85	6,820 (18.0)	7,599 (17.1)	7,358 (16.8)	7,829 (17.3)	7,626 (17.0)
Sex, n (%)					
Male	21,257 (56.1)	24,471 (55.1)	24,409 (55.8)	25,413 (56.3)	25,594 (57.0)
Female	16,648 (43.9)	19,955 (44.9)	19,358 (44.2)	19,731 (43.7)	19,287 (43.0)
Immigration background[†], n (%)					
Native Dutch	33,443 (88.2)	39,179 (88.2)	38,382 (87.7)	39,659 (87.8)	39,417 (87.8)
First-generation immigrants	2,505 (6.6)	2,863 (6.4)	2,892 (6.6)	2,969 (6.6)	3,046 (6.8)
Second-generation immigrants	1,957 (5.2)	2,384 (5.4)	2,493 (5.7)	2,516 (5.6)	2,418 (5.4)
Standardized household income[‡], n (%)					
First quintile (0-20%)	5,963 (15.8)	6,768 (15.3)	7,029 (16.1)	7,365 (16.3)	7,215 (16.1)
Second quintile (20-40%)	11,083 (29.3)	13,204 (29.8)	12,871 (29.4)	13,349 (29.6)	13,267 (29.6)
Third quintile (40-60%)	9,195 (24.3)	10,761 (24.3)	10,556 (24.2)	11,071 (24.6)	11,266 (25.1)
Fourth quintile (60-80%)	5,818 (15.4)	7,011 (15.8)	6,991 (16.0)	7,004 (15.5)	6,950 (15.5)
Fifth quintile (80-100%)	4,480 (11.8)	5,373 (12.1)	5,236 (12.0)	5,357 (11.9)	5,232 (11.7)
Private household with an unknown income	17 (0.0)	29 (0.1)	23 (0.1)	23 (0.1)	16 (0.0)
Institutional household	1,299 (3.4)	1,212 (2.7)	1,001 (2.3)	889 (2.0)	852 (1.9)
Marital status, n (%)					
Married or in partnership	21,481 (56.7)	25,674 (57.8)	25,272 (57.7)	25,856 (57.3)	25,779 (57.4)
Unmarried or single	2,394 (6.3)	2,840 (6.4)	2,902 (6.6)	3,200 (7.1)	3,231 (7.2)
Divorced	3,660 (9.7)	4,324 (9.7)	4,414 (10.1)	4,701 (10.4)	4,812 (10.7)
Widowed	10,370 (27.4)	11,588 (26.1)	11,179 (25.5)	11,387 (25.2)	11,059 (24.6)
Type of atrial fibrillation, n (%)					
Paroxysmal atrial fibrillation (I48.0)	-	7,105 (16.0)	7,038 (16.1)	7,217 (16.0)	6,846 (15.3)
Persistent atrial fibrillation (I48.1)	-	1,541 (3.5)	1,644 (3.8)	1,670 (3.7)	1,668 (3.7)

© 2023 Chen Q et al. *JAMA Network Open.*

Chronic atrial fibrillation (I48.2)	-	2,533 (5.7)	2,572 (5.9)	2,631 (5.8)	2,604 (5.8)
Type I atrial flutter (I48.3)	-	291 (0.7)	349 (0.8)	434 (1.0)	526 (1.2)
Type II atrial flutter (I48.4)	-	139 (0.3)	111 (0.3)	105 (0.2)	104 (0.2)
Unspecified atrial fibrillation (I48.9)	-	32,817 (73.9)	32,053 (73.2)	33,087 (73.3)	33,133 (73.8)
Diagnosis made before 2015	37,905 (100.0)	-	-	-	-
CHA_2DS_2-VASc score, mean ± SD	2.98 ± 1.63	2.93 ± 1.63	2.92 ± 1.62	2.94 ± 1.62	2.94 ± 1.61
CHA_2DS_2-VASc score, n (%)					
0	2,311 (6.1)	2,880 (6.5)	2,880 (6.6)	2,795 (6.2)	2,780 (6.2)
1	4,839 (12.8)	5,861 (13.2)	5,745 (13.1)	5,956 (13.2)	5,989 (13.3)
**≥2**	**30,755 (81.1)**	**35,685 (80.3)**	**35,142 (80.3)**	**36,393 (80.6)**	**36,112 (80.5)**
2	7,810 (20.6)	9,414 (21.2)	9,355 (21.4)	9,454 (20.9)	9,329 (20.8)
3	9,093 (24.0)	10,562 (23.8)	10,506 (24.0)	10,936 (24.2)	10,856 (24.2)
4	7,333 (19.3)	8,325 (18.7)	8,158 (18.6)	8,595 (19.0)	8,618 (19.2)
5	4,060 (10.7)	4,688 (10.6)	4,500 (10.3)	4,646 (10.3)	4,646 (10.4)
6	1,716 (4.5)	1,871 (4.2)	1,850 (4.2)	1,937 (4.3)	1,922 (4.3)
≥7	743 (2.0)	825 (1.9)	773 (1.8)	825 (1.8)	741 (1.7)
HAS-BLED score*, mean ± SD	1.79 ± 0.99	1.74 ± 0.98	1.73 ± 0.99	1.72 ± 0.98	1.70 ± 0.98
HAS-BLED score*, n (%)					
0	2,925 (7.7)	3,788 (8.5)	3,832 (8.8)	3,992 (8.8)	4,059 (9.0)
1	12,483 (32.9)	15,193 (34.2)	15,288 (34.9)	15,944 (35.3)	16,073 (35.8)
2	13912 (36.7)	16,262 (36.6)	15,709 (35.9)	16,049 (35.6)	15,860 (35.3)
≥3	8,585 (22.6)	9,183 (20.7)	8,938 (20.4)	9,159 (20.3)	8,889 (19.8)
Pre-existing chronic use of antithrombotic agents§					
**Oral anticoagulant, n (%)**	**13,490 (35.6)**	**15,304 (34.4)**	**14,976 (34.2)**	**16,284 (36.1)**	**17,323 (38.6)**
Vitamin K antagonist, n (%)	11,698 (30.9)	11,416 (25.7)	9,319 (21.3)	7,929 (17.6)	6,487 (14.5)
Direct oral anticoagulant, n (%)	1,849 (4.9)	4,020 (9.0)	5,814 (13.3)	8,503 (18.8)	10,950 (24.4)
Heparin group, n (%)	651 (1.7)	656 (1.5)	554 (1.3)	454 (1.0)	377 (0.8)
Antiplatelet agent, n (%)	9,723 (25.7)	11,022 (24.8)	10,721 (24.5)	10,897 (24.1)	10,675 (23.8)
Comorbidities (or medical history)‖, n (%)					
**Asthma**	**720 (1.9)**	**929 (2.1)**	**954 (2.2)**	**1,013 (2.2)**	**1,191 (2.7)**
Prior 4 years	329 (0.9)	451 (1.0)	506 (1.2)	506 (1.1)	622 (1.4)
Index hospitalization	489 (1.3)	601 (1.4)	593 (1.4)	672 (1.5)	809 (1.8)
**Chronic obstructive pulmonary disease**	**4,606 (12.2)**	**5,095 (11.5)**	**4,947 (11.3)**	**4,987 (11.0)**	**4,899 (10.9)**
Prior 4 years	2,154 (5.7)	2,501 (5.6)	2,598 (5.9)	2,623 (5.8)	2,596 (5.8)
Index hospitalization	3,786 (10.0)	4,144 (9.3)	3,979 (9.1)	3,998 (8.9)	4,014 (8.9)
**Other chronic lung diseases**	**708 (1.9)**	**703 (1.6)**	**732 (1.7)**	**795 (1.8)**	**896 (2.0)**
Prior 4 years	414 (1.1)	375 (0.8)	404 (0.9)	397 (0.9)	426 (0.9)

© 2023 Chen Q et al. *JAMA Network Open.*

Index hospitalization	336 (0.9)	388 (0.9)	392 (0.9)	497 (1.1)	578 (1.3)
**Heart failure**	**8,285 (21.9)**	**9,163 (20.6)**	**8,931 (20.4)**	**9,408 (20.8)**	**9,507 (21.2)**
Prior 4 years	2,165 (5.7)	2,285 (5.1)	2,311 (5.3)	2,353 (5.2)	2,302 (5.1)
Index hospitalization	7,075 (18.7)	7,887 (17.8)	7,707 (17.6)	8,129 (18.0)	8,302 (18.5)
**Myocardial infarction (history)**	**4,232 (11.2)**	**4,933 (11.1)**	**5,095 (11.6)**	**5,337 (11.8)**	**5,155 (11.5)**
Prior 4 years	2,906 (7.7)	3321 (7.5)	3,458 (7.9)	3,638 (8.1)	3,419 (7.6)
Index hospitalization	1,581 (4.2)	1971 (4.4)	2,022 (4.6)	2,184 (4.8)	2,211 (4.9)
**Hypertension**	**13,842 (36.5)**	**16,308 (36.7)**	**16,268 (37.2)**	**17,386 (38.5)**	**17,778 (39.6)**
Prior 4 years	5,018 (13.2)	6,368 (14.3)	6,641 (15.2)	7,292 (16.2)	7,401 (16.5)
Index hospitalization	11,499 (30.3)	13,393 (30.1)	13,273 (30.3)	1,4132 (31.3)	14,677 (32.7)
**Other valvular heart disease[#]**	**5,928 (15.6)**	**6,415 (14.4)**	**6,176 (14.1)**	**6,415 (14.2)**	**6,306 (14.1)**
Prior 4 years	2,204 (5.8)	2,482 (5.6)	2,560 (5.8)	2,679 (5.9)	2,656 (5.9)
Index hospitalization	4,966 (13.1)	5,397 (12.1)	5,182 (11.8)	5,318 (11.8)	5,232 (11.7)
**Peripheral artery disease**	**1,020 (2.7)**	**1,235 (2.8)**	**1,197 (2.7)**	**1,242 (2.8)**	**1,234 (2.7)**
Prior 4 years	501 (1.3)	642 (1.4)	709 (1.6)	748 (1.7)	726 (1.6)
Index hospitalization	667 (1.8)	792 (1.8)	697 (1.6)	698 (1.5)	699 (1.6)
**Liver diseases**	**822 (2.2)**	**951 (2.1)**	**923 (2.1)**	**964 (2.1)**	**950 (2.1)**
Prior 4 years	329 (0.9)	417 (0.9)	458 (1.0)	471 (1.0)	494 (1.1)
Index hospitalization	552 (1.5)	593 (1.3)	547 (1.2)	584 (1.3)	561 (1.2)
**Gastroesophageal reflux disease**	**175 (0.5)**	**190 (0.4)**	**230 (0.5)**	**275 (0.6)**	**263 (0.6)**
Prior 4 years	105 (0.3)	140 (0.3)	167 (0.4)	199 (0.4)	195 (0.4)
Index hospitalization	72 (0.2)	54 (0.1)	69 (0.2)	82 (0.2)	72 (0.2)
**Peptic ulcer disease**	**261 (0.7)**	**260 (0.6)**	**245 (0.6)**	**275 (0.6)**	**260 (0.6)**
Prior 4 years	133 (0.4)	133 (0.3)	154 (0.4)	149 (0.3)	141 (0.3)
Index hospitalization	133 (0.4)	135 (0.3)	94 (0.2)	132 (0.3)	126 (0.3)
**Chronic kidney diseases**	**4,600 (12.1)**	**5,175 (11.6)**	**5,640 (12.9)**	**5,978 (13.2)**	**5,630 (12.5)**
Prior 4 years	1,851 (4.9)	2,162 (4.9)	2,439 (5.6)	2,603 (5.8)	2,556 (5.7)
Index hospitalization	3,608 (9.5)	4,050 (9.1)	4,358 (10.0)	4,647 (10.3)	4,400 (9.8)
**Anaemia**	**3,661 (9.7)**	**4,069 (9.2)**	**4,287 (9.8)**	**4,730 (10.5)**	**4,847 (10.8)**
Prior 4 years	1,933 (5.1)	2,172 (4.9)	2,288 (5.2)	2,492 (5.5)	2,527 (5.6)
Index hospitalization	2,103 (5.5)	2,314 (5.2)	2,444 (5.6)	2,788 (6.2)	2,882 (6.4)
**Coagulopathy**	**580 (1.5)**	**891 (2.0)**	**858 (2.0)**	**963 (2.1)**	**1,036 (2.3)**
Prior 4 years	184 (0.5)	309 (0.7)	335 (0.8)	393 (0.9)	392 (0.9)
Index hospitalization	414 (1.1)	614 (1.4)	561 (1.3)	601 (1.3)	692 (1.5)
**Diabetes**	**7,441 (19.6)**	**8,481 (19.1)**	**8,434 (19.3)**	**8,732 (19.3)**	**8,646 (19.3)**
Prior 4 years	3,429 (9.0)	4,171 (9.4)	4,293 (9.8)	4,534 (10.0)	4,382 (9.8)
Index hospitalization	6,328 (16.7)	7,197 (16.2)	7,209 (16.5)	7,495 (16.6)	7,638 (17.0)

© 2023 Chen Q et al. *JAMA Network Open.*

**Thyroid disease**	**1,230 (3.2)**	**1,406 (3.2)**	**1,431 (3.3)**	**1,536 (3.4)**	**1,463 (3.3)**
Prior 4 years	375 (1.0)	435 (1.0)	495 (1.1)	533 (1.2)	494 (1.1)
Index hospitalization	945 (2.5)	1,086 (2.4)	1,064 (2.4)	1,159 (2.6)	1,140 (2.5)
**Ischemic stroke (history)**	**1,572 (4.1)**	**1,709 (3.8)**	**1,569 (3.6)**	**1,658 (3.7)**	**1,603 (3.6)**
Prior 4 years	1,068 (2.8)	1,221 (2.7)	1,150 (2.6)	1,256 (2.8)	1,164 (2.6)
Index hospitalization	703 (1.9)	667 (1.5)	555 (1.3)	595 (1.3)	636 (1.4)
**TIA**	**1,081 (2.9)**	**1,174 (2.6)**	**1,214 (2.8)**	**1,183 (2.6)**	**1,108 (2.5)**
Prior 4 years	560 (1.5)	623 (1.4)	624 (1.4)	665 (1.5)	584 (1.3)
Index hospitalization	545 (1.4)	579 (1.3)	637 (1.5)	550 (1.2)	564 (1.3)
**Other arterial thromboembolism**	**510 (1.3)**	**502 (1.1)**	**484 (1.1)**	**489 (1.1)**	**405 (0.9)**
Prior 4 years	389 (1.0)	364 (0.8)	352 (0.8)	322 (0.7)	276 (0.6)
Index hospitalization	145 (0.4)	169 (0.4)	151 (0.3)	185 (0.4)	139 (0.3)
**Parkinson's disease**	**278 (0.7)**	**412 (0.9)**	**384 (0.9)**	**435 (1.0)**	**404 (0.9)**
Prior 4 years	137 (0.4)	196 (0.4)	173 (0.4)	207 (0.5)	170 (0.4)
Index hospitalization	210 (0.6)	323 (0.7)	310 (0.7)	352 (0.8)	349 (0.8)
**Alzheimer's disease**	**260 (0.7)**	**290 (0.7)**	**297 (0.7)**	**300 (0.7)**	**319 (0.7)**
Prior 4 years	82 (0.2)	103 (0.2)	100 (0.2)	82 (0.2)	68 (0.2)
Index hospitalization	216 (0.6)	238 (0.5)	249 (0.6)	260 (0.6)	290 (0.6)
**Autoimmune disease**	**117 (0.3)**	**139 (0.3)**	**183 (0.4)**	**203 (0.4)**	**204 (0.5)**
Prior 4 years	74 (0.2)	92 (0.2)	136 (0.3)	137 (0.3)	142 (0.3)
Index hospitalization	57 (0.2)	65 (0.1)	73 (0.2)	99 (0.2)	91 (0.2)
**Systemic connective tissue disorders**	**441 (1.2)**	**540 (1.2)**	**583 (1.3)**	**585 (1.3)**	**655 (1.5)**
Prior 4 years	186 (0.5)	243 (0.5)	325 (0.7)	334 (0.7)	321 (0.7)
Index hospitalization	308 (0.8)	378 (0.9)	377 (0.9)	366 (0.8)	457 (1.0)
**Venous thromboembolism**	**759 (2.0)**	**832 (1.9)**	**822 (1.9)**	**931 (2.1)**	**918 (2.0)**
Prior 4 years to prior 6 months	263 (0.7)	309 (0.7)	301 (0.7)	299 (0.7)	303 (0.7)
Prior 6 months	518 (1.4)	551 (1.2)	550 (1.3)	663 (1.5)	636 (1.4)
**Deep vein thrombosis**	**307 (0.8)**	**341 (0.8)**	**306 (0.7)**	**364 (0.8)**	**325 (0.7)**
Prior 4 years to prior 6 months	111 (0.3)	129 (0.3)	106 (0.2)	118 (0.3)	104 (0.2)
Prior 6 months	200 (0.5)	220 (0.5)	205 (0.5)	252 (0.6)	224 (0.5)
**Pulmonary embolism**	**545 (1.4)**	**581 (1.3)**	**612 (1.4)**	**655 (1.5)**	**671 (1.5)**
Prior 4 years to prior 6 months	183 (0.5)	216 (0.5)	228 (0.5)	221 (0.5)	232 (0.5)
Prior 6 months	377 (1.0)	381 (0.9)	405 (0.9)	452 (1.0)	456 (1.0)
**Major bleeding**	**2,398 (6.3)**	**2,703 (6.1)**	**2,526 (5.8)**	**2,913 (6.5)**	**2,865 (6.4)**
Prior 4 years	1,398 (3.7)	1,573 (3.5)	1,520 (3.5)	1,701 (3.8)	1,656 (3.7)
Index hospitalization	1,132 (3.0)	1,282 (2.9)	1,141 (2.6)	1,354 (3.0)	1,360 (3.0)
**Intracranial haemorrhage**	**267 (0.7)**	**272 (0.6)**	**217 (0.5)**	**270 (0.6)**	**270 (0.6)**

© 2023 Chen Q et al. *JAMA Network Open.*

Prior 4 years	133 (0.4)	129 (0.3)	106 (0.2)	148 (0.3)	123 (0.3)
Index hospitalization	146 (0.4)	162 (0.4)	119 (0.3)	138 (0.3)	164 (0.4)
**Gastrointestinal bleeding**	**1,085 (2.9)**	**1,121 (2.5)**	**1,100 (2.5)**	**1,282 (2.8)**	**1,214 (2.7)**
Prior 4 years	664 (1.8)	716 (1.6)	726 (1.7)	796 (1.8)	773 (1.7)
Index hospitalization	464 (1.2)	452 (1.0)	426 (1.0)	539 (1.2)	498 (1.1)
**Malignant tumour**	**4,250 (11.2)**	**4,856 (10.9)**	**4,848 (11.1)**	**5,152 (11.4)**	**5,131 (11.4)**
Prior 4 years	3,360 (8.9)	3,826 (8.6)	3,845 (8.8)	4,109 (9.1)	4,030 (9.0)
Index hospitalization	1,973 (5.2)	2,382 (5.4)	2,373 (5.4)	2,546 (5.6)	2,675 (6.0)
≥1 hospitalization within the prior 4 years, n (%)	24,624 (65.0)	28,009 (63.0)	27,432 (62.7)	28,028 (62.1)	27,146 (60.5)
Information about the index hospitalization					
Length of hospital stay, days, median (IQR)	3 (1-7)	2 (0-7)	2 (0-7)	2 (0-7)	2 (0-7)
In-hospital mortality, n (%)	34 (0.1)	45 (0.1)	28 (0.1)	53 (0.1)	78 (0.2)
**Main reason for admission to the index hospitalization**					
Atrial fibrillation	14,764 (39.0)	19,018 (42.8)	18,857 (43.1)	18,671 (41.4)	18,092 (40.3)
Heart failure	3,510 (9.3)	3,921 (8.8)	3,792 (8.7)	3,895 (8.6)	3,925 (8.7)
Ischemic heart diseases	2,874 (7.6)	3,131 (7.0)	3,070 (7.0)	3,242 (7.2)	3,291 (7.3)
Respiratory diseases	2,387 (6.3)	3,021 (6.8)	2,932 (6.7)	3,240 (7.2)	3,424 (7.6)
Injury/poisoning	1,602 (4.2)	1,767 (4.0)	1,841 (4.2)	1,981 (4.4)	2,070 (4.6)
Symptoms/signs/abnormal laboratory findings**	2,003 (5.3)	2,188 (4.9)	2,064 (4.7)	2,057 (4.6)	1,973 (4.4)
Neoplasms	1,215 (3.2)	1,475 (3.3)	1,391 (3.2)	1,489 (3.3)	1,578 (3.5)
Digestive diseases	1,433 (3.8)	1,543 (3.5)	1,366 (3.1)	1,616 (3.6)	1,544 (3.4)
Other heart valve disorders§§	1,174 (3.1)	1,166 (2.6)	1,295 (3.0)	1,274 (2.8)	1,242 (2.8)
Genitourinary diseases	910 (2.4)	917 (2.1)	991 (2.3)	1,086 (2.4)	1,162 (2.6)
Infectious/parasitic diseases	523 (1.4)	566 (1.3)	579 (1.3)	714 (1.6)	712 (1.6)
Endocrine/nutritional/metabolic diseases	402 (1.1)	418 (0.9)	389 (0.9)	428 (0.9)	483 (1.1)
Diseases of blood/blood-forming organs	281 (0.7)	272 (0.6)	286 (0.7)	328 (0.7)	310 (0.7)
Neurological disorders##	286 (0.8)	268 (0.6)	234 (0.5)	281 (0.6)	245 (0.5)

† First-generation immigrants refer to persons who were born abroad with at least one parent who was born abroad; second-generation immigrants refer to persons who was born in the Netherlands with at least one parent who was born abroad.

‡ Percentile groups were determined based on disposable income of private households of the complete target population in the database (instead of the study population only).

* Labile INR (international normalized ratio) was not included for calculation.

§ Pre-existing chronic use was defined as ≥2 (outpatient) prescription records of the same type of antithrombotic agents within six months (*i.e.*, 183 days) before the index dates (exclusive). Pre-existing chronic use of oral anticoagulant refers to persons with pre-existing chronic use of vitamin K antagonist or direct oral anticoagulant.

|| Unless otherwise stated, for each comorbidity (or medical history), prevalence calculated by screening all hospitalizations within the prior four years (including the index hospitalization), all hospitalizations within the prior four years (excluding the index hospitalization), and only the index hospitalization is presented, respectively.

Except for rheumatic mitral stenosis and mechanical heart valves

** Except for abnormalities of heart beat (ICD-10 code: R00) and cardiac murmurs and other cardiac sounds (ICD-10 code: R01)

§§ Except for endocarditis, valve unspecified, in diseases classified elsewhere (ICD-10 code: I398)

Except for transient cerebral ischaemic attacks and related syndromes (ICD-10 code: G45) and vascular syndromes of brain in cerebrovascular diseases (ICD-10 code: G46) Abbreviations: SD, standard deviation; TIA, transient ischemic attack; IQR, interquartile range; ICD, International Classification of Diseases.

© 2023 Chen Q et al. *JAMA Network Open.*

eTable 9. Time Trends in Patient Characteristics of Incident Nonvalvular Atrial Fibrillation Among Patients Who First Received Vitamin K Antagonist as Oral Anticoagulant Within the 1-Year Follow-up

Cohort	2014 (N=32,803)	2015 (N=31,387)	2016 (N=23,152)	2017 (N=16,979)	2018 (N=12,567)
Age, years, mean ± SD	75.6 ± 10.7	76.2 ± 10.4	76.7 ± 10.2	77.4 ± 10.2	77.5 ± 10.2
Age group, years, n (%)					
18-24	12 (0.0)	10 (0.0)	[masked][‖]	[masked][‖]	[masked][‖]
25-34	46 (0.1)	41 (0.1)	[masked][‖]	[masked][‖]	[masked][‖]
35-44	203 (0.6)	187 (0.6)	109 (0.5)	70 (0.4)	47 (0.4)
45-54	1,091 (3.3)	873 (2.8)	527 (2.3)	369 (2.2)	287 (2.3)
55-64	3,651 (11.1)	3,061 (9.8)	2,134 (9.2)	1,393 (8.2)	1,044 (8.3)
65-74	9,378 (28.6)	8,846 (28.2)	6,409 (27.7)	4,460 (26.3)	3,228 (25.7)
75-84	11,997 (36.6)	11,862 (37.8)	8,861 (38.3)	6,577 (38.7)	4,823 (38.4)
≥85	6,425 (19.6)	6,507 (20.7)	5,072 (21.9)	4,080 (24.0)	3,117 (24.8)
Sex, n (%)					
Male	18,144 (55.3)	16,982 (54.1)	12,666 (54.7)	9,515 (56.0)	7,193 (57.2)
Female	14,659 (44.7)	14,405 (45.9)	10,486 (45.3)	7,464 (44.0)	5,374 (42.8)
Immigration background[†], n (%)					
Native Dutch	28,944 (88.2)	27,746 (88.4)	20,353 (87.9)	14,963 (88.1)	11,074 (88.1)
First-generation immigrants	2,189 (6.7)	2,020 (6.4)	1,527 (6.6)	1,105 (6.5)	833 (6.6)
Second-generation immigrants	1,670 (5.1)	1,621 (5.2)	1,272 (5.5)	911 (5.4)	660 (5.3)
Standardized household income[‡], n (%)					
First quintile (0-20%)	5,368 (16.4)	5,201 (16.6)	4,178 (18.1)	3,217 (19.0)	2,280 (18.2)
Second quintile (20-40%)	9,907 (30.2)	9,971 (31.8)	7,456 (32.3)	5,585 (32.9)	4,158 (33.1)
Third quintile (40-60%)	7,934 (24.2)	7,552 (24.1)	5,484 (23.7)	4,027 (23.7)	3,128 (24.9)
Fourth quintile (60-80%)	4,828 (14.7)	4,495 (14.3)	3,191 (13.8)	2,204 (13.0)	1,624 (12.9)
Fifth quintile (80-100%)	3,468 (10.6)	3,051 (9.7)	2,072 (9.0)	1,413 (8.3)	1,005 (8.0)
Private household with an unknown income	14 (0.0)	19 (0.1)	[masked][‖]	10 (0.1)	[masked][‖]
Institutional household	1,241 (3.8)	1,056 (3.4)	[masked][‖]	504 (3.0)	[masked][‖]
Marital status, n (%)					
Married or in partnership	18,182 (55.4)	17,337 (55.2)	12,623 (54.5)	9,118 (53.7)	6,795 (54.1)
Unmarried or single	2,025 (6.2)	1,882 (6.0)	1,392 (6.0)	1,076 (6.3)	784 (6.2)
Divorced	3,106 (9.5)	2,910 (9.3)	2,212 (9.6)	1,605 (9.5)	1,202 (9.6)
Widowed	9,490 (28.9)	9,258 (29.5)	6,925 (29.9)	5,180 (30.5)	3,786 (30.1)
Type of atrial fibrillation, n (%)					
Paroxysmal atrial fibrillation (I48.0)	-	4,823 (15.4)	3,519 (15.2)	2,470 (14.5)	1,775 (14.1)
Persistent atrial fibrillation (I48.1)	-	994 (3.2)	827 (3.6)	591 (3.5)	419 (3.3)

© 2023 Chen Q et al. *JAMA Network Open.*

Chronic atrial fibrillation (I48.2)	-	2,221 (7.1)	2,058 (8.9)	1,848 (10.9)	1,634 (13.0)
Type I atrial flutter (I48.3)	-	180 (0.6)	147 (0.6)	103 (0.6)	85 (0.7)
Type II atrial flutter (I48.4)	-	85 (0.3)	46 (0.2)	34 (0.2)	26 (0.2)
Unspecified atrial fibrillation (I48.9)	-	23,084 (73.5)	16,555 (71.5)	11,933 (70.3)	8,628 (68.7)
Diagnosis made before 2015	32,803 (100.0)	-	-	-	-
CHA_2DS_2-VASc score, mean ± SD	3.10 ± 1.61	3.19 ± 1.59	3.26 ± 1.57	3.34 ± 1.54	3.36 ± 1.52
CHA_2DS_2-VASc score, n (%)					
0	1,639 (5.0)	1,304 (4.2)	840 (3.6)	479 (2.8)	328 (2.6)
1	3,718 (11.3)	3,171 (10.1)	2,130 (9.2)	1,450 (8.5)	1,011 (8.0)
**≥2**	**27,446 (83.7)**	**26,912 (85.7)**	**20,182 (87.2)**	**15,050 (88.6)**	**11,228 (89.3)**
2	6,500 (19.8)	6,165 (19.6)	4,372 (18.9)	3,019 (17.8)	2,231 (17.8)
3	8,125 (24.8)	7,858 (25.0)	5,917 (25.6)	4,467 (26.3)	3,318 (26.4)
4	6,761 (20.6)	6,668 (21.2)	5,143 (22.2)	3,907 (23.0)	2,969 (23.6)
5	3,769 (11.5)	3,923 (12.5)	2,954 (12.8)	2,291 (13.5)	1,703 (13.6)
6	1,590 (4.8)	1,573 (5.0)	1,265 (5.5)	970 (5.7)	720 (5.7)
≥7	701 (2.1)	725 (2.3)	531 (2.3)	396 (2.3)	287 (2.3)
HAS-BLED score[*], mean ± SD	1.84 ± 0.99	1.84 ± 0.97	1.86 ± 0.98	1.87 ± 0.98	1.84 ± 0.97
HAS-BLED score[*], n (%)					
0	2,191 (6.7)	1,849 (5.9)	1,237 (5.3)	817 (4.8)	615 (4.9)
1	10,582 (32.3)	10,234 (32.6)	7,692 (33.2)	5,700 (33.6)	4,329 (34.4)
2	12,225 (37.3)	11,998 (38.2)	8,695 (37.6)	6,337 (37.3)	4,765 (37.9)
≥3	7,805 (23.8)	7,306 (23.3)	5,528 (23.9)	4,125 (24.3)	2,858 (22.7)
Pre-existing chronic use of antithrombotic agents[§]					
**Oral anticoagulant, n (%)**	**11,576 (35.3)**	**11,178 (35.6)**	**8,949 (38.7)**	**7,461 (43.9)**	**6,022 (47.9)**
Vitamin K antagonist, n (%)	11,460 (34.9)	10,998 (35.0)	8,769 (37.9)	7,234 (42.6)	5,788 (46.1)
Direct oral anticoagulant, n (%)	133 (0.4)	221 (0.7)	212 (0.9)	254 (1.5)	258 (2.1)
Heparin group, n (%)	627 (1.9)	587 (1.9)	440 (1.9)	288 (1.7)	197 (1.6)
Antiplatelet agent, n (%)	8,374 (25.5)	7,703 (24.5)	5,073 (21.9)	3,053 (18.0)	1,968 (15.7)
Comorbidities (or medical history)[‖], n (%)					
**Asthma**	**640 (2.0)**	**702 (2.2)**	**523 (2.3)**	**407 (2.4)**	**348 (2.8)**
Prior 4 years	297 (0.9)	345 (1.1)	289 (1.2)	213 (1.3)	179 (1.4)
Index hospitalization	430 (1.3)	462 (1.5)	308 (1.3)	263 (1.5)	235 (1.9)
**Chronic obstructive pulmonary disease**	**4,215 (12.8)**	**4,106 (13.1)**	**2,986 (12.9)**	**2,160 (12.7)**	**1,575 (12.5)**
Prior 4 years	1,979 (6.0)	1,992 (6.3)	1,587 (6.9)	1,142 (6.7)	851 (6.8)
Index hospitalization	3,472 (10.6)	3,357 (10.7)	2,378 (10.3)	1,734 (10.2)	1,263 (10.1)
**Other chronic lung diseases**	**646 (2.0)**	**570 (1.8)**	**450 (1.9)**	**325 (1.9)**	**265 (2.1)**
Prior 4 years	377 (1.1)	304 (1.0)	248 (1.1)	160 (0.9)	124 (1.0)

© 2023 Chen Q et al. *JAMA Network Open.*

Index hospitalization	309 (0.9)	310 (1.0)	239 (1.0)	208 (1.2)	163 (1.3)
**Heart failure**	**7,715 (23.5)**	**7,558 (24.1)**	**5,899 (25.5)**	**4,559 (26.9)**	**3,494 (27.8)**
Prior 4 years	2,061 (6.3)	2,010 (6.4)	1,725 (7.5)	1,383 (8.1)	1,123 (8.9)
Index hospitalization	6,571 (20.0)	6,450 (20.5)	4,997 (21.6)	3,849 (22.7)	2,924 (23.3)
**Myocardial infarction (history)**	**3,881 (11.8)**	**4,048 (12.9)**	**3,250 (14.0)**	**2,400 (14.1)**	**1,641 (13.1)**
Prior 4 years	2,649 (8.1)	2,684 (8.6)	2,177 (9.4)	1,696 (10.0)	1,184 (9.4)
Index hospitalization	1,476 (4.5)	1,683 (5.4)	1,326 (5.7)	924 (5.4)	590 (4.7)
**Hypertension**	**12,282 (37.4)**	**12,331 (39.3)**	**9,316 (40.2)**	**7,228 (42.6)**	**5,591 (44.5)**
Prior 4 years	4,547 (13.9)	5,025 (16.0)	3,998 (17.3)	3,176 (18.7)	2,374 (18.9)
Index hospitalization	10,182 (31.0)	10,110 (32.2)	7,521 (32.5)	5,843 (34.4)	4,605 (36.6)
**Other valvular heart disease**[#]	**5,503 (16.8)**	**5,396 (17.2)**	**4,348 (18.8)**	**3,570 (21.0)**	**2,835 (22.6)**
Prior 4 years	2,089 (6.4)	2,191 (7.0)	1,906 (8.2)	1,623 (9.6)	1,383 (11.0)
Index hospitalization	4,621 (14.1)	4,556 (14.5)	3,677 (15.9)	2,990 (17.6)	2,390 (19.0)
**Peripheral artery disease**	**940 (2.9)**	**1,013 (3.2)**	**804 (3.5)**	**596 (3.5)**	**470 (3.7)**
Prior 4 years	461 (1.4)	526 (1.7)	474 (2.0)	364 (2.1)	287 (2.3)
Index hospitalization	621 (1.9)	651 (2.1)	473 (2.0)	327 (1.9)	258 (2.1)
**Liver diseases**	**760 (2.3)**	**747 (2.4)**	**589 (2.5)**	**452 (2.7)**	**329 (2.6)**
Prior 4 years	306 (0.9)	324 (1.0)	302 (1.3)	230 (1.4)	178 (1.4)
Index hospitalization	512 (1.6)	470 (1.5)	344 (1.5)	272 (1.6)	199 (1.6)
**Gastroesophageal reflux disease**	**161 (0.5)**	**145 (0.5)**	**134 (0.6)**	**103 (0.6)**	**85 (0.7)**
Prior 4 years	95 (0.3)	108 (0.3)	96 (0.4)	74 (0.4)	65 (0.5)
Index hospitalization	67 (0.2)	41 (0.1)	43 (0.2)	32 (0.2)	21 (0.2)
**Peptic ulcer disease**	**250 (0.8)**	**215 (0.7)**	**164 (0.7)**	**143 (0.8)**	**110 (0.9)**
Prior 4 years	124 (0.4)	106 (0.3)	98 (0.4)	76 (0.4)	63 (0.5)
Index hospitalization	131 (0.4)	116 (0.4)	68 (0.3)	71 (0.4)	50 (0.4)
**Chronic kidney diseases**	**4,379 (13.3)**	**4,529 (14.4)**	**4,158 (18.0)**	**3,570 (21.0)**	**2,662 (21.2)**
Prior 4 years	1,776 (5.4)	1,934 (6.2)	1,848 (8.0)	1,658 (9.8)	1,298 (10.3)
Index hospitalization	3,443 (10.5)	3,561 (11.3)	3,266 (14.1)	2,845 (16.8)	2,140 (17.0)
**Anaemia**	**3,428 (10.5)**	**3,488 (11.1)**	**2,906 (12.6)**	**2,388 (14.1)**	**1,884 (15.0)**
Prior 4 years	1,798 (5.5)	1,852 (5.9)	1,556 (6.7)	1,288 (7.6)	1,025 (8.2)
Index hospitalization	1,989 (6.1)	2,014 (6.4)	1,677 (7.2)	1,396 (8.2)	1,096 (8.7)
**Coagulopathy**	**551 (1.7)**	**794 (2.5)**	**673 (2.9)**	**652 (3.8)**	**582 (4.6)**
Prior 4 years	170 (0.5)	268 (0.9)	256 (1.1)	261 (1.5)	205 (1.6)
Index hospitalization	398 (1.2)	555 (1.8)	444 (1.9)	410 (2.4)	404 (3.2)
**Diabetes**	**6,782 (20.7)**	**6,794 (21.6)**	**5,296 (22.9)**	**4,057 (23.9)**	**3,084 (24.5)**
Prior 4 years	3,158 (9.6)	3,432 (10.9)	2,778 (12.0)	2,173 (12.8)	1,589 (12.6)
Index hospitalization	5,782 (17.6)	5,767 (18.4)	4,553 (19.7)	3,503 (20.6)	2,753 (21.9)

© 2023 Chen Q et al. *JAMA Network Open.*

**Thyroid disease**	**1,098 (3.3)**	**1,055 (3.4)**	**784 (3.4)**	**558 (3.3)**	**426 (3.4)**
Prior 4 years	343 (1.0)	349 (1.1)	293 (1.3)	232 (1.4)	166 (1.3)
Index hospitalization	837 (2.6)	795 (2.5)	572 (2.5)	386 (2.3)	319 (2.5)
**Ischemic stroke (history)**	**1,402 (4.3)**	**1,314 (4.2)**	**902 (3.9)**	**665 (3.9)**	**509 (4.1)**
Prior 4 years	949 (2.9)	925 (2.9)	620 (2.7)	467 (2.8)	342 (2.7)
Index hospitalization	632 (1.9)	521 (1.7)	366 (1.6)	277 (1.6)	241 (1.9)
**TIA**	**948 (2.9)**	**839 (2.7)**	**608 (2.6)**	**409 (2.4)**	**257 (2.0)**
Prior 4 years	506 (1.5)	448 (1.4)	342 (1.5)	243 (1.4)	145 (1.2)
Index hospitalization	462 (1.4)	408 (1.3)	290 (1.3)	179 (1.1)	124 (1.0)
**Other arterial thromboembolism**	**489 (1.5)**	**423 (1.3)**	**333 (1.4)**	**264 (1.6)**	**170 (1.4)**
Prior 4 years	372 (1.1)	299 (1.0)	241 (1.0)	169 (1.0)	118 (0.9)
Index hospitalization	141 (0.4)	151 (0.5)	107 (0.5)	106 (0.6)	58 (0.5)
**Parkinson's disease**	**254 (0.8)**	**339 (1.1)**	**229 (1.0)**	**181 (1.1)**	**132 (1.1)**
Prior 4 years	125 (0.4)	164 (0.5)	103 (0.4)	85 (0.5)	60 (0.5)
Index hospitalization	192 (0.6)	267 (0.9)	188 (0.8)	146 (0.9)	107 (0.9)
**Alzheimer's disease**	**248 (0.8)**	**245 (0.8)**	**213 (0.9)**	**159 (0.9)**	**135 (1.1)**
Prior 4 years	79 (0.2)	85 (0.3)	70 (0.3)	44 (0.3)	26 (0.2)
Index hospitalization	207 (0.6)	202 (0.6)	178 (0.8)	140 (0.8)	124 (1.0)
**Autoimmune disease**	**105 (0.3)**	**107 (0.3)**	**108 (0.5)**	**91 (0.5)**	**75 (0.6)**
Prior 4 years	66 (0.2)	71 (0.2)	81 (0.3)	59 (0.3)	50 (0.4)
Index hospitalization	52 (0.2)	51 (0.2)	37 (0.2)	47 (0.3)	34 (0.3)
**Systemic connective tissue disorders**	**413 (1.3)**	**429 (1.4)**	**367 (1.6)**	**249 (1.5)**	**222 (1.8)**
Prior 4 years	175 (0.5)	193 (0.6)	203 (0.9)	135 (0.8)	107 (0.9)
Index hospitalization	289 (0.9)	300 (1.0)	242 (1.0)	160 (0.9)	157 (1.2)
**Venous thromboembolism**	**723 (2.2)**	**734 (2.3)**	**544 (2.3)**	**355 (2.1)**	**266 (2.1)**
Prior 4 years to prior 6 months	244 (0.7)	263 (0.8)	206 (0.9)	135 (0.8)	118 (0.9)
Prior 6 months	501 (1.5)	494 (1.6)	359 (1.6)	229 (1.3)	157 (1.2)
**Deep vein thrombosis**	**293 (0.9)**	**294 (0.9)**	**208 (0.9)**	**148 (0.9)**	**108 (0.9)**
Prior 4 years to prior 6 months	104 (0.3)	109 (0.3)	70 (0.3)	50 (0.3)	41 (0.3)
Prior 6 months	193 (0.6)	192 (0.6)	141 (0.6)	100 (0.6)	67 (0.5)
**Pulmonary embolism**	**520 (1.6)**	**517 (1.6)**	**392 (1.7)**	**235 (1.4)**	**179 (1.4)**
Prior 4 years to prior 6 months	168 (0.5)	184 (0.6)	154 (0.7)	104 (0.6)	90 (0.7)
Prior 6 months	367 (1.1)	346 (1.1)	254 (1.1)	137 (0.8)	97 (0.8)
**Major bleeding**	**2,221 (6.8)**	**2,213 (7.1)**	**1,691 (7.3)**	**1,510 (8.9)**	**1,132 (9.0)**
Prior 4 years	1,287 (3.9)	1,280 (4.1)	997 (4.3)	890 (5.2)	648 (5.2)
Index hospitalization	1,060 (3.2)	1,067 (3.4)	789 (3.4)	706 (4.2)	550 (4.4)
**Intracranial haemorrhage**	**238 (0.7)**	**202 (0.6)**	**117 (0.5)**	**111 (0.7)**	**89 (0.7)**

© 2023 Chen Q et al. *JAMA Network Open.*

Prior 4 years	120 (0.4)	100 (0.3)	55 (0.2)	63 (0.4)	37 (0.3)
Index hospitalization	129 (0.4)	118 (0.4)	64 (0.3)	57 (0.3)	55 (0.4)
**Gastrointestinal bleeding**	**1,013 (3.1)**	**914 (2.9)**	**729 (3.1)**	**645 (3.8)**	**463 (3.7)**
Prior 4 years	614 (1.9)	583 (1.9)	457 (2.0)	401 (2.4)	296 (2.4)
Index hospitalization	442 (1.3)	373 (1.2)	308 (1.3)	280 (1.6)	194 (1.5)
**Malignant tumour**	**3,852 (11.7)**	**3,825 (12.2)**	**2,875 (12.4)**	**2,192 (12.9)**	**1,511 (12.0)**
Prior 4 years	3,033 (9.2)	3,004 (9.6)	2,253 (9.7)	1,711 (10.1)	1,161 (9.2)
Index hospitalization	1,800 (5.5)	1,929 (6.1)	1,438 (6.2)	1,061 (6.2)	747 (5.9)
≥1 hospitalization within the prior 4 years, n (%)	21,747 (66.3)	20,836 (66.4)	15,554 (67.2)	11,538 (68.0)	8,486 (67.5)
Information about the index hospitalization					
Length of hospital stay, days, median (IQR)	4 (1-8)	4 (1-8)	4 (1-8)	4 (1-8)	4 (1-9)
In-hospital mortality, n (%)	31 (0.1)	35 (0.1)	<10 (0.0)[‖]	19 (0.1)	17 (0.1)
**Main reason for admission to the index hospitalization**					
Atrial fibrillation	11,547 (35.2)	10,603 (33.8)	7,039 (30.4)	4,425 (26.1)	2,816 (22.4)
Heart failure	3,255 (9.9)	3,199 (10.2)	2,512 (10.9)	1,876 (11.0)	1,415 (11.3)
Ischemic heart diseases	2,669 (8.1)	2,622 (8.4)	1,975 (8.5)	1,425 (8.4)	1,064 (8.5)
Respiratory diseases	2,226 (6.8)	2,468 (7.9)	1,781 (7.7)	1,451 (8.5)	1,121 (8.9)
Injury/poisoning	1,488 (4.5)	1,505 (4.8)	1,296 (5.6)	1,074 (6.3)	947 (7.5)
Symptoms/signs/abnormal laboratory findings[**]	1,780 (5.4)	1,718 (5.5)	1,286 (5.6)	936 (5.5)	680 (5.4)
Neoplasms	1,115 (3.4)	1,215 (3.9)	836 (3.6)	622 (3.7)	462 (3.7)
Digestive diseases	1,308 (4.0)	1,257 (4.0)	869 (3.8)	748 (4.4)	526 (4.2)
Other heart valve disorders[§§]	1,134 (3.5)	1,078 (3.4)	1,073 (4.6)	912 (5.4)	779 (6.2)
Genitourinary diseases	844 (2.6)	775 (2.5)	719 (3.1)	583 (3.4)	483 (3.8)
Infectious/parasitic diseases	496 (1.5)	484 (1.5)	350 (1.5)	334 (2.0)	260 (2.1)
Endocrine/nutritional/metabolic diseases	379 (1.2)	349 (1.1)	265 (1.1)	194 (1.1)	194 (1.5)
Diseases of blood/blood-forming organs	266 (0.8)	232 (0.7)	203 (0.9)	173 (1.0)	111 (0.9)
Neurological disorders[##]	242 (0.7)	203 (0.6)	128 (0.6)	113 (0.7)	79 (0.6)

† First-generation immigrants refer to persons who were born abroad with at least one parent who was born abroad; second-generation immigrants refer to persons who was born in the Netherlands with at least one parent who was born abroad.
‡ Percentile groups were determined based on disposable income of private households of the complete target population in the database (instead of the study population only).
* Labile INR (international normalized ratio) was not included for calculation.
§ Pre-existing chronic use was defined as ≥2 (outpatient) prescription records of the same type of antithrombotic agents within six months (*i.e.*, 183 days) before the index dates (exclusive). Pre-existing chronic use of oral anticoagulant refers to persons with pre-existing chronic use of vitamin K antagonist or direct oral anticoagulant.
|| Unless otherwise stated, for each comorbidity (or medical history), prevalence calculated by screening all hospitalizations within the prior four years (including the index hospitalization), all hospitalizations within the prior four years (excluding the index hospitalization), and only the index hospitalization is presented, respectively.
Except for rheumatic mitral stenosis and mechanical heart valves
** Except for abnormalities of heart beat (ICD-10 code: R00) and cardiac murmurs and other cardiac sounds (ICD-10 code: R01)
§§ Except for endocarditis, valve unspecified, in diseases classified elsewhere (ICD-10 code: I398)

© 2023 Chen Q et al. *JAMA Network Open.*

Except for transient cerebral ischaemic attacks and related syndromes (ICD-10 code: G45) and vascular syndromes of brain in cerebrovascular diseases (ICD-10 code: G46)
||| Exact result was masked to prevent potential individual or group disclosure.
Abbreviations: SD, standard deviation; TIA, transient ischemic attack; IQR, interquartile range; ICD, International Classification of Diseases.

© 2023 Chen Q et al. *JAMA Network Open.*

eTable 10. Time Trends in Patient Characteristics of Incident Nonvalvular Atrial Fibrillation Among Patients Who First Received Direct Oral Anticoagulant as Oral Anticoagulant Within the 1-Year Follow-up

Cohort	2014 (N=5,102)	2015 (N=13,039)	2016 (N=20,615)	2017 (N=28,165)	2018 (N=32,314)
Age, years, mean ± SD	69.9 ± 11.0	70.4 ± 11.0	71.8 ± 11.0	72.9 ± 10.9	73.4 ± 10.9
Age group, years, n (%)					
18-24	[masked][‖]	[masked][‖]	11 (0.1)	17 (0.1)	24 (0.1)
25-34	[masked][‖]	[masked][‖]	52 (0.3)	48 (0.2)	87 (0.3)
35-44	75 (1.5)	200 (1.5)	219 (1.1)	266 (0.9)	281 (0.9)
45-54	380 (7.4)	897 (6.9)	1,203 (5.8)	1,388 (4.9)	1,448 (4.5)
55-64	1,033 (20.2)	2,518 (19.3)	3,530 (17.1)	4,372 (15.5)	4,658 (14.4)
65-74	1,924 (37.7)	4,827 (37.0)	7,260 (35.2)	9,619 (34.2)	10,950 (33.9)
75-84	1,275 (25.0)	3,457 (26.5)	6,054 (29.4)	8,706 (30.9)	10,357 (32.1)
≥85	395 (7.7)	1,092 (8.4)	2,286 (11.1)	3,749 (13.3)	4,509 (14.0)
Sex, n (%)					
Male	3,113 (61.0)	7,489 (57.4)	11,743 (57.0)	15,898 (56.4)	18,401 (56.9)
Female	1,989 (39.0)	5,550 (42.6)	8,872 (43.0)	12,267 (43.6)	13,913 (43.1)
Immigration background[†], n (%)					
Native Dutch	4,499 (88.2)	11,433 (87.7)	18,029 (87.5)	24,696 (87.7)	28,343 (87.7)
First-generation immigrants	316 (6.2)	843 (6.5)	1,365 (6.6)	1,864 (6.6)	2,213 (6.8)
Second-generation immigrants	287 (5.6)	763 (5.9)	1,221 (5.9)	1,605 (5.7)	1,758 (5.4)
Standardized household income[‡], n (%)					
First quintile (0-20%)	595 (11.7)	1,567 (12.0)	2,851 (13.8)	4,148 (14.8)	4,935 (15.3)
Second quintile (20-40%)	1,176 (23.1)	3,233 (24.8)	5,415 (26.3)	7,764 (27.6)	9,109 (28.2)
Third quintile (40-60%)	1,261 (24.7)	3,209 (24.7)	5,072 (24.6)	7,044 (25.1)	8,138 (25.2)
Fourth quintile (60-80%)	990 (19.4)	2,516 (19.3)	3,800 (18.5)	4,800 (17.1)	5,326 (16.5)
Fifth quintile (80-100%)	1,012 (19.9)	2,322 (17.8)	3,164 (15.4)	3,944 (14.0)	4,227 (13.1)
Private household with an unknown income	[masked][‖]	10 (0.1)	17 (0.1)	13 (0.0)	11 (0.0)
Institutional household	[masked][‖]	156 (1.2)	273 (1.3)	385 (1.4)	499 (1.5)
Marital status, n (%)					
Married or in partnership	3,299 (64.7)	8,337 (63.9)	12,649 (61.4)	16,738 (59.4)	18,984 (58.7)
Unmarried or single	369 (7.2)	958 (7.3)	1,510 (7.3)	2,124 (7.5)	2,447 (7.6)
Divorced	554 (10.9)	1,414 (10.8)	2,202 (10.7)	3,096 (11.0)	3,610 (11.2)
Widowed	880 (17.2)	2,330 (17.9)	4,254 (20.6)	6,207 (22.0)	7,273 (22.5)
Type of atrial fibrillation, n (%)					
Paroxysmal atrial fibrillation (I48.0)	-	2,282 (17.5)	3,519 (17.1)	4,747 (16.9)	5,071 (15.7)
Persistent atrial fibrillation (I48.1)	-	547 (4.2)	817 (4.0)	1,079 (3.8)	1,249 (3.9)

© 2023 Chen Q et al. *JAMA Network Open.*

Chronic atrial fibrillation (I48.2)	-	312 (2.4)	514 (2.5)	783 (2.8)	970 (3.0)
Type I atrial flutter (I48.3)	-	111 (0.9)	202 (1.0)	331 (1.2)	441 (1.4)
Type II atrial flutter (I48.4)	-	54 (0.4)	65 (0.3)	71 (0.3)	78 (0.2)
Unspecified atrial fibrillation (I48.9)	-	9,733 (74.6)	15,498 (75.2)	21,154 (75.1)	24,505 (75.8)
Diagnosis made before 2015	5,102 (100.0)	-	-	-	-
CHA_2DS_2-VASc score, mean ± SD	2.24 ± 1.57	2.32 ± 1.55	2.54 ± 1.59	2.71 ± 1.62	2.77 ± 1.61
CHA_2DS_2-VASc score, n (%)					
0	672 (13.2)	1,576 (12.1)	2,040 (9.9)	2,316 (8.2)	2,452 (7.6)
1	1,121 (22.0)	2,690 (20.6)	3,615 (17.5)	4,506 (16.0)	4,978 (15.4)
**≥2**	**3,309 (64.9)**	**8,773 (67.3)**	**14,960 (72.6)**	**21,343 (75.8)**	**24,884 (77.0)**
2	1,310 (25.7)	3,249 (24.9)	4,983 (24.2)	6,435 (22.8)	7,098 (22.0)
3	968 (19.0)	2,704 (20.7)	4,589 (22.3)	6,469 (23.0)	7,538 (23.3)
4	572 (11.2)	1,657 (12.7)	3,015 (14.6)	4,688 (16.6)	5,649 (17.5)
5	291 (5.7)	765 (5.9)	1,546 (7.5)	2,355 (8.4)	2,943 (9.1)
6	126 (2.5)	298 (2.3)	585 (2.8)	967 (3.4)	1,202 (3.7)
≥7	42 (0.8)	100 (0.8)	242 (1.2)	429 (1.5)	454 (1.4)
HAS-BLED score*, mean ± SD	1.51 ± 0.96	1.49 ± 0.97	1.57 ± 0.98	1.62 ± 0.98	1.65 ± 0.98
HAS-BLED score*, n (%)					
0	734 (14.4)	1,939 (14.9)	2,595 (12.6)	3,175 (11.3)	3,444 (10.7)
1	1,901 (37.3)	4,959 (38.0)	7,596 (36.8)	10,244 (36.4)	11,744 (36.3)
2	1,687 (33.1)	4,264 (32.7)	7,014 (34.0)	9,712 (34.5)	11,095 (34.3)
≥3	780 (15.3)	1,877 (14.4)	3,410 (16.5)	5,034 (17.9)	6,031 (18.7)
Pre-existing chronic use of antithrombotic agents§					
**Oral anticoagulant, n (%)**	**1,914 (37.5)**	**4,126 (31.6)**	**6,027 (29.2)**	**8,823 (31.3)**	**11,301 (35.0)**
Vitamin K antagonist, n (%)	238 (4.7)	418 (3.2)	550 (2.7)	695 (2.5)	699 (2.2)
Direct oral anticoagulant, n (%)	1,716 (33.6)	3,799 (29.1)	5,602 (27.2)	8,249 (29.3)	10,692 (33.1)
Heparin group, n (%)	24 (0.5)	69 (0.5)	114 (0.6)	166 (0.6)	180 (0.6)
Antiplatelet agent, n (%)	1,349 (26.4)	3,319 (25.5)	5,648 (27.4)	7,844 (27.9)	8,707 (26.9)
Comorbidities (or medical history)‖, n (%)					
**Asthma**	**80 (1.6)**	**227 (1.7)**	**431 (2.1)**	**606 (2.2)**	**843 (2.6)**
Prior 4 years	32 (0.6)	106 (0.8)	217 (1.1)	293 (1.0)	443 (1.4)
Index hospitalization	59 (1.2)	139 (1.1)	285 (1.4)	409 (1.5)	574 (1.8)
**Chronic obstructive pulmonary disease**	**391 (7.7)**	**989 (7.6)**	**1,961 (9.5)**	**2,827 (10.0)**	**3,324 (10.3)**
Prior 4 years	175 (3.4)	509 (3.9)	1,011 (4.9)	1,481 (5.3)	1,745 (5.4)
Index hospitalization	314 (6.2)	787 (6.0)	1,601 (7.8)	2,264 (8.0)	2,751 (8.5)
**Other chronic lung diseases**	**62 (1.2)**	**133 (1.0)**	**282 (1.4)**	**470 (1.7)**	**631 (2.0)**
Prior 4 years	37 (0.7)	71 (0.5)	156 (0.8)	237 (0.8)	302 (0.9)

© 2023 Chen Q et al. *JAMA Network Open.*

Index hospitalization	27 (0.5)	78 (0.6)	153 (0.7)	289 (1.0)	415 (1.3)
**Heart failure**	**570 (11.2)**	**1,605 (12.3)**	**3,032 (14.7)**	**4,849 (17.2)**	**6,013 (18.6)**
Prior 4 years	104 (2.0)	275 (2.1)	586 (2.8)	970 (3.4)	1,179 (3.6)
Index hospitalization	504 (9.9)	1,437 (11.0)	2,710 (13.1)	4,280 (15.2)	5,378 (16.6)
**Myocardial infarction (history)**	**351 (6.9)**	**885 (6.8)**	**1,845 (8.9)**	**2,937 (10.4)**	**3,514 (10.9)**
Prior 4 years	257 (5.0)	637 (4.9)	1,281 (6.2)	1,942 (6.9)	2,235 (6.9)
Index hospitalization	105 (2.1)	288 (2.2)	696 (3.4)	1,260 (4.5)	1,621 (5.0)
**Hypertension**	**1,560 (30.6)**	**3,977 (30.5)**	**6,952 (33.7)**	**10,158 (36.1)**	**12,187 (37.7)**
Prior 4 years	471 (9.2)	1,343 (10.3)	2,643 (12.8)	4,116 (14.6)	5,027 (15.6)
Index hospitalization	1,317 (25.8)	3,283 (25.2)	5,752 (27.9)	8,289 (29.4)	10,072 (31.2)
**Other valvular heart disease**[#]	**425 (8.3)**	**1,019 (7.8)**	**1,828 (8.9)**	**2,845 (10.1)**	**3,471 (10.7)**
Prior 4 years	115 (2.3)	291 (2.2)	654 (3.2)	1,056 (3.7)	1,273 (3.9)
Index hospitalization	345 (6.8)	841 (6.4)	1,505 (7.3)	2,328 (8.3)	2,842 (8.8)
**Peripheral artery disease**	**80 (1.6)**	**222 (1.7)**	**393 (1.9)**	**646 (2.3)**	**764 (2.4)**
Prior 4 years	40 (0.8)	116 (0.9)	235 (1.1)	384 (1.4)	439 (1.4)
Index hospitalization	46 (0.9)	141 (1.1)	224 (1.1)	371 (1.3)	441 (1.4)
**Liver diseases**	**62 (1.2)**	**204 (1.6)**	**334 (1.6)**	**512 (1.8)**	**621 (1.9)**
Prior 4 years	23 (0.5)	93 (0.7)	156 (0.8)	241 (0.9)	316 (1.0)
Index hospitalization	40 (0.8)	123 (0.9)	203 (1.0)	312 (1.1)	362 (1.1)
**Gastroesophageal reflux disease**	**14 (0.3)**	**45 (0.3)**	**96 (0.5)**	**172 (0.6)**	**178 (0.6)**
Prior 4 years	[masked][‖]	32 (0.2)	71 (0.3)	125 (0.4)	130 (0.4)
Index hospitalization	[masked][‖]	13 (0.1)	26 (0.1)	50 (0.2)	51 (0.2)
**Peptic ulcer disease**	**11 (0.2)**	**45 (0.3)**	**81 (0.4)**	**132 (0.5)**	**150 (0.5)**
Prior 4 years	[masked][‖]	27 (0.2)	56 (0.3)	73 (0.3)	78 (0.2)
Index hospitalization	[masked][‖]	19 (0.1)	26 (0.1)	61 (0.2)	76 (0.2)
**Chronic kidney diseases**	**221 (4.3)**	**646 (5.0)**	**1,482 (7.2)**	**2,408 (8.5)**	**2,968 (9.2)**
Prior 4 years	75 (1.5)	228 (1.7)	591 (2.9)	945 (3.4)	1,258 (3.9)
Index hospitalization	165 (3.2)	489 (3.8)	1,092 (5.3)	1,802 (6.4)	2,260 (7.0)
**Anaemia**	**233 (4.6)**	**581 (4.5)**	**1,381 (6.7)**	**2,342 (8.3)**	**2,963 (9.2)**
Prior 4 years	135 (2.6)	320 (2.5)	732 (3.6)	1,204 (4.3)	1,502 (4.6)
Index hospitalization	114 (2.2)	300 (2.3)	767 (3.7)	1,392 (4.9)	1,786 (5.5)
**Coagulopathy**	**29 (0.6)**	**97 (0.7)**	**185 (0.9)**	**311 (1.1)**	**454 (1.4)**
Prior 4 years	14 (0.3)	41 (0.3)	79 (0.4)	132 (0.5)	187 (0.6)
Index hospitalization	16 (0.3)	59 (0.5)	117 (0.6)	191 (0.7)	288 (0.9)
**Diabetes**	**659 (12.9)**	**1,687 (12.9)**	**3,138 (15.2)**	**4,675 (16.6)**	**5,562 (17.2)**
Prior 4 years	271 (5.3)	739 (5.7)	1,515 (7.3)	2,361 (8.4)	2,793 (8.6)
Index hospitalization	546 (10.7)	1,430 (11.0)	2,656 (12.9)	3,992 (14.2)	4,885 (15.1)

© 2023 Chen Q et al. *JAMA Network Open.*

**Thyroid disease**	**132 (2.6)**	**351 (2.7)**	**647 (3.1)**	**978 (3.5)**	**1,037 (3.2)**
Prior 4 years	32 (0.6)	86 (0.7)	202 (1.0)	301 (1.1)	328 (1.0)
Index hospitalization	108 (2.1)	291 (2.2)	492 (2.4)	773 (2.7)	821 (2.5)
**Ischemic stroke (history)**	**170 (3.3)**	**395 (3.0)**	**667 (3.2)**	**993 (3.5)**	**1,094 (3.4)**
Prior 4 years	119 (2.3)	296 (2.3)	530 (2.6)	789 (2.8)	822 (2.5)
Index hospitalization	71 (1.4)	146 (1.1)	189 (0.9)	318 (1.1)	395 (1.2)
**TIA**	**133 (2.6)**	**335 (2.6)**	**606 (2.9)**	**774 (2.7)**	**851 (2.6)**
Prior 4 years	54 (1.1)	175 (1.3)	282 (1.4)	422 (1.5)	439 (1.4)
Index hospitalization	83 (1.6)	171 (1.3)	347 (1.7)	371 (1.3)	440 (1.4)
**Other arterial thromboembolism**	**21 (0.4)**	**79 (0.6)**	**151 (0.7)**	**225 (0.8)**	**235 (0.7)**
Prior 4 years	[masked][‖]	65 (0.5)	111 (0.5)	153 (0.5)	158 (0.5)
Index hospitalization	[masked][‖]	18 (0.1)	44 (0.2)	79 (0.3)	81 (0.3)
**Parkinson's disease**	**24 (0.5)**	**73 (0.6)**	**155 (0.8)**	**254 (0.9)**	**272 (0.8)**
Prior 4 years	12 (0.2)	32 (0.2)	70 (0.3)	122 (0.4)	110 (0.3)
Index hospitalization	18 (0.4)	56 (0.4)	122 (0.6)	206 (0.7)	242 (0.7)
**Alzheimer's disease**	**12 (0.2)**	**45 (0.3)**	**84 (0.4)**	**141 (0.5)**	**184 (0.6)**
Prior 4 years	[masked][‖]	18 (0.1)	30 (0.1)	38 (0.1)	42 (0.1)
Index hospitalization	[masked][‖]	36 (0.3)	71 (0.3)	120 (0.4)	166 (0.5)
**Autoimmune disease**	**12 (0.2)**	**32 (0.2)**	**75 (0.4)**	**112 (0.4)**	**129 (0.4)**
Prior 4 years	[masked][‖]	21 (0.2)	55 (0.3)	78 (0.3)	92 (0.3)
Index hospitalization	[masked][‖]	14 (0.1)	36 (0.2)	52 (0.2)	57 (0.2)
**Systemic connective tissue disorders**	**28 (0.5)**	**111 (0.9)**	**216 (1.0)**	**336 (1.2)**	**433 (1.3)**
Prior 4 years	11 (0.2)	50 (0.4)	122 (0.6)	199 (0.7)	214 (0.7)
Index hospitalization	19 (0.4)	78 (0.6)	135 (0.7)	206 (0.7)	300 (0.9)
**Venous thromboembolism**	**36 (0.7)**	**98 (0.8)**	**278 (1.3)**	**576 (2.0)**	**652 (2.0)**
Prior 4 years to prior 6 months	19 (0.4)	46 (0.4)	95 (0.5)	164 (0.6)	185 (0.6)
Prior 6 months	17 (0.3)	57 (0.4)	191 (0.9)	434 (1.5)	479 (1.5)
**Deep vein thrombosis**	**14 (0.3)**	**47 (0.4)**	**98 (0.5)**	**216 (0.8)**	**217 (0.7)**
Prior 4 years to prior 6 months	[masked][‖]	20 (0.2)	36 (0.2)	68 (0.2)	63 (0.2)
Prior 6 months	[masked][‖]	28 (0.2)	64 (0.3)	152 (0.5)	157 (0.5)
**Pulmonary embolism**	**25 (0.5)**	**64 (0.5)**	**220 (1.1)**	**420 (1.5)**	**492 (1.5)**
Prior 4 years to prior 6 months	15 (0.3)	32 (0.2)	74 (0.4)	117 (0.4)	142 (0.4)
Prior 6 months	10 (0.2)	35 (0.3)	151 (0.7)	315 (1.1)	359 (1.1)
**Major bleeding**	**177 (3.5)**	**490 (3.8)**	**835 (4.1)**	**1,403 (5.0)**	**1,733 (5.4)**
Prior 4 years	111 (2.2)	293 (2.2)	523 (2.5)	811 (2.9)	1,008 (3.1)
Index hospitalization	72 (1.4)	215 (1.6)	352 (1.7)	648 (2.3)	810 (2.5)
**Intracranial haemorrhage**	**29 (0.6)**	**70 (0.5)**	**100 (0.5)**	**159 (0.6)**	**181 (0.6)**

© 2023 Chen Q et al. *JAMA Network Open.*

Prior 4 years	13 (0.3)	29 (0.2)	51 (0.2)	85 (0.3)	86 (0.3)
Index hospitalization	17 (0.3)	44 (0.3)	55 (0.3)	81 (0.3)	109 (0.3)
**Gastrointestinal bleeding**	**72 (1.4)**	**207 (1.6)**	**371 (1.8)**	**637 (2.3)**	**751 (2.3)**
Prior 4 years	50 (1.0)	133 (1.0)	269 (1.3)	395 (1.4)	477 (1.5)
Index hospitalization	22 (0.4)	79 (0.6)	118 (0.6)	259 (0.9)	304 (0.9)
**Malignant tumour**	**398 (7.8)**	**1,031 (7.9)**	**1,973 (9.6)**	**2,960 (10.5)**	**3,620 (11.2)**
Prior 4 years	327 (6.4)	822 (6.3)	1,592 (7.7)	2,398 (8.5)	2,869 (8.9)
Index hospitalization	173 (3.4)	453 (3.5)	935 (4.5)	1,485 (5.3)	1,928 (6.0)
≥1 hospitalization within the prior 4 years, n (%)	2,877 (56.4)	7,173 (55.0)	11,878 (57.6)	16,490 (58.5)	18,660 (57.7)
Information about the index hospitalization					
Length of hospital stay, days, median (IQR)	1 (0-4)	1 (0-4)	1 (0-5)	2 (0-6)	2 (0-6)
In-hospital mortality, n (%)	<10 (<0.1)[‖]	10 (0.1)	20 (0.1)	34 (0.1)	61 (0.2)
**Main reason for admission to the index hospitalization**					
Atrial fibrillation	3,217 (63.1)	8,415 (64.5)	11,818 (57.3)	14,246 (50.6)	15,276 (47.3)
Heart failure	255 (5.0)	722 (5.5)	1,280 (6.2)	2,019 (7.2)	2,510 (7.8)
Ischemic heart diseases	205 (4.0)	509 (3.9)	1,095 (5.3)	1,817 (6.5)	2,227 (6.9)
Respiratory diseases	161 (3.2)	553 (4.2)	1,151 (5.6)	1,789 (6.4)	2,303 (7.1)
Injury/poisoning	114 (2.2)	262 (2.0)	545 (2.6)	907 (3.2)	1,123 (3.5)
Symptoms/signs/abnormal laboratory findings[**]	223 (4.4)	470 (3.6)	778 (3.8)	1,121 (4.0)	1293 (4.0)
Neoplasms	100 (2.0)	260 (2.0)	555 (2.7)	867 (3.1)	1,116 (3.5)
Digestive diseases	125 (2.5)	286 (2.2)	497 (2.4)	868 (3.1)	1,018 (3.2)
Other heart valve disorders[§§]	40 (0.8)	88 (0.7)	222 (1.1)	362 (1.3)	463 (1.4)
Genitourinary diseases	66 (1.3)	142 (1.1)	272 (1.3)	503 (1.8)	679 (2.1)
Infectious/parasitic diseases	27 (0.5)	82 (0.6)	229 (1.1)	380 (1.3)	452 (1.4)
Endocrine/nutritional/metabolic diseases	23 (0.5)	69 (0.5)	124 (0.6)	234 (0.8)	289 (0.9)
Diseases of blood/blood-forming organs	15 (0.3)	40 (0.3)	83 (0.4)	155 (0.6)	199 (0.6)
Neurological disorders[##]	44 (0.9)	65 (0.5)	106 (0.5)	168 (0.6)	166 (0.5)

† First-generation immigrants refer to persons who were born abroad with at least one parent who was born abroad; second-generation immigrants refer to persons who was born in the Netherlands with at least one parent who was born abroad.
‡ Percentile groups were determined based on disposable income of private households of the complete target population in the database (instead of the study population only).
* Labile INR (international normalized ratio) was not included for calculation.
§ Pre-existing chronic use was defined as ≥2 (outpatient) prescription records of the same type of antithrombotic agents within six months (*i.e.*, 183 days) before the index dates (exclusive). Pre-existing chronic use of oral anticoagulant refers to persons with pre-existing chronic use of vitamin K antagonist or direct oral anticoagulant.
|| Unless otherwise stated, for each comorbidity (or medical history), prevalence calculated by screening all hospitalizations within the prior four years (including the index hospitalization), all hospitalizations within the prior four years (excluding the index hospitalization), and only the index hospitalization is presented, respectively.
Except for rheumatic mitral stenosis and mechanical heart valves
** Except for abnormalities of heart beat (ICD-10 code: R00) and cardiac murmurs and other cardiac sounds (ICD-10 code: R01)
§§ Except for endocarditis, valve unspecified, in diseases classified elsewhere (ICD-10 code: I398)

© 2023 Chen Q et al. *JAMA Network Open.*

Except for transient cerebral ischaemic attacks and related syndromes (ICD-10 code: G45) and vascular syndromes of brain in cerebrovascular diseases (ICD-10 code: G46)
||| Exact result was masked to prevent potential individual or group disclosure.
Abbreviations: SD, standard deviation; TIA, transient ischemic attack; IQR, interquartile range; ICD, International Classification of Diseases.

© 2023 Chen Q et al. *JAMA Network Open.*

eTable 11. Time Trends in Patient Characteristics of Incident Nonvalvular Atrial Fibrillation Among Patients Who Did Not Receive Any Oral Anticoagulants Within the 1-Year Follow-up, Excluding Patients With Preexisting Chronic Oral Anticoagulant Treatment

Cohort	2014 (N=16,248)	2015 (N=15,027)	2016 (N=14,562)	2017 (N=15,557)	2018 (N=15,028)
Age, years, mean ± SD	72.9 ± 14.0	72.7 ± 14.7	72.3 ± 14.6	72.1 ± 14.4	72.0 ± 14.2
Age group, years, n (%)					
18-24	58 (0.4)	58 (0.4)	60 (0.4)	65 (0.4)	65 (0.4)
25-34	147 (0.9)	169 (1.1)	175 (1.2)	194 (1.2)	168 (1.1)
35-44	355 (2.2)	404 (2.7)	387 (2.7)	393 (2.5)	356 (2.4)
45-54	1,153 (7.1)	1,234 (8.2)	1,190 (8.2)	1,199 (7.7)	1,144 (7.6)
55-64	2,666 (16.4)	2,425 (16.1)	2,531 (17.4)	2,743 (17.6)	2,709 (18.0)
65-74	4,027 (24.8)	3,481 (23.2)	3,421 (23.5)	3,953 (25.4)	3,911 (26.0)
75-84	4,350 (26.8)	3,771 (25.1)	3,630 (24.9)	3,836 (24.7)	3,717 (24.7)
≥85	3,492 (21.5)	3,485 (23.2)	3,168 (21.8)	3,174 (20.4)	2,958 (19.7)
Sex, n (%)					
Male	9,207 (56.7)	8,578 (57.1)	8,399 (57.7)	8,946 (57.5)	8,652 (57.6)
Female	7,041 (43.3)	6,449 (42.9)	6,163 (42.3)	6,611 (42.5)	6,376 (42.4)
Immigration background†, n (%)					
Native Dutch	14,212 (87.5)	13,130 (87.4)	12,575 (86.4)	13,385 (86.0)	12,892 (85.8)
First-generation immigrants	1,145 (7.0)	1,059 (7.0)	1,161 (8.0)	1,280 (8.2)	1,287 (8.6)
Second-generation immigrants	891 (5.5)	838 (5.6)	826 (5.7)	892 (5.7)	849 (5.6)
Standardized household income‡, n (%)					
First quintile (0-20%)	3,072 (19.0)	3,098 (20.7)	3,018 (20.8)	3,332 (21.5)	3,321 (22.2)
Second quintile (20-40%)	3,916 (24.2)	3,457 (23.1)	3,431 (23.6)	3,764 (24.3)	3,507 (23.4)
Third quintile (40-60%)	3,475 (21.4)	2,923 (19.5)	2,935 (20.2)	3,150 (20.3)	3,031 (20.2)
Fourth quintile (60-80%)	2,450 (15.1)	2,290 (15.3)	2,136 (14.7)	2,287 (14.7)	2,222 (14.8)
Fifth quintile (80-100%)	2,157 (13.3)	2,098 (14.0)	1,951 (13.4)	2,077 (13.4)	2,049 (13.7)
Private household with an unknown income	11 (0.1)	16 (0.1)	25 (0.2)	16 (0.1)	21 (0.1)
Institutional household	1,130 (7.0)	1,105 (7.4)	1,024 (7.1)	880 (5.7)	839 (5.6)
Marital status, n (%)					
Married or in partnership	8,738 (53.8)	7,864 (52.3)	7,555 (51.9)	8,180 (52.6)	8,007 (53.3)
Unmarried or single	1,361 (8.4)	1,449 (9.6)	1,589 (10.9)	1,681 (10.8)	1,605 (10.7)
Divorced	1,697 (10.4)	1,625 (10.8)	1,630 (11.2)	1,819 (11.7)	1,772 (11.8)
Widowed	4,452 (27.4)	4,089 (27.2)	3,788 (26.0)	3,877 (24.9)	3,644 (24.2)
Type of atrial fibrillation, n (%)					
Paroxysmal atrial fibrillation (I48.0)	-	2,412 (16.1)	2,256 (15.5)	2,260 (14.5)	2,010 (13.4)
Persistent atrial fibrillation (I48.1)	-	293 (1.9)	309 (2.1)	419 (2.7)	385 (2.6)

© 2023 Chen Q et al. *JAMA Network Open.*

Chronic atrial fibrillation (I48.2)	-	489 (3.3)	437 (3.0)	446 (2.9)	431 (2.9)
Type I atrial flutter (I48.3)	-	90 (0.6)	81 (0.6)	124 (0.8)	148 (1.0)
Type II atrial flutter (I48.4)	-	32 (0.2)	34 (0.2)	26 (0.2)	24 (0.2)
Unspecified atrial fibrillation (I48.9)	-	11,711 (77.9)	11,445 (78.6)	12,282 (78.9)	12,030 (80.1)
Diagnosis made before 2015	16,248 (100.0)	-	-	-	-
CHA_2DS_2-VASc score, mean ± SD	2.79 ± 1.75	2.75 ± 1.80	2.71 ± 1.79	2.72 ± 1.79	2.72 ± 1.77
CHA_2DS_2-VASc score, n (%)					
0	1,666 (10.3)	1,834 (12.2)	1,816 (12.5)	1,877 (12.1)	1,728 (11.5)
1	2,514 (15.5)	2,371 (15.8)	2,341 (16.1)	2,542 (16.3)	2,489 (16.6)
**≥2**	**12,068 (74.3)**	**10,822 (72.0)**	**10,405 (71.5)**	**11,138 (71.6)**	**10,811 (71.9)**
2	3,023 (18.6)	2,522 (16.8)	2,581 (17.7)	2,806 (18.0)	2,778 (18.5)
3	3,572 (22.0)	3,204 (21.3)	3,073 (21.1)	3,213 (20.7)	3,109 (20.7)
4	2,757 (17.0)	2,590 (17.2)	2,391 (16.4)	2,574 (16.5)	2,475 (16.5)
5	1,650 (10.2)	1,471 (9.8)	1,388 (9.5)	1,534 (9.9)	1,495 (9.9)
6	739 (4.5)	692 (4.6)	663 (4.6)	668 (4.3)	654 (4.4)
≥7	327 (2.0)	343 (2.3)	309 (2.1)	343 (2.2)	300 (2.0)
HAS-BLED score*, mean ± SD	1.74 ± 1.09	1.68 ± 1.12	1.65 ± 1.12	1.64 ± 1.11	1.62 ± 1.10
HAS-BLED score*, n (%)					
0	2,046 (12.6)	2,226 (14.8)	2,298 (15.8)	2,426 (15.6)	2,383 (15.9)
1	4,971 (30.6)	4,714 (31.4)	4,624 (31.8)	5,013 (32.2)	4,827 (32.1)
2	5,469 (33.7)	4,760 (31.7)	4,480 (30.8)	4,810 (30.9)	4,783 (31.8)
≥3	3,762 (23.2)	3,327 (22.1)	3,160 (21.7)	3,308 (21.3)	3,035 (20.2)
Pre-existing chronic use of antithrombotic agents§					
**Oral anticoagulant, n (%)**	-	-	-	-	-
Vitamin K antagonist, n (%)	-	-	-	-	-
Direct oral anticoagulant, n (%)	-	-	-	-	-
Heparin group, n (%)	195 (1.2)	183 (1.2)	219 (1.5)	180 (1.2)	125 (0.8)
Antiplatelet agent, n (%)	6,211 (38.2)	5,134 (34.2)	4,971 (34.1)	5,681 (36.5)	5,489 (36.5)
Comorbidities (or medical history)‖, n (%)					
**Asthma**	**321 (2.0)**	**339 (2.3)**	**321 (2.2)**	**353 (2.3)**	**383 (2.5)**
Prior 4 years	165 (1.0)	189 (1.3)	197 (1.4)	193 (1.2)	225 (1.5)
Index hospitalization	181 (1.1)	212 (1.4)	175 (1.2)	215 (1.4)	209 (1.4)
**Chronic obstructive pulmonary disease**	**2,287 (14.1)**	**2,037 (13.6)**	**1,883 (12.9)**	**1,971 (12.7)**	**1,885 (12.5)**
Prior 4 years	1,178 (7.3)	1,167 (7.8)	1,086 (7.5)	1,230 (7.9)	1,105 (7.4)
Index hospitalization	1,787 (11.0)	1,640 (10.9)	1,471 (10.1)	1,478 (9.5)	1,432 (9.5)
**Other chronic lung diseases**	**590 (3.6)**	**560 (3.7)**	**537 (3.7)**	**591 (3.8)**	**635 (4.2)**
Prior 4 years	262 (1.6)	240 (1.6)	210 (1.4)	223 (1.4)	228 (1.5)

© 2023 Chen Q et al. *JAMA Network Open.*

Index hospitalization	361 (2.2)	373 (2.5)	372 (2.6)	416 (2.7)	463 (3.1)
**Heart failure**	**2,834 (17.4)**	**2,694 (17.9)**	**2,592 (17.8)**	**2,655 (17.1)**	**2,614 (17.4)**
Prior 4 years	807 (5.0)	820 (5.5)	788 (5.4)	803 (5.2)	816 (5.4)
Index hospitalization	2,385 (14.7)	2,262 (15.1)	2,180 (15.0)	2,229 (14.3)	2,177 (14.5)
**Myocardial infarction (history)**	**2,231 (13.7)**	**2,029 (13.5)**	**2,073 (14.2)**	**2,288 (14.7)**	**2,222 (14.8)**
Prior 4 years	1,491 (9.2)	1,352 (9.0)	1,375 (9.4)	1,580 (10.2)	1,528 (10.2)
Index hospitalization	907 (5.6)	866 (5.8)	918 (6.3)	956 (6.1)	953 (6.3)
**Hypertension**	**5,430 (33.4)**	**5,025 (33.4)**	**4,782 (32.8)**	**5,344 (34.4)**	**5,227 (34.8)**
Prior 4 years	2,350 (14.5)	2,440 (16.2)	2,498 (17.2)	2,805 (18.0)	2,809 (18.7)
Index hospitalization	4,183 (25.7)	3,924 (26.1)	3,576 (24.6)	3,862 (24.8)	3,768 (25.1)
**Other valvular heart disease[#]**	**1,737 (10.7)**	**1,659 (11.0)**	**1,543 (10.6)**	**1,734 (11.1)**	**1,643 (10.9)**
Prior 4 years	693 (4.3)	738 (4.9)	725 (5.0)	866 (5.6)	833 (5.5)
Index hospitalization	1,389 (8.5)	1,360 (9.1)	1,227 (8.4)	1,302 (8.4)	1,218 (8.1)
**Peripheral artery disease**	**519 (3.2)**	**547 (3.6)**	**458 (3.1)**	**486 (3.1)**	**458 (3.0)**
Prior 4 years	271 (1.7)	296 (2.0)	285 (2.0)	294 (1.9)	273 (1.8)
Index hospitalization	311 (1.9)	345 (2.3)	255 (1.8)	270 (1.7)	245 (1.6)
**Liver diseases**	**639 (3.9)**	**582 (3.9)**	**639 (4.4)**	**618 (4.0)**	**595 (4.0)**
Prior 4 years	282 (1.7)	253 (1.7)	268 (1.8)	278 (1.8)	283 (1.9)
Index hospitalization	444 (2.7)	407 (2.7)	452 (3.1)	423 (2.7)	415 (2.8)
**Gastroesophageal reflux disease**	**83 (0.5)**	**108 (0.7)**	**119 (0.8)**	**141 (0.9)**	**118 (0.8)**
Prior 4 years	46 (0.3)	76 (0.5)	81 (0.6)	103 (0.7)	88 (0.6)
Index hospitalization	41 (0.3)	33 (0.2)	42 (0.3)	42 (0.3)	32 (0.2)
**Peptic ulcer disease**	**189 (1.2)**	**181 (1.2)**	**179 (1.2)**	**153 (1.0)**	**162 (1.1)**
Prior 4 years	98 (0.6)	80 (0.5)	76 (0.5)	81 (0.5)	70 (0.5)
Index hospitalization	97 (0.6)	103 (0.7)	105 (0.7)	80 (0.5)	97 (0.6)
**Chronic kidney diseases**	**2,374 (14.6)**	**2,416 (16.1)**	**2,354 (16.2)**	**2,437 (15.7)**	**2,338 (15.6)**
Prior 4 years	984 (6.1)	1,089 (7.2)	1,080 (7.4)	1,163 (7.5)	1,162 (7.7)
Index hospitalization	1,847 (11.4)	1,877 (12.5)	1,825 (12.5)	1,864 (12.0)	1,801 (12.0)
**Anaemia**	**2,201 (13.5)**	**2,343 (15.6)**	**2,282 (15.7)**	**2,319 (14.9)**	**2,386 (15.9)**
Prior 4 years	1,246 (7.7)	1,331 (8.9)	1,251 (8.6)	1,267 (8.1)	1,313 (8.7)
Index hospitalization	1,228 (7.6)	1,340 (8.9)	1,352 (9.3)	1,380 (8.9)	1,440 (9.6)
**Coagulopathy**	**375 (2.3)**	**450 (3.0)**	**495 (3.4)**	**518 (3.3)**	**553 (3.7)**
Prior 4 years	130 (0.8)	169 (1.1)	193 (1.3)	200 (1.3)	210 (1.4)
Index hospitalization	269 (1.7)	303 (2.0)	335 (2.3)	355 (2.3)	373 (2.5)
**Diabetes**	**3,183 (19.6)**	**2,856 (19.0)**	**2,840 (19.5)**	**3,040 (19.5)**	**2,890 (19.2)**
Prior 4 years	1,664 (10.2)	1,629 (10.8)	1,662 (11.4)	1,876 (12.1)	1,771 (11.8)
Index hospitalization	2,493 (15.3)	2,355 (15.7)	2,336 (16.0)	2,384 (15.3)	2,280 (15.2)

© 2023 Chen Q et al. *JAMA Network Open.*

**Thyroid disease**	**549 (3.4)**	**481 (3.2)**	**473 (3.2)**	**555 (3.6)**	**548 (3.6)**
Prior 4 years	192 (1.2)	178 (1.2)	209 (1.4)	255 (1.6)	244 (1.6)
Index hospitalization	396 (2.4)	352 (2.3)	307 (2.1)	365 (2.3)	370 (2.5)
**Ischemic stroke (history)**	**854 (5.3)**	**774 (5.2)**	**748 (5.1)**	**736 (4.7)**	**705 (4.7)**
Prior 4 years	477 (2.9)	478 (3.2)	479 (3.3)	480 (3.1)	460 (3.1)
Index hospitalization	476 (2.9)	409 (2.7)	364 (2.5)	339 (2.2)	353 (2.3)
**TIA**	**300 (1.8)**	**265 (1.8)**	**228 (1.6)**	**245 (1.6)**	**227 (1.5)**
Prior 4 years	210 (1.3)	191 (1.3)	159 (1.1)	180 (1.2)	173 (1.2)
Index hospitalization	97 (0.6)	86 (0.6)	73 (0.5)	74 (0.5)	59 (0.4)
**Other arterial thromboembolism**	**240 (1.5)**	**227 (1.5)**	**203 (1.4)**	**202 (1.3)**	**171 (1.1)**
Prior 4 years	174 (1.1)	153 (1.0)	138 (0.9)	137 (0.9)	107 (0.7)
Index hospitalization	76 (0.5)	88 (0.6)	75 (0.5)	73 (0.5)	67 (0.4)
**Parkinson's disease**	**240 (1.5)**	**198 (1.3)**	**190 (1.3)**	**219 (1.4)**	**188 (1.3)**
Prior 4 years	107 (0.7)	106 (0.7)	94 (0.6)	128 (0.8)	101 (0.7)
Index hospitalization	188 (1.2)	154 (1.0)	158 (1.1)	175 (1.1)	150 (1.0)
**Alzheimer's disease**	**304 (1.9)**	**299 (2.0)**	**294 (2.0)**	**298 (1.9)**	**274 (1.8)**
Prior 4 years	104 (0.6)	83 (0.6)	72 (0.5)	89 (0.6)	71 (0.5)
Index hospitalization	252 (1.6)	269 (1.8)	267 (1.8)	268 (1.7)	259 (1.7)
**Autoimmune disease**	**110 (0.7)**	**82 (0.5)**	**106 (0.7)**	**131 (0.8)**	**127 (0.8)**
Prior 4 years	76 (0.5)	53 (0.4)	82 (0.6)	95 (0.6)	99 (0.7)
Index hospitalization	43 (0.3)	37 (0.2)	35 (0.2)	51 (0.3)	44 (0.3)
**Systemic connective tissue disorders**	**216 (1.3)**	**261 (1.7)**	**223 (1.5)**	**247 (1.6)**	**235 (1.6)**
Prior 4 years	108 (0.7)	129 (0.9)	137 (0.9)	159 (1.0)	130 (0.9)
Index hospitalization	141 (0.9)	193 (1.3)	146 (1.0)	139 (0.9)	159 (1.1)
**Venous thromboembolism**	**377 (2.3)**	**318 (2.1)**	**392 (2.7)**	**396 (2.5)**	**318 (2.1)**
Prior 4 years to prior 6 months	88 (0.5)	81 (0.5)	121 (0.8)	102 (0.7)	76 (0.5)
Prior 6 months	294 (1.8)	243 (1.6)	280 (1.9)	301 (1.9)	249 (1.7)
**Deep vein thrombosis**	**165 (1.0)**	**128 (0.9)**	**169 (1.2)**	**162 (1.0)**	**146 (1.0)**
Prior 4 years to prior 6 months	44 (0.3)	42 (0.3)	55 (0.4)	52 (0.3)	38 (0.3)
Prior 6 months	123 (0.8)	87 (0.6)	115 (0.8)	114 (0.7)	108 (0.7)
**Pulmonary embolism**	**237 (1.5)**	**203 (1.4)**	**259 (1.8)**	**261 (1.7)**	**193 (1.3)**
Prior 4 years to prior 6 months	52 (0.3)	43 (0.3)	76 (0.5)	60 (0.4)	42 (0.3)
Prior 6 months	188 (1.2)	163 (1.1)	189 (1.3)	203 (1.3)	157 (1.0)
**Major bleeding**	**1,509 (9.3)**	**1,537 (10.2)**	**1,552 (10.7)**	**1,535 (9.9)**	**1,539 (10.2)**
Prior 4 years	763 (4.7)	747 (5.0)	789 (5.4)	796 (5.1)	759 (5.1)
Index hospitalization	849 (5.2)	897 (6.0)	871 (6.0)	853 (5.5)	895 (6.0)
**Intracranial haemorrhage**	**320 (2.0)**	**311 (2.1)**	**273 (1.9)**	**306 (2.0)**	**236 (1.6)**

© 2023 Chen Q et al. *JAMA Network Open.*

Prior 4 years	110 (0.7)	118 (0.8)	86 (0.6)	108 (0.7)	85 (0.6)
Index hospitalization	226 (1.4)	215 (1.4)	200 (1.4)	218 (1.4)	169 (1.1)
**Gastrointestinal bleeding**	**633 (3.9)**	**661 (4.4)**	**667 (4.6)**	**628 (4.0)**	**678 (4.5)**
Prior 4 years	379 (2.3)	362 (2.4)	373 (2.6)	377 (2.4)	364 (2.4)
Index hospitalization	287 (1.8)	330 (2.2)	326 (2.2)	287 (1.8)	346 (2.3)
**Malignant tumour**	**3,203 (19.7)**	**2,876 (19.1)**	**2,877 (19.8)**	**2,935 (18.9)**	**2,838 (18.9)**
Prior 4 years	2,341 (14.4)	2,117 (14.1)	2,096 (14.4)	2,220 (14.3)	2,141 (14.2)
Index hospitalization	2,108 (13.0)	2,021 (13.4)	2,047 (14.1)	2,006 (12.9)	1,998 (13.3)
≥1 hospitalization within the prior 4 years, n (%)	11,120 (68.4)	9,968 (66.3)	9,618 (66.0)	10,434 (67.1)	9,841 (65.5)
Information about the index hospitalization					
Length of hospital stay, days, median (IQR)	4 (1-9)	4 (1-10)	4 (1-10)	3 (0-9)	3 (0-9)
In-hospital mortality, n (%)	1,835 (11.3)	2,014 (13.4)	2,018 (13.9)	2,105 (13.5)	2,168 (14.4)
**Main reason for admission to the index hospitalization**					
Atrial fibrillation	4,567 (28.1)	4,033 (26.8)	3,988 (27.4)	4,931 (31.7)	4,586 (30.5)
Heart failure	846 (5.2)	778 (5.2)	770 (5.3)	761 (4.9)	738 (4.9)
Ischemic heart diseases	1,568 (9.7)	1,350 (9.0)	1,355 (9.3)	1,426 (9.2)	1,497 (10.0)
Respiratory diseases	1,304 (8.0)	1,596 (10.6)	1,495 (10.3)	1,569 (10.1)	1,569 (10.4)
Injury/poisoning	1,157 (7.1)	1,106 (7.4)	1,085 (7.5)	1,019 (6.6)	1,130 (7.5)
Symptoms/signs/abnormal laboratory findings[**]	816 (5.0)	698 (4.6)	620 (4.3)	524 (3.4)	448 (3.0)
Neoplasms	1,361 (8.4)	1,176 (7.8)	1,189 (8.2)	1,217 (7.8)	1,175 (7.8)
Digestive diseases	946 (5.8)	822 (5.5)	833 (5.7)	814 (5.2)	759 (5.1)
Other heart valve disorders[§§]	279 (1.7)	308 (2.0)	312 (2.1)	332 (2.1)	272 (1.8)
Genitourinary diseases	505 (3.1)	537 (3.6)	508 (3.5)	512 (3.3)	486 (3.2)
Infectious/parasitic diseases	458 (2.8)	449 (3.0)	405 (2.8)	428 (2.8)	424 (2.8)
Endocrine/nutritional/metabolic diseases	237 (1.5)	222 (1.5)	190 (1.3)	176 (1.1)	211 (1.4)
Diseases of blood/blood-forming organs	174 (1.1)	187 (1.2)	145 (1.0)	156 (1.0)	143 (1.0)
Neurological disorders[##]	184 (1.1)	157 (1.0)	144 (1.0)	142 (0.9)	135 (0.9)

† First-generation immigrants refer to persons who were born abroad with at least one parent who was born abroad; second-generation immigrants refer to persons who was born in the Netherlands with at least one parent who was born abroad.

‡ Percentile groups were determined based on disposable income of private households of the complete target population in the database (instead of the study population only).

* Labile INR (international normalized ratio) was not included for calculation.

§ Pre-existing chronic use was defined as ≥2 (outpatient) prescription records of the same type of antithrombotic agents within six months (*i.e.*, 183 days) before the index dates (exclusive). Pre-existing chronic use of oral anticoagulant refers to persons with pre-existing chronic use of vitamin K antagonist or direct oral anticoagulant.

|| Unless otherwise stated, for each comorbidity (or medical history), prevalence calculated by screening all hospitalizations within the prior four years (including the index hospitalization), all hospitalizations within the prior four years (excluding the index hospitalization), and only the index hospitalization is presented, respectively.

Except for rheumatic mitral stenosis and mechanical heart valves

** Except for abnormalities of heart beat (ICD-10 code: R00) and cardiac murmurs and other cardiac sounds (ICD-10 code: R01)

§§ Except for endocarditis, valve unspecified, in diseases classified elsewhere (ICD-10 code: I398)

Except for transient cerebral ischaemic attacks and related syndromes (ICD-10 code: G45) and vascular syndromes of brain in cerebrovascular diseases (ICD-10 code: G46) Abbreviations: SD, standard deviation; TIA, transient ischemic attack; IQR, interquartile range; ICD, International Classification of Diseases.

© 2023 Chen Q et al. *JAMA Network Open.*

eTable 12. Time Trends in Patient Characteristics of Incident Nonvalvular Atrial Fibrillation Among Patients Who Received at Least 1 Oral Anticoagulant Within the 1-Year Follow-up, Excluding Patients With Preexisting Chronic Oral Anticoagulant Treatment

Cohort	2014 (N=24,415)	2015 (N=29,122)	2016 (N=28,791)	2017 (N=28,860)	2018 (N=27,558)
Age, years, mean ± SD	75.0 ± 11.0	74.7 ± 11.1	74.5 ± 11.1	74.7 ± 11.1	74.5 ± 11.2
Age group, years, n (%)					
18-24	11 (0.0)	13 (0.0)	15 (0.1)	19 (0.1)	20 (0.1)
25-34	48 (0.2)	58 (0.2)	66 (0.2)	58 (0.2)	85 (0.3)
35-44	191 (0.8)	272 (0.9)	229 (0.8)	233 (0.8)	235 (0.9)
45-54	937 (3.8)	1,181 (4.1)	1,178 (4.1)	1,160 (4.0)	1,132 (4.1)
55-64	2,976 (12.2)	3,651 (12.5)	3,687 (12.8)	3,687 (12.8)	3,514 (12.8)
65-74	7,093 (29.1)	8,619 (29.6)	8,723 (30.3)	8,727 (30.2)	8,528 (30.9)
75-84	8,509 (34.9)	10,073 (34.6)	9,714 (33.7)	9,693 (33.6)	9,144 (33.2)
≥85	4,650 (19.0)	5,255 (18.0)	5,179 (18.0)	5,283 (18.3)	4,900 (17.8)
Sex, n (%)					
Male	13,200 (54.1)	15,419 (52.9)	15,439 (53.6)	15,663 (54.3)	15,123 (54.9)
Female	11,215 (45.9)	13,703 (47.1)	13,352 (46.4)	13,197 (45.7)	12,435 (45.1)
Immigration background[†], n (%)					
Native Dutch	21,484 (88.0)	25,490 (87.5)	25,111 (87.2)	25,198 (87.3)	24,016 (87.1)
First-generation immigrants	1,703 (7.0)	2,052 (7.0)	2,029 (7.0)	2,040 (7.1)	2,082 (7.6)
Second-generation immigrants	1,228 (5.0)	1,580 (5.4)	1,651 (5.7)	1,622 (5.6)	1,460 (5.3)
Standardized household income[‡], n (%)					
First quintile (0-20%)	3,978 (16.3)	4,642 (16.0)	4,917 (17.1)	4,913 (17.1)	4,774 (17.4)
Second quintile (20-40%)	7,208 (29.6)	8,797 (30.3)	8,590 (29.9)	8,704 (30.2)	8,267 (30.1)
Third quintile (40-60%)	5,917 (24.3)	6,932 (23.9)	6,761 (23.5)	6,945 (24.1)	6,730 (24.5)
Fourth quintile (60-80%)	3,686 (15.1)	4,523 (15.6)	4,467 (15.5)	4,322 (15.0)	4,117 (15.0)
Fifth quintile (80-100%)	2,770 (11.4)	3,366 (11.6)	3,318 (11.5)	3,319 (11.5)	3,042 (11.1)
Private household with an unknown income	[masked][‖]	23 (0.1)	17 (0.1)	16 (0.1)	10 (0.0)
Institutional household	[masked][‖]	781 (2.7)	670 (2.3)	572 (2.0)	548 (2.0)
Marital status, n (%)					
Married or in partnership	13,565 (55.6)	16,540 (56.8)	16,265 (56.5)	16,186 (56.1)	15,429 (56.0)
Unmarried or single	1,570 (6.4)	1,897 (6.5)	1,958 (6.8)	2,122 (7.4)	2,101 (7.6)
Divorced	2,353 (9.6)	2,854 (9.8)	3,005 (10.4)	3,044 (10.5)	3,073 (11.2)
Widowed	6,927 (28.4)	7,831 (26.9)	7,563 (26.3)	7,508 (26.0)	6,955 (25.2)
Type of atrial fibrillation, n (%)					
Paroxysmal atrial fibrillation (I48.0)	-	4,721 (16.2)	4,749 (16.5)	4,600 (15.9)	4,266 (15.5)
Persistent atrial fibrillation (I48.1)	-	672 (2.3)	696 (2.4)	628 (2.2)	577 (2.1)

© 2023 Chen Q et al. *JAMA Network Open.*

Chronic atrial fibrillation (I48.2)	-	1,082 (3.7)	1,074 (3.7)	1,043 (3.6)	906 (3.3)
Type I atrial flutter (I48.3)	-	185 (0.6)	241 (0.8)	292 (1.0)	316 (1.1)
Type II atrial flutter (I48.4)	-	96 (0.3)	69 (0.2)	75 (0.3)	69 (0.3)
Unspecified atrial fibrillation (I48.9)	-	22,366 (76.8)	21,962 (76.3)	22,222 (77.0)	21,424 (77.7)
Diagnosis made before 2015	24,415 (100.0)	-	-	-	-
CHA_2DS_2-VASc score, mean ± SD	3.05 ± 1.61	3.01 ± 1.61	3.00 ± 1.61	3.02 ± 1.60	3.01 ± 1.59
CHA_2DS_2-VASc score, n (%)					
0	1,259 (5.2)	1,641 (5.6)	1,644 (5.7)	1,543 (5.3)	1,463 (5.3)
1	2,910 (11.9)	3,554 (12.2)	3,519 (12.2)	3,506 (12.1)	3,415 (12.4)
**≥2**	**20,246 (82.9)**	**23,927 (82.2)**	**23,628 (82.1)**	**23,811 (82.5)**	**22,680 (82.3)**
2	5,034 (20.6)	6,069 (20.8)	6,024 (20.9)	5,956 (20.6)	5,639 (20.5)
3	5,961 (24.4)	7,066 (24.3)	7,054 (24.5)	7,167 (24.8)	6,851 (24.9)
4	4,894 (20.0)	5,724 (19.7)	5,600 (19.5)	5,727 (19.8)	5,480 (19.9)
5	2,740 (11.2)	3,224 (11.1)	3,093 (10.7)	3,111 (10.8)	2,955 (10.7)
6	1,126 (4.6)	1,276 (4.4)	1,299 (4.5)	1,276 (4.4)	1,264 (4.6)
≥7	491 (2.0)	568 (2.0)	558 (1.9)	574 (2.0)	491 (1.8)
HAS-BLED score*, mean ± SD	1.82 ± 1.01	1.76 ± 1.00	1.75 ± 1.00	1.74 ± 1.00	1.73 ± 1.01
HAS-BLED score*, n (%)					
0	1,884 (7.7)	2,503 (8.6)	2,537 (8.8)	2,570 (8.9)	2,530 (9.2)
1	7,859 (32.2)	9,724 (33.4)	9,765 (33.9)	9,949 (34.5)	9,586 (34.8)
2	8,890 (36.4)	10,575 (36.3)	10,329 (35.9)	10,180 (35.3)	9,611 (34.9)
≥3	5,782 (23.7)	6,320 (21.7)	6,160 (21.4)	6,161 (21.3)	5,831 (21.2)
Pre-existing chronic use of antithrombotic agents§					
**Oral anticoagulant, n (%)**	-	-	-	-	-
Vitamin K antagonist, n (%)	-	-	-	-	-
Direct oral anticoagulant, n (%)	-	-	-	-	-
Heparin group, n (%)	277 (1.1)	300 (1.0)	276 (1.0)	244 (0.8)	230 (0.8)
Antiplatelet agent, n (%)	8,332 (34.1)	9,511 (32.7)	9,294 (32.3)	9,395 (32.6)	9,071 (32.9)
Comorbidities (or medical history)‖, n (%)					
**Asthma**	**477 (2.0)**	**620 (2.1)**	**680 (2.4)**	**684 (2.4)**	**775 (2.8)**
Prior 4 years	215 (0.9)	305 (1.0)	353 (1.2)	331 (1.1)	407 (1.5)
Index hospitalization	336 (1.4)	408 (1.4)	437 (1.5)	470 (1.6)	547 (2.0)
**Chronic obstructive pulmonary disease**	**3,017 (12.4)**	**3,430 (11.8)**	**3,425 (11.9)**	**3,366 (11.7)**	**3,243 (11.8)**
Prior 4 years	1,397 (5.7)	1,678 (5.8)	1,788 (6.2)	1,747 (6.1)	1,693 (6.1)
Index hospitalization	2,537 (10.4)	2,838 (9.7)	2,800 (9.7)	2,752 (9.5)	2,701 (9.8)
**Other chronic lung diseases**	**492 (2.0)**	**468 (1.6)**	**518 (1.8)**	**599 (2.1)**	**632 (2.3)**
Prior 4 years	288 (1.2)	238 (0.8)	273 (0.9)	287 (1.0)	300 (1.1)

© 2023 Chen Q et al. *JAMA Network Open.*

Index hospitalization	233 (1.0)	271 (0.9)	294 (1.0)	387 (1.3)	414 (1.5)
**Heart failure**	**5,667 (23.2)**	**6,512 (22.4)**	**6,353 (22.1)**	**6,435 (22.3)**	**6,372 (23.1)**
Prior 4 years	1,242 (5.1)	1,381 (4.7)	1,459 (5.1)	1,409 (4.9)	1,275 (4.6)
Index hospitalization	4,995 (20.5)	5,764 (19.8)	5,635 (19.6)	5,684 (19.7)	5,744 (20.8)
**Myocardial infarction (history)**	**3,010 (12.3)**	**3,591 (12.3)**	**3,698 (12.8)**	**3,818 (13.2)**	**3,629 (13.2)**
Prior 4 years	1,881 (7.7)	2,194 (7.5)	2,348 (8.2)	2,396 (8.3)	2,199 (8.0)
Index hospitalization	1,343 (5.5)	1,688 (5.8)	1,683 (5.8)	1,824 (6.3)	1,830 (6.6)
**Hypertension**	**9,050 (37.1)**	**11,041 (37.9)**	**11,058 (38.4)**	**11,420 (39.6)**	**11,181 (40.6)**
Prior 4 years	3,201 (13.1)	4,319 (14.8)	4,520 (15.7)	4,753 (16.5)	4,664 (16.9)
Index hospitalization	7,622 (31.2)	9,146 (31.4)	9,097 (31.6)	9,384 (32.5)	9,344 (33.9)
**Other valvular heart disease[#]**	**3,993 (16.4)**	**4,374 (15.0)**	**4,208 (14.6)**	**4,275 (14.8)**	**4,075 (14.8)**
Prior 4 years	1,523 (6.2)	1,727 (5.9)	1,783 (6.2)	1,780 (6.2)	1,712 (6.2)
Index hospitalization	3,375 (13.8)	3,745 (12.9)	3,583 (12.4)	3,619 (12.5)	3,469 (12.6)
**Peripheral artery disease**	**652 (2.7)**	**855 (2.9)**	**810 (2.8)**	**819 (2.8)**	**803 (2.9)**
Prior 4 years	325 (1.3)	443 (1.5)	467 (1.6)	493 (1.7)	464 (1.7)
Index hospitalization	425 (1.7)	553 (1.9)	494 (1.7)	460 (1.6)	465 (1.7)
**Liver diseases**	**576 (2.4)**	**669 (2.3)**	**669 (2.3)**	**684 (2.4)**	**664 (2.4)**
Prior 4 years	218 (0.9)	271 (0.9)	317 (1.1)	312 (1.1)	326 (1.2)
Index hospitalization	400 (1.6)	439 (1.5)	416 (1.4)	438 (1.5)	405 (1.5)
**Gastroesophageal reflux disease**	**116 (0.5)**	**129 (0.4)**	**151 (0.5)**	**171 (0.6)**	**169 (0.6)**
Prior 4 years	69 (0.3)	93 (0.3)	99 (0.3)	124 (0.4)	121 (0.4)
Index hospitalization	49 (0.2)	39 (0.1)	54 (0.2)	52 (0.2)	52 (0.2)
**Peptic ulcer disease**	**175 (0.7)**	**169 (0.6)**	**169 (0.6)**	**184 (0.6)**	**167 (0.6)**
Prior 4 years	87 (0.4)	80 (0.3)	100 (0.3)	98 (0.3)	85 (0.3)
Index hospitalization	91 (0.4)	94 (0.3)	72 (0.3)	91 (0.3)	88 (0.3)
**Chronic kidney diseases**	**3,063 (12.5)**	**3,609 (12.4)**	**3,898 (13.5)**	**4,041 (14.0)**	**3,697 (13.4)**
Prior 4 years	1,239 (5.1)	1,476 (5.1)	1,644 (5.7)	1707 (5.9)	1,651 (6.0)
Index hospitalization	2,417 (9.9)	2,871 (9.9)	3,071 (10.7)	3,193 (11.1)	2,970 (10.8)
**Anaemia**	**2,414 (9.9)**	**2,763 (9.5)**	**2,983 (10.4)**	**3,191 (11.1)**	**3,180 (11.5)**
Prior 4 years	1,249 (5.1)	1,439 (4.9)	1,533 (5.3)	1,610 (5.6)	1,613 (5.9)
Index hospitalization	1,403 (5.7)	1,597 (5.5)	1,747 (6.1)	1,936 (6.7)	1,918 (7.0)
**Coagulopathy**	**353 (1.4)**	**494 (1.7)**	**528 (1.8)**	**573 (2.0)**	**580 (2.1)**
Prior 4 years	103 (0.4)	171 (0.6)	203 (0.7)	223 (0.8)	212 (0.8)
Index hospitalization	260 (1.1)	339 (1.2)	351 (1.2)	365 (1.3)	396 (1.4)
**Diabetes**	**4,703 (19.3)**	**5,518 (18.9)**	**5,616 (19.5)**	**5,640 (19.5)**	**5,445 (19.8)**
Prior 4 years	2,155 (8.8)	2,684 (9.2)	2,825 (9.8)	2,898 (10.0)	2,721 (9.9)
Index hospitalization	4,067 (16.7)	4,770 (16.4)	4,902 (17.0)	4,938 (17.1)	4,920 (17.9)

© 2023 Chen Q et al. *JAMA Network Open.*

**Thyroid disease**	**871 (3.6)**	**987 (3.4)**	**1,036 (3.6)**	**1,091 (3.8)**	**984 (3.6)**
Prior 4 years	248 (1.0)	282 (1.0)	340 (1.2)	344 (1.2)	297 (1.1)
Index hospitalization	683 (2.8)	788 (2.7)	782 (2.7)	854 (3.0)	797 (2.9)
**Ischemic stroke (history)**	**1,017 (4.2)**	**1,133 (3.9)**	**1,083 (3.8)**	**1,152 (4.0)**	**1,021 (3.7)**
Prior 4 years	683 (2.8)	804 (2.8)	775 (2.7)	860 (3.0)	716 (2.6)
Index hospitalization	465 (1.9)	445 (1.5)	408 (1.4)	423 (1.5)	437 (1.6)
**TIA**	**793 (3.2)**	**819 (2.8)**	**848 (2.9)**	**822 (2.8)**	**719 (2.6)**
Prior 4 years	387 (1.6)	405 (1.4)	409 (1.4)	456 (1.6)	359 (1.3)
Index hospitalization	427 (1.7)	431 (1.5)	472 (1.6)	386 (1.3)	379 (1.4)
**Other arterial thromboembolism**	**331 (1.4)**	**342 (1.2)**	**320 (1.1)**	**325 (1.1)**	**260 (0.9)**
Prior 4 years	239 (1.0)	238 (0.8)	218 (0.8)	197 (0.7)	158 (0.6)
Index hospitalization	110 (0.5)	126 (0.4)	115 (0.4)	140 (0.5)	107 (0.4)
**Parkinson's disease**	**168 (0.7)**	**283 (1.0)**	**277 (1.0)**	**273 (0.9)**	**263 (1.0)**
Prior 4 years	79 (0.3)	128 (0.4)	130 (0.5)	129 (0.4)	116 (0.4)
Index hospitalization	133 (0.5)	229 (0.8)	224 (0.8)	224 (0.8)	225 (0.8)
**Alzheimer's disease**	**177 (0.7)**	**206 (0.7)**	**215 (0.7)**	**199 (0.7)**	**201 (0.7)**
Prior 4 years	56 (0.2)	71 (0.2)	74 (0.3)	59 (0.2)	46 (0.2)
Index hospitalization	148 (0.6)	168 (0.6)	185 (0.6)	173 (0.6)	182 (0.7)
**Autoimmune disease**	**79 (0.3)**	**94 (0.3)**	**124 (0.4)**	**135 (0.5)**	**143 (0.5)**
Prior 4 years	47 (0.2)	62 (0.2)	95 (0.3)	93 (0.3)	104 (0.4)
Index hospitalization	42 (0.2)	44 (0.2)	48 (0.2)	62 (0.2)	61 (0.2)
**Systemic connective tissue disorders**	**300 (1.2)**	**385 (1.3)**	**400 (1.4)**	**398 (1.4)**	**410 (1.5)**
Prior 4 years	122 (0.5)	172 (0.6)	223 (0.8)	225 (0.8)	201 (0.7)
Index hospitalization	215 (0.9)	269 (0.9)	257 (0.9)	251 (0.9)	292 (1.1)
**Venous thromboembolism**	**547 (2.2)**	**612 (2.1)**	**616 (2.1)**	**692 (2.4)**	**642 (2.3)**
Prior 4 years to prior 6 months	131 (0.5)	166 (0.6)	177 (0.6)	155 (0.5)	127 (0.5)
Prior 6 months	428 (1.8)	464 (1.6)	456 (1.6)	550 (1.9)	519 (1.9)
**Deep vein thrombosis**	**207 (0.8)**	**241 (0.8)**	**226 (0.8)**	**280 (1.0)**	**217 (0.8)**
Prior 4 years to prior 6 months	52 (0.2)	72 (0.2)	68 (0.2)	74 (0.3)	51 (0.2)
Prior 6 months	157 (0.6)	174 (0.6)	160 (0.6)	209 (0.7)	166 (0.6)
**Pulmonary embolism**	**408 (1.7)**	**434 (1.5)**	**463 (1.6)**	**474 (1.6)**	**475 (1.7)**
Prior 4 years to prior 6 months	94 (0.4)	110 (0.4)	126 (0.4)	103 (0.4)	89 (0.3)
Prior 6 months	323 (1.3)	334 (1.1)	349 (1.2)	378 (1.3)	389 (1.4)
**Major bleeding**	**1,414 (5.8)**	**1,642 (5.6)**	**1,537 (5.3)**	**1,763 (6.1)**	**1,649 (6.0)**
Prior 4 years	859 (3.5)	968 (3.3)	936 (3.3)	1,025 (3.6)	937 (3.4)
Index hospitalization	628 (2.6)	761 (2.6)	677 (2.4)	825 (2.9)	785 (2.8)
**Intracranial haemorrhage**	**151 (0.6)**	**176 (0.6)**	**136 (0.5)**	**151 (0.5)**	**138 (0.5)**

© 2023 Chen Q et al. *JAMA Network Open.*

Prior 4 years	82 (0.3)	91 (0.3)	73 (0.3)	88 (0.3)	75 (0.3)
Index hospitalization	73 (0.3)	94 (0.3)	68 (0.2)	73 (0.3)	72 (0.3)
**Gastrointestinal bleeding**	**666 (2.7)**	**701 (2.4)**	**678 (2.4)**	**768 (2.7)**	**690 (2.5)**
Prior 4 years	422 (1.7)	457 (1.6)	471 (1.6)	476 (1.6)	428 (1.6)
Index hospitalization	264 (1.1)	270 (0.9)	235 (0.8)	326 (1.1)	287 (1.0)
**Malignant tumour**	**2,786 (11.4)**	**3,216 (11.0)**	**3,288 (11.4)**	**3,421 (11.9)**	**3,340 (12.1)**
Prior 4 years	2,193 (9.0)	2,544 (8.7)	2,586 (9.0)	2,723 (9.4)	2,624 (9.5)
Index hospitalization	1,378 (5.6)	1,648 (5.7)	1,720 (6.0)	1,788 (6.2)	1,856 (6.7)
≥1 hospitalization within the prior 4 years, n (%)	15,523 (63.6)	18,091 (62.1)	17,769 (61.7)	17,749 (61.5)	16,461 (59.7)
Information about the index hospitalization					
Length of hospital stay, days, median (IQR)	4 (1-8)	3 (1-8)	3 (1-8)	3 (1-8)	4 (1-8)
In-hospital mortality, n (%)	13 (0.1)	19 (0.1)	11 (0.0)	17 (0.1)	19 (0.1)
**Main reason for admission to the index hospitalization**					
Atrial fibrillation	8,720 (35.7)	11,603 (39.8)	11,478 (39.9)	10,913 (37.8)	9,926 (36.0)
Heart failure	2,414 (9.9)	2,818 (9.7)	2,751 (9.6)	2,661 (9.2)	2,650 (9.6)
Ischemic heart diseases	2,222 (9.1)	2,475 (8.5)	2,399 (8.3)	2,480 (8.6)	2,484 (9.0)
Respiratory diseases	1,730 (7.1)	2,178 (7.5)	2,162 (7.5)	2,298 (8.0)	2,410 (8.7)
Injury/poisoning	919 (3.8)	1,042 (3.6)	1,065 (3.7)	1,176 (4.1)	1,180 (4.3)
Symptoms/signs/abnormal laboratory findings**	1,207 (4.9)	1,357 (4.7)	1,285 (4.5)	1,239 (4.3)	1,141 (4.1)
Neoplasms	802 (3.3)	944 (3.2)	964 (3.3)	1,001 (3.5)	1,028 (3.7)
Digestive diseases	903 (3.7)	1,032 (3.5)	907 (3.2)	1,052 (3.6)	969 (3.5)
Other heart valve disorders§§	879 (3.6)	880 (3.0)	921 (3.2)	934 (3.2)	867 (3.1)
Genitourinary diseases	598 (2.4)	624 (2.1)	672 (2.3)	747 (2.6)	768 (2.8)
Infectious/parasitic diseases	363 (1.5)	394 (1.4)	433 (1.5)	498 (1.7)	477 (1.7)
Endocrine/nutritional/metabolic diseases	251 (1.0)	269 (0.9)	274 (1.0)	267 (0.9)	327 (1.2)
Diseases of blood/blood-forming organs	139 (0.6)	123 (0.4)	143 (0.5)	176 (0.6)	147 (0.5)
Neurological disorders##	167 (0.7)	169 (0.6)	135 (0.5)	185 (0.6)	138 (0.5)

† First-generation immigrants refer to persons who were born abroad with at least one parent who was born abroad; second-generation immigrants refer to persons who was born in the Netherlands with at least one parent who was born abroad.

‡ Percentile groups were determined based on disposable income of private households of the complete target population in the database (instead of the study population only).

* Labile INR (international normalized ratio) was not included for calculation.

§ Pre-existing chronic use was defined as ≥2 (outpatient) prescription records of the same type of antithrombotic agents within six months (*i.e.*, 183 days) before the index dates (exclusive). Pre-existing chronic use of oral anticoagulant refers to persons with pre-existing chronic use of vitamin K antagonist or direct oral anticoagulant.

|| Unless otherwise stated, for each comorbidity (or medical history), prevalence calculated by screening all hospitalizations within the prior four years (including the index hospitalization), all hospitalizations within the prior four years (excluding the index hospitalization), and only the index hospitalization is presented, respectively.

Except for rheumatic mitral stenosis and mechanical heart valves

** Except for abnormalities of heart beat (ICD-10 code: R00) and cardiac murmurs and other cardiac sounds (ICD-10 code: R01)

§§ Except for endocarditis, valve unspecified, in diseases classified elsewhere (ICD-10 code: I398)

© 2023 Chen Q et al. *JAMA Network Open.*

Except for transient cerebral ischaemic attacks and related syndromes (ICD-10 code: G45) and vascular syndromes of brain in cerebrovascular diseases (ICD-10 code: G46)
||| Exact result was masked to prevent potential individual or group disclosure.
Abbreviations: SD, standard deviation; TIA, transient ischemic attack; IQR, interquartile range; ICD, International Classification of Diseases.

© 2023 Chen Q et al. *JAMA Network Open.*

eTable 13. Time Trends in Patient Characteristics of Incident Nonvalvular Atrial Fibrillation Among Patients Who First Received Vitamin K Antagonist as Oral Anticoagulant Within the 1-Year Follow-up, Excluding Patients With Preexisting Chronic Oral Anticoagulant Treatment

Cohort	2014 (N=21,227)	2015 (N=20,209)	2016 (N=14,203)	2017 (N=9,518)	2018 (N=6,545)
Age, years, mean ± SD	75.7 ± 10.8	76.5 ± 10.4	76.9 ± 10.3	77.3 ± 10.5	76.9 ± 10.7
Age group, years, n (%)					
18-24	[masked]‖	[masked]‖	[masked]‖	[masked]‖	[masked]‖
25-34	[masked]‖	[masked]‖	[masked]‖	[masked]‖	[masked]‖
35-44	138 (0.7)	119 (0.6)	66 (0.5)	45 (0.5)	29 (0.4)
45-54	714 (3.4)	547 (2.7)	329 (2.3)	231 (2.4)	184 (2.8)
55-64	2,344 (11.0)	1,965 (9.7)	1,278 (9.0)	818 (8.6)	627 (9.6)
65-74	5,920 (27.9)	5,506 (27.2)	3,819 (26.9)	2,478 (26.0)	1,700 (26.0)
75-84	7,692 (36.2)	7,640 (37.8)	5,369 (37.8)	3,584 (37.7)	2,395 (36.6)
≥85	4,378 (20.6)	4,402 (21.8)	3,315 (23.3)	2,341 (24.6)	1,594 (24.4)
Sex, n (%)					
Male	11,402 (53.7)	10,608 (52.5)	7,527 (53.0)	5,240 (55.1)	3,696 (56.5)
Female	9,825 (46.3)	9,601 (47.5)	6,676 (47.0)	4,278 (44.9)	2,849 (43.5)
Immigration background†, n (%)					
Native Dutch	18,686 (88.0)	17,730 (87.7)	12,370 (87.1)	8,313 (87.3)	5,686 (86.9)
First-generation immigrants	1,497 (7.1)	1,435 (7.1)	1,043 (7.3)	683 (7.2)	506 (7.7)
Second-generation immigrants	1,044 (4.9)	1,044 (5.2)	790 (5.6)	522 (5.5)	353 (5.4)
Standardized household income‡, n (%)					
First quintile (0-20%)	3,574 (16.9)	3,472 (17.2)	2,711 (19.1)	1,837 (19.3)	1,219 (18.7)
Second quintile (20-40%)	6,442 (30.4)	6,505 (32.3)	4,640 (32.7)	3,165 (33.3)	2,102 (32.2)
Third quintile (40-60%)	5,140 (24.3)	4,791 (23.8)	3,235 (22.8)	2,203 (23.2)	1,642 (25.1)
Fourth quintile (60-80%)	3,094 (14.6)	2,868 (14.2)	1,916 (13.5)	1,204 (12.7)	822 (12.6)
Fifth quintile (80-100%)	2,173 (10.3)	1,867 (9.3)	1,226 (8.7)	816 (8.6)	580 (8.9)
Private household with an unknown income	[masked]‖	16 (0.1)	[masked]‖	[masked]‖	[masked]‖
Institutional household	[masked]‖	651 (3.2)	[masked]‖	[masked]‖	[masked]‖
Marital status, n (%)					
Married or in partnership	11,540 (54.4)	10,987 (54.4)	7,616 (53.6)	5,047 (53.0)	3,558 (54.4)
Unmarried or single	1,327 (6.3)	1,221 (6.0)	876 (6.2)	629 (6.6)	419 (6.4)
Divorced	2,026 (9.5)	1,888 (9.3)	1,389 (9.8)	919 (9.7)	650 (9.9)
Widowed	6,334 (29.8)	6,113 (30.2)	4,322 (30.4)	2,923 (30.7)	1,918 (29.3)
Type of atrial fibrillation, n (%)					
Paroxysmal atrial fibrillation (I48.0)	-	3,101 (15.3)	2,219 (15.6)	1,369 (14.4)	980 (15.0)
Persistent atrial fibrillation (I48.1)	-	453 (2.2)	384 (2.7)	243 (2.6)	196 (3.0)

© 2023 Chen Q et al. *JAMA Network Open.*

Chronic atrial fibrillation (I48.2)	-	970 (4.8)	895 (6.3)	804 (8.4)	657 (10.0)
Type I atrial flutter (I48.3)	-	116 (0.6)	100 (0.7)	61 (0.6)	38 (0.6)
Type II atrial flutter (I48.4)	-	58 (0.3)	29 (0.2)	22 (0.2)	15 (0.2)
Unspecified atrial fibrillation (I48.9)	-	15,511 (76.8)	10,576 (74.5)	7,019 (73.7)	4,659 (71.2)
Diagnosis made before 2015	21,227 (100.0)	-	-	-	-
CHA_2DS_2-VASc score, mean ± SD	3.15 ± 1.59	3.26 ± 1.57	3.36 ± 1.56	3.40 ± 1.54	3.37 ± 1.55
CHA_2DS_2-VASc score, n (%)					
0	906 (4.3)	709 (3.5)	414 (2.9)	244 (2.6)	180 (2.8)
1	2,257 (10.6)	1,839 (9.1)	1,177 (8.3)	741 (7.8)	529 (8.1)
**≥2**	**18,064 (85.1)**	**17,661 (87.4)**	**12,612 (88.8)**	**8,533 (89.7)**	**5,836 (89.2)**
2	4,204 (19.8)	3,889 (19.2)	2,539 (17.9)	1,647 (17.3)	1,160 (17.7)
3	5,310 (25.0)	5,098 (25.2)	3,649 (25.7)	2,502 (26.3)	1,711 (26.1)
4	4,505 (21.2)	4,487 (22.2)	3,274 (23.1)	2,215 (23.3)	1,498 (22.9)
5	2,540 (12.0)	2,642 (13.1)	1,923 (13.5)	1,366 (14.4)	899 (13.7)
6	1,041 (4.9)	1,059 (5.2)	855 (6.0)	563 (5.9)	402 (6.1)
≥7	464 (2.2)	486 (2.4)	372 (2.6)	240 (2.5)	166 (2.5)
HAS-BLED score[*], mean ± SD	1.86 ± 1.01	1.88 ± 0.99	1.93 ± 1.00	1.93 ± 1.03	1.90 ± 1.03
HAS-BLED score[*], n (%)					
0	1,428 (6.7)	1,183 (5.9)	726 (5.1)	490 (5.1)	380 (5.8)
1	6,648 (31.3)	6,326 (31.3)	4,379 (30.8)	3,017 (31.7)	2,112 (32.3)
2	7,855 (37.0)	7,674 (38.0)	5,345 (37.6)	3,420 (35.9)	2,344 (35.8)
≥3	5,296 (24.9)	5,026 (24.9)	3,753 (26.4)	2,591 (27.2)	1,709 (26.1)
Pre-existing chronic use of antithrombotic agents[§]					
**Oral anticoagulant, n (%)**	-	-	-	-	-
Vitamin K antagonist, n (%)	-	-	-	-	-
Direct oral anticoagulant, n (%)	-	-	-	-	-
Heparin group, n (%)	264 (1.2)	259 (1.3)	201 (1.4)	127 (1.3)	88 (1.3)
Antiplatelet agent, n (%)	7,284 (34.3)	6,771 (33.5)	4,462 (31.4)	2,615 (27.5)	1,667 (25.5)
Comorbidities (or medical history)[‖], n (%)					
**Asthma**	**421 (2.0)**	**449 (2.2)**	**336 (2.4)**	**229 (2.4)**	**183 (2.8)**
Prior 4 years	195 (0.9)	226 (1.1)	181 (1.3)	114 (1.2)	101 (1.5)
Index hospitalization	291 (1.4)	300 (1.5)	201 (1.4)	150 (1.6)	122 (1.9)
**Chronic obstructive pulmonary disease**	**2,755 (13.0)**	**2,678 (13.3)**	**1,910 (13.4)**	**1,226 (12.9)**	**824 (12.6)**
Prior 4 years	1,282 (6.0)	1,300 (6.4)	1,016 (7.2)	660 (6.9)	446 (6.8)
Index hospitalization	2,318 (10.9)	2,216 (11.0)	1,533 (10.8)	988 (10.4)	653 (10.0)
**Other chronic lung diseases**	**448 (2.1)**	**371 (1.8)**	**294 (2.1)**	**223 (2.3)**	**158 (2.4)**
Prior 4 years	263 (1.2)	188 (0.9)	151 (1.1)	112 (1.2)	76 (1.2)

© 2023 Chen Q et al. *JAMA Network Open.*

Index hospitalization	213 (1.0)	214 (1.1)	170 (1.2)	144 (1.5)	94 (1.4)
**Heart failure**	**5,265 (24.8)**	**5,219 (25.8)**	**3,942 (27.8)**	**2,741 (28.8)**	**1,926 (29.4)**
Prior 4 years	1,197 (5.6)	1,214 (6.0)	1,074 (7.6)	817 (8.6)	596 (9.1)
Index hospitalization	4,620 (21.8)	4,567 (22.6)	3,423 (24.1)	2,326 (24.4)	1,629 (24.9)
**Myocardial infarction (history)**	**2,786 (13.1)**	**2,941 (14.6)**	**2,297 (16.2)**	**1,563 (16.4)**	**1,019 (15.6)**
Prior 4 years	1,734 (8.2)	1,769 (8.8)	1,439 (10.1)	1,028 (10.8)	684 (10.5)
Index hospitalization	1,256 (5.9)	1,429 (7.1)	1,075 (7.6)	707 (7.4)	438 (6.7)
**Hypertension**	**7,998 (37.7)**	**8,119 (40.2)**	**5,863 (41.3)**	**4,090 (43.0)**	**2,907 (44.4)**
Prior 4 years	2,918 (13.7)	3,375 (16.7)	2,623 (18.5)	1,887 (19.8)	1,350 (20.6)
Index hospitalization	6,696 (31.5)	6,680 (33.1)	4,701 (33.1)	3,279 (34.5)	2,371 (36.2)
**Other valvular heart disease**[#]	**3,731 (17.6)**	**3,655 (18.1)**	**2,892 (20.4)**	**2,253 (23.7)**	**1,704 (26.0)**
Prior 4 years	1,456 (6.9)	1,544 (7.6)	1,336 (9.4)	1,080 (11.3)	895 (13.7)
Index hospitalization	3,158 (14.9)	3,124 (15.5)	2,476 (17.4)	1,920 (20.2)	1,466 (22.4)
**Peripheral artery disease**	**600 (2.8)**	**691 (3.4)**	**522 (3.7)**	**350 (3.7)**	**263 (4.0)**
Prior 4 years	298 (1.4)	361 (1.8)	295 (2.1)	214 (2.2)	164 (2.5)
Index hospitalization	396 (1.9)	445 (2.2)	326 (2.3)	191 (2.0)	144 (2.2)
**Liver diseases**	**539 (2.5)**	**516 (2.6)**	**409 (2.9)**	**287 (3.0)**	**208 (3.2)**
Prior 4 years	206 (1.0)	207 (1.0)	197 (1.4)	138 (1.4)	106 (1.6)
Index hospitalization	374 (1.8)	342 (1.7)	255 (1.8)	186 (2.0)	131 (2.0)
**Gastroesophageal reflux disease**	**104 (0.5)**	**97 (0.5)**	**87 (0.6)**	**58 (0.6)**	**40 (0.6)**
Prior 4 years	61 (0.3)	71 (0.4)	54 (0.4)	41 (0.4)	31 (0.5)
Index hospitalization	44 (0.2)	29 (0.1)	35 (0.2)	20 (0.2)	10 (0.2)
**Peptic ulcer disease**	**168 (0.8)**	**135 (0.7)**	**112 (0.8)**	**95 (1.0)**	**61 (0.9)**
Prior 4 years	82 (0.4)	59 (0.3)	59 (0.4)	50 (0.5)	34 (0.5)
Index hospitalization	89 (0.4)	80 (0.4)	55 (0.4)	48 (0.5)	30 (0.5)
**Chronic kidney diseases**	**2,918 (13.7)**	**3,122 (15.4)**	**2,803 (19.7)**	**2,289 (24.0)**	**1,587 (24.2)**
Prior 4 years	1,192 (5.6)	1,314 (6.5)	1,224 (8.6)	1,054 (11.1)	802 (12.3)
Index hospitalization	2,306 (10.9)	2,496 (12.4)	2,250 (15.8)	1,861 (19.6)	1,311 (20.0)
**Anaemia**	**2,263 (10.7)**	**2,345 (11.6)**	**1,960 (13.8)**	**1,457 (15.3)**	**1,098 (16.8)**
Prior 4 years	1,170 (5.5)	1,210 (6.0)	1,011 (7.1)	755 (7.9)	598 (9.1)
Index hospitalization	1,322 (6.2)	1,380 (6.8)	1,167 (8.2)	882 (9.3)	628 (9.6)
**Coagulopathy**	**335 (1.6)**	**435 (2.2)**	**396 (2.8)**	**358 (3.8)**	**291 (4.4)**
Prior 4 years	94 (0.4)	147 (0.7)	147 (1.0)	138 (1.4)	99 (1.5)
Index hospitalization	250 (1.2)	304 (1.5)	267 (1.9)	228 (2.4)	206 (3.1)
**Diabetes**	**4,275 (20.1)**	**4,304 (21.3)**	**3,288 (23.2)**	**2,230 (23.4)**	**1,549 (23.7)**
Prior 4 years	1,995 (9.4)	2,188 (10.8)	1,738 (12.2)	1,239 (13.0)	856 (13.1)
Index hospitalization	3,690 (17.4)	3,700 (18.3)	2,875 (20.2)	1,934 (20.3)	1,382 (21.1)

© 2023 Chen Q et al. *JAMA Network Open.*

**Thyroid disease**	**776 (3.7)**	**712 (3.5)**	**518 (3.6)**	**320 (3.4)**	**220 (3.4)**
Prior 4 years	231 (1.1)	220 (1.1)	197 (1.4)	140 (1.5)	93 (1.4)
Index hospitalization	598 (2.8)	553 (2.7)	373 (2.6)	219 (2.3)	160 (2.4)
**Ischemic stroke (history)**	**911 (4.3)**	**860 (4.3)**	**605 (4.3)**	**430 (4.5)**	**274 (4.2)**
Prior 4 years	612 (2.9)	611 (3.0)	402 (2.8)	302 (3.2)	174 (2.7)
Index hospitalization	414 (2.0)	334 (1.7)	265 (1.9)	178 (1.9)	138 (2.1)
**TIA**	**689 (3.2)**	**572 (2.8)**	**395 (2.8)**	**247 (2.6)**	**129 (2.0)**
Prior 4 years	349 (1.6)	295 (1.5)	223 (1.6)	155 (1.6)	77 (1.2)
Index hospitalization	357 (1.7)	287 (1.4)	187 (1.3)	99 (1.0)	55 (0.8)
**Other arterial thromboembolism**	**319 (1.5)**	**282 (1.4)**	**213 (1.5)**	**159 (1.7)**	**95 (1.5)**
Prior 4 years	229 (1.1)	188 (0.9)	141 (1.0)	89 (0.9)	60 (0.9)
Index hospitalization	108 (0.5)	112 (0.6)	81 (0.6)	78 (0.8)	38 (0.6)
**Parkinson's disease**	**151 (0.7)**	**231 (1.1)**	**150 (1.1)**	**86 (0.9)**	**71 (1.1)**
Prior 4 years	72 (0.3)	108 (0.5)	72 (0.5)	41 (0.4)	38 (0.6)
Index hospitalization	120 (0.6)	187 (0.9)	121 (0.9)	69 (0.7)	55 (0.8)
**Alzheimer's disease**	**168 (0.8)**	**173 (0.9)**	**143 (1.0)**	**94 (1.0)**	**71 (1.1)**
Prior 4 years	53 (0.2)	58 (0.3)	48 (0.3)	28 (0.3)	13 (0.2)
Index hospitalization	142 (0.7)	143 (0.7)	124 (0.9)	84 (0.9)	66 (1.0)
**Autoimmune disease**	**70 (0.3)**	**75 (0.4)**	**67 (0.5)**	**59 (0.6)**	**46 (0.7)**
Prior 4 years	42 (0.2)	51 (0.3)	53 (0.4)	38 (0.4)	32 (0.5)
Index hospitalization	37 (0.2)	33 (0.2)	22 (0.2)	28 (0.3)	20 (0.3)
**Systemic connective tissue disorders**	**281 (1.3)**	**300 (1.5)**	**230 (1.6)**	**151 (1.6)**	**126 (1.9)**
Prior 4 years	118 (0.6)	135 (0.7)	130 (0.9)	84 (0.9)	68 (1.0)
Index hospitalization	199 (0.9)	208 (1.0)	148 (1.0)	98 (1.0)	88 (1.3)
**Venous thromboembolism**	**523 (2.5)**	**547 (2.7)**	**400 (2.8)**	**237 (2.5)**	**148 (2.3)**
Prior 4 years to prior 6 months	120 (0.6)	141 (0.7)	116 (0.8)	56 (0.6)	44 (0.7)
Prior 6 months	415 (2.0)	422 (2.1)	297 (2.1)	182 (1.9)	105 (1.6)
**Deep vein thrombosis**	**198 (0.9)**	**211 (1.0)**	**155 (1.1)**	**104 (1.1)**	**59 (0.9)**
Prior 4 years to prior 6 months	47 (0.2)	62 (0.3)	48 (0.3)	29 (0.3)	20 (0.3)
Prior 6 months	153 (0.7)	153 (0.8)	109 (0.8)	75 (0.8)	39 (0.6)
**Pulmonary embolism**	**390 (1.8)**	**390 (1.9)**	**289 (2.0)**	**150 (1.6)**	**100 (1.5)**
Prior 4 years to prior 6 months	85 (0.4)	92 (0.5)	78 (0.5)	36 (0.4)	28 (0.4)
Prior 6 months	314 (1.5)	308 (1.5)	220 (1.5)	114 (1.2)	72 (1.1)
**Major bleeding**	**1,321 (6.2)**	**1,312 (6.5)**	**974 (6.9)**	**845 (8.9)**	**570 (8.7)**
Prior 4 years	800 (3.8)	766 (3.8)	581 (4.1)	504 (5.3)	322 (4.9)
Index hospitalization	592 (2.8)	621 (3.1)	446 (3.1)	390 (4.1)	279 (4.3)
**Intracranial haemorrhage**	**140 (0.7)**	**130 (0.6)**	**65 (0.5)**	**57 (0.6)**	**41 (0.6)**

© 2023 Chen Q et al. *JAMA Network Open.*

Prior 4 years	76 (0.4)	71 (0.4)	33 (0.2)	37 (0.4)	18 (0.3)
Index hospitalization	68 (0.3)	67 (0.3)	33 (0.2)	25 (0.3)	25 (0.4)
**Gastrointestinal bleeding**	**628 (3.0)**	**556 (2.8)**	**431 (3.0)**	**373 (3.9)**	**235 (3.6)**
Prior 4 years	397 (1.9)	358 (1.8)	278 (2.0)	230 (2.4)	143 (2.2)
Index hospitalization	251 (1.2)	219 (1.1)	172 (1.2)	164 (1.7)	103 (1.6)
**Malignant tumour**	**2,537 (12.0)**	**2,492 (12.3)**	**1,832 (12.9)**	**1,275 (13.4)**	**817 (12.5)**
Prior 4 years	1,990 (9.4)	1,963 (9.7)	1,424 (10.0)	995 (10.5)	650 (9.9)
Index hospitalization	1,259 (5.9)	1,317 (6.5)	989 (7.0)	644 (6.8)	403 (6.2)
≥1 hospitalization within the prior 4 years, n (%)	13,770 (64.9)	13,292 (65.8)	9,512 (67.0)	6,536 (68.7)	4,513 (69.0)
Information about the index hospitalization					
Length of hospital stay, days, median (IQR)	5 (1-9)	5 (1-9)	5 (1-9)	5 (1-10)	5 (1-10)
In-hospital mortality, n (%)	12 (0.1)	16 (0.1)	<10 (0.1)[‖]	10 (0.1)	<10 (<0.1)[‖]
**Main reason for admission to the index hospitalization**					
Atrial fibrillation	6,898 (32.5)	6,163 (30.5)	3,725 (26.2)	2,136 (22.4)	1,262 (19.3)
Heart failure	2,231 (10.5)	2,228 (11.0)	1,696 (11.9)	1,127 (11.8)	774 (11.8)
Ischemic heart diseases	2,061 (9.7)	2,075 (10.3)	1,522 (10.7)	1,010 (10.6)	727 (11.1)
Respiratory diseases	1,598 (7.5)	1,719 (8.5)	1,187 (8.4)	815 (8.6)	544 (8.3)
Injury/poisoning	840 (4.0)	875 (4.3)	678 (4.8)	532 (5.6)	448 (6.8)
Symptoms/signs/abnormal laboratory findings[**]	1,049 (4.9)	996 (4.9)	725 (5.1)	460 (4.8)	317 (4.8)
Neoplasms	731 (3.4)	779 (3.9)	543 (3.8)	362 (3.8)	228 (3.5)
Digestive diseases	822 (3.9)	829 (4.1)	528 (3.7)	405 (4.3)	252 (3.9)
Other heart valve disorders[§§]	855 (4.0)	817 (4.0)	765 (5.4)	670 (7.0)	548 (8.4)
Genitourinary diseases	556 (2.6)	534 (2.6)	467 (3.3)	356 (3.7)	272 (4.2)
Infectious/parasitic diseases	342 (1.6)	328 (1.6)	249 (1.8)	193 (2.0)	127 (1.9)
Endocrine/nutritional/metabolic diseases	231 (1.1)	217 (1.1)	174 (1.2)	91 (1.0)	103 (1.6)
Diseases of blood/blood-forming organs	133 (0.6)	105 (0.5)	102 (0.7)	85 (0.9)	47 (0.7)
Neurological disorders[##]	140 (0.7)	128 (0.6)	75 (0.5)	62 (0.7)	32 (0.5)

† First-generation immigrants refer to persons who were born abroad with at least one parent who was born abroad; second-generation immigrants refer to persons who was born in the Netherlands with at least one parent who was born abroad.
‡ Percentile groups were determined based on disposable income of private households of the complete target population in the database (instead of the study population only).
* Labile INR (international normalized ratio) was not included for calculation.
§ Pre-existing chronic use was defined as ≥2 (outpatient) prescription records of the same type of antithrombotic agents within six months (*i.e.*, 183 days) before the index dates (exclusive). Pre-existing chronic use of oral anticoagulant refers to persons with pre-existing chronic use of vitamin K antagonist or direct oral anticoagulant.
|| Unless otherwise stated, for each comorbidity (or medical history), prevalence calculated by screening all hospitalizations within the prior four years (including the index hospitalization), all hospitalizations within the prior four years (excluding the index hospitalization), and only the index hospitalization is presented, respectively.
Except for rheumatic mitral stenosis and mechanical heart valves
** Except for abnormalities of heart beat (ICD-10 code: R00) and cardiac murmurs and other cardiac sounds (ICD-10 code: R01)
§§ Except for endocarditis, valve unspecified, in diseases classified elsewhere (ICD-10 code: I398)

© 2023 Chen Q et al. *JAMA Network Open.*

Except for transient cerebral ischaemic attacks and related syndromes (ICD-10 code: G45) and vascular syndromes of brain in cerebrovascular diseases (ICD-10 code: G46)
||| Exact result was masked to prevent potential individual or group disclosure.
Abbreviations: SD, standard deviation; TIA, transient ischemic attack; IQR, interquartile range; ICD, International Classification of Diseases.

© 2023 Chen Q et al. *JAMA Network Open*.

eTable 14. Time Trends in Patient Characteristics of Incident Nonvalvular Atrial Fibrillation Among Patients Who First Received Direct Oral Anticoagulant as Oral Anticoagulant Within the 1-Year Follow-up, Excluding Patients With Preexisting Chronic Oral Anticoagulant Treatment

Cohort	2014 (N=3,188)	2015 (N=8,913)	2016 (N=14,588)	2017 (N=19,342)	2018 (N=21,013)
Age, years, mean ± SD	70.2 ± 11.4	70.6 ± 11.4	72.2 ± 11.3	73.4 ± 11.1	73.7 ± 11.2
Age group, years, n (%)					
18-24	[masked][‖]	[masked][‖]	[masked][‖]	14 (0.1)	19 (0.1)
25-34	[masked][‖]	[masked][‖]	[masked][‖]	42 (0.2)	70 (0.3)
35-44	53 (1.7)	153 (1.7)	163 (1.1)	188 (1.0)	206 (1.0)
45-54	223 (7.0)	634 (7.1)	849 (5.8)	929 (4.8)	948 (4.5)
55-64	632 (19.8)	1,686 (18.9)	2,409 (16.5)	2,869 (14.8)	2,887 (13.7)
65-74	1,173 (36.8)	3,113 (34.9)	4,904 (33.6)	6,249 (32.3)	6,828 (32.5)
75-84	817 (25.6)	2,433 (27.3)	4,345 (29.8)	6,109 (31.6)	6,749 (32.1)
≥85	272 (8.5)	853 (9.6)	1,864 (12.8)	2,942 (15.2)	3,306 (15.7)
Sex, n (%)					
Male	1,798 (56.4)	4,811 (54.0)	7,912 (54.2)	10,423 (53.9)	11,427 (54.4)
Female	1,390 (43.6)	4,102 (46.0)	6,676 (45.8)	8,919 (46.1)	9,586 (45.6)
Immigration background[†], n (%)					
Native Dutch	2,798 (87.8)	7,760 (87.1)	12,741 (87.3)	16,885 (87.3)	18,330 (87.2)
First-generation immigrants	206 (6.5)	617 (6.9)	986 (6.8)	1,357 (7.0)	1,576 (7.5)
Second-generation immigrants	184 (5.8)	536 (6.0)	861 (5.9)	1,100 (5.7)	1,107 (5.3)
Standardized household income[‡], n (%)					
First quintile (0-20%)	404 (12.7)	1,170 (13.2)	2,206 (15.1)	3,076 (15.9)	3,555 (17.0)
Second quintile (20-40%)	766 (24.1)	2,292 (25.8)	3,950 (27.1)	5,539 (28.7)	6,165 (29.4)
Third quintile (40-60%)	777 (24.4)	2,141 (24.1)	3,526 (24.2)	4,742 (24.6)	5,088 (24.3)
Fourth quintile (60-80%)	592 (18.6)	1,655 (18.6)	2,551 (17.5)	3,118 (16.2)	3,295 (15.7)
Fifth quintile (80-100%)	597 (18.8)	1,499 (16.9)	2,092 (14.4)	2,503 (13.0)	2,462 (11.7)
Private household with an unknown income	[masked][‖]	[masked][‖]	12 (0.1)	10 (0.1)	[masked][‖]
Institutional household	[masked][‖]	[masked][‖]	232 (1.6)	302 (1.6)	[masked][‖]
Marital status, n (%)					
Married or in partnership	2,025 (63.5)	5,553 (62.3)	8,649 (59.3)	11,139 (57.6)	11,871 (56.5)
Unmarried or single	243 (7.6)	676 (7.6)	1,082 (7.4)	1,493 (7.7)	1,682 (8.0)
Divorced	327 (10.3)	966 (10.8)	1,616 (11.1)	2,125 (11.0)	2,423 (11.5)
Widowed	593 (18.6)	1,718 (19.3)	3,241 (22.2)	4,585 (23.7)	5,037 (24.0)
Type of atrial fibrillation, n (%)					
Paroxysmal atrial fibrillation (I48.0)	-	1,620 (18.2)	2,530 (17.3)	3,231 (16.7)	3,286 (15.6)
Persistent atrial fibrillation (I48.1)	-	219 (2.5)	312 (2.1)	385 (2.0)	381 (1.8)

© 2023 Chen Q et al. *JAMA Network Open.*

Chronic atrial fibrillation (I48.2)	-	112 (1.3)	179 (1.2)	239 (1.2)	249 (1.2)
Type I atrial flutter (I48.3)	-	69 (0.8)	141 (1.0)	231 (1.2)	278 (1.3)
Type II atrial flutter (I48.4)	-	38 (0.4)	40 (0.3)	53 (0.3)	54 (0.3)
Unspecified atrial fibrillation (I48.9)	-	6,855 (76.9)	11,386 (78.1)	15,203 (78.6)	16,765 (79.8)
Diagnosis made before 2015	3,188 (100.0)	-	-	-	-
CHA_2DS_2-VASc score, mean ± SD	2.36 ± 1.56	2.44 ± 1.56	2.65 ± 1.58	2.84 ± 1.60	2.90 ± 1.59
CHA_2DS_2-VASc score, n (%)					
0	353 (11.1)	932 (10.5)	1,230 (8.4)	1,299 (6.7)	1,283 (6.1)
1	653 (20.5)	1,715 (19.2)	2,342 (16.1)	2,765 (14.3)	2,886 (13.7)
**≥2**	**2,182 (68.4)**	**6,266 (70.3)**	**11,016 (75.5)**	**15,278 (79.0)**	**16,844 (80.2)**
2	830 (26.0)	2,180 (24.5)	3,485 (23.9)	4,309 (22.3)	4,479 (21.3)
3	651 (20.4)	1,968 (22.1)	3,405 (23.3)	4,665 (24.1)	5,140 (24.5)
4	389 (12.2)	1,237 (13.9)	2,326 (15.9)	3,512 (18.2)	3,982 (19.0)
5	200 (6.3)	582 (6.5)	1,170 (8.0)	1,745 (9.0)	2056 (9.8)
6	85 (2.7)	217 (2.4)	444 (3.0)	713 (3.7)	862 (4.1)
≥7	27 (0.8)	82 (0.9)	186 (1.3)	334 (1.7)	325 (1.5)
HAS-BLED score[*], mean ± SD	1.51 ± 0.97	1.49 ± 0.97	1.58 ± 0.97	1.65 ± 0.98	1.68 ± 0.99
HAS-BLED score[*], n (%)					
0	456 (14.3)	1,320 (14.8)	1,811 (12.4)	2,080 (10.8)	2,150 (10.2)
1	1,211 (38.0)	3,398 (38.1)	5,386 (36.9)	6,932 (35.8)	7,474 (35.6)
2	1,035 (32.5)	2,901 (32.5)	4,984 (34.2)	6,760 (34.9)	7,267 (34.6)
≥3	486 (15.2)	1,294 (14.5)	2,407 (16.5)	3,570 (18.5)	4,122 (19.6)
Pre-existing chronic use of antithrombotic agents[§]					
**Oral anticoagulant, n (%)**	-	-	-	-	-
Vitamin K antagonist, n (%)	-	-	-	-	-
Direct oral anticoagulant, n (%)	-	-	-	-	-
Heparin group, n (%)	13 (0.4)	41 (0.5)	75 (0.5)	117 (0.6)	142 (0.7)
Antiplatelet agent, n (%)	1,048 (32.9)	2,740 (30.7)	4,832 (33.1)	6,780 (35.1)	7,404 (35.2)
Comorbidities (or medical history)[‖], n (%)					
**Asthma**	**56 (1.8)**	**171 (1.9)**	**344 (2.4)**	**455 (2.4)**	**592 (2.8)**
Prior 4 years	20 (0.6)	79 (0.9)	172 (1.2)	217 (1.1)	306 (1.5)
Index hospitalization	45 (1.4)	108 (1.2)	236 (1.6)	320 (1.7)	425 (2.0)
**Chronic obstructive pulmonary disease**	**262 (8.2)**	**752 (8.4)**	**1,515 (10.4)**	**2,140 (11.1)**	**2,419 (11.5)**
Prior 4 years	115 (3.6)	378 (4.2)	772 (5.3)	1,087 (5.6)	1,247 (5.9)
Index hospitalization	219 (6.9)	622 (7.0)	1,267 (8.7)	1,764 (9.1)	2,048 (9.7)
**Other chronic lung diseases**	**44 (1.4)**	**97 (1.1)**	**224 (1.5)**	**376 (1.9)**	**474 (2.3)**
Prior 4 years	25 (0.8)	50 (0.6)	122 (0.8)	175 (0.9)	224 (1.1)

© 2023 Chen Q et al. *JAMA Network Open.*

Index hospitalization	20 (0.6)	57 (0.6)	124 (0.9)	243 (1.3)	320 (1.5)
**Heart failure**	**402 (12.6)**	**1,293 (14.5)**	**2,411 (16.5)**	**3,694 (19.1)**	**4,446 (21.2)**
Prior 4 years	45 (1.4)	167 (1.9)	385 (2.6)	592 (3.1)	679 (3.2)
Index hospitalization	375 (11.8)	1,197 (13.4)	2,212 (15.2)	3,358 (17.4)	4,115 (19.6)
**Myocardial infarction (history)**	**224 (7.0)**	**650 (7.3)**	**1,401 (9.6)**	**2,255 (11.7)**	**2,610 (12.4)**
Prior 4 years	147 (4.6)	425 (4.8)	909 (6.2)	1,368 (7.1)	1,515 (7.2)
Index hospitalization	87 (2.7)	259 (2.9)	608 (4.2)	1,117 (5.8)	1,392 (6.6)
**Hypertension**	**1,052 (33.0)**	**2,922 (32.8)**	**5,195 (35.6)**	**7,330 (37.9)**	**8,274 (39.4)**
Prior 4 years	283 (8.9)	944 (10.6)	1,897 (13.0)	2,866 (14.8)	3,314 (15.8)
Index hospitalization	926 (29.0)	2,466 (27.7)	4,396 (30.1)	6,105 (31.6)	6,973 (33.2)
**Other valvular heart disease**[#]	**262 (8.2)**	**719 (8.1)**	**1,316 (9.0)**	**2,022 (10.5)**	**2,371 (11.3)**
Prior 4 years	67 (2.1)	183 (2.1)	447 (3.1)	700 (3.6)	817 (3.9)
Index hospitalization	217 (6.8)	621 (7.0)	1,107 (7.6)	1,699 (8.8)	2,003 (9.5)
**Peripheral artery disease**	**52 (1.6)**	**164 (1.8)**	**288 (2.0)**	**469 (2.4)**	**540 (2.6)**
Prior 4 years	27 (0.8)	82 (0.9)	172 (1.2)	279 (1.4)	300 (1.4)
Index hospitalization	29 (0.9)	108 (1.2)	168 (1.2)	269 (1.4)	321 (1.5)
**Liver diseases**	**37 (1.2)**	**153 (1.7)**	**260 (1.8)**	**397 (2.1)**	**456 (2.2)**
Prior 4 years	12 (0.4)	64 (0.7)	120 (0.8)	174 (0.9)	220 (1.0)
Index hospitalization	26 (0.8)	97 (1.1)	161 (1.1)	252 (1.3)	274 (1.3)
**Gastroesophageal reflux disease**	**12 (0.4)**	**32 (0.4)**	**64 (0.4)**	**113 (0.6)**	**129 (0.6)**
Prior 4 years	[masked][‖]	22 (0.2)	45 (0.3)	83 (0.4)	90 (0.4)
Index hospitalization	[masked][‖]	10 (0.1)	19 (0.1)	32 (0.2)	42 (0.2)
**Peptic ulcer disease**	**<10 (0.2)**[‖]	**34 (0.4)**	**57 (0.4)**	**89 (0.5)**	**106 (0.5)**
Prior 4 years	[masked][‖]	21 (0.2)	41 (0.3)	48 (0.2)	51 (0.2)
Index hospitalization	[masked][‖]	14 (0.2)	17 (0.1)	43 (0.2)	58 (0.3)
**Chronic kidney diseases**	**145 (4.5)**	**487 (5.5)**	**1,095 (7.5)**	**1,752 (9.1)**	**2,110 (10.0)**
Prior 4 years	47 (1.5)	162 (1.8)	420 (2.9)	653 (3.4)	849 (4.0)
Index hospitalization	111 (3.5)	375 (4.2)	821 (5.6)	1,332 (6.9)	1,659 (7.9)
**Anaemia**	**151 (4.7)**	**418 (4.7)**	**1,023 (7.0)**	**1,734 (9.0)**	**2,082 (9.9)**
Prior 4 years	79 (2.5)	229 (2.6)	522 (3.6)	855 (4.4)	1,015 (4.8)
Index hospitalization	81 (2.5)	217 (2.4)	580 (4.0)	1,054 (5.4)	1,290 (6.1)
**Coagulopathy**	**18 (0.6)**	**59 (0.7)**	**132 (0.9)**	**215 (1.1)**	**289 (1.4)**
Prior 4 years	[masked][‖]	24 (0.3)	56 (0.4)	85 (0.4)	113 (0.5)
Index hospitalization	[masked][‖]	35 (0.4)	84 (0.6)	137 (0.7)	190 (0.9)
**Diabetes**	**428 (13.4)**	**1,214 (13.6)**	**2,328 (16.0)**	**3,410 (17.6)**	**3,896 (18.5)**
Prior 4 years	160 (5.0)	496 (5.6)	1,087 (7.5)	1,659 (8.6)	1,865 (8.9)
Index hospitalization	377 (11.8)	1,070 (12.0)	2,027 (13.9)	3,004 (15.5)	3,538 (16.8)

© 2023 Chen Q et al. *JAMA Network Open.*

**Thyroid disease**	**95 (3.0)**	**275 (3.1)**	**518 (3.6)**	**771 (4.0)**	**764 (3.6)**
Prior 4 years	17 (0.5)	62 (0.7)	143 (1.0)	204 (1.1)	204 (1.0)
Index hospitalization	85 (2.7)	235 (2.6)	409 (2.8)	635 (3.3)	637 (3.0)
**Ischemic stroke (history)**	**106 (3.3)**	**273 (3.1)**	**478 (3.3)**	**722 (3.7)**	**747 (3.6)**
Prior 4 years	71 (2.2)	193 (2.2)	373 (2.6)	558 (2.9)	542 (2.6)
Index hospitalization	51 (1.6)	111 (1.2)	143 (1.0)	245 (1.3)	299 (1.4)
**TIA**	**104 (3.3)**	**247 (2.8)**	**453 (3.1)**	**575 (3.0)**	**590 (2.8)**
Prior 4 years	38 (1.2)	110 (1.2)	186 (1.3)	301 (1.6)	282 (1.3)
Index hospitalization	70 (2.2)	144 (1.6)	285 (2.0)	287 (1.5)	324 (1.5)
**Other arterial thromboembolism**	**12 (0.4)**	**60 (0.7)**	**107 (0.7)**	**166 (0.9)**	**165 (0.8)**
Prior 4 years	[masked][‖]	50 (0.6)	77 (0.5)	108 (0.6)	98 (0.5)
Index hospitalization	[masked][‖]	14 (0.2)	34 (0.2)	62 (0.3)	69 (0.3)
**Parkinson's disease**	**17 (0.5)**	**52 (0.6)**	**127 (0.9)**	**187 (1.0)**	**192 (0.9)**
Prior 4 years	[masked][‖]	20 (0.2)	58 (0.4)	88 (0.5)	78 (0.4)
Index hospitalization	[masked][‖]	42 (0.5)	103 (0.7)	155 (0.8)	170 (0.8)
**Alzheimer's disease**	**<10 (0.3)**[‖]	**33 (0.4)**	**72 (0.5)**	**105 (0.5)**	**130 (0.6)**
Prior 4 years	[masked][‖]	13 (0.1)	26 (0.2)	31 (0.2)	33 (0.2)
Index hospitalization	[masked][‖]	25 (0.3)	61 (0.4)	89 (0.5)	116 (0.6)
**Autoimmune disease**	**<10 (0.3)**[‖]	**19 (0.2)**	**57 (0.4)**	**76 (0.4)**	**97 (0.5)**
Prior 4 years	[masked][‖]	11 (0.1)	42 (0.3)	55 (0.3)	72 (0.3)
Index hospitalization	[masked][‖]	11 (0.1)	26 (0.2)	34 (0.2)	41 (0.2)
**Systemic connective tissue disorders**	**19 (0.6)**	**85 (1.0)**	**170 (1.2)**	**247 (1.3)**	**284 (1.4)**
Prior 4 years	[masked][‖]	37 (0.4)	93 (0.6)	141 (0.7)	133 (0.6)
Index hospitalization	[masked][‖]	61 (0.7)	109 (0.7)	153 (0.8)	204 (1.0)
**Venous thromboembolism**	**24 (0.8)**	**65 (0.7)**	**216 (1.5)**	**455 (2.4)**	**494 (2.4)**
Prior 4 years to prior 6 months	11 (0.3)	25 (0.3)	61 (0.4)	99 (0.5)	83 (0.4)
Prior 6 months	13 (0.4)	42 (0.5)	159 (1.1)	368 (1.9)	414 (2.0)
**Deep vein thrombosis**	**<10 (0.3)**[‖]	**30 (0.3)**	**71 (0.5)**	**176 (0.9)**	**158 (0.8)**
Prior 4 years to prior 6 months	[masked][‖]	10 (0.1)	20 (0.1)	45 (0.2)	31 (0.1)
Prior 6 months	[masked][‖]	21 (0.2)	51 (0.3)	134 (0.7)	127 (0.6)
**Pulmonary embolism**	**18 (0.6)**	**44 (0.5)**	**174 (1.2)**	**324 (1.7)**	**375 (1.8)**
Prior 4 years to prior 6 months	[masked][‖]	18 (0.2)	48 (0.3)	67 (0.3)	61 (0.3)
Prior 6 months	[masked][‖]	26 (0.3)	129 (0.9)	264 (1.4)	317 (1.5)
**Major bleeding**	**93 (2.9)**	**330 (3.7)**	**563 (3.9)**	**918 (4.7)**	**1,079 (5.1)**
Prior 4 years	59 (1.9)	202 (2.3)	355 (2.4)	521 (2.7)	615 (2.9)
Index hospitalization	36 (1.1)	140 (1.6)	231 (1.6)	435 (2.2)	506 (2.4)
**Intracranial haemorrhage**	**11 (0.3)**	**46 (0.5)**	**71 (0.5)**	**94 (0.5)**	**97 (0.5)**

© 2023 Chen Q et al. *JAMA Network Open.*

Prior 4 years	[masked][‖‖]	20 (0.2)	40 (0.3)	51 (0.3)	57 (0.3)
Index hospitalization	[masked][‖‖]	27 (0.3)	35 (0.2)	48 (0.2)	47 (0.2)
**Gastrointestinal bleeding**	**38 (1.2)**	**145 (1.6)**	**247 (1.7)**	**395 (2.0)**	**455 (2.2)**
Prior 4 years	25 (0.8)	99 (1.1)	193 (1.3)	246 (1.3)	285 (1.4)
Index hospitalization	13 (0.4)	51 (0.6)	63 (0.4)	162 (0.8)	184 (0.9)
**Malignant tumour**	**249 (7.8)**	**724 (8.1)**	**1,456 (10.0)**	**2,146 (11.1)**	**2,523 (12.0)**
Prior 4 years	203 (6.4)	581 (6.5)	1,162 (8.0)	1,728 (8.9)	1,974 (9.4)
Index hospitalization	119 (3.7)	331 (3.7)	731 (5.0)	1,144 (5.9)	1,453 (6.9)
≥1 hospitalization within the prior 4 years, n (%)	1,753 (55.0)	4,799 (53.8)	8,257 (56.6)	11,213 (58.0)	11,948 (56.9)
Information about the index hospitalization					
Length of hospital stay, days, median (IQR)	1 (1-5)	1 (0-5)	2 (0-6)	3 (1-7)	3 (1-7)
In-hospital mortality, n (%)	<10 (0.0)[‖‖]	<10 (0.0)[‖‖]	<10 (0.0)[‖‖]	<10 (0.0)[‖‖]	14 (0.1)
**Main reason for admission to the index hospitalization**					
Atrial fibrillation	1,822 (57.2)	5,440 (61.0)	7,753 (53.1)	8,777 (45.4)	8,664 (41.2)
Heart failure	183 (5.7)	590 (6.6)	1,055 (7.2)	1,534 (7.9)	1,876 (8.9)
Ischemic heart diseases	161 (5.1)	400 (4.5)	877 (6.0)	1,470 (7.6)	1,757 (8.4)
Respiratory diseases	132 (4.1)	459 (5.1)	975 (6.7)	1,483 (7.7)	1,866 (8.9)
Injury/poisoning	79 (2.5)	167 (1.9)	387 (2.7)	644 (3.3)	732 (3.5)
Symptoms/signs/abnormal laboratory findings[**]	158 (5.0)	361 (4.1)	560 (3.8)	779 (4.0)	824 (3.9)
Neoplasms	71 (2.2)	165 (1.9)	421 (2.9)	639 (3.3)	800 (3.8)
Digestive diseases	81 (2.5)	203 (2.3)	379 (2.6)	647 (3.3)	717 (3.4)
Other heart valve disorders[§§]	24 (0.8)	63 (0.7)	156 (1.1)	264 (1.4)	319 (1.5)
Genitourinary diseases	42 (1.3)	90 (1.0)	205 (1.4)	391 (2.0)	496 (2.4)
Infectious/parasitic diseases	21 (0.7)	66 (0.7)	184 (1.3)	305 (1.6)	350 (1.7)
Endocrine/nutritional/metabolic diseases	20 (0.6)	52 (0.6)	100 (0.7)	176 (0.9)	224 (1.1)
Diseases of blood/blood-forming organs	<10 (<0.2)[‖‖]	18 (0.2)	41 (0.3)	91 (0.5)	100 (0.5)
Neurological disorders[##]	27 (0.8)	41 (0.5)	60 (0.4)	123 (0.6)	106 (0.5)

† First-generation immigrants refer to persons who were born abroad with at least one parent who was born abroad; second-generation immigrants refer to persons who was born in the Netherlands with at least one parent who was born abroad.
‡ Percentile groups were determined based on disposable income of private households of the complete target population in the database (instead of the study population only).
* Labile INR (international normalized ratio) was not included for calculation.
§ Pre-existing chronic use was defined as ≥2 (outpatient) prescription records of the same type of antithrombotic agents within six months (*i.e.*, 183 days) before the index dates (exclusive). Pre-existing chronic use of oral anticoagulant refers to persons with pre-existing chronic use of vitamin K antagonist or direct oral anticoagulant.
|| Unless otherwise stated, for each comorbidity (or medical history), prevalence calculated by screening all hospitalizations within the prior four years (including the index hospitalization), all hospitalizations within the prior four years (excluding the index hospitalization), and only the index hospitalization is presented, respectively.
Except for rheumatic mitral stenosis and mechanical heart valves
** Except for abnormalities of heart beat (ICD-10 code: R00) and cardiac murmurs and other cardiac sounds (ICD-10 code: R01)
§§ Except for endocarditis, valve unspecified, in diseases classified elsewhere (ICD-10 code: I398)

© 2023 Chen Q et al. *JAMA Network Open.*

Except for transient cerebral ischaemic attacks and related syndromes (ICD-10 code: G45) and vascular syndromes of brain in cerebrovascular diseases (ICD-10 code: G46)
||| Exact result was masked to prevent potential individual or group disclosure.
Abbreviations: SD, standard deviation; TIA, transient ischemic attack; IQR, interquartile range; ICD, International Classification of Diseases.

© 2023 Chen Q et al. *JAMA Network Open*.

eTable 15. Risk of Clinical Events Within the 1-Year Follow-up After Incident Nonvalvular Atrial Fibrillation Diagnosis

Clinical event†	Cohort	No. at risk‡	Follow-up (days)	Observation time (PYs)	No. events	Cumulative incidence (%, 95%CI)*	Incidence rate (per 100 PYs, 95%CI)
Ischemic stroke	2014	55,880	0-30	4,392	279	0.50 (0.44-0.56)	6.35 (5.63-7.14)
			0-90	12,687	468	0.84 (0.76-0.92)	3.69 (3.36-4.04)
			0-180	24,605	632	1.13 (1.05-1.22)	2.57 (2.37-2.78)
			0-270	36,122	770	1.38 (1.28-1.48)	2.13 (1.98-2.29)
			0-365	47,930	909	1.63 (1.52-1.73)	1.90 (1.78-2.02)
	2015	61,317	0-30	4,819	312	0.51 (0.45-0.57)	6.47 (5.78-7.23)
			0-90	13,944	514	0.84 (0.77-0.91)	3.69 (3.37-4.02)
			0-180	27,089	697	1.14 (1.06-1.22)	2.57 (2.39-2.77)
			0-270	39,810	866	1.41 (1.32-1.51)	2.18 (2.03-2.33)
			0-365	52,877	1,003	1.64 (1.54-1.74)	1.90 (1.78-2.02)
	2016	60,018	0-30	4,719	313	0.52 (0.47-0.58)	6.63 (5.92-7.41)
			0-90	13,666	472	0.79 (0.72-0.86)	3.45 (3.15-3.78)
			0-180	26,578	643	1.07 (0.99-1.16)	2.42 (2.24-2.61)
			0-270	39,101	777	1.29 (1.21-1.39)	1.99 (1.85-2.13)
			0-365	51,971	912	1.52 (1.42-1.62)	1.75 (1.64-1.87)
	2017	62,390	0-30	4,905	273	0.44 (0.39-0.49)	5.57 (4.92-6.27)
			0-90	14,214	428	0.69 (0.62-0.75)	3.01 (2.73-3.31)
			0-180	27,663	610	0.98 (0.90-1.06)	2.21 (2.03-2.39)
			0-270	40,709	756	1.21 (1.13-1.30)	1.86 (1.73-1.99)
			0-365	54,099	917	1.47 (1.38-1.57)	1.70 (1.59-1.81)
	2018	61,696	0-30	4,837	264	0.43 (0.38-0.48)	5.46 (4.82-6.16)
			0-90	14,002	443	0.72 (0.65-0.79)	3.16 (2.88-3.47)
			0-180	27,258	616	1.00 (0.92-1.08)	2.26 (2.08-2.45)
			0-270	40,121	724	1.17 (1.09-1.26)	1.80 (1.68-1.94)
			0-365	53,363	857	1.39 (1.30-1.48)	1.61 (1.50-1.72)
Major bleeding	2014	54,773	0-30	4,311	313	0.57 (0.51-0.64)	7.26 (6.48-8.11)
			0-90	12,440	593	1.08 (1.00-1.17)	4.77 (4.39-5.17)
			0-180	24,098	897	1.64 (1.53-1.75)	3.72 (3.48-3.97)
			0-270	35,345	1,123	2.05 (1.93-2.17)	3.18 (2.99-3.37)
			0-365	46,860	1,368	2.50 (2.37-2.63)	2.92 (2.77-3.08)
	2015	60,221	0-30	4,740	336	0.56 (0.50-0.62)	7.09 (6.35-7.89)
			0-90	13,704	606	1.01 (0.93-1.09)	4.42 (4.08-4.79)
			0-180	26,600	888	1.47 (1.38-1.57)	3.34 (3.12-3.57)
			0-270	39,069	1,103	1.83 (1.73-1.94)	2.82 (2.66-2.99)
			0-365	51,862	1,336	2.22 (2.10-2.34)	2.58 (2.44-2.72)
	2016	59,078	0-30	4,654	293	0.50 (0.44-0.56)	6.30 (5.60-7.06)
			0-90	13,469	555	0.94 (0.86-1.02)	4.12 (3.78-4.48)
			0-180	26,172	823	1.39 (1.30-1.49)	3.14 (2.93-3.37)
			0-270	38,477	1,043	1.77 (1.66-1.87)	2.71 (2.55-2.88)
			0-365	51,108	1,266	2.14 (2.03-2.26)	2.48 (2.34-2.62)
	2017	61,372	0-30	4,832	268	0.44 (0.39-0.49)	5.55 (4.90-6.25)
			0-90	13,989	549	0.89 (0.82-0.97)	3.92 (3.60-4.27)
			0-180	27,198	796	1.30 (1.21-1.39)	2.93 (2.73-3.14)
			0-270	40,004	1020	1.66 (1.56-1.77)	2.55 (2.40-2.71)
			0-365	53,134	1246	2.03 (1.92-2.14)	2.35 (2.22-2.48)
	2018	60,701	0-30	4,763	308	0.51 (0.45-0.57)	6.47 (5.76-7.23)
			0-90	13,783	552	0.91 (0.84-0.99)	4.01 (3.68-4.35)
			0-180	26,813	825	1.36 (1.27-1.45)	3.08 (2.87-3.29)
			0-270	39,433	1,045	1.72 (1.62-1.83)	2.65 (2.49-2.82)
			0-365	52,412	1,259	2.07 (1.96-2.19)	2.40 (2.27-2.54)
ICH	2014	55,468	0-30	4,373	75	0.14 (0.11-0.17)	1.72 (1.35-2.15)
			0-90	12,649	139	0.25 (0.21-0.30)	1.10 (0.92-1.30)
			0-180	24,558	214	0.39 (0.34-0.44)	0.87 (0.76-1.00)

© 2023 Chen Q et al. *JAMA Network Open.*

			0-270	36,083	262	0.47 (0.42-0.53)	0.73 (0.64-0.82)
			0-365	47,915	332	0.60 (0.54-0.67)	0.69 (0.62-0.77)
	2015	60,907	0-30	4,802	72	0.12 (0.09-0.15)	1.50 (1.17-1.89)
			0-90	13,913	126	0.21 (0.17-0.25)	0.91 (0.75-1.08)
			0-180	27,061	187	0.31 (0.27-0.35)	0.69 (0.60-0.80)
			0-270	39,806	252	0.41 (0.37-0.47)	0.63 (0.56-0.72)
			0-365	52,912	313	0.51 (0.46-0.57)	0.59 (0.53-0.66)
	2016	59,655	0-30	4,707	50	0.08 (0.06-0.11)	1.06 (0.79-1.40)
			0-90	13,649	100	0.17 (0.14-0.20)	0.73 (0.60-0.89)
			0-180	26,575	156	0.26 (0.22-0.31)	0.59 (0.50-0.69)
			0-270	39,130	206	0.35 (0.30-0.40)	0.53 (0.46-0.60)
			0-365	52,047	259	0.43 (0.38-0.49)	0.50 (0.44-0.56)
	2017	62,005	0-30	4,888	58	0.09 (0.07-0.12)	1.19 (0.90-1.53)
			0-90	14,183	107	0.17 (0.14-0.21)	0.75 (0.62-0.91)
			0-180	27,630	149	0.24 (0.20-0.28)	0.54 (0.46-0.63)
			0-270	40,700	188	0.30 (0.26-0.35)	0.46 (0.40-0.53)
			0-365	54,138	223	0.36 (0.32-0.41)	0.41 (0.36-0.47)
	2018	61,339	0-30	4,822	44	0.07 (0.05-0.10)	0.91 (0.66-1.23)
			0-90	13,979	83	0.14 (0.11-0.17)	0.59 (0.47-0.74)
			0-180	27,244	143	0.23 (0.20-0.27)	0.52 (0.44-0.62)
			0-270	40,128	203	0.33 (0.29-0.38)	0.51 (0.44-0.58)
			0-365	53,408	247	0.40 (0.36-0.46)	0.46 (0.41-0.52)
GI bleeding	2014	55,522	0-30	4,368	131	0.24 (0.20-0.28)	3.00 (2.51-3.56)
			0-90	12,619	244	0.44 (0.39-0.50)	1.93 (1.70-2.19)
			0-180	24,479	366	0.66 (0.59-0.73)	1.50 (1.35-1.66)
			0-270	35,943	455	0.82 (0.75-0.90)	1.27 (1.15-1.39)
			0-365	47,701	548	0.99 (0.91-1.07)	1.15 (1.05-1.25)
	2015	60,982	0-30	4,799	159	0.26 (0.22-0.30)	3.31 (2.82-3.87)
			0-90	13,889	276	0.45 (0.40-0.51)	1.99 (1.76-2.24)
			0-180	26,990	394	0.65 (0.58-0.71)	1.46 (1.32-1.61)
			0-270	39,675	468	0.77 (0.70-0.84)	1.18 (1.08-1.29)
			0-365	52,710	571	0.94 (0.86-1.02)	1.08 (1.00-1.18)
	2016	59,716	0-30	4,703	138	0.23 (0.20-0.27)	2.93 (2.47-3.47)
			0-90	13,621	263	0.44 (0.39-0.50)	1.93 (1.70-2.18)
			0-180	26,495	388	0.65 (0.59-0.72)	1.46 (1.32-1.62)
			0-270	38,985	482	0.81 (0.74-0.88)	1.24 (1.13-1.35)
			0-365	51,826	566	0.95 (0.87-1.03)	1.09 (1.00-1.19)
	2017	62,071	0-30	4,885	133	0.21 (0.18-0.25)	2.72 (2.28-3.23)
			0-90	14,154	273	0.44 (0.39-0.49)	1.93 (1.71-2.17)
			0-180	27,543	397	0.64 (0.58-0.70)	1.44 (1.30-1.59)
			0-270	40,539	505	0.81 (0.75-0.89)	1.25 (1.14-1.36)
			0-365	53,883	615	0.99 (0.92-1.07)	1.14 (1.05-1.24)
	2018	61,388	0-30	4,818	163	0.27 (0.23-0.31)	3.38 (2.88-3.94)
			0-90	13,946	302	0.49 (0.44-0.55)	2.17 (1.93-2.42)
			0-180	27,154	417	0.68 (0.62-0.75)	1.54 (1.39-1.69)
			0-270	39,970	510	0.83 (0.76-0.91)	1.28 (1.17-1.39)
			0-365	53,169	615	1.00 (0.93-1.08)	1.16 (1.07-1.25)
All-cause mortality	2014	55,880	0-30	4,401	3,815	6.83 (6.62-7.04)	86.69 (83.96-89.49)
			0-90	12,729	6,242	11.17 (10.91-11.43)	49.04 (47.83-50.27)
			0-180	24,720	8,090	14.48 (14.19-14.77)	32.73 (32.02-33.45)
			0-270	36,330	9,404	16.83 (16.52-17.14)	25.88 (25.36-26.41)
			0-365	48,254	10,689	19.13 (18.80-19.45)	22.15 (21.73-22.58)
	2015	61,317	0-30	4,831	4,152	6.77 (6.57-6.97)	85.95 (83.35-88.60)
			0-90	13,997	6,540	10.67 (10.42-10.91)	46.73 (45.60-47.87)
			0-180	27,227	8,531	13.91 (13.64-14.19)	31.33 (30.67-32.00)
			0-270	40,056	9,943	16.22 (15.92-16.51)	24.82 (24.34-25.32)
			0-365	53,256	11,194	18.26 (17.95-18.56)	21.02 (20.63-21.41)
	2016	60,018	0-30	4,730	3,960	6.60 (6.40-6.80)	83.71 (81.13-86.36)

© 2023 Chen Q et al. *JAMA Network Open.*

			0-90	13,718	6,362	10.60 (10.35-10.85)	46.38 (45.25-47.53)
			0-180	26,712	8,104	13.50 (13.23-13.78)	30.34 (29.68-31.01)
			0-270	39,339	9,422	15.70 (15.41-15.99)	23.95 (23.47-24.44)
			0-365	52,337	10,675	17.79 (17.48-18.09)	20.40 (20.01-20.79)
	2017	62,390	0-30	4,915	4,170	6.68 (6.49-6.88)	84.84 (82.29-87.46)
			0-90	14,260	6,544	10.49 (10.25-10.73)	45.89 (44.79-47.02)
			0-180	27,782	8,354	13.39 (13.12-13.66)	30.07 (29.43-30.72)
			0-270	40,926	9,741	15.61 (15.33-15.90)	23.80 (23.33-24.28)
			0-365	54,443	11,062	17.73 (17.43-18.03)	20.32 (19.94-20.70)
	2018	61,696	0-30	4,846	4,343	7.04 (6.84-7.24)	89.61 (86.97-92.32)
			0-90	14,048	6,641	10.76 (10.52-11.01)	47.27 (46.14-48.43)
			0-180	27,384	8,439	13.68 (13.41-13.95)	30.82 (30.16-31.48)
			0-270	40,345	9,734	15.78 (15.49-16.06)	24.13 (23.65-24.61)
			0-365	53,709	10,900	17.67 (17.37-17.97)	20.29 (19.92-20.68)

† Including death caused by the clinical event

‡ Individuals who were primarily admitted to the index hospitalization for the clinical event were excluded.

* Estimated by the cumulative incidence competing risk method, except for all-cause mortality which was estimated by the Kaplan-Meier estimator

Abbreviations: PY, person-year; CI, confidence interval; ICH, intracranial haemorrhage; GI, gastrointestinal.

© 2023 Chen Q et al. *JAMA Network Open.*

eTable 16. Time Trends in Prognosis Within 1 Year After Incident Nonvalvular Atrial Fibrillation Diagnosis, Excluding Patients With Preexisting Chronic Use of Oral Anticoagulants

Clinical event†	Cohort	No. at risk‡	Observation time (PYs)	No. events	Cumulative incidence (%, 95%CI)*	Incidence rate (per 100 PYs, 95%CI)	Crude HR (95%CI)	Adjusted HR (95%CI)§
Ischemic stroke	2014	40,663	34,556	717	1.76 (1.64-1.89)	2.07 (1.93-2.23)	1.26 (1.13-1.41)	1.27 (1.10-1.47)
	2015	44,149	37,667	733	1.66 (1.54-1.78)	1.95 (1.81-2.09)	1.19 (1.06-1.32)	1.18 (1.06-1.31)
	2016	43,353	37,136	683	1.58 (1.46-1.70)	1.84 (1.70-1.98)	1.12 (1.01-1.25)	1.13 (1.02-1.27)
	2017	44,417	38,068	677	1.52 (1.41-1.64)	1.78 (1.65-1.92)	1.09 (0.97-1.21)	1.10 (0.98-1.23)
	2018	42,586	36,366	596	1.40 (1.29-1.51)	1.64 (1.51-1.78)	1 (Reference)	1 (Reference)
Major bleeding	2014	40,002	33,894	992	2.48 (2.33-2.64)	2.93 (2.75-3.11)	1.17 (1.07-1.28)	1.12 (1.00-1.26)
	2015	43,474	37,049	964	2.22 (2.08-2.36)	2.60 (2.44-2.77)	1.04 (0.95-1.14)	1.07 (0.98-1.17)
	2016	42,799	36,607	924	2.16 (2.02-2.30)	2.52 (2.36-2.69)	1.01 (0.92-1.11)	1.03 (0.94-1.13)
	2017	43,801	37,472	890	2.03 (1.90-2.17)	2.38 (2.22-2.54)	0.95 (0.87-1.04)	0.97 (0.88-1.06)
	2018	42,036	35,801	894	2.13 (1.99-2.27)	2.50 (2.34-2.67)	1 (Reference)	1 (Reference)
ICH	2014	40,419	34,588	238	0.59 (0.52-0.67)	0.69 (0.60-0.78)	1.55 (1.27-1.89)	1.57 (1.20-2.04)
	2015	43,897	37,731	217	0.49 (0.43-0.56)	0.58 (0.50-0.66)	1.29 (1.05-1.58)	1.29 (1.05-1.58)
	2016	43,132	37,217	191	0.44 (0.38-0.51)	0.51 (0.44-0.59)	1.15 (0.94-1.42)	1.17 (0.95-1.44)
	2017	44,173	38,126	160	0.36 (0.31-0.42)	0.42 (0.36-0.49)	0.94 (0.76-1.17)	0.94 (0.76-1.17)
	2018	42,392	36,435	162	0.38 (0.33-0.44)	0.44 (0.38-0.52)	1 (Reference)	1 (Reference)
GI bleeding	2014	40,442	34,438	404	1.00 (0.91-1.10)	1.17 (1.06-1.29)	0.97 (0.84-1.11)	0.93 (0.78-1.11)
	2015	43,931	37,579	425	0.97 (0.88-1.06)	1.13 (1.03-1.24)	0.93 (0.82-1.07)	0.96 (0.84-1.10)
	2016	43,172	37,065	419	0.97 (0.88-1.07)	1.13 (1.02-1.24)	0.93 (0.82-1.07)	0.96 (0.84-1.09)
	2017	44,234	37,966	435	0.98 (0.89-1.08)	1.15 (1.04-1.26)	0.95 (0.83-1.08)	0.96 (0.84-1.09)
	2018	42,415	36,260	439	1.04 (0.94-1.13)	1.21 (1.10-1.33)	1 (Reference)	1 (Reference)
All-cause mortality	2014	40,663	34,807	8,149	20.04 (19.65-20.43)	23.41 (22.91-23.93)	1.06 (1.02-1.09)	1.27 (1.22-1.33)
	2015	44,149	37,947	8,550	19.37 (19.00-19.73)	22.53 (22.06-23.01)	1.02 (0.99-1.05)	1.03 (1.00-1.06)
	2016	43,353	37,408	8,198	18.91 (18.54-19.28)	21.91 (21.44-22.39)	0.99 (0.96-1.02)	1.00 (0.97-1.03)
	2017	44,417	38,330	8,387	18.88 (18.52-19.25)	21.88 (21.42-22.35)	0.99 (0.96-1.02)	1.00 (0.97-1.03)
	2018	42,586	36,608	8,086	18.99 (18.61-19.36)	22.09 (21.61-22.57)	1 (Reference)	1 (Reference)

† Including death caused by the clinical event

‡ Individuals who were primarily admitted to the index hospitalization for the clinical event were excluded.

* Estimated by the cumulative incidence competing risk method, except for all-cause mortality which was estimated by the Kaplan-Meier estimator

§ Adjusted for age, sex, immigration background, standard household income, type of atrial fibrillation, and comorbidities including asthma, chronic obstructive pulmonary disease, other chronic lung diseases, heart failure, myocardial infarction (history), hypertension, other valvular heart disease (except for rheumatic mitral stenosis and mechanical heart valves), peripheral artery disease, liver diseases, gastroesophageal reflux disease, peptic ulcer disease, chronic kidney diseases, anemia, coagulopathy, diabetes, thyroid disease, ischemic stroke (history), transient ischemic attack, other arterial thromboembolism, Parkinson's disease, Alzheimer's disease, autoimmune disease, systemic connective tissue disorders, venous thromboembolism, major bleeding, and malignant tumor. The comorbidities were identified within the prior four years and within index hospitalization respectively, except for venous thromboembolism, which were identified between the prior four years and prior six months and within the prior six months respectively.

Abbreviations: PY, person-year; CI, confidence interval; HR, hazard ratio; ICH, intracranial hemorrhage; GI, gastrointestinal.

© 2023 Chen Q et al. *JAMA Network Open.*

eTable 17. Risk of Clinical Events Within the 1-Year Follow-up After Incident Nonvalvular Atrial Fibrillation Diagnosis, Excluding Patients With Preexisting Chronic Oral Anticoagulant Treatment

Clinical event[†]	Cohort	No. at risk[‡]	Follow-up (days)	Observation time (PYs)	No. events	Cumulative incidence (%, 95%CI)[*]	Incidence rate (per 100 PYs, 95%CI)
Ischemic stroke	2014	40,663	0-30	3,187	234	0.58 (0.51-0.65)	7.34 (6.43-8.34)
			0-90	9,177	383	0.94 (0.85-1.04)	4.17 (3.77-4.61)
			0-180	17,768	503	1.24 (1.13-1.35)	2.83 (2.59-3.09)
			0-270	26,062	609	1.50 (1.38-1.62)	2.34 (2.15-2.53)
			0-365	34,556	717	1.76 (1.64-1.89)	2.07 (1.93-2.23)
	2015	44,149	0-30	3,460	235	0.53 (0.47-0.60)	6.79 (5.95-7.72)
			0-90	9,976	385	0.87 (0.79-0.96)	3.86 (3.48-4.26)
			0-180	19,339	513	1.16 (1.07-1.27)	2.65 (2.43-2.89)
			0-270	28,387	634	1.44 (1.33-1.55)	2.23 (2.06-2.41)
			0-365	37,667	733	1.66 (1.54-1.78)	1.95 (1.81-2.09)
	2016	43,353	0-30	3,395	247	0.57 (0.50-0.64)	7.27 (6.40-8.24)
			0-90	9,801	362	0.84 (0.75-0.92)	3.69 (3.32-4.09)
			0-180	19,026	488	1.13 (1.03-1.23)	2.56 (2.34-2.80)
			0-270	27,963	577	1.33 (1.23-1.44)	2.06 (1.90-2.24)
			0-365	37,136	683	1.58 (1.46-1.70)	1.84 (1.70-1.98)
	2017	44,417	0-30	3,478	211	0.48 (0.41-0.54)	6.07 (5.28-6.94)
			0-90	10,044	325	0.73 (0.66-0.81)	3.24 (2.89-3.61)
			0-180	19,507	453	1.02 (0.93-1.12)	2.32 (2.11-2.55)
			0-270	28,674	563	1.27 (1.17-1.37)	1.96 (1.80-2.13)
			0-365	38,068	677	1.52 (1.41-1.64)	1.78 (1.65-1.92)
	2018	42,586	0-30	3,325	194	0.46 (0.40-0.52)	5.83 (5.04-6.72)
			0-90	9,589	312	0.73 (0.66-0.82)	3.25 (2.90-3.64)
			0-180	18,620	434	1.02 (0.93-1.12)	2.33 (2.12-2.56)
			0-270	27,367	508	1.19 (1.09-1.30)	1.86 (1.70-2.02)
			0-365	36,366	596	1.40 (1.29-1.51)	1.64 (1.51-1.78)
Major bleeding	2014	40,002	0-30	3,139	226	0.56 (0.50-0.64)	7.20 (6.29-8.20)
			0-90	9,027	440	1.10 (1.00-1.21)	4.87 (4.43-5.35)
			0-180	17,457	656	1.64 (1.52-1.77)	3.76 (3.48-4.06)
			0-270	25,583	819	2.05 (1.91-2.19)	3.20 (2.99-3.43)
			0-365	33,894	992	2.48 (2.33-2.64)	2.93 (2.75-3.11)
	2015	43,474	0-30	3,412	253	0.58 (0.51-0.66)	7.42 (6.53-8.39)
			0-90	9,829	448	1.03 (0.94-1.13)	4.56 (4.15-5.00)
			0-180	19,039	637	1.47 (1.36-1.58)	3.35 (3.09-3.62)
			0-270	27,935	796	1.83 (1.71-1.96)	2.85 (2.65-3.05)
			0-365	37,049	964	2.22 (2.08-2.36)	2.60 (2.44-2.77)
	2016	42,799	0-30	3,358	215	0.50 (0.44-0.57)	6.40 (5.58-7.32)
			0-90	9,684	411	0.96 (0.87-1.06)	4.24 (3.84-4.67)
			0-180	18,782	614	1.43 (1.33-1.55)	3.27 (3.02-3.54)
			0-270	27,583	768	1.79 (1.67-1.92)	2.78 (2.59-2.99)
			0-365	36,607	924	2.16 (2.02-2.30)	2.52 (2.36-2.69)
	2017	43,801	0-30	3,434	197	0.45 (0.39-0.52)	5.74 (4.96-6.60)
			0-90	9,906	401	0.92 (0.83-1.01)	4.05 (3.66-4.46)
			0-180	19,220	570	1.30 (1.20-1.41)	2.97 (2.73-3.22)
			0-270	28,239	724	1.65 (1.54-1.78)	2.56 (2.38-2.76)
			0-365	37,472	890	2.03 (1.90-2.17)	2.38 (2.22-2.54)
	2018	42,036	0-30	3,284	230	0.55 (0.48-0.62)	7.00 (6.13-7.97)
			0-90	9,464	404	0.96 (0.87-1.06)	4.27 (3.86-4.71)
			0-180	18,363	588	1.40 (1.29-1.51)	3.20 (2.95-3.47)
			0-270	26,965	754	1.79 (1.67-1.92)	2.80 (2.60-3.00)
			0-365	35,801	894	2.13 (1.99-2.27)	2.50 (2.34-2.67)
ICH	2014	40,419	0-30	3,177	48	0.12 (0.09-0.16)	1.51 (1.11-2.00)
			0-90	9,161	97	0.24 (0.20-0.29)	1.06 (0.86-1.29)
			0-180	17,755	153	0.38 (0.32-0.44)	0.86 (0.73-1.01)

© 2023 Chen Q et al. *JAMA Network Open.*

			0-270	26,065	187	0.46 (0.40-0.53)	0.72 (0.62-0.83)
			0-365	34,588	238	0.59 (0.52-0.67)	0.69 (0.60-0.78)
	2015	43,897	0-30	3,451	46	0.10 (0.08-0.14)	1.33 (0.98-1.78)
			0-90	9,963	83	0.19 (0.15-0.23)	0.83 (0.66-1.03)
			0-180	19,337	119	0.27 (0.23-0.32)	0.62 (0.51-0.74)
			0-270	28,412	174	0.40 (0.34-0.46)	0.61 (0.52-0.71)
			0-365	37,731	217	0.49 (0.43-0.56)	0.58 (0.50-0.66)
	2016	43,132	0-30	3,390	34	0.08 (0.06-0.11)	1.00 (0.69-1.40)
			0-90	9,797	69	0.16 (0.13-0.20)	0.70 (0.55-0.89)
			0-180	19,040	111	0.26 (0.21-0.31)	0.58 (0.48-0.70)
			0-270	28,005	152	0.35 (0.30-0.41)	0.54 (0.46-0.64)
			0-365	37,217	191	0.44 (0.38-0.51)	0.51 (0.44-0.59)
	2017	44,173	0-30	3,469	34	0.08 (0.05-0.11)	0.98 (0.68-1.37)
			0-90	10,031	72	0.16 (0.13-0.20)	0.72 (0.56-0.90)
			0-180	19,499	104	0.24 (0.19-0.28)	0.53 (0.44-0.65)
			0-270	28,690	133	0.30 (0.25-0.36)	0.46 (0.39-0.55)
			0-365	38,126	160	0.36 (0.31-0.42)	0.42 (0.36-0.49)
	2018	42,392	0-30	3,318	27	0.06 (0.04-0.09)	0.81 (0.54-1.18)
			0-90	9,583	51	0.12 (0.09-0.16)	0.53 (0.40-0.70)
			0-180	18,630	89	0.21 (0.17-0.26)	0.48 (0.38-0.59)
			0-270	27,402	132	0.31 (0.26-0.37)	0.48 (0.40-0.57)
			0-365	36,435	162	0.38 (0.33-0.44)	0.44 (0.38-0.52)
GI bleeding	2014	40,442	0-30	3,173	104	0.26 (0.21-0.31)	3.28 (2.68-3.97)
			0-90	9,138	186	0.46 (0.40-0.53)	2.04 (1.75-2.35)
			0-180	17,699	275	0.68 (0.60-0.76)	1.55 (1.38-1.75)
			0-270	25,966	338	0.84 (0.75-0.93)	1.30 (1.17-1.45)
			0-365	34,438	404	1.00 (0.91-1.10)	1.17 (1.06-1.29)
	2015	43,931	0-30	3,448	128	0.29 (0.24-0.35)	3.71 (3.10-4.41)
			0-90	9,944	211	0.48 (0.42-0.55)	2.12 (1.85-2.43)
			0-180	19,282	298	0.68 (0.60-0.76)	1.55 (1.37-1.73)
			0-270	28,312	350	0.80 (0.72-0.88)	1.24 (1.11-1.37)
			0-365	37,579	425	0.97 (0.88-1.06)	1.13 (1.03-1.24)
	2016	43,172	0-30	3,387	105	0.24 (0.20-0.29)	3.10 (2.54-3.75)
			0-90	9,778	202	0.47 (0.41-0.54)	2.07 (1.79-2.37)
			0-180	18,983	301	0.70 (0.62-0.78)	1.59 (1.41-1.78)
			0-270	27,903	360	0.83 (0.75-0.92)	1.29 (1.16-1.43)
			0-365	37,065	419	0.97 (0.88-1.07)	1.13 (1.02-1.24)
	2017	44,234	0-30	3,468	101	0.23 (0.19-0.28)	2.91 (2.37-3.54)
			0-90	10,013	201	0.45 (0.40-0.52)	2.01 (1.74-2.30)
			0-180	19,447	282	0.64 (0.57-0.72)	1.45 (1.29-1.63)
			0-270	28,591	358	0.81 (0.73-0.90)	1.25 (1.13-1.39)
			0-365	37,966	435	0.98 (0.89-1.08)	1.15 (1.04-1.26)
	2018	42,415	0-30	3,315	128	0.30 (0.25-0.36)	3.86 (3.22-4.59)
			0-90	9,559	224	0.53 (0.46-0.60)	2.34 (2.05-2.67)
			0-180	18,563	302	0.71 (0.64-0.80)	1.63 (1.45-1.82)
			0-270	27,286	372	0.88 (0.79-0.97)	1.36 (1.23-1.51)
			0-365	36,260	439	1.04 (0.94-1.13)	1.21 (1.10-1.33)
All-cause mortality	2014	40,663	0-30	3,194	2,956	7.27 (7.02-7.52)	92.54 (89.23-95.94)
			0-90	9,211	4,839	11.90 (11.58-12.21)	52.54 (51.07-54.04)
			0-180	17,859	6,225	15.31 (14.96-15.66)	34.86 (34.00-35.73)
			0-270	26,224	7,193	17.69 (17.32-18.06)	27.43 (26.80-28.07)
			0-365	34,807	8,149	20.04 (19.65-20.43)	23.41 (22.91-23.93)
	2015	44,149	0-30	3,469	3,209	7.27 (7.03-7.51)	92.51 (89.34-95.77)
			0-90	10,016	5,095	11.54 (11.24-11.84)	50.87 (49.48-52.28)
			0-180	19,442	6,569	14.88 (14.55-15.21)	33.79 (32.98-34.61)
			0-270	28,570	7,618	17.26 (16.90-17.61)	26.66 (26.07-27.27)
			0-365	37,947	8,550	19.37 (19.00-19.73)	22.53 (22.06-23.01)
	2016	43,353	0-30	3,404	3,137	7.24 (6.99-7.48)	92.15 (88.95-95.43)

© 2023 Chen Q et al. *JAMA Network Open.*

			0-90	9,840	4,967	11.46 (11.16-11.76)	50.48 (49.08-51.90)
			0-180	19,128	6,278	14.48 (14.15-14.81)	32.82 (32.01-33.64)
			0-270	28,141	7,260	16.75 (16.39-17.10)	25.80 (25.21-26.40)
			0-365	37,408	8,198	18.91 (18.54-19.28)	21.91 (21.44-22.39)
	2017	44,417	0-30	3,486	3,259	7.34 (7.09-7.58)	93.48 (90.30-96.75)
			0-90	10,080	5,054	11.38 (11.08-11.67)	50.14 (48.76-51.54)
			0-180	19,600	6,404	14.42 (14.09-14.74)	32.67 (31.88-33.48)
			0-270	28,840	7,435	16.74 (16.39-17.09)	25.78 (25.20-26.37)
			0-365	38,330	8,387	18.88 (18.52-19.25)	21.88 (21.42-22.35)
	2018	42,586	0-30	3,332	3,288	7.72 (7.47-7.97)	98.69 (95.34-102.12)
			0-90	9,622	5,033	11.82 (11.51-12.12)	52.31 (50.87-53.78)
			0-180	18,708	6,330	14.86 (14.53-15.20)	33.84 (33.01-34.68)
			0-270	27,524	7,255	17.04 (16.68-17.39)	26.36 (25.76-26.97)
			0-365	36,608	8,086	18.99 (18.61-19.36)	22.09 (21.61-22.57)

† Including death caused by the clinical event

‡ Individuals who were primarily admitted to the index hospitalization for the clinical event were excluded.

* Estimated by the cumulative incidence competing risk method, except for all-cause mortality which was estimated by the Kaplan-Meier estimator

Abbreviations: PY, person-year; CI, confidence interval; ICH, intracranial haemorrhage; GI, gastrointestinal.

© 2023 Chen Q et al. *JAMA Network Open.*

eTable 18. Hazard Ratios of Prognosis Within 1 Year After Incident Nonvalvular Atrial Fibrillation Diagnosis Between Cohorts According to Other Adjustment Models

Clinical event†	Cohort	HR (95%CI)‡				
		Model 1	Model 2	Model 3	Model 4	Model 5
Ischemic stroke	2014	1.16 (1.05-1.27)	1.28 (1.13-1.45)	1.27 (1.12-1.44)	1.24 (1.09-1.40)	1.27 (1.12-1.44)
	2015	1.17 (1.06-1.28)	1.17 (1.07-1.28)	1.16 (1.06-1.27)	1.14 (1.04-1.25)	1.16 (1.06-1.27)
	2016	1.09 (0.99-1.20)	1.09 (0.99-1.20)	1.09 (0.99-1.20)	1.07 (0.97-1.18)	1.09 (1.00-1.20)
	2017	1.05 (0.96-1.16)	1.06 (0.96-1.16)	1.05 (0.96-1.15)	1.04 (0.95-1.15)	1.05 (0.96-1.15)
	2018	1 (Reference)	1 (Reference)	1 (Reference)	1 (Reference)	1 (Reference)
Major bleeding	2014	1.20 (1.12-1.30)	1.14 (1.03-1.26)	1.12 (1.01-1.24)	1.08 (0.98-1.20)	1.16 (1.05-1.28)
	2015	1.07 (0.99-1.16)	1.08 (1.00-1.17)	1.07 (0.99-1.15)	1.04 (0.96-1.12)	1.10 (1.01-1.18)
	2016	1.03 (0.96-1.12)	1.04 (0.96-1.12)	1.03 (0.95-1.11)	1.01 (0.93-1.09)	1.05 (0.97-1.14)
	2017	0.98 (0.90-1.06)	0.98 (0.91-1.06)	0.98 (0.90-1.06)	0.97 (0.89-1.05)	0.98 (0.91-1.06)
	2018	1 (Reference)	1 (Reference)	1 (Reference)	1 (Reference)	1 (Reference)
ICH	2014	1.48 (1.25-1.74)	1.62 (1.29-2.03)	1.59 (1.27-1.99)	1.51 (1.20-1.89)	1.65 (1.31-2.06)
	2015	1.27 (1.08-1.50)	1.28 (1.08-1.51)	1.26 (1.07-1.49)	1.21 (1.02-1.43)	1.29 (1.09-1.52)
	2016	1.08 (0.91-1.28)	1.08 (0.91-1.29)	1.07 (0.90-1.28)	1.04 (0.87-1.24)	1.10 (0.92-1.30)
	2017	0.89 (0.74-1.07)	0.89 (0.75-1.07)	0.89 (0.74-1.07)	0.88 (0.73-1.05)	0.90 (0.75-1.07)
	2018	1 (Reference)	1 (Reference)	1 (Reference)	1 (Reference)	1 (Reference)
GI bleeding	2014	0.98 (0.88-1.10)	0.90 (0.78-1.05)	0.89 (0.76-1.03)	0.88 (0.76-1.02)	0.91 (0.78-1.05)
	2015	0.93 (0.83-1.05)	0.94 (0.84-1.05)	0.93 (0.83-1.04)	0.92 (0.82-1.03)	0.95 (0.85-1.07)
	2016	0.95 (0.84-1.06)	0.94 (0.84-1.06)	0.94 (0.84-1.05)	0.93 (0.83-1.05)	0.96 (0.85-1.07)
	2017	0.99 (0.88-1.10)	0.99 (0.88-1.10)	0.98 (0.88-1.10)	0.98 (0.88-1.10)	0.98 (0.88-1.10)
	2018	1 (Reference)	1 (Reference)	1 (Reference)	1 (Reference)	1 (Reference)
All-cause mortality	2014	1.06 (1.04-1.09)	1.30 (1.25-1.34)	1.29 (1.24-1.34)	1.22 (1.17-1.26)	1.24 (1.20-1.29)
	2015	1.02 (1.00-1.05)	1.02 (0.99-1.05)	1.02 (0.99-1.04)	0.97 (0.95-1.00)	1.02 (0.99-1.05)
	2016	1.00 (0.98-1.03)	1.00 (0.97-1.03)	1.00 (0.97-1.02)	0.96 (0.93-0.98)	0.99 (0.97-1.02)
	2017	1.00 (0.97-1.03)	1.00 (0.97-1.03)	1.00 (0.97-1.02)	0.98 (0.95-1.00)	0.99 (0.96-1.02)
	2018	1 (Reference)	1 (Reference)	1 (Reference)	1 (Reference)	1 (Reference)

† Including death caused by the clinical event

‡ Model 1 was adjusted for age and sex; Model 2 was adjusted for model 1, immigration background, standard household income, and type of atrial fibrillation;

Model 3 was adjusted for model 2, CHA_2DS_2-VASc score, and HAS-BLED score; Model 4 was adjusted for model 2, and pre-existing chronic use of antithrombotic agents including vitamin K antagonist, direct oral anticoagulant, heparin group, and antiplatelet agent; Model 5 was adjusted for model 2, and comorbidities including asthma, chronic obstructive pulmonary disease, other chronic lung diseases, heart failure, myocardial infarction (history), hypertension, other valvular heart disease (except for rheumatic mitral stenosis and mechanical heart valves), peripheral artery disease, liver diseases, gastroesophageal reflux disease, peptic ulcer disease, chronic kidney diseases, anaemia, coagulopathy, diabetes, thyroid disease, ischemic stroke (history), transient ischemic attack, other arterial thromboembolism, Parkinson's disease, Alzheimer's disease, autoimmune disease, systemic connective tissue disorders, venous thromboembolism, major bleeding, and malignant tumour. The comorbidities were identified within four years prior to the index hospitalization (inclusive).

Abbreviations: HR, hazard ratio; CI, confidence interval; ICH, intracranial haemorrhage; GI, gastrointestinal.

© 2023 Chen Q et al. *JAMA Network Open.*

eTable 19. Hazard Ratios of Prognosis Within 1 Year After Incident Nonvalvular Atrial Fibrillation Diagnosis Between Cohorts According to Other Adjustment Models, Excluding Patients With Preexisting Chronic Oral Anticoagulant Treatment

Clinical event†	Cohort	HR (95%CI)‡				
		Model 1	Model 2	Model 3	Model 4	Model 5
Ischemic stroke	2014	1.23 (1.10-1.37)	1.30 (1.13-1.50)	1.31 (1.13-1.51)	1.30 (1.13-1.50)	1.29 (1.12-1.49)
	2015	1.16 (1.04-1.29)	1.17 (1.05-1.30)	1.16 (1.04-1.30)	1.17 (1.05-1.30)	1.16 (1.04-1.29)
	2016	1.11 (0.99-1.24)	1.11 (1.00-1.24)	1.11 (1.00-1.24)	1.11 (1.00-1.24)	1.11 (0.99-1.24)
	2017	1.08 (0.97-1.20)	1.08 (0.97-1.21)	1.08 (0.97-1.20)	1.08 (0.97-1.21)	1.07 (0.96-1.19)
	2018	1 (Reference)	1 (Reference)	1 (Reference)	1 (Reference)	1 (Reference)
Major bleeding	2014	1.15 (1.05-1.26)	1.07 (0.95-1.20)	1.05 (0.93-1.18)	1.07 (0.95-1.20)	1.08 (0.96-1.22)
	2015	1.03 (0.94-1.13)	1.04 (0.95-1.14)	1.03 (0.94-1.13)	1.04 (0.95-1.14)	1.06 (0.97-1.16)
	2016	1.01 (0.92-1.10)	1.01 (0.92-1.11)	1.01 (0.92-1.10)	1.01 (0.92-1.11)	1.02 (0.93-1.12)
	2017	0.95 (0.86-1.04)	0.95 (0.87-1.04)	0.95 (0.86-1.04)	0.95 (0.87-1.04)	0.95 (0.87-1.04)
	2018	1 (Reference)	1 (Reference)	1 (Reference)	1 (Reference)	1 (Reference)
ICH	2014	1.51 (1.24-1.85)	1.53 (1.18-2.00)	1.51 (1.16-1.96)	1.53 (1.18-2.00)	1.55 (1.19-2.02)
	2015	1.27 (1.04-1.56)	1.28 (1.04-1.56)	1.26 (1.03-1.55)	1.27 (1.04-1.56)	1.29 (1.05-1.58)
	2016	1.15 (0.93-1.41)	1.15 (0.93-1.42)	1.14 (0.93-1.41)	1.15 (0.93-1.42)	1.16 (0.94-1.43)
	2017	0.94 (0.75-1.17)	0.94 (0.76-1.17)	0.94 (0.75-1.17)	0.94 (0.76-1.17)	0.94 (0.75-1.17)
	2018	1 (Reference)	1 (Reference)	1 (Reference)	1 (Reference)	1 (Reference)
GI bleeding	2014	0.95 (0.83-1.09)	0.89 (0.75-1.06)	0.88 (0.74-1.04)	0.89 (0.75-1.06)	0.91 (0.76-1.08)
	2015	0.92 (0.81-1.06)	0.93 (0.82-1.07)	0.92 (0.81-1.05)	0.93 (0.82-1.07)	0.95 (0.83-1.09)
	2016	0.93 (0.81-1.06)	0.93 (0.81-1.06)	0.92 (0.81-1.06)	0.93 (0.81-1.06)	0.94 (0.83-1.08)
	2017	0.94 (0.83-1.08)	0.95 (0.83-1.08)	0.94 (0.82-1.08)	0.94 (0.83-1.08)	0.94 (0.83-1.08)
	2018	1 (Reference)	1 (Reference)	1 (Reference)	1 (Reference)	1 (Reference)
All-cause mortality	2014	1.02 (0.99-1.06)	1.29 (1.23-1.34)	1.28 (1.23-1.34)	1.28 (1.23-1.34)	1.23 (1.18-1.29)
	2015	1.00 (0.97-1.03)	1.00 (0.97-1.03)	1.00 (0.97-1.03)	1.00 (0.97-1.03)	1.01 (0.98-1.05)
	2016	0.98 (0.95-1.01)	0.98 (0.95-1.01)	0.98 (0.95-1.01)	0.98 (0.95-1.01)	0.98 (0.95-1.01)
	2017	0.98 (0.95-1.01)	0.99 (0.96-1.02)	0.99 (0.96-1.02)	0.99 (0.96-1.02)	0.98 (0.95-1.01)
	2018	1 (Reference)	1 (Reference)	1 (Reference)	1 (Reference)	1 (Reference)

† Including death caused by the clinical event

‡ Model 1 was adjusted for age and sex; Model 2 was adjusted for model 1, immigration background, standard household income, and type of atrial fibrillation;
Model 3 was adjusted for model 2, CHA_2DS_2-VASc score, and HAS-BLED score; Model 4 was adjusted for model 2, and pre-existing chronic use of antithrombotic agents including vitamin K antagonist, direct oral anticoagulant, heparin group, and antiplatelet agent; Model 5 was adjusted for model 2, and comorbidities including asthma, chronic obstructive pulmonary disease, other chronic lung diseases, heart failure, myocardial infarction (history), hypertension, other valvular heart disease (except for rheumatic mitral stenosis and mechanical heart valves), peripheral artery disease, liver diseases, gastroesophageal reflux disease, peptic ulcer disease, chronic kidney diseases, anaemia, coagulopathy, diabetes, thyroid disease, ischemic stroke (history), transient ischemic attack, other arterial thromboembolism, Parkinson's disease, Alzheimer's disease, autoimmune disease, systemic connective tissue disorders, venous thromboembolism, major bleeding, and malignant tumour. The comorbidities were identified within four years prior to the index hospitalization (inclusive).

Abbreviations: HR, hazard ratio; CI, confidence interval; ICH, intracranial haemorrhage; GI, gastrointestinal.

© 2023 Chen Q et al. *JAMA Network Open.*

eTable 20. Subgroup Analysis of Risk of Ischemic Stroke Within the 1-Year Follow-up After Incident Nonvalvular Atrial Fibrillation Diagnosis

Subgroup	Cohort	2014	2015	2016	2017	2018
Sex						
Male	Events/At risk	427/31,391	471/34,046	445/33,761	453/35,304	426/35,246
	**CumI†**	**1.36 (1.24-1.49)**	**1.38 (1.26-1.51)**	**1.32 (1.20-1.44)**	**1.28 (1.17-1.40)**	**1.21 (1.10-1.33)**
	IR‡	1.57 (1.42-1.72)	1.58 (1.44-1.73)	1.51 (1.37-1.65)	1.46 (1.33-1.60)	1.38 (1.25-1.52)
Female	Events/At risk	482/24,489	532/27,271	467/26,257	464/27,086	431/26,450
	**CumI†**	**1.97 (1.80-2.15)**	**1.95 (1.79-2.12)**	**1.78 (1.62-1.94)**	**1.71 (1.56-1.87)**	**1.63 (1.48-1.79)**
	IR‡	2.33 (2.13-2.55)	2.30 (2.11-2.50)	2.08 (1.90-2.28)	2.01 (1.83-2.21)	1.91 (1.74-2.10)
Immigration background						
Native Dutch	Events/At risk	786/49,197	878/53,983	793/52,449	800/54,543	748/53,914
	**CumI†**	**1.60 (1.49-1.71)**	**1.63 (1.52-1.74)**	**1.51 (1.41-1.62)**	**1.47 (1.37-1.57)**	**1.39 (1.29-1.49)**
	IR‡	1.86 (1.74-2.00)	1.89 (1.77-2.02)	1.75 (1.63-1.88)	1.69 (1.58-1.81)	1.61 (1.49-1.73)
First/Second-generation immigrants	Events/At risk	123/6,683	125/7,334	119/7,569	117/7,847	109/7,782
	**CumI†**	**1.84 (1.54-2.18)**	**1.70 (1.43-2.02)**	**1.57 (1.31-1.87)**	**1.49 (1.24-1.78)**	**1.40 (1.16-1.68)**
	IR‡	2.13 (1.77-2.54)	1.96 (1.63-2.33)	1.79 (1.49-2.15)	1.71 (1.41-2.05)	1.59 (1.31-1.92)
CHA_2DS_2-VASc score						
0	Events/At risk	15/4,038	13/4,787	24/4,790	17/4,760	22/4,589
	**CumI†**	**0.37 (0.22-0.60)**	**0.27 (0.15-0.46)**	**0.50 (0.33-0.74)**	**0.36 (0.22-0.56)**	**0.48 (0.31-0.72)**
	IR‡	0.38 (0.21-0.63)	0.28 (0.15-0.48)	0.52 (0.33-0.77)	0.37 (0.21-0.59)	0.49 (0.31-0.75)
1	Events/At risk	41/7,422	47/8,309	47/8,160	63/8,577	44/8,565
	**CumI†**	**0.55 (0.40-0.74)**	**0.57 (0.42-0.75)**	**0.58 (0.43-0.76)**	**0.73 (0.57-0.93)**	**0.51 (0.38-0.68)**
	IR‡	0.58 (0.42-0.79)	0.60 (0.44-0.79)	0.61 (0.45-0.81)	0.77 (0.59-0.99)	0.54 (0.39-0.73)
≥2	Events/At risk	853/44,420	943/48,221	841/47,068	837/49,053	791/48,542
	**CumI†**	**1.92 (1.80-2.05)**	**1.96 (1.83-2.08)**	**1.79 (1.67-1.91)**	**1.71 (1.59-1.82)**	**1.63 (1.52-1.75)**
	IR‡	2.31 (2.15-2.47)	2.34 (2.19-2.49)	2.12 (1.98-2.27)	2.03 (1.89-2.17)	1.94 (1.81-2.08)
HAS-BLED score						
0	Events/At risk	21/5,034	21/6,083	28/6,227	24/6,506	28/6,531
	**CumI†**	**0.42 (0.27-0.63)**	**0.35 (0.22-0.52)**	**0.45 (0.31-0.64)**	**0.37 (0.24-0.54)**	**0.43 (0.29-0.61)**
	IR‡	0.43 (0.27-0.66)	0.36 (0.22-0.55)	0.46 (0.31-0.67)	0.38 (0.24-0.56)	0.44 (0.29-0.64)
1	Events/At risk	195/17,923	217/20,476	185/20,433	243/21,426	209/21,440
	**CumI†**	**1.09 (0.94-1.25)**	**1.06 (0.93-1.21)**	**0.91 (0.78-1.04)**	**1.13 (1.00-1.28)**	**0.97 (0.85-1.11)**
	IR‡	1.23 (1.06-1.41)	1.19 (1.04-1.36)	1.01 (0.87-1.17)	1.27 (1.11-1.44)	1.09 (0.95-1.25)
2	Events/At risk	339/20,011	364/21,706	375/20,811	333/21,490	310/21,309
	**CumI†**	**1.69 (1.52-1.88)**	**1.68 (1.51-1.85)**	**1.80 (1.63-1.99)**	**1.55 (1.39-1.72)**	**1.45 (1.30-1.62)**
	IR‡	1.99 (1.79-2.22)	1.96 (1.76-2.17)	2.10 (1.90-2.33)	1.81 (1.62-2.01)	1.71 (1.52-1.91)
≥3	Events/At risk	354/12,912	401/13,052	324/12,547	317/12,968	310/12,416

© 2023 Chen Q et al. *JAMA Network Open.*

	**CumI†**	**2.74 (2.47-3.03)**	**3.07 (2.79-3.38)**	**2.58 (2.32-2.87)**	**2.44 (2.19-2.72)**	**2.50 (2.23-2.78)**
	IR‡	3.49 (3.14-3.88)	3.93 (3.56-4.34)	3.30 (2.95-3.68)	3.13 (2.79-3.49)	3.19 (2.84-3.56)
Use of antithrombotic agents						
Pre-existing chronic use of antiplatelet agents*	Events/At risk	303/16,060	356/16,304	307/15,790	291/16,683	272/16,264
	**CumI†**	**1.89 (1.68-2.11)**	**2.18 (1.97-2.42)**	**1.94 (1.74-2.17)**	**1.74 (1.55-1.95)**	**1.67 (1.48-1.88)**
	IR‡	2.23 (1.98-2.49)	2.58 (2.32-2.87)	2.30 (2.05-2.58)	2.05 (1.82-2.30)	1.97 (1.75-2.22)
≥1 oral anticoagulant prescription within the 1-year follow-up	Events/At risk	559/37,905	667/44,426	624/43,767	632/45,144	589/44,881
	**CumI†**	**1.47 (1.36-1.60)**	**1.50 (1.39-1.62)**	**1.43 (1.32-1.54)**	**1.40 (1.29-1.51)**	**1.31 (1.21-1.42)**
	IR‡	1.58 (1.45-1.71)	1.60 (1.48-1.72)	1.52 (1.40-1.64)	1.49 (1.38-1.61)	1.40 (1.29-1.52)
Comorbidities (or medical history)§						
Asthma	Events/At risk	10/687	13/837	<10/791‖‖	14/911	15/1,046
	**CumI†**	**1.46 (0.75-2.58)**	**1.55 (0.87-2.58)**	**0.63 (0.24-1.41)**	**1.54 (0.88-2.51)**	**1.43 (0.84-2.30)**
	IR‡	1.65 (0.79-3.03)	1.77 (0.94-3.02)	0.71 (0.23-1.67)	1.76 (0.96-2.95)	1.66 (0.93-2.73)
Chronic obstructive pulmonary disease	Events/At risk	80/5,837	115/6,073	85/5,713	77/5,718	70/5,684
	**CumI†**	**1.37 (1.10-1.69)**	**1.89 (1.57-2.26)**	**1.49 (1.20-1.83)**	**1.35 (1.07-1.67)**	**1.23 (0.97-1.55)**
	IR‡	1.79 (1.42-2.23)	2.51 (2.07-3.01)	1.94 (1.55-2.40)	1.77 (1.40-2.22)	1.61 (1.26-2.04)
Other chronic lung disease	Events/At risk	28/748	27/835	27/811	28/973	32/1,116
	**CumI†**	**3.74 (2.55-5.28)**	**3.23 (2.18-4.60)**	**3.33 (2.25-4.73)**	**2.88 (1.96-4.07)**	**2.87 (2.00-3.97)**
	IR‡	5.88 (3.91-8.50)	5.25 (3.46-7.63)	5.33 (3.51-7.76)	4.65 (3.09-6.72)	4.67 (3.20-6.59)
Heart failure	Events/At risk	183/9,984	193/10,705	182/10,407	183/10,882	176/11,043
	**CumI†**	**1.83 (1.58-2.11)**	**1.80 (1.56-2.07)**	**1.75 (1.51-2.01)**	**1.68 (1.45-1.94)**	**1.59 (1.37-1.84)**
	IR‡	2.49 (2.15-2.88)	2.43 (2.10-2.80)	2.36 (2.03-2.73)	2.26 (1.94-2.61)	2.15 (1.84-2.49)
Myocardial infarction (history)	Events/At risk	40/2,552	48/2,899	48/2,997	44/3,190	45/3,216
	**CumI†**	**1.57 (1.14-2.11)**	**1.66 (1.24-2.17)**	**1.60 (1.20-2.10)**	**1.38 (1.02-1.83)**	**1.40 (1.04-1.85)**
	IR‡	1.90 (1.35-2.58)	2.00 (1.48-2.66)	1.92 (1.42-2.55)	1.64 (1.19-2.20)	1.65 (1.20-2.21)
Hypertension	Events/At risk	335/16,265	362/17,925	328/17,379	347/18,579	322/19,113
	**CumI†**	**2.06 (1.85-2.29)**	**2.02 (1.82-2.23)**	**1.89 (1.69-2.10)**	**1.87 (1.68-2.07)**	**1.68 (1.51-1.87)**
	IR‡	2.43 (2.17-2.70)	2.37 (2.13-2.63)	2.19 (1.96-2.44)	2.19 (1.97-2.43)	1.98 (1.77-2.20)
Other valvular heart disease‖	Events/At risk	108/6,639	122/7,057	115/6,653	119/6,875	99/6,721
	**CumI†**	**1.63 (1.34-1.95)**	**1.73 (1.44-2.05)**	**1.73 (1.44-2.06)**	**1.73 (1.44-2.06)**	**1.47 (1.21-1.78)**
	IR‡	1.98 (1.62-2.39)	2.12 (1.76-2.54)	2.11 (1.74-2.53)	2.11 (1.75-2.52)	1.80 (1.46-2.19)
Peripheral artery disease	Events/At risk	23/1,025	32/1,212	22/994	21/1,023	17/986
	**CumI†**	**2.24 (1.46-3.29)**	**2.64 (1.84-3.66)**	**2.21 (1.43-3.27)**	**2.05 (1.31-3.07)**	**1.72 (1.05-2.69)**
	IR‡	2.82 (1.79-4.23)	3.58 (2.45-5.06)	2.88 (1.80-4.36)	2.67 (1.66-4.09)	2.28 (1.33-3.66)
Liver diseases	Events/At risk	16/1,052	24/1,058	15/1,037	10/1,051	10/1,036
	**CumI†**	**1.52 (0.91-2.41)**	**2.27 (1.49-3.30)**	**1.45 (0.85-2.32)**	**0.95 (0.49-1.70)**	**0.97 (0.50-1.72)**
	IR‡	2.18 (1.25-3.55)	3.31 (2.12-4.93)	2.11 (1.18-3.48)	1.40 (0.67-2.58)	1.49 (0.72-2.75)

© 2023 Chen Q et al. *JAMA Network Open.*

Gastroesophageal reflux disease	Events/At risk	<10/117[‖]	<10/92[‖]	<10/114[‖]	<10/127[‖]	<10/111[‖]
	**CumI[†]**	**3.42 (1.11-7.93)**	**1.09 (0.09-5.34)**	**1.75 (0.34-5.65)**	**1.57 (0.31-5.10)**	**2.70 (0.73-7.09)**
	IR[‡]	4.01 (1.09-10.27)	1.34 (0.03-7.45)	2.18 (0.26-7.87)	1.88 (0.23-6.81)	3.54 (0.73-10.33)
Peptic ulcer disease	Events/At risk	<10/239[‖]	<10/244[‖]	<10/212[‖]	<10/226[‖]	<10/235[‖]
	**CumI[†]**	**3.35 (1.57-6.21)**	**1.64 (0.55-3.90)**	**1.89 (0.63-4.48)**	**0.88 (0.18-2.94)**	**1.70 (0.57-4.05)**
	IR[‡]	4.60 (1.98-9.06)	2.21 (0.60-5.65)	2.69 (0.73-6.90)	1.18 (0.14-4.25)	2.23 (0.61-5.72)
Chronic kidney diseases	Events/At risk	122/5,808	118/6,304	131/6,516	131/6,896	116/6,585
	**CumI[†]**	**2.10 (1.76-2.49)**	**1.87 (1.56-2.23)**	**2.01 (1.69-2.37)**	**1.90 (1.60-2.24)**	**1.76 (1.46-2.10)**
	IR[‡]	2.98 (2.48-3.56)	2.64 (2.19-3.17)	2.80 (2.34-3.32)	2.63 (2.20-3.12)	2.47 (2.04-2.96)
Anaemia	Events/At risk	60/3,527	74/3,868	80/4,015	75/4,390	83/4,546
	**CumI[†]**	**1.70 (1.31-2.17)**	**1.91 (1.52-2.38)**	**1.99 (1.59-2.46)**	**1.71 (1.36-2.13)**	**1.83 (1.47-2.25)**
	IR[‡]	2.28 (1.74-2.93)	2.60 (2.04-3.26)	2.71 (2.15-3.37)	2.33 (1.83-2.92)	2.50 (1.99-3.10)
Coagulopathy	Events/At risk	14/749	19/1,036	18/982	18/1,044	33/1,179
	**CumI[†]**	**1.87 (1.07-3.04)**	**1.83 (1.14-2.80)**	**1.83 (1.13-2.83)**	**1.72 (1.06-2.66)**	**2.80 (1.97-3.86)**
	IR[‡]	2.64 (1.44-4.43)	2.53 (1.53-3.96)	2.64 (1.57-4.18)	2.55 (1.51-4.02)	4.13 (2.84-5.79)
Diabetes	Events/At risk	196/9,242	246/10,024	221/9,944	195/10,308	190/10,341
	**CumI[†]**	**2.12 (1.84-2.43)**	**2.45 (2.16-2.77)**	**2.22 (1.95-2.53)**	**1.89 (1.64-2.17)**	**1.84 (1.59-2.11)**
	IR[‡]	2.62 (2.27-3.02)	3.03 (2.66-3.43)	2.74 (2.39-3.13)	2.34 (2.02-2.69)	2.29 (1.98-2.64)
Thyroid disease	Events/At risk	17/1,408	23/1,504	29/1,409	19/1,580	24/1,570
	**CumI[†]**	**1.21 (0.73-1.89)**	**1.53 (1.00-2.25)**	**2.06 (1.41-2.90)**	**1.20 (0.75-1.84)**	**1.53 (1.01-2.23)**
	IR[‡]	1.44 (0.84-2.30)	1.82 (1.16-2.74)	2.41 (1.61-3.46)	1.41 (0.85-2.20)	1.85 (1.18-2.75)
Ischemic stroke (history)[#]	Events/At risk	75/1,613	82/1,759	75/1,687	72/1,792	72/1,685
	**CumI[†]**	**4.65 (3.70-5.76)**	**4.66 (3.75-5.72)**	**4.45 (3.53-5.50)**	**4.02 (3.18-5.00)**	**4.27 (3.38-5.31)**
	IR[‡]	5.96 (4.69-7.48)	5.89 (4.69-7.31)	5.66 (4.45-7.10)	5.08 (3.97-6.40)	5.33 (4.17-6.71)
Transient ischemic attack[#]	Events/At risk	33/793	41/832	23/804	32/860	26/778
	**CumI[†]**	**4.16 (2.93-5.72)**	**4.93 (3.60-6.55)**	**2.86 (1.87-4.19)**	**3.72 (2.60-5.14)**	**3.34 (2.24-4.78)**
	IR[‡]	5.00 (3.44-7.02)	5.77 (4.14-7.83)	3.39 (2.15-5.08)	4.25 (2.91-6.00)	3.92 (2.56-5.74)
Other arterial thromboembolism[#]	Events/At risk	11/593	13/541	<10/509[‖]	10/474	<10/398[‖]
	**CumI[†]**	**1.85 (0.99-3.20)**	**2.40 (1.35-3.96)**	**1.77 (0.88-3.22)**	**2.11 (1.08-3.72)**	**1.51 (0.63-3.11)**
	IR[‡]	2.36 (1.18-4.22)	3.02 (1.61-5.16)	2.18 (1.00-4.14)	2.72 (1.30-5.00)	1.94 (0.71-4.22)
Parkinson's disease	Events/At risk	16/429	12/514	<10/496[‖]	10/557	12/528
	**CumI[†]**	**3.73 (2.22-5.84)**	**2.33 (1.28-3.93)**	**1.61 (0.76-3.04)**	**1.80 (0.92-3.18)**	**2.27 (1.24-3.82)**
	IR[‡]	5.09 (2.91-8.26)	3.06 (1.58-5.35)	2.08 (0.90-4.11)	2.35 (1.13-4.33)	2.99 (1.54-5.22)
Alzheimer's disease	Events/At risk	15/506	13/573	14/559	<10/575[‖]	<10/616[‖]
	**CumI[†]**	**2.96 (1.73-4.72)**	**2.27 (1.27-3.75)**	**2.50 (1.44-4.06)**	**1.04 (0.44-2.17)**	**1.30 (0.62-2.46)**
	IR[‡]	4.38 (2.45-7.23)	3.54 (1.88-6.05)	3.95 (2.16-6.63)	1.59 (0.58-3.46)	1.95 (0.84-3.84)
Autoimmune disease	Events/At risk	<10/106[‖]	<10/105[‖]	<10/117[‖]	<10/154[‖]	<10/139[‖]

© 2023 Chen Q et al. *JAMA Network Open.*

	**CumI[†]**	**2.83 (0.76-7.41)**	**1.90 (0.37-6.11)**	**0.85 (0.08-4.26)**	**1.95 (0.53-5.18)**	**28.06 (20.84-35.71)**
	IR[‡]	3.74 (0.77-10.94)	2.56 (0.31-9.26)	1.05 (0.03-5.86)	2.55 (0.52-7.44)	0.00 (0.00-3.37)
Systemic connective tissue disorders	Events/At risk	13/466	16/601	<10/538[‖]	<10/528[‖]	11/640
	**CumI[†]**	**2.79 (1.56-4.59)**	**2.66 (1.59-4.19)**	**1.49 (0.70-2.81)**	**1.14 (0.48-2.36)**	**1.72 (0.91-2.97)**
	IR[‡]	3.59 (1.91-6.14)	3.50 (2.00-5.68)	1.81 (0.78-3.56)	1.46 (0.54-3.19)	2.24 (1.12-4.00)
Venous thromboembolism[#]	Events/At risk	14/839	11/833	15/854	18/998	10/918
	**CumI[†]**	**1.67 (0.96-2.72)**	**1.32 (0.70-2.29)**	**1.76 (1.03-2.82)**	**1.80 (1.11-2.78)**	**1.09 (0.56-1.94)**
	IR[‡]	2.32 (1.27-3.90)	1.75 (0.88-3.14)	2.42 (1.35-3.99)	2.48 (1.47-3.92)	1.49 (0.72-2.74)
Major bleeding[#]	Events/At risk	65/2,287	68/2,454	53/2,444	47/2,617	44/2,545
	**CumI[†]**	**2.84 (2.22-3.58)**	**2.77 (2.17-3.48)**	**2.17 (1.65-2.80)**	**1.80 (1.34-2.36)**	**1.73 (1.28-2.29)**
	IR[‡]	3.66 (2.83-4.67)	3.56 (2.77-4.51)	2.84 (2.12-3.71)	2.29 (1.68-3.04)	2.24 (1.63-3.01)
Malignant tumour	Events/At risk	50/4,364	66/4,664	57/4,682	64/4,830	46/4,979
	**CumI[†]**	**1.15 (0.86-1.50)**	**1.42 (1.11-1.79)**	**1.22 (0.93-1.56)**	**1.33 (1.03-1.68)**	**0.92 (0.69-1.22)**
	IR[‡]	1.83 (1.36-2.42)	2.22 (1.72-2.83)	1.94 (1.47-2.51)	2.09 (1.61-2.67)	1.48 (1.08-1.97)
≥1 hospitalization within the prior 4 years	Events/At risk	631/37,004	694/39,243	589/38,189	581/39,582	530/38,138
	**CumI[†]**	**1.71 (1.58-1.84)**	**1.77 (1.64-1.90)**	**1.54 (1.42-1.67)**	**1.47 (1.35-1.59)**	**1.39 (1.28-1.51)**
	IR[‡]	2.02 (1.87-2.19)	2.09 (1.93-2.25)	1.81 (1.67-1.96)	1.72 (1.58-1.87)	1.63 (1.50-1.78)
Without any hospitalization within the prior 4 years	Events/At risk	278/18,876	309/22,074	323/21,829	336/22,808	327/23,558
	**CumI[†]**	**1.47 (1.31-1.65)**	**1.40 (1.25-1.56)**	**1.48 (1.33-1.65)**	**1.47 (1.32-1.64)**	**1.39 (1.24-1.54)**
	IR[‡]	1.66 (1.47-1.87)	1.57 (1.40-1.76)	1.67 (1.49-1.86)	1.65 (1.48-1.84)	1.56 (1.40-1.74)
Survived until discharge from the index hospitalization	Events/At risk	855/53,468	955/58,695	864/57,461	866/59,681	812/58,831
	**CumI[†]**	**1.60 (1.50-1.71)**	**1.63 (1.53-1.73)**	**1.50 (1.41-1.61)**	**1.45 (1.36-1.55)**	**1.38 (1.29-1.48)**
	IR[‡]	1.79 (1.67-1.91)	1.81 (1.70-1.93)	1.66 (1.56-1.78)	1.60 (1.50-1.71)	1.52 (1.42-1.63)
Main reason for admission to the index hospitalization						
Atrial fibrillation	Events/At risk	183/19,496	238/23,257	220/23,047	223/23,782	193/22,877
	**CumI[†]**	**0.94 (0.81-1.08)**	**1.02 (0.90-1.16)**	**0.95 (0.84-1.09)**	**0.94 (0.82-1.07)**	**0.84 (0.73-0.97)**
	IR[‡]	0.98 (0.84-1.13)	1.06 (0.93-1.21)	0.99 (0.86-1.13)	0.97 (0.85-1.11)	0.87 (0.75-1.01)
Not primarily for atrial fibrillation	Events/At risk	726/36,384	765/38,060	692/36,971	694/38,608	664/38,819
	**CumI[†]**	**2.00 (1.86-2.14)**	**2.01 (1.87-2.15)**	**1.87 (1.74-2.01)**	**1.80 (1.67-1.93)**	**1.71 (1.59-1.84)**
	IR[‡]	2.48 (2.31-2.67)	2.51 (2.33-2.69)	2.33 (2.15-2.50)	2.23 (2.06-2.40)	2.12 (1.97-2.29)
Heart failure	Events/At risk	83/4,566	93/4,906	87/4,767	89/4,872	69/4,905
	**CumI[†]**	**1.82 (1.46-2.24)**	**1.90 (1.54-2.31)**	**1.83 (1.47-2.24)**	**1.83 (1.48-2.23)**	**1.41 (1.11-1.77)**
	IR[‡]	2.41 (1.92-2.99)	2.49 (2.01-3.05)	2.41 (1.93-2.98)	2.41 (1.94-2.97)	1.86 (1.45-2.35)
Ischemic heart diseases	Events/At risk	58/4,504	59/4,544	54/4,469	64/4,714	58/4,831
	**CumI[†]**	**1.29 (0.99-1.65)**	**1.30 (1.00-1.66)**	**1.21 (0.92-1.56)**	**1.36 (1.06-1.72)**	**1.20 (0.92-1.54)**
	IR[‡]	1.41 (1.07-1.83)	1.44 (1.10-1.86)	1.33 (1.00-1.73)	1.49 (1.14-1.90)	1.30 (0.99-1.68)

© 2023 Chen Q et al. *JAMA Network Open.*

Respiratory diseases	Events/At risk	61/3,880	93/4,863	74/4,641	80/5,038	94/5,244
	**CumI[†]**	**1.57 (1.22-2.00)**	**1.91 (1.56-2.33)**	**1.59 (1.26-1.99)**	**1.59 (1.27-1.96)**	**1.79 (1.46-2.18)**
	IR[‡]	2.17 (1.66-2.79)	2.64 (2.13-3.23)	2.18 (1.72-2.74)	2.19 (1.74-2.73)	2.47 (2.00-3.03)
Injury/poisoning	Events/At risk	66/2,972	76/3,120	77/3,138	58/3,209	73/3,464
	**CumI[†]**	**2.22 (1.74-2.80)**	**2.44 (1.94-3.02)**	**2.45 (1.95-3.04)**	**1.81 (1.39-2.31)**	**2.11 (1.67-2.63)**
	IR[‡]	2.90 (2.25-3.69)	3.18 (2.50-3.97)	3.20 (2.53-4.00)	2.33 (1.77-3.01)	2.74 (2.15-3.45)
Symptoms/signs/abnormal laboratory findings[**]	Events/At risk	60/2,922	55/2,997	48/2,761	33/2,655	64/2,483
	**CumI[†]**	**2.05 (1.59-2.62)**	**1.84 (1.40-2.36)**	**1.74 (1.30-2.28)**	**1.24 (0.87-1.72)**	**2.58 (2.01-3.26)**
	IR[‡]	2.44 (1.86-3.13)	2.13 (1.60-2.77)	2.00 (1.48-2.65)	1.40 (0.96-1.96)	2.92 (2.25-3.73)
Neoplasms	Events/At risk	29/2,749	36/2,794	37/2,722	35/2,869	20/2,929
	**CumI[†]**	**1.05 (0.72-1.49)**	**1.29 (0.92-1.76)**	**1.36 (0.97-1.85)**	**1.22 (0.87-1.68)**	**0.68 (0.43-1.04)**
	IR[‡]	1.59 (1.06-2.28)	1.92 (1.34-2.66)	2.07 (1.45-2.85)	1.84 (1.28-2.56)	1.02 (0.63-1.58)
Digestive diseases	Events/At risk	47/2,477	36/2,470	33/2,297	30/2,515	35/2,396
	**CumI[†]**	**1.90 (1.41-2.49)**	**1.46 (1.04-1.99)**	**1.44 (1.01-1.99)**	**1.19 (0.82-1.68)**	**1.46 (1.04-2.00)**
	IR[‡]	2.34 (1.72-3.11)	1.80 (1.26-2.49)	1.78 (1.23-2.50)	1.46 (0.98-2.08)	1.82 (1.27-2.53)
Other heart valve disorders[§§]	Events/At risk	28/1,477	18/1,498	29/1,630	27/1,623	22/1,534
	**CumI[†]**	**1.90 (1.29-2.69)**	**1.20 (0.74-1.86)**	**1.78 (1.22-2.51)**	**1.66 (1.12-2.38)**	**1.43 (0.93-2.13)**
	IR[‡]	2.05 (1.36-2.96)	1.30 (0.77-2.05)	1.92 (1.29-2.76)	1.79 (1.18-2.60)	1.53 (0.96-2.31)
Genitourinary diseases	Events/At risk	24/1,469	34/1,531	34/1,570	38/1,672	40/1,707
	**CumI[†]**	**1.63 (1.08-2.39)**	**2.22 (1.57-3.05)**	**2.17 (1.53-2.98)**	**2.27 (1.64-3.07)**	**2.34 (1.70-3.14)**
	IR[‡]	2.02 (1.29-3.01)	2.82 (1.96-3.95)	2.71 (1.88-3.79)	2.84 (2.01-3.90)	2.98 (2.13-4.06)
Infectious/parasitic diseases	Events/At risk	21/1,059	26/1,069	15/1,037	19/1,204	15/1,189
	**CumI[†]**	**1.98 (1.27-2.96)**	**2.43 (1.63-3.49)**	**1.45 (0.85-2.32)**	**1.58 (0.99-2.41)**	**1.26 (0.74-2.03)**
	IR[‡]	2.84 (1.76-4.35)	3.54 (2.31-5.18)	1.95 (1.09-3.21)	2.15 (1.29-3.36)	1.72 (0.96-2.83)
Endocrine/nutritional/metabolic diseases	Events/At risk	13/680	14/670	12/616	13/634	12/721
	**CumI[†]**	**1.91 (1.07-3.16)**	**2.09 (1.20-3.40)**	**1.95 (1.07-3.29)**	**2.05 (1.15-3.39)**	**1.66 (0.91-2.81)**
	IR[‡]	2.36 (1.26-4.03)	2.71 (1.48-4.55)	2.59 (1.34-4.52)	2.63 (1.40-4.49)	2.15 (1.11-3.76)
Diseases of blood/blood-forming organs	Events/At risk	<10/492[‖]	<10/503[‖]	<10/465[‖]	12/514	10/483
	**CumI[†]**	**1.42 (0.64-2.80)**	**1.79 (0.89-3.26)**	**1.72 (0.81-3.24)**	**2.33 (1.28-3.93)**	**2.07 (1.06-3.65)**
	IR[‡]	1.79 (0.72-3.70)	2.32 (1.06-4.41)	2.18 (0.94-4.30)	3.00 (1.55-5.24)	2.67 (1.28-4.91)
Neurological disorders[##]	Events/At risk	22/491	19/455	13/402	13/441	<10/405[‖]
	**CumI[†]**	**4.48 (2.89-6.57)**	**4.18 (2.60-6.30)**	**3.23 (1.81-5.31)**	**2.95 (1.65-4.85)**	**1.98 (0.93-3.71)**
	IR[‡]	5.32 (3.34-8.06)	5.28 (3.18-8.25)	3.94 (2.10-6.73)	3.55 (1.89-6.07)	2.49 (1.07-4.90)

† Cumulative incidence (%, 95% confidence interval), estimated by the cumulative incidence competing risk method
‡ Incidence rate (per 100-person-year, 95% confidence interval)
* Defined as ≥2 (outpatient) prescription records of antiplatelet agents within six months (*i.e.*, 183 days) before the index dates (exclusive)
§ Identified within the index hospitalizations unless otherwise stated

© 2023 Chen Q et al. *JAMA Network Open.*

|| Except for rheumatic mitral stenosis and mechanical heart valves
Identified within the prior four years (except for venous thromboembolism which was identified within the prior six months)
** Except for abnormalities of heart beat (ICD-10 code: R00) and cardiac murmurs and other cardiac sounds (ICD-10 code: R01)
§§ Except for endocarditis, valve unspecified, in diseases classified elsewhere (ICD-10 code: I398)
Except for transient cerebral ischaemic attacks and related syndromes (ICD-10 code: G45) and vascular syndromes of brain in cerebrovascular diseases (ICD-10 code: G46)
||| Exact result was masked to prevent potential individual or group disclosure.
Abbreviations: CumI, cumulative incidence; IR, incidence rate.

© 2023 Chen Q et al. *JAMA Network Open.*

eTable 21. Subgroup Analysis of Risk of Major Bleeding Within the 1-Year Follow-up After Incident Nonvalvular Atrial Fibrillation Diagnosis

Subgroup	Cohort	2014	2015	2016	2017	2018
Sex						
Male	Events/At risk	788/30,814	786/33,428	772/33,229	784/34,741	781/34,686
	**CumI**†	**2.56 (2.39-2.74)**	**2.35 (2.19-2.52)**	**2.32 (2.17-2.49)**	**2.26 (2.10-2.42)**	**2.25 (2.10-2.41)**
	IR‡	2.96 (2.75-3.17)	2.70 (2.51-2.89)	2.66 (2.48-2.86)	2.57 (2.40-2.76)	2.58 (2.40-2.77)
Female	Events/At risk	580/23,959	550/26,793	494/25,849	462/26,631	478/26,015
	**CumI**†	**2.42 (2.23-2.62)**	**2.05 (1.89-2.23)**	**1.91 (1.75-2.08)**	**1.73 (1.58-1.90)**	**1.84 (1.68-2.01)**
	IR‡	2.87 (2.64-3.11)	2.42 (2.22-2.63)	2.23 (2.04-2.44)	2.04 (1.86-2.23)	2.16 (1.97-2.36)
Immigration background						
Native Dutch	Events/At risk	1,189/48,223	1,149/53,009	1,089/51,618	1,069/53,670	1,062/53,044
	**CumI**†	**2.47 (2.33-2.61)**	**2.17 (2.05-2.29)**	**2.11 (1.99-2.24)**	**1.99 (1.88-2.11)**	**2.00 (1.89-2.12)**
	IR‡	2.88 (2.72-3.05)	2.52 (2.38-2.67)	2.44 (2.30-2.59)	2.30 (2.17-2.44)	2.32 (2.19-2.47)
First/Second-generation immigrants	Events/At risk	179/6,550	187/7,212	177/7,460	177/7,702	197/7,657
	**CumI**†	**2.73 (2.36-3.15)**	**2.59 (2.24-2.98)**	**2.37 (2.05-2.74)**	**2.30 (1.98-2.65)**	**2.57 (2.24-2.95)**
	IR‡	3.18 (2.73-3.68)	2.99 (2.58-3.45)	2.72 (2.33-3.15)	2.64 (2.26-3.06)	2.93 (2.54-3.37)
CHA_2DS_2-VASc score						
0	Events/At risk	33/4,019	40/4,766	37/4,765	33/4,734	27/4,571
	**CumI**†	**0.82 (0.58-1.14)**	**0.84 (0.61-1.13)**	**0.78 (0.56-1.06)**	**0.70 (0.49-0.97)**	**0.59 (0.40-0.85)**
	IR‡	0.85 (0.58-1.19)	0.87 (0.62-1.18)	0.80 (0.56-1.10)	0.72 (0.49-1.00)	0.61 (0.40-0.88)
1	Events/At risk	115/7,355	121/8,233	114/8,088	89/8,519	100/8,494
	**CumI**†	**1.56 (1.30-1.87)**	**1.47 (1.23-1.75)**	**1.41 (1.17-1.68)**	**1.04 (0.85-1.28)**	**1.18 (0.96-1.42)**
	IR‡	1.66 (1.37-1.99)	1.55 (1.29-1.86)	1.49 (1.23-1.79)	1.10 (0.88-1.36)	1.25 (1.01-1.52)
≥2	Events/At risk	1,220/43,399	1,175/47,222	1,115/46,225	1124/48,119	1,132/47,636
	**CumI**†	**2.81 (2.66-2.97)**	**2.49 (2.35-2.63)**	**2.41 (2.28-2.55)**	**2.34 (2.20-2.47)**	**2.38 (2.24-2.52)**
	IR‡	3.39 (3.20-3.58)	2.98 (2.81-3.15)	2.87 (2.70-3.04)	2.78 (2.62-2.95)	2.83 (2.67-3.00)
HAS-BLED score						
0	Events/At risk	41/5,034	60/6,083	45/6,227	41/6,506	45/6,531
	**CumI**†	**0.81 (0.59-1.09)**	**0.99 (0.76-1.26)**	**0.72 (0.54-0.96)**	**0.63 (0.46-0.85)**	**0.69 (0.51-0.91)**
	IR‡	0.84 (0.60-1.14)	1.02 (0.78-1.32)	0.74 (0.54-1.00)	0.65 (0.47-0.88)	0.71 (0.52-0.95)
1	Events/At risk	380/17,893	358/20,446	357/20,399	359/21,395	370/21,407
	**CumI**†	**2.12 (1.92-2.34)**	**1.75 (1.58-1.94)**	**1.75 (1.58-1.94)**	**1.68 (1.51-1.86)**	**1.73 (1.56-1.91)**
	IR‡	2.41 (2.17-2.66)	1.97 (1.78-2.19)	1.97 (1.77-2.18)	1.88 (1.69-2.08)	1.94 (1.75-2.15)
2	Events/At risk	508/19,634	517/21,258	466/20,449	482/21,068	469/20,898
	**CumI**†	**2.59 (2.37-2.82)**	**2.43 (2.23-2.65)**	**2.28 (2.08-2.49)**	**2.29 (2.09-2.50)**	**2.24 (2.05-2.45)**
	IR‡	3.05 (2.79-3.33)	2.85 (2.61-3.11)	2.66 (2.43-2.92)	2.68 (2.44-2.93)	2.64 (2.40-2.89)
≥3	Events/At risk	439/12,212	401/12,434	398/12,003	364/12,403	375/11,865

© 2023 Chen Q et al. *JAMA Network Open.*

	**CumI†**	**3.59 (3.28-3.94)**	**3.23 (2.92-3.55)**	**3.32 (3.01-3.65)**	**2.93 (2.65-3.24)**	**3.16 (2.86-3.49)**
	IR‡	4.59 (4.17-5.04)	4.13 (3.73-4.55)	4.23 (3.82-4.67)	3.76 (3.38-4.16)	4.05 (3.65-4.48)
Use of antithrombotic agents						
Pre-existing chronic use of antiplatelet agents*	Events/At risk	434/15,823	418/16,107	406/15,605	373/16,502	408/16,101
	**CumI†**	**2.74 (2.50-3.01)**	**2.60 (2.36-2.85)**	**2.60 (2.36-2.86)**	**2.26 (2.04-2.50)**	**2.53 (2.30-2.79)**
	IR‡	3.25 (2.95-3.57)	3.08 (2.79-3.39)	3.09 (2.80-3.41)	2.67 (2.40-2.95)	3.00 (2.72-3.31)
≥1 oral anticoagulant prescription within the 1-year follow-up	Events/At risk	984/37,379	1,032/43,876	976/43,325	967/44,606	965/44,357
	**CumI†**	**2.63 (2.47-2.80)**	**2.35 (2.21-2.50)**	**2.25 (2.12-2.40)**	**2.17 (2.04-2.31)**	**2.18 (2.04-2.31)**
	IR‡	2.83 (2.65-3.01)	2.52 (2.36-2.67)	2.41 (2.26-2.56)	2.32 (2.18-2.47)	2.33 (2.19-2.48)
Comorbidities (or medical history)§						
Asthma	Events/At risk	17/667	20/828	14/776	22/898	20/1,033
	**CumI†**	**2.55 (1.54-3.96)**	**2.42 (1.53-3.64)**	**1.80 (1.04-2.94)**	**2.45 (1.58-3.62)**	**1.94 (1.22-2.92)**
	IR‡	2.90 (1.69-4.65)	2.77 (1.69-4.28)	2.05 (1.12-3.43)	2.79 (1.75-4.23)	2.26 (1.38-3.48)
Chronic obstructive pulmonary disease	Events/At risk	159/5,740	143/5,996	164/5,639	134/5,648	152/5,621
	**CumI†**	**2.77 (2.37-3.22)**	**2.38 (2.02-2.79)**	**2.91 (2.49-3.37)**	**2.37 (2.00-2.79)**	**2.70 (2.30-3.15)**
	IR‡	3.66 (3.11-4.28)	3.17 (2.67-3.73)	3.83 (3.26-4.46)	3.15 (2.64-3.73)	3.57 (3.02-4.18)
Other chronic lung disease	Events/At risk	14/710	28/804	23/778	27/921	23/1,077
	**CumI†**	**1.97 (1.13-3.21)**	**3.48 (2.37-4.92)**	**2.96 (1.93-4.33)**	**2.93 (1.98-4.17)**	**2.14 (1.39-3.14)**
	IR‡	3.06 (1.68-5.14)	5.60 (3.72-8.10)	4.75 (3.01-7.12)	4.72 (3.11-6.86)	3.47 (2.20-5.20)
Heart failure	Events/At risk	236/9,874	259/10,631	262/10,346	232/10,802	273/10,961
	**CumI†**	**2.39 (2.10-2.71)**	**2.44 (2.16-2.74)**	**2.53 (2.24-2.85)**	**2.15 (1.89-2.43)**	**2.49 (2.21-2.80)**
	IR‡	3.26 (2.85-3.70)	3.29 (2.90-3.71)	3.43 (3.03-3.88)	2.89 (2.53-3.29)	3.37 (2.98-3.79)
Myocardial infarction (history)	Events/At risk	75/2,539	85/2,884	94/2,992	82/3,183	83/3,207
	**CumI†**	**2.95 (2.35-3.67)**	**2.95 (2.38-3.61)**	**3.14 (2.56-3.81)**	**2.58 (2.07-3.17)**	**2.59 (2.08-3.18)**
	IR‡	3.62 (2.85-4.53)	3.58 (2.86-4.43)	3.80 (3.07-4.66)	3.08 (2.45-3.82)	3.08 (2.45-3.82)
Hypertension	Events/At risk	470/15,835	443/17,490	403/17,027	419/18,150	447/18,733
	**CumI†**	**2.97 (2.71-3.24)**	**2.53 (2.31-2.77)**	**2.37 (2.15-2.60)**	**2.31 (2.10-2.53)**	**2.39 (2.17-2.61)**
	IR‡	3.50 (3.19-3.83)	2.97 (2.70-3.26)	2.75 (2.49-3.03)	2.71 (2.45-2.98)	2.80 (2.55-3.07)
Other valvular heart disease‖	Events/At risk	184/6,575	189/6,972	188/6,591	178/6,816	178/6,675
	**CumI†**	**2.80 (2.42-3.22)**	**2.71 (2.35-3.11)**	**2.85 (2.47-3.27)**	**2.61 (2.25-3.01)**	**2.67 (2.30-3.07)**
	IR‡	3.42 (2.94-3.95)	3.35 (2.89-3.86)	3.50 (3.01-4.03)	3.20 (2.75-3.71)	3.28 (2.81-3.79)
Peripheral artery disease	Events/At risk	38/1,014	40/1,197	28/986	25/1,010	28/973
	**CumI†**	**3.75 (2.70-5.05)**	**3.34 (2.43-4.47)**	**2.84 (1.93-4.02)**	**2.48 (1.65-3.58)**	**2.88 (1.96-4.07)**
	IR‡	4.78 (3.38-6.56)	4.52 (3.23-6.16)	3.70 (2.46-5.35)	3.25 (2.10-4.80)	3.86 (2.56-5.58)
Liver diseases	Events/At risk	38/1,024	25/1,038	29/1,020	32/1,035	43/1,021
	**CumI†**	**3.71 (2.67-5.00)**	**2.41 (1.60-3.48)**	**2.84 (1.95-4.00)**	**3.09 (2.16-4.28)**	**4.21 (3.10-5.57)**
	IR‡	5.39 (3.81-7.40)	3.51 (2.27-5.19)	4.17 (2.80-6.00)	4.62 (3.16-6.52)	6.74 (4.88-9.08)

© 2023 Chen Q et al. *JAMA Network Open.*

Gastroesophageal reflux disease	Events/At risk	<10/105[‖]	<10/85[‖]	<10/92[‖]	<10/116[‖]	<10/101[‖]
	**CumI[†]**	**5.71 (2.33-11.32)**	**3.53 (0.93-9.14)**	**6.52 (2.65-12.84)**	**6.03 (2.65-11.40)**	**6.93 (3.04-13.01)**
	IR[‡]	6.95 (2.55-15.13)	4.32 (0.89-12.63)	8.67 (3.18-18.88)	7.52 (3.02-15.49)	9.64 (3.88-19.86)
Peptic ulcer disease	Events/At risk	11/175	17/172	15/149	10/147	11/154
	**CumI[†]**	**6.29 (3.32-10.56)**	**9.88 (5.99-14.91)**	**10.07 (5.89-15.55)**	**6.80 (3.47-11.66)**	**7.14 (3.77-11.94)**
	IR[‡]	9.09 (4.54-16.26)	14.65 (8.53-23.45)	16.07 (9.00-26.51)	10.03 (4.81-18.45)	10.25 (5.12-18.34)
Chronic kidney diseases	Events/At risk	222/5,683	192/6,193	205/6,420	192/6,777	210/6,475
	**CumI[†]**	**3.91 (3.42-4.43)**	**3.10 (2.69-3.55)**	**3.19 (2.78-3.64)**	**2.83 (2.46-3.25)**	**3.24 (2.83-3.70)**
	IR[‡]	5.60 (4.88-6.38)	4.42 (3.81-5.09)	4.46 (3.87-5.12)	3.96 (3.42-4.56)	4.59 (3.99-5.25)
Anaemia	Events/At risk	164/3,396	174/3,740	171/3,916	175/4,255	183/4,411
	**CumI[†]**	**4.83 (4.14-5.59)**	**4.65 (4.01-5.36)**	**4.37 (3.76-5.04)**	**4.11 (3.55-4.74)**	**4.15 (3.59-4.77)**
	IR[‡]	6.63 (5.65-7.72)	6.44 (5.52-7.47)	6.05 (5.18-7.03)	5.70 (4.89-6.61)	5.77 (4.96-6.66)
Coagulopathy	Events/At risk	29/659	28/912	35/891	28/946	38/1,075
	**CumI[†]**	**4.40 (3.02-6.16)**	**3.07 (2.09-4.34)**	**3.93 (2.79-5.35)**	**2.96 (2.01-4.19)**	**3.53 (2.55-4.76)**
	IR[‡]	6.31 (4.23-9.07)	4.31 (2.87-6.23)	5.69 (3.96-7.91)	4.42 (2.94-6.38)	5.32 (3.76-7.30)
Diabetes	Events/At risk	233/9,007	231/9,823	225/9,771	239/10,113	248/10,157
	**CumI[†]**	**2.59 (2.27-2.93)**	**2.35 (2.07-2.67)**	**2.30 (2.02-2.61)**	**2.36 (2.08-2.67)**	**2.44 (2.15-2.76)**
	IR[‡]	3.21 (2.81-3.65)	2.90 (2.54-3.30)	2.84 (2.48-3.24)	2.93 (2.57-3.32)	3.05 (2.68-3.45)
Thyroid disease	Events/At risk	33/1,380	39/1,482	15/1,385	26/1,556	30/1,548
	**CumI[†]**	**2.39 (1.68-3.30)**	**2.63 (1.90-3.54)**	**1.08 (0.64-1.74)**	**1.67 (1.12-2.40)**	**1.94 (1.34-2.72)**
	IR[‡]	2.86 (1.97-4.01)	3.15 (2.24-4.31)	1.26 (0.71-2.08)	1.96 (1.28-2.87)	2.34 (1.58-3.34)
Ischemic stroke (history)[#]	Events/At risk	38/1,574	43/1,718	42/1,653	42/1,761	44/1,642
	**CumI[†]**	**2.41 (1.74-3.26)**	**2.50 (1.84-3.32)**	**2.54 (1.86-3.38)**	**2.39 (1.75-3.18)**	**2.68 (1.98-3.55)**
	IR[‡]	3.07 (2.17-4.21)	3.12 (2.26-4.21)	3.19 (2.30-4.31)	2.97 (2.14-4.01)	3.32 (2.41-4.46)
Transient ischemic attack[#]	Events/At risk	11/773	19/819	26/794	28/844	27/763
	**CumI[†]**	**1.42 (0.76-2.46)**	**2.32 (1.45-3.53)**	**3.27 (2.19-4.68)**	**3.32 (2.26-4.69)**	**3.54 (2.39-5.03)**
	IR[‡]	1.68 (0.84-3.00)	2.67 (1.61-4.17)	3.91 (2.55-5.73)	3.79 (2.52-5.48)	4.14 (2.73-6.03)
Other arterial thromboembolism[#]	Events/At risk	23/591	16/531	14/499	15/469	11/392
	**CumI[†]**	**3.89 (2.54-5.68)**	**3.01 (1.80-4.73)**	**2.81 (1.61-4.54)**	**3.20 (1.87-5.09)**	**2.81 (1.49-4.81)**
	IR[‡]	5.02 (3.18-7.53)	3.84 (2.20-6.24)	3.49 (1.91-5.86)	4.12 (2.30-6.79)	3.63 (1.81-6.50)
Parkinson's disease	Events/At risk	11/418	14/500	17/489	11/539	17/511
	**CumI[†]**	**2.63 (1.40-4.51)**	**2.80 (1.61-4.53)**	**3.48 (2.10-5.38)**	**2.04 (1.08-3.51)**	**3.33 (2.01-5.15)**
	IR[‡]	3.61 (1.80-6.47)	3.66 (2.00-6.14)	4.55 (2.65-7.29)	2.68 (1.34-4.79)	4.40 (2.56-7.04)
Alzheimer's disease	Events/At risk	11/485	12/549	6/546	13/553	11/595
	**CumI[†]**	**2.27 (1.20-3.90)**	**2.19 (1.20-3.68)**	**1.10 (0.46-2.28)**	**2.35 (1.32-3.88)**	**1.85 (0.98-3.19)**
	IR[‡]	3.31 (1.65-5.92)	3.37 (1.74-5.89)	1.72 (0.63-3.75)	3.66 (1.95-6.26)	2.77 (1.39-4.96)
Autoimmune disease	Events/At risk	<10/105[‖]	<10/103[‖]	<10/116[‖]	<10/154[‖]	<10/137[‖]

© 2023 Chen Q et al. *JAMA Network Open.*

	**CumI†**	**1.90 (0.37-6.11)**	**0.97 (0.08-4.80)**	**1.72 (0.33-5.56)**	**2.60 (0.85-6.09)**	**2.19 (0.59-5.80)**
	IR‡	2.51 (0.30-9.06)	1.30 (0.03-7.22)	2.15 (0.26-7.76)	3.39 (0.92-8.67)	2.80 (0.58-8.19)
Systemic connective tissue disorders	Events/At risk	14/461	11/585	13/532	15/520	17/633
	**CumI†**	**3.04 (1.74-4.91)**	**1.88 (1.00-3.24)**	**2.44 (1.37-4.03)**	**2.88 (1.69-4.60)**	**2.69 (1.63-4.17)**
	IR‡	3.91 (2.14-6.55)	2.46 (1.23-4.39)	2.99 (1.59-5.11)	3.76 (2.10-6.20)	3.55 (2.07-5.68)
Venous thromboembolism#	Events/At risk	27/829	21/815	27/843	30/983	28/899
	**CumI†**	**3.26 (2.20-4.63)**	**2.58 (1.65-3.84)**	**3.20 (2.16-4.55)**	**3.05 (2.11-4.27)**	**3.11 (2.12-4.40)**
	IR‡	4.57 (3.01-6.65)	3.43 (2.12-5.25)	4.44 (2.92-6.45)	4.23 (2.86-6.04)	4.32 (2.87-6.25)
Major bleeding#	Events/At risk	129/2,145	129/2,318	126/2,314	144/2,483	132/2,403
	**CumI†**	**6.01 (5.06-7.07)**	**5.57 (4.68-6.55)**	**5.45 (4.57-6.42)**	**5.80 (4.93-6.77)**	**5.49 (4.63-6.45)**
	IR‡	7.88 (6.58-9.37)	7.29 (6.09-8.66)	7.28 (6.07-8.67)	7.56 (6.38-8.90)	7.26 (6.08-8.61)
Malignant tumour	Events/At risk	147/4,320	145/4,614	125/4,612	140/4,770	122/4,906
	**CumI†**	**3.40 (2.89-3.97)**	**3.14 (2.67-3.68)**	**2.71 (2.27-3.21)**	**2.94 (2.48-3.44)**	**2.49 (2.08-2.95)**
	IR‡	5.54 (4.68-6.51)	5.01 (4.23-5.89)	4.35 (3.62-5.18)	4.69 (3.95-5.54)	4.02 (3.34-4.80)
≥1 hospitalization within the prior 4 years	Events/At risk	997/36,263	965/38,555	914/37,611	893/38,947	898/37,538
	**CumI†**	**2.75 (2.58-2.92)**	**2.50 (2.35-2.66)**	**2.43 (2.28-2.59)**	**2.29 (2.15-2.45)**	**2.39 (2.24-2.55)**
	IR‡	3.28 (3.07-3.48)	2.96 (2.78-3.15)	2.86 (2.67-3.05)	2.70 (2.52-2.88)	2.82 (2.64-3.01)
Without any hospitalization within the prior 4 years	Events/At risk	371/18,510	371/21,666	352/21,467	353/22,425	361/23,163
	**CumI†**	**2.00 (1.81-2.21)**	**1.71 (1.55-1.89)**	**1.64 (1.48-1.82)**	**1.57 (1.42-1.74)**	**1.56 (1.41-1.72)**
	IR‡	2.26 (2.04-2.50)	1.93 (1.73-2.13)	1.84 (1.65-2.04)	1.76 (1.58-1.96)	1.75 (1.58-1.94)
Survived until discharge from the index hospitalization	Events/At risk	1,340/52,524	1,308/57,731	1,236/56,662	1,211/58,789	1,232/57,959
	**CumI†**	**2.55 (2.42-2.69)**	**2.27 (2.15-2.39)**	**2.18 (2.06-2.30)**	**2.06 (1.95-2.18)**	**2.13 (2.01-2.25)**
	IR‡	2.86 (2.71-3.02)	2.53 (2.39-2.67)	2.42 (2.29-2.56)	2.28 (2.16-2.41)	2.35 (2.22-2.49)
Main reason for admission to the index hospitalization						
Atrial fibrillation	Events/At risk	319/19,496	320/23,256	322/23,047	307/23,782	306/22,876
	**CumI†**	**1.64 (1.47-1.82)**	**1.38 (1.23-1.53)**	**1.40 (1.25-1.56)**	**1.29 (1.15-1.44)**	**1.34 (1.20-1.49)**
	IR‡	1.71 (1.53-1.91)	1.43 (1.28-1.60)	1.45 (1.30-1.62)	1.34 (1.19-1.50)	1.39 (1.24-1.55)
Not primarily for atrial fibrillation	Events/At risk	1,049/35,277	1,016/36,965	944/36,031	939/37,590	953/37,825
	**CumI†**	**2.97 (2.80-3.15)**	**2.75 (2.59-2.92)**	**2.62 (2.46-2.79)**	**2.50 (2.34-2.66)**	**2.52 (2.37-2.68)**
	IR‡	3.72 (3.50-3.95)	3.44 (3.23-3.66)	3.26 (3.06-3.48)	3.11 (2.91-3.31)	3.14 (2.94-3.35)
Heart failure	Events/At risk	107/4,566	123/4,906	118/4,767	110/4,872	115/4,905
	**CumI†**	**2.34 (1.93-2.81)**	**2.51 (2.10-2.97)**	**2.48 (2.06-2.95)**	**2.26 (1.87-2.70)**	**2.34 (1.95-2.80)**
	IR‡	3.12 (2.56-3.77)	3.31 (2.75-3.95)	3.28 (2.72-3.93)	2.99 (2.45-3.60)	3.11 (2.57-3.74)
Ischemic heart diseases	Events/At risk	108/4,504	116/4,543	127/4,469	115/4,714	120/4,831
	**CumI†**	**2.40 (1.98-2.88)**	**2.55 (2.12-3.04)**	**2.84 (2.38-3.36)**	**2.44 (2.03-2.91)**	**2.48 (2.07-2.95)**
	IR‡	2.65 (2.18-3.20)	2.85 (2.35-3.42)	3.15 (2.63-3.75)	2.69 (2.22-3.23)	2.72 (2.25-3.25)

© 2023 Chen Q et al. *JAMA Network Open.*

Respiratory diseases	Events/At risk	87/3,871	110/4,860	115/4,634	100/5,032	125/5238
	**CumI†**	**2.25 (1.82-2.75)**	**2.26 (1.87-2.71)**	**2.48 (2.06-2.96)**	**1.99 (1.63-2.40)**	**2.39 (2.00-2.83)**
	IR‡	3.12 (2.50-3.84)	3.13 (2.57-3.77)	3.42 (2.83-4.11)	2.76 (2.24-3.35)	3.31 (2.76-3.94)
Injury/poisoning	Events/At risk	105/2,826	77/2,976	89/2,991	84/3,049	98/3,267
	**CumI†**	**3.72 (3.06-4.46)**	**2.59 (2.06-3.20)**	**2.98 (2.41-3.63)**	**2.76 (2.22-3.38)**	**3.00 (2.45-3.63)**
	IR‡	4.89 (4.00-5.92)	3.38 (2.67-4.22)	3.89 (3.13-4.79)	3.56 (2.84-4.41)	3.92 (3.18-4.77)
Symptoms/signs/abnormal laboratory findings**	Events/At risk	100/2,778	90/2,853	57/2,684	60/2,559	63/2,393
	**CumI†**	**3.60 (2.95-4.34)**	**3.15 (2.56-3.84)**	**2.12 (1.63-2.72)**	**2.34 (1.81-2.99)**	**2.63 (2.05-3.33)**
	IR‡	4.31 (3.51-5.24)	3.70 (2.98-4.55)	2.44 (1.85-3.17)	2.65 (2.02-3.41)	2.97 (2.28-3.80)
Neoplasms	Events/At risk	95/2,748	97/2,793	71/2,721	84/2,868	75/2,929
	**CumI†**	**3.46 (2.82-4.19)**	**3.47 (2.84-4.20)**	**2.61 (2.06-3.26)**	**2.93 (2.36-3.59)**	**2.56 (2.03-3.18)**
	IR‡	5.30 (4.29-6.48)	5.28 (4.28-6.44)	4.01 (3.13-5.06)	4.48 (3.58-5.55)	3.90 (3.07-4.89)
Digestive diseases	Events/At risk	78/2,123	70/2,138	75/1,996	77/2,200	61/2,093
	**CumI†**	**3.67 (2.93-4.54)**	**3.27 (2.58-4.09)**	**3.76 (2.99-4.66)**	**3.50 (2.79-4.33)**	**2.91 (2.26-3.70)**
	IR‡	4.61 (3.64-5.75)	4.10 (3.19-5.18)	4.73 (3.72-5.93)	4.34 (3.42-5.42)	3.67 (2.81-4.71)
Other heart valve disorders§§	Events/At risk	41/1,477	39/1,498	41/1,630	38/1,623	33/1,534
	**CumI†**	**2.78 (2.03-3.71)**	**2.60 (1.88-3.50)**	**2.52 (1.84-3.36)**	**2.34 (1.69-3.17)**	**2.15 (1.51-2.97)**
	IR‡	3.02 (2.17-4.10)	2.84 (2.02-3.88)	2.73 (1.96-3.70)	2.53 (1.79-3.47)	2.29 (1.58-3.22)
Genitourinary diseases	Events/At risk	49/1,466	58/1,527	53/1,565	47/1,669	53/1,702
	**CumI†**	**3.34 (2.51-4.35)**	**3.80 (2.92-4.84)**	**3.39 (2.57-4.37)**	**2.82 (2.10-3.69)**	**3.11 (2.36-4.02)**
	IR‡	4.18 (3.09-5.52)	4.88 (3.70-6.30)	4.29 (3.21-5.61)	3.56 (2.61-4.73)	3.99 (2.99-5.22)
Infectious/parasitic diseases	Events/At risk	35/1,059	17/1,069	29/1,037	29/1,204	28/1,189
	**CumI†**	**3.31 (2.35-4.51)**	**1.59 (0.96-2.48)**	**2.80 (1.92-3.94)**	**2.41 (1.65-3.39)**	**2.35 (1.60-3.34)**
	IR‡	4.78 (3.33-6.65)	2.29 (1.34-3.67)	3.79 (2.54-5.45)	3.30 (2.21-4.74)	3.22 (2.14-4.66)
Endocrine/nutritional/metabolic diseases	Events/At risk	16/680	19/670	11/616	15/634	21/721
	**CumI†**	**2.35 (1.40-3.71)**	**2.84 (1.77-4.30)**	**1.79 (0.95-3.08)**	**2.37 (1.38-3.78)**	**2.91 (1.86-4.33)**
	IR‡	2.92 (1.67-4.74)	3.70 (2.23-5.78)	2.37 (1.18-4.24)	3.02 (1.69-4.98)	3.82 (2.36-5.83)
Diseases of blood/blood-forming organs	Events/At risk	29/454	28/446	24/425	26/465	25/450
	**CumI†**	**6.39 (4.39-8.89)**	**6.28 (4.28-8.79)**	**5.65 (3.72-8.13)**	**5.59 (3.75-7.94)**	**5.56 (3.70-7.94)**
	IR‡	8.38 (5.61-12.03)	8.45 (5.61-12.21)	7.44 (4.76-11.06)	7.33 (4.79-10.74)	7.36 (4.76-10.87)
Neurological disorders##	Events/At risk	10/491	15/455	8/402	11/441	10/405
	**CumI†**	**2.04 (1.05-3.59)**	**3.30 (1.93-5.24)**	**1.99 (0.94-3.74)**	**2.49 (1.32-4.28)**	**2.47 (1.27-4.34)**
	IR‡	2.42 (1.16-4.45)	4.18 (2.34-6.90)	2.44 (1.05-4.81)	3.00 (1.50-5.36)	3.10 (1.49-5.71)

† Cumulative incidence (%, 95% confidence interval), estimated by the cumulative incidence competing risk method

‡ Incidence rate (per 100-person-year, 95% confidence interval)

* Defined as ≥2 (outpatient) prescription records of antiplatelet agents within six months (*i.e.*, 183 days) before the index dates (exclusive)

§ Identified within the index hospitalizations unless otherwise stated

© 2023 Chen Q et al. *JAMA Network Open.*

|| Except for rheumatic mitral stenosis and mechanical heart valves
Identified within the prior four years (except for venous thromboembolism which was identified within the prior six months)
** Except for abnormalities of heart beat (ICD-10 code: R00) and cardiac murmurs and other cardiac sounds (ICD-10 code: R01)
§§ Except for endocarditis, valve unspecified, in diseases classified elsewhere (ICD-10 code: I398)
Except for transient cerebral ischaemic attacks and related syndromes (ICD-10 code: G45) and vascular syndromes of brain in cerebrovascular diseases (ICD-10 code: G46)
||| Exact result was masked to prevent potential individual or group disclosure.
Abbreviations: CumI, cumulative incidence; IR, incidence rate.

© 2023 Chen Q et al. *JAMA Network Open.*

eTable 22. Subgroup Analysis of Risk of Intracranial Hemorrhage Within the 1-Year Follow-up After Incident Nonvalvular Atrial Fibrillation Diagnosis

Subgroup	Cohort	2014	2015	2016	2017	2018
Sex						
Male	Events/At risk	168/31,187	176/33,817	155/33,567	131/35,104	136/35,048
	**CumI[†]**	**0.54 (0.46-0.63)**	**0.52 (0.45-0.60)**	**0.46 (0.39-0.54)**	**0.37 (0.31-0.44)**	**0.39 (0.33-0.46)**
	IR[‡]	0.62 (0.53-0.72)	0.59 (0.51-0.69)	0.52 (0.45-0.61)	0.42 (0.35-0.50)	0.44 (0.37-0.52)
Female	Events/At risk	164/24,281	137/27,090	104/26,088	92/26,901	111/26,291
	**CumI[†]**	**0.68 (0.58-0.78)**	**0.51 (0.43-0.60)**	**0.40 (0.33-0.48)**	**0.34 (0.28-0.42)**	**0.42 (0.35-0.51)**
	IR[‡]	0.79 (0.68-0.93)	0.59 (0.50-0.70)	0.46 (0.38-0.56)	0.40 (0.32-0.49)	0.49 (0.41-0.59)
Immigration background						
Native Dutch	Events/At risk	284/48,830	264/53,616	220/52,122	192/54,219	205/53,603
	**CumI[†]**	**0.58 (0.52-0.65)**	**0.49 (0.44-0.55)**	**0.42 (0.37-0.48)**	**0.35 (0.31-0.41)**	**0.38 (0.33-0.44)**
	IR[‡]	0.67 (0.60-0.76)	0.57 (0.50-0.64)	0.48 (0.42-0.55)	0.41 (0.35-0.47)	0.44 (0.38-0.50)
First/Second-generation immigrants	Events/At risk	48/6,638	49/7,291	39/7,533	31/7,786	42/7,736
	**CumI[†]**	**0.72 (0.54-0.95)**	**0.67 (0.50-0.88)**	**0.52 (0.37-0.70)**	**0.40 (0.28-0.56)**	**0.54 (0.40-0.73)**
	IR[‡]	0.83 (0.61-1.10)	0.77 (0.57-1.01)	0.59 (0.42-0.80)	0.45 (0.31-0.64)	0.61 (0.44-0.83)
CHA_2DS_2-VASc score						
0	Events/At risk	<10/4,034[‖]	<10/4,782[‖]	<10/4,782[‖]	<10/4,753[‖]	<10/4,585[‖]
	**CumI[†]**	**0.07 (0.02-0.21)**	**0.15 (0.07-0.29)**	**0.08 (0.03-0.21)**	**0.04 (0.01-0.15)**	**0.07 (0.02-0.19)**
	IR[‡]	0.08 (0.02-0.22)	0.15 (0.06-0.31)	0.09 (0.02-0.22)	0.04 (0.01-0.16)	0.07 (0.01-0.20)
1	Events/At risk	22/7,391	21/8,276	16/8,133	13/8,558	16/8,533
	**CumI[†]**	**0.30 (0.19-0.45)**	**0.25 (0.16-0.38)**	**0.20 (0.12-0.32)**	**0.15 (0.09-0.26)**	**0.19 (0.11-0.30)**
	IR[‡]	0.31 (0.20-0.48)	0.27 (0.16-0.41)	0.21 (0.12-0.34)	0.16 (0.08-0.27)	0.20 (0.11-0.32)
≥2	Events/At risk	307/44,043	285/47,849	239/46,740	208/48,694	228/48,221
	**CumI[†]**	**0.70 (0.62-0.78)**	**0.60 (0.53-0.67)**	**0.51 (0.45-0.58)**	**0.43 (0.37-0.49)**	**0.47 (0.41-0.54)**
	IR[‡]	0.83 (0.74-0.93)	0.71 (0.63-0.79)	0.60 (0.53-0.68)	0.50 (0.44-0.58)	0.56 (0.49-0.64)
HAS-BLED score						
0	Events/At risk	<10/5,034[‖]	11/6,083	<10/6,227[‖]	<10/6,506[‖]	<10/6,531[‖]
	**CumI[†]**	**0.06 (0.02-0.17)**	**0.18 (0.10-0.32)**	**0.11 (0.05-0.23)**	**0.02 (0.00-0.09)**	**0.14 (0.07-0.26)**
	IR[‡]	0.06 (0.01-0.18)	0.19 (0.09-0.33)	0.12 (0.05-0.24)	0.02 (0.00-0.09)	0.14 (0.06-0.27)
1	Events/At risk	85/17,908	92/20,457	58/20,415	72/21,409	74/21,424
	**CumI[†]**	**0.47 (0.38-0.58)**	**0.45 (0.37-0.55)**	**0.28 (0.22-0.37)**	**0.34 (0.27-0.42)**	**0.35 (0.27-0.43)**
	IR[‡]	0.53 (0.43-0.66)	0.50 (0.41-0.62)	0.32 (0.24-0.41)	0.37 (0.29-0.47)	0.39 (0.30-0.48)
2	Events/At risk	123/19,862	118/21,533	99/20,661	83/21,319	92/21,147
	**CumI[†]**	**0.62 (0.52-0.74)**	**0.55 (0.46-0.65)**	**0.48 (0.39-0.58)**	**0.39 (0.31-0.48)**	**0.44 (0.35-0.53)**
	IR[‡]	0.72 (0.60-0.86)	0.64 (0.53-0.76)	0.55 (0.45-0.68)	0.45 (0.36-0.56)	0.51 (0.41-0.62)
≥3	Events/At risk	121/12,664	92/12,834	95/12,352	67/12,771	72/12,237

© 2023 Chen Q et al. *JAMA Network Open.*

	**CumI[†]**	**0.96 (0.80-1.14)**	**0.72 (0.58-0.88)**	**0.77 (0.63-0.94)**	**0.52 (0.41-0.66)**	**0.59 (0.46-0.74)**
	IR[‡]	1.20 (1.00-1.44)	0.90 (0.73-1.11)	0.97 (0.78-1.18)	0.66 (0.51-0.84)	0.74 (0.58-0.94)
Use of antithrombotic agents						
Pre-existing chronic use of antiplatelet agents[*]	Events/At risk	101/15,978	87/16,231	82/15,724	61/16,614	66/16,213
	**CumI[†]**	**0.63 (0.52-0.76)**	**0.54 (0.43-0.66)**	**0.52 (0.42-0.64)**	**0.37 (0.28-0.47)**	**0.41 (0.32-0.52)**
	IR[‡]	0.74 (0.60-0.90)	0.63 (0.50-0.77)	0.61 (0.49-0.76)	0.43 (0.33-0.55)	0.48 (0.37-0.61)
≥1 oral anticoagulant prescription within the 1-year follow-up	Events/At risk	211/37,781	233/44,281	188/43,662	145/45,024	176/44,734
	**CumI[†]**	**0.56 (0.49-0.64)**	**0.53 (0.46-0.60)**	**0.43 (0.37-0.50)**	**0.32 (0.27-0.38)**	**0.39 (0.34-0.46)**
	IR[‡]	0.59 (0.52-0.68)	0.56 (0.49-0.63)	0.46 (0.39-0.53)	0.34 (0.29-0.40)	0.42 (0.36-0.48)
Comorbidities (or medical history)[§]						
Asthma	Events/At risk	<10/681[⫴]	<10/833[⫴]	<10/782[⫴]	<10/907[⫴]	<10/1,043[⫴]
	**CumI[†]**	**0.73 (0.28-1.64)**	**0.72 (0.30-1.50)**	**0.38 (0.11-1.07)**	**15.33 (13.07-17.75)**	**0.19 (0.04-0.66)**
	IR[‡]	0.83 (0.27-1.93)	0.82 (0.30-1.78)	0.43 (0.09-1.26)	0.00 (0.00-0.46)	0.22 (0.03-0.80)
Chronic obstructive pulmonary disease	Events/At risk	22/5,816	29/6,056	25/5,682	20/5,689	22/5,668
	**CumI[†]**	**0.38 (0.25-0.57)**	**0.48 (0.33-0.68)**	**0.44 (0.29-0.64)**	**0.35 (0.22-0.54)**	**0.39 (0.25-0.58)**
	IR[‡]	0.49 (0.31-0.75)	0.63 (0.42-0.90)	0.57 (0.37-0.84)	0.46 (0.28-0.71)	0.51 (0.32-0.76)
Other chronic lung disease	Events/At risk	<10/728[⫴]	<10/814[⫴]	<10/795[⫴]	<10/939[⫴]	<10/1,095[⫴]
	**CumI[†]**	**1.10 (0.52-2.08)**	**0.61 (0.24-1.37)**	**0.38 (0.11-1.05)**	**0.53 (0.21-1.19)**	**0.82 (0.41-1.51)**
	IR[‡]	1.70 (0.73-3.35)	0.97 (0.32-2.27)	0.60 (0.12-1.74)	0.84 (0.27-1.96)	1.32 (0.60-2.51)
Heart failure	Events/At risk	57/9,960	52/10,679	44/10,386	36/10,862	43/11,017
	**CumI[†]**	**0.57 (0.44-0.74)**	**0.49 (0.37-0.63)**	**0.42 (0.31-0.56)**	**0.33 (0.24-0.46)**	**0.39 (0.29-0.52)**
	IR[‡]	0.77 (0.58-1.00)	0.65 (0.49-0.85)	0.57 (0.41-0.76)	0.44 (0.31-0.61)	0.52 (0.38-0.70)
Myocardial infarction (history)	Events/At risk	10/2,550	16/2,896	13/2,996	<10/3,188[⫴]	<10/3,214[⫴]
	**CumI[†]**	**0.39 (0.20-0.70)**	**0.55 (0.33-0.88)**	**0.43 (0.25-0.73)**	**0.22 (0.10-0.44)**	**0.22 (0.10-0.44)**
	IR[‡]	0.47 (0.23-0.87)	0.66 (0.38-1.08)	0.52 (0.27-0.88)	0.26 (0.10-0.53)	0.25 (0.10-0.53)
Hypertension	Events/At risk	126/16,082	112/17,721	92/17,208	85/18,371	91/18,932
	**CumI[†]**	**0.78 (0.66-0.93)**	**0.63 (0.52-0.76)**	**0.53 (0.43-0.65)**	**0.46 (0.37-0.57)**	**0.48 (0.39-0.59)**
	IR[‡]	0.91 (0.76-1.09)	0.73 (0.60-0.88)	0.62 (0.50-0.75)	0.54 (0.43-0.66)	0.56 (0.45-0.69)
Other valvular heart disease[‖]	Events/At risk	38/6,628	36/7,033	34/6,630	24/6,856	29/6,712
	**CumI[†]**	**0.57 (0.41-0.78)**	**0.51 (0.37-0.70)**	**0.51 (0.36-0.71)**	**0.35 (0.23-0.51)**	**0.43 (0.30-0.61)**
	IR[‡]	0.69 (0.49-0.95)	0.62 (0.44-0.86)	0.62 (0.43-0.87)	0.42 (0.27-0.63)	0.52 (0.35-0.75)
Peripheral artery disease	Events/At risk	<10/1,021[⫴]	11/1,206	<10/987[⫴]	<10/1,015[⫴]	<10/,982[⫴]
	**CumI[†]**	**0.69 (0.31-1.36)**	**0.91 (0.49-1.59)**	**0.61 (0.26-1.27)**	**0.49 (0.19-1.10)**	**0.71 (0.32-1.42)**
	IR[‡]	0.86 (0.34-1.77)	1.21 (0.61-2.17)	0.78 (0.29-1.70)	0.64 (0.21-1.49)	0.94 (0.38-1.94)
Liver diseases	Events/At risk	<10/1,044[⫴]	<10/1,053[⫴]	<10/1,033[⫴]	<10/1,049[⫴]	<10/1,036[⫴]
	**CumI[†]**	**0.86 (0.43-1.58)**	**0.28 (0.08-0.80)**	**0.48 (0.19-1.08)**	**0.29 (0.08-0.80)**	**0.39 (0.13-0.95)**
	IR[‡]	1.23 (0.56-2.34)	0.41 (0.08-1.20)	0.70 (0.23-1.63)	0.42 (0.09-1.23)	0.60 (0.16-1.53)

© 2023 Chen Q et al. *JAMA Network Open.*

Gastroesophageal reflux disease	Events/At risk	<10/117[‖]	<10/92[‖]	<10/113[‖]	<10/127[‖]	<10/111[‖]
	**CumI[†]**	**0.85 (0.08-4.27)**	**1.09 (0.09-5.34)**	**0.88 (0.08-4.41)**	**21.26 (14.60-28.76)**	**33.33 (24.71-42.17)**
	IR[‡]	1.00 (0.03-5.55)	1.33 (0.03-7.42)	1.10 (0.03-6.12)	0.00 (0.00-3.44)	0.00 (0.00-4.33)
Peptic ulcer disease	Events/At risk	<10/239[‖]	<10/244[‖]	<10/212[‖]	<10/226[‖]	<10/234[‖]
	**CumI[†]**	**0.42 (0.04-2.18)**	**0.41 (0.04-2.13)**	**0.47 (0.04-2.44)**	**0.88 (0.18-2.94)**	**0.43 (0.04-2.22)**
	IR[‡]	0.57 (0.01-3.15)	0.55 (0.01-3.06)	0.67 (0.02-3.74)	1.17 (0.14-4.23)	0.56 (0.01-3.12)
Chronic kidney diseases	Events/At risk	49/5,773	35/6,272	43/6,488	28/6,867	40/6,563
	**CumI[†]**	**0.85 (0.64-1.11)**	**0.56 (0.40-0.77)**	**0.66 (0.49-0.89)**	**0.41 (0.28-0.58)**	**0.61 (0.44-0.82)**
	IR[‡]	1.19 (0.88-1.58)	0.78 (0.54-1.09)	0.91 (0.66-1.23)	0.56 (0.37-0.81)	0.85 (0.61-1.16)
Anaemia	Events/At risk	34/3,520	35/3,860	29/4,011	20/4,385	31/4,535
	**CumI[†]**	**0.97 (0.68-1.33)**	**0.91 (0.64-1.25)**	**0.72 (0.50-1.03)**	**0.46 (0.29-0.69)**	**0.68 (0.48-0.96)**
	IR[‡]	1.29 (0.89-1.80)	1.22 (0.85-1.70)	0.98 (0.65-1.40)	0.62 (0.38-0.95)	0.93 (0.63-1.32)
Coagulopathy	Events/At risk	<10/736[‖]	10/1,012	13/962	<10/1,024[‖]	<10/1,165[‖]
	**CumI[†]**	**1.09 (0.52-2.06)**	**0.99 (0.51-1.76)**	**1.35 (0.76-2.25)**	**0.49 (0.19-1.09)**	**0.60 (0.27-1.20)**
	IR[‡]	1.52 (0.65-2.99)	1.36 (0.65-2.50)	1.92 (1.02-3.28)	0.71 (0.23-1.66)	0.88 (0.35-1.81)
Diabetes	Events/At risk	57/9,153	52/9,954	37/9,882	42/1,0231	44/10,287
	**CumI[†]**	**0.62 (0.48-0.80)**	**0.52 (0.40-0.68)**	**0.37 (0.27-0.51)**	**0.41 (0.30-0.55)**	**0.43 (0.32-0.57)**
	IR[‡]	0.76 (0.58-0.99)	0.64 (0.48-0.84)	0.46 (0.32-0.63)	0.50 (0.36-0.68)	0.53 (0.38-0.71)
Thyroid disease	Events/At risk	<10/1,397[‖]	<10/1,497[‖]	<10/1,403[‖]	<10/1,572[‖]	<10/1,561[‖]
	**CumI[†]**	**0.64 (0.32-1.19)**	**0.53 (0.26-1.02)**	**0.07 (0.01-0.40)**	**0.38 (0.16-0.80)**	**0.45 (0.20-0.90)**
	IR[‡]	0.76 (0.35-1.45)	0.63 (0.27-1.25)	0.08 (0.00-0.46)	0.44 (0.16-0.97)	0.54 (0.22-1.11)
Ischemic stroke (history)[#]	Events/At risk	14/1,593	13/1,732	13/1,672	<10/1,777[‖]	<10/1,668[‖]
	**CumI[†]**	**0.88 (0.51-1.44)**	**0.75 (0.42-1.25)**	**0.78 (0.44-1.30)**	**0.45 (0.22-0.86)**	**0.54 (0.27-1.00)**
	IR[‡]	1.11 (0.61-1.86)	0.93 (0.49-1.59)	0.97 (0.52-1.66)	0.56 (0.24-1.10)	0.66 (0.30-1.25)
Transient ischemic attack[#]	Events/At risk	<10/783[‖]	<10/825[‖]	<10/799[‖]	<10/855[‖]	<10/769[‖]
	**CumI[†]**	**0.13 (0.01-0.70)**	**0.61 (0.23-1.35)**	**0.50 (0.17-1.22)**	**1.05 (0.52-1.93)**	**0.65 (0.25-1.45)**
	IR[‡]	0.15 (0.00-0.83)	0.69 (0.22-1.61)	0.59 (0.16-1.50)	1.19 (0.54-2.25)	0.75 (0.24-1.76)
Other arterial thromboembolism[#]	Events/At risk	<10/593[‖]	<10/537[‖]	<10/506[‖]	<10/472[‖]	<10/396[‖]
	**CumI[†]**	**0.67 (0.23-1.64)**	**0.56 (0.16-1.54)**	**0.20 (0.02-1.06)**	**0.64 (0.18-1.75)**	**0.76 (0.21-2.08)**
	IR[‡]	0.85 (0.23-2.18)	0.70 (0.14-2.05)	0.24 (0.01-1.35)	0.81 (0.17-2.36)	0.97 (0.20-2.83)
Parkinson's disease	Events/At risk	<10/426[‖]	<10/505[‖]	<10/494[‖]	<10/549[‖]	<10/524[‖]
	**CumI[†]**	**0.70 (0.20-1.93)**	**0.59 (0.17-1.64)**	**0.20 (0.02-1.09)**	**0.73 (0.25-1.77)**	**0.95 (0.37-2.12)**
	IR[‡]	0.95 (0.20-2.78)	0.77 (0.16-2.24)	0.26 (0.01-1.45)	0.95 (0.26-2.43)	1.24 (0.40-2.89)
Alzheimer's disease	Events/At risk	<10/499[‖]	<10/563[‖]	<10/555[‖]	<10/567[‖]	<10/611[‖]
	**CumI[†]**	**1.20 (0.50-2.49)**	**0.53 (0.15-1.47)**	**0.54 (0.15-1.49)**	**1.23 (0.55-2.43)**	**0.49 (0.14-1.36)**
	IR[‡]	1.76 (0.65-3.83)	0.82 (0.17-2.40)	0.85 (0.17-2.48)	1.89 (0.76-3.90)	0.73 (0.15-2.15)
Autoimmune disease	Events/At risk	<10/105[‖]	<10/104[‖]	<10/117[‖]	<10/154[‖]	<10/139[‖]

© 2023 Chen Q et al. *JAMA Network Open.*

	**CumI[†]**	**28.57 (20.25-37.43)**	**28.85 (20.45-37.76)**	**24.79 (17.36-32.92)**	**30.52 (23.41-37.90)**	**0.72 (0.06-3.63)**
	IR[‡]	0.00 (0.00-4.55)	0.00 (0.00-4.66)	0.00 (0.00-3.84)	0.00 (0.00-3.06)	0.91 (0.02-5.10)
Systemic connective tissue disorders	Events/At risk	<10/465[‖]	<10/597[‖]	<10/538[‖]	<10/525[‖]	<10/638[‖]
	**CumI[†]**	**0.65 (0.18-1.77)**	**0.34 (0.07-1.15)**	**0.93 (0.36-2.06)**	**0.76 (0.26-1.85)**	**0.47 (0.13-1.30)**
	IR[‡]	0.82 (0.17-2.39)	0.44 (0.05-1.57)	1.13 (0.37-2.63)	0.98 (0.27-2.50)	0.61 (0.13-1.78)
Venous thromboembolism[#]	Events/At risk	<10/833[‖]	<10/823[‖]	<10/852[‖]	<10/996[‖]	<10/910[‖]
	**CumI[†]**	**0.48 (0.16-1.17)**	**0.49 (0.17-1.19)**	**0.82 (0.37-1.63)**	**0.70 (0.32-1.40)**	**0.88 (0.42-1.67)**
	IR[‡]	0.66 (0.18-1.70)	0.64 (0.17-1.63)	1.13 (0.45-2.32)	0.96 (0.39-1.98)	1.20 (0.52-2.36)
Major bleeding[#]	Events/At risk	34/2,234	30/2,401	21/2,404	18/2,568	15/2,495
	**CumI[†]**	**1.52 (1.08-2.10)**	**1.25 (0.86-1.76)**	**0.87 (0.56-1.31)**	**0.70 (0.43-1.09)**	**0.60 (0.35-0.97)**
	IR[‡]	1.94 (1.34-2.71)	1.59 (1.07-2.27)	1.13 (0.70-1.73)	0.89 (0.53-1.40)	0.77 (0.43-1.27)
Malignant tumour	Events/At risk	26/4,359	25/4,657	19/4,663	15/4,818	18/4,967
	**CumI[†]**	**0.60 (0.40-0.86)**	**0.54 (0.36-0.78)**	**0.41 (0.26-0.63)**	**0.31 (0.18-0.51)**	**0.36 (0.22-0.56)**
	IR[‡]	0.95 (0.62-1.40)	0.84 (0.54-1.24)	0.64 (0.39-1.01)	0.49 (0.27-0.81)	0.58 (0.34-0.91)
≥1 hospitalization within the prior 4 years	Events/At risk	230/36,748	213/38,992	180/37,989	148/39,362	147/37,935
	**CumI[†]**	**0.63 (0.55-0.71)**	**0.55 (0.48-0.62)**	**0.47 (0.41-0.55)**	**0.38 (0.32-0.44)**	**0.39 (0.33-0.45)**
	IR[‡]	0.74 (0.65-0.84)	0.64 (0.56-0.73)	0.55 (0.47-0.64)	0.44 (0.37-0.51)	0.45 (0.38-0.53)
Without any hospitalization within the prior 4 years	Events/At risk	102/18,720	100/21,915	79/21,666	75/22,643	100/23,404
	**CumI[†]**	**0.54 (0.45-0.66)**	**0.46 (0.37-0.55)**	**0.36 (0.29-0.45)**	**0.33 (0.26-0.41)**	**0.43 (0.35-0.52)**
	IR[‡]	0.61 (0.50-0.74)	0.51 (0.42-0.62)	0.41 (0.32-0.51)	0.37 (0.29-0.46)	0.48 (0.39-0.58)
Survived until discharge from the index hospitalization	Events/At risk	311/53,158	299/58,366	246/57,187	208/59,378	234/58,548
	**CumI[†]**	**0.59 (0.52-0.65)**	**0.51 (0.46-0.57)**	**0.43 (0.38-0.49)**	**0.35 (0.31-0.40)**	**0.40 (0.35-0.45)**
	IR[‡]	0.65 (0.58-0.73)	0.57 (0.50-0.63)	0.47 (0.42-0.54)	0.38 (0.33-0.44)	0.44 (0.38-0.50)
Main reason for admission to the index hospitalization						
Atrial fibrillation	Events/At risk	72/19,496	68/23,257	55/23,047	56/23,782	55/22,877
	**CumI[†]**	**0.37 (0.29-0.46)**	**0.29 (0.23-0.37)**	**0.24 (0.18-0.31)**	**0.24 (0.18-0.30)**	**0.24 (0.18-0.31)**
	IR[‡]	0.38 (0.30-0.48)	0.30 (0.24-0.38)	0.25 (0.19-0.32)	0.24 (0.18-0.32)	0.25 (0.19-0.32)
Not primarily for atrial fibrillation	Events/At risk	260/35,972	245/37,650	204/36,608	167/38,223	192/38,462
	**CumI[†]**	**0.72 (0.64-0.81)**	**0.65 (0.57-0.74)**	**0.56 (0.49-0.64)**	**0.44 (0.37-0.51)**	**0.50 (0.43-0.57)**
	IR[‡]	0.89 (0.79-1.01)	0.80 (0.71-0.91)	0.69 (0.59-0.79)	0.54 (0.46-0.62)	0.61 (0.53-0.71)
Heart failure	Events/At risk	22/4,566	24/4,906	20/4,767	15/4,872	18/4,905
	**CumI[†]**	**0.48 (0.31-0.72)**	**0.49 (0.32-0.72)**	**0.42 (0.27-0.64)**	**0.31 (0.18-0.50)**	**0.37 (0.23-0.57)**
	IR[‡]	0.63 (0.40-0.96)	0.64 (0.41-0.95)	0.55 (0.34-0.85)	0.40 (0.23-0.66)	0.48 (0.29-0.76)
Ischemic heart diseases	Events/At risk	19/4,504	23/4,544	18/4,469	11/4,714	15/4,831
	**CumI[†]**	**0.42 (0.26-0.65)**	**0.51 (0.33-0.75)**	**0.40 (0.25-0.63)**	**0.23 (0.13-0.41)**	**0.31 (0.18-0.50)**
	IR[‡]	0.46 (0.28-0.72)	0.56 (0.35-0.84)	0.44 (0.26-0.69)	0.25 (0.13-0.45)	0.33 (0.19-0.55)

© 2023 Chen Q et al. *JAMA Network Open.*

Respiratory diseases	Events/At risk	16/3,880	30/4,863	28/4,641	10/5,038	25/5,244
	**CumI†**	**0.41 (0.25-0.66)**	**0.62 (0.43-0.87)**	**0.60 (0.41-0.86)**	**0.20 (0.10-0.36)**	**0.48 (0.32-0.70)**
	IR‡	0.57 (0.32-0.92)	0.85 (0.57-1.21)	0.82 (0.55-1.19)	0.27 (0.13-0.50)	0.65 (0.42-0.97)
Injury/poisoning	Events/At risk	48/2,970	45/3,117	43/3,137	41/3,207	42/3,462
	**CumI†**	**1.62 (1.21-2.12)**	**1.44 (1.07-1.91)**	**1.37 (1.01-1.83)**	**1.28 (0.93-1.72)**	**1.21 (0.89-1.62)**
	IR‡	2.11 (1.56-2.80)	1.88 (1.37-2.52)	1.78 (1.29-2.40)	1.65 (1.18-2.23)	1.57 (1.13-2.13)
Symptoms/signs/abnormal laboratory findings**	Events/At risk	34/2,922	22/2,997	12/2,761	11/2,655	15/2,483
	**CumI†**	**1.16 (0.82-1.61)**	**0.73 (0.48-1.09)**	**0.43 (0.24-0.74)**	**0.41 (0.22-0.72)**	**0.60 (0.36-0.98)**
	IR‡	1.38 (0.95-1.92)	0.85 (0.53-1.28)	0.50 (0.26-0.87)	0.46 (0.23-0.83)	0.68 (0.38-1.12)
Neoplasms	Events/At risk	17/2,749	13/2,794	<10/2,722‖	10/2,868	<10/2,929‖
	**CumI†**	**0.62 (0.38-0.97)**	**0.47 (0.26-0.78)**	**0.33 (0.17-0.61)**	**0.35 (0.18-0.63)**	**0.24 (0.11-0.48)**
	IR‡	0.93 (0.54-1.49)	0.69 (0.37-1.18)	0.50 (0.23-0.95)	0.52 (0.25-0.96)	0.36 (0.14-0.74)
Digestive diseases	Events/At risk	12/2,477	10/2,470	13/2,297	10/2,515	<10/2,396‖
	**CumI†**	**0.48 (0.27-0.83)**	**0.40 (0.21-0.73)**	**0.57 (0.32-0.95)**	**0.40 (0.21-0.71)**	**0.33 (0.16-0.64)**
	IR‡	0.59 (0.31-1.04)	0.50 (0.24-0.91)	0.70 (0.37-1.20)	0.48 (0.23-0.89)	0.41 (0.18-0.81)
Other heart valve disorders§§	Events/At risk	<10/1,477‖	12/1,498	<10/1,630‖	<10/1,623‖	<10/1,534‖
	**CumI†**	**0.41 (0.17-0.85)**	**0.80 (0.44-1.36)**	**0.37 (0.16-0.78)**	**0.25 (0.09-0.61)**	**0.52 (0.25-1.00)**
	IR‡	0.44 (0.16-0.95)	0.86 (0.45-1.51)	0.39 (0.14-0.86)	0.26 (0.07-0.67)	0.55 (0.24-1.08)
Genitourinary diseases	Events/At risk	<10/1,469‖	15/1,531	<10/1,569‖	<10/1,672‖	<10/1,707‖
	**CumI†**	**0.48 (0.22-0.95)**	**0.98 (0.58-1.58)**	**0.38 (0.16-0.80)**	**0.54 (0.27-0.99)**	**0.53 (0.26-0.97)**
	IR‡	0.59 (0.24-1.21)	1.24 (0.69-2.05)	0.48 (0.17-1.04)	0.67 (0.31-1.27)	0.67 (0.30-1.26)
Infectious/parasitic diseases	Events/At risk	10/1,059	<10/1,069‖	<10/1,037‖	<10/1,204‖	<10/1,189‖
	**CumI†**	**0.94 (0.49-1.68)**	**0.19 (0.04-0.65)**	**0.48 (0.19-1.08)**	**0.58 (0.26-1.16)**	**0.42 (0.16-0.94)**
	IR‡	1.35 (0.65-2.48)	0.27 (0.03-0.97)	0.65 (0.21-1.51)	0.79 (0.32-1.62)	0.57 (0.18-1.33)
Endocrine/nutritional/metabolic diseases	Events/At risk	<10/680‖	<10/670‖	<10/616‖	<10/634‖	<10/721‖
	**CumI†**	**0.88 (0.37-1.84)**	**0.45 (0.13-1.24)**	**0.32 (0.07-1.11)**	**0.47 (0.13-1.31)**	**0.69 (0.27-1.55)**
	IR‡	1.09 (0.40-2.36)	0.58 (0.12-1.69)	0.43 (0.05-1.55)	0.60 (0.12-1.75)	0.90 (0.29-2.09)
Diseases of blood/blood-forming organs	Events/At risk	<10/492‖	<10/503‖	<10/465‖	<10/514‖	<10/483‖
	**CumI†**	**1.02 (0.39-2.25)**	**0.99 (0.38-2.20)**	**0.86 (0.29-2.08)**	**0.19 (0.02-1.05)**	**0.21 (0.02-1.11)**
	IR‡	1.28 (0.42-2.98)	1.28 (0.42-3.00)	1.09 (0.30-2.78)	0.25 (0.01-1.38)	0.27 (0.01-1.48)
Neurological disorders##	Events/At risk	<10/491‖	<10/455‖	<10/402‖	<10/441‖	<10/405‖
	**CumI†**	**0.41 (0.08-1.39)**	**1.76 (0.83-3.31)**	**1.24 (0.47-2.74)**	**2.04 (1.01-3.71)**	**1.48 (0.62-3.06)**
	IR‡	0.48 (0.06-1.73)	2.21 (0.95-4.36)	1.51 (0.49-3.53)	2.45 (1.12-4.65)	1.85 (0.68-4.03)

† Cumulative incidence (%, 95% confidence interval), estimated by the cumulative incidence competing risk method

‡ Incidence rate (per 100-person-year, 95% confidence interval)

* Defined as ≥2 (outpatient) prescription records of antiplatelet agents within six months (*i.e.*, 183 days) before the index dates (exclusive)

§ Identified within the index hospitalizations unless otherwise stated

© 2023 Chen Q et al. *JAMA Network Open.*

|| Except for rheumatic mitral stenosis and mechanical heart valves
Identified within the prior four years (except for venous thromboembolism which was identified within the prior six months)
** Except for abnormalities of heart beat (ICD-10 code: R00) and cardiac murmurs and other cardiac sounds (ICD-10 code: R01)
§§ Except for endocarditis, valve unspecified, in diseases classified elsewhere (ICD-10 code: I398)
Except for transient cerebral ischaemic attacks and related syndromes (ICD-10 code: G45) and vascular syndromes of brain in cerebrovascular diseases (ICD-10 code: G46)
||| Exact result was masked to prevent potential individual or group disclosure.
Abbreviations: CumI, cumulative incidence; IR, incidence rate.

© 2023 Chen Q et al. *JAMA Network Open.*

eTable 23. Subgroup Analysis of Risk of Gastrointestinal Bleeding Within the 1-Year Follow-up After Incident Nonvalvular Atrial Fibrillation Diagnosis

Subgroup	Cohort	2014	2015	2016	2017	2018
Sex						
Male	Events/At risk	305/31,216	314/33,862	316/33,589	363/35,132	354/35,080
	**CumI**†	**0.98 (0.87-1.09)**	**0.93 (0.83-1.03)**	**0.94 (0.84-1.05)**	**1.03 (0.93-1.14)**	**1.01 (0.91-1.12)**
	IR‡	1.12 (1.00-1.26)	1.06 (0.95-1.18)	1.07 (0.96-1.20)	1.17 (1.06-1.30)	1.15 (1.03-1.28)
Female	Events/At risk	243/24,306	257/27,120	250/26,127	252/26,939	261/26,308
	**CumI**†	**1.00 (0.88-1.13)**	**0.95 (0.84-1.07)**	**0.96 (0.84-1.08)**	**0.94 (0.83-1.06)**	**0.99 (0.88-1.12)**
	IR‡	1.18 (1.04-1.34)	1.11 (0.98-1.26)	1.12 (0.98-1.26)	1.10 (0.97-1.24)	1.16 (1.03-1.31)
Immigration background						
Native Dutch	Events/At risk	472/48,888	492/53,681	479/52,184	532/54,264	511/53,648
	**CumI**†	**0.97 (0.88-1.06)**	**0.92 (0.84-1.00)**	**0.92 (0.84-1.00)**	**0.98 (0.90-1.07)**	**0.95 (0.87-1.04)**
	IR‡	1.12 (1.03-1.23)	1.06 (0.97-1.16)	1.06 (0.97-1.16)	1.13 (1.04-1.23)	1.10 (1.01-1.20)
First/Second-generation immigrants	Events/At risk	76/6,634	79/7,301	87/7,532	83/7,807	104/7,740
	**CumI**†	**1.15 (0.91-1.42)**	**1.08 (0.86-1.34)**	**1.16 (0.93-1.42)**	**1.06 (0.85-1.31)**	**1.34 (1.11-1.62)**
	IR‡	1.32 (1.04-1.66)	1.24 (0.98-1.55)	1.32 (1.06-1.63)	1.22 (0.97-1.51)	1.53 (1.25-1.85)
CHA_2DS_2-VASc score						
0	Events/At risk	15/4,037	20/4,781	18/4,783	21/4,752	13/4,584
	**CumI**†	**0.37 (0.22-0.60)**	**0.42 (0.27-0.64)**	**0.38 (0.23-0.59)**	**0.44 (0.28-0.67)**	**0.28 (0.16-0.48)**
	IR‡	0.38 (0.21-0.63)	0.43 (0.26-0.67)	0.39 (0.23-0.61)	0.45 (0.28-0.69)	0.29 (0.16-0.50)
1	Events/At risk	52/7,405	57/8,286	54/8,137	38/8,556	49/8,546
	**CumI**†	**0.70 (0.53-0.91)**	**0.69 (0.53-0.89)**	**0.66 (0.51-0.86)**	**0.44 (0.32-0.60)**	**0.57 (0.43-0.75)**
	IR‡	0.74 (0.55-0.97)	0.73 (0.55-0.94)	0.70 (0.53-0.91)	0.47 (0.33-0.64)	0.61 (0.45-0.80)
≥2	Events/At risk	481/44,080	494/47,915	494/46,796	556/48,763	553/48,258
	**CumI**†	**1.09 (1.00-1.19)**	**1.03 (0.94-1.12)**	**1.06 (0.97-1.15)**	**1.14 (1.05-1.24)**	**1.15 (1.05-1.24)**
	IR‡	1.31 (1.19-1.43)	1.23 (1.12-1.34)	1.25 (1.14-1.37)	1.35 (1.24-1.47)	1.36 (1.25-1.48)
HAS-BLED score						
0	Events/At risk	21/5,034	33/6,083	21/6,227	27/6,506	25/6,531
	**CumI**†	**0.42 (0.27-0.63)**	**0.54 (0.38-0.75)**	**0.34 (0.22-0.51)**	**0.42 (0.28-0.60)**	**0.38 (0.26-0.56)**
	IR‡	0.43 (0.27-0.66)	0.56 (0.39-0.79)	0.35 (0.21-0.53)	0.43 (0.28-0.62)	0.39 (0.25-0.58)
1	Events/At risk	138/17,919	146/20,468	164/20,427	163/21,421	172/21,434
	**CumI**†	**0.77 (0.65-0.91)**	**0.71 (0.61-0.84)**	**0.80 (0.69-0.93)**	**0.76 (0.65-0.88)**	**0.80 (0.69-0.93)**
	IR‡	0.87 (0.73-1.02)	0.80 (0.68-0.94)	0.90 (0.77-1.05)	0.85 (0.72-0.99)	0.90 (0.77-1.04)
2	Events/At risk	207/19,894	225/21,594	209/20,711	240/21,362	218/21,199
	**CumI**†	**1.04 (0.91-1.19)**	**1.04 (0.91-1.18)**	**1.01 (0.88-1.15)**	**1.12 (0.99-1.27)**	**1.03 (0.90-1.17)**
	IR‡	1.22 (1.06-1.40)	1.21 (1.06-1.38)	1.18 (1.02-1.35)	1.31 (1.15-1.49)	1.21 (1.05-1.38)
≥3	Events/At risk	182/12,675	167/12,837	172/12,351	185/12,782	200/12,224

© 2023 Chen Q et al. *JAMA Network Open.*

	**CumI[†]**	**1.44 (1.24-1.65)**	**1.30 (1.12-1.51)**	**1.39 (1.20-1.61)**	**1.45 (1.25-1.67)**	**1.64 (1.42-1.87)**
	IR[‡]	1.82 (1.57-2.11)	1.66 (1.42-1.93)	1.77 (1.51-2.05)	1.85 (1.59-2.14)	2.09 (1.81-2.40)
Use of antithrombotic agents						
Pre-existing chronic use of antiplatelet agents[*]	Events/At risk	190/15,981	177/16,234	187/15,719	192/16,629	196/16,209
	**CumI[†]**	**1.19 (1.03-1.37)**	**1.09 (0.94-1.26)**	**1.19 (1.03-1.37)**	**1.15 (1.00-1.33)**	**1.21 (1.05-1.39)**
	IR[‡]	1.40 (1.21-1.61)	1.29 (1.10-1.49)	1.41 (1.21-1.62)	1.36 (1.17-1.56)	1.42 (1.23-1.64)
≥1 oral anticoagulant prescription within the 1-year follow-up	Events/At risk	386/37,674	421/44,224	424/43,590	484/44,914	455/44,689
	**CumI[†]**	**1.02 (0.93-1.13)**	**0.95 (0.86-1.05)**	**0.97 (0.88-1.07)**	**1.08 (0.99-1.18)**	**1.02 (0.93-1.11)**
	IR[‡]	1.09 (0.99-1.21)	1.01 (0.92-1.11)	1.03 (0.94-1.14)	1.15 (1.05-1.25)	1.09 (0.99-1.19)
Comorbidities (or medical history)[§]						
Asthma	Events/At risk	<10/683[|||]	<10/835[|||]	<10/787[|||]	11/904	<10/1,039[|||]
	**CumI[†]**	**0.88 (0.37-1.83)**	**0.60 (0.23-1.34)**	**0.51 (0.17-1.24)**	**1.22 (0.65-2.11)**	**0.87 (0.43-1.59)**
	IR[‡]	0.99 (0.37-2.17)	0.68 (0.22-1.59)	0.57 (0.16-1.47)	1.38 (0.69-2.47)	1.00 (0.46-1.90)
Chronic obstructive pulmonary disease	Events/At risk	72/5,794	58/6,053	76/5,697	73/5,697	79/5,663
	**CumI[†]**	**1.24 (0.98-1.55)**	**0.96 (0.74-1.23)**	**1.33 (1.06-1.66)**	**1.28 (1.01-1.60)**	**1.40 (1.11-1.73)**
	IR[‡]	1.63 (1.27-2.05)	1.26 (0.96-1.63)	1.74 (1.37-2.18)	1.69 (1.33-2.13)	1.83 (1.45-2.28)
Other chronic lung disease	Events/At risk	<10/743[|||]	12/832	12/804	13/968	13/1,109
	**CumI[†]**	**0.27 (0.06-0.93)**	**1.44 (0.79-2.44)**	**1.49 (0.82-2.53)**	**1.34 (0.76-2.23)**	**1.17 (0.66-1.95)**
	IR[‡]	0.42 (0.05-1.51)	2.33 (1.21-4.07)	2.39 (1.23-4.17)	2.17 (1.16-3.71)	1.90 (1.01-3.25)
Heart failure	Events/At risk	92/9,947	114/10,676	121/10,382	121/10,850	144/11,009
	**CumI[†]**	**0.92 (0.75-1.13)**	**1.07 (0.89-1.28)**	**1.17 (0.97-1.39)**	**1.12 (0.93-1.33)**	**1.31 (1.11-1.53)**
	IR[‡]	1.25 (1.01-1.54)	1.43 (1.18-1.72)	1.57 (1.30-1.87)	1.50 (1.24-1.79)	1.76 (1.48-2.07)
Myocardial infarction (history)	Events/At risk	34/2,546	41/2,891	46/2,993	48/3,188	47/3,212
	**CumI[†]**	**1.34 (0.94-1.84)**	**1.42 (1.03-1.90)**	**1.54 (1.14-2.03)**	**1.51 (1.13-1.98)**	**1.46 (1.09-1.93)**
	IR[‡]	1.62 (1.12-2.26)	1.71 (1.23-2.32)	1.84 (1.35-2.46)	1.79 (1.32-2.37)	1.73 (1.27-2.30)
Hypertension	Events/At risk	191/16,134	190/17,816	164/17,292	199/18,473	222/19,013
	**CumI[†]**	**1.18 (1.03-1.36)**	**1.07 (0.92-1.23)**	**0.95 (0.81-1.10)**	**1.08 (0.94-1.23)**	**1.17 (1.02-1.33)**
	IR[‡]	1.39 (1.20-1.60)	1.25 (1.08-1.44)	1.10 (0.94-1.28)	1.26 (1.09-1.45)	1.37 (1.19-1.56)
Other valvular heart disease[||]	Events/At risk	77/6,611	88/7,024	81/6,634	93/6,852	85/6,698
	**CumI[†]**	**1.16 (0.93-1.45)**	**1.25 (1.01-1.53)**	**1.22 (0.98-1.51)**	**1.36 (1.10-1.65)**	**1.27 (1.02-1.56)**
	IR[‡]	1.41 (1.12-1.77)	1.54 (1.23-1.90)	1.49 (1.18-1.85)	1.66 (1.34-2.03)	1.55 (1.24-1.92)
Peripheral artery disease	Events/At risk	18/1,022	19/1,208	12/993	17/1,022	16/982
	**CumI[†]**	**1.76 (1.08-2.72)**	**1.57 (0.98-2.40)**	**1.21 (0.66-2.05)**	**1.66 (1.01-2.60)**	**1.63 (0.97-2.58)**
	IR[‡]	2.22 (1.31-3.51)	2.12 (1.28-3.31)	1.56 (0.81-2.73)	2.17 (1.27-3.48)	2.17 (1.24-3.53)
Liver diseases	Events/At risk	19/1,043	16/1,046	15/1,029	20/1,040	26/1,023
	**CumI[†]**	**1.82 (1.14-2.78)**	**1.53 (0.91-2.42)**	**1.46 (0.85-2.34)**	**1.92 (1.22-2.90)**	**2.54 (1.70-3.65)**
	IR[‡]	2.63 (1.58-4.10)	2.23 (1.27-3.61)	2.13 (1.19-3.51)	2.86 (1.75-4.42)	4.01 (2.62-5.88)

© 2023 Chen Q et al. *JAMA Network Open.*

Gastroesophageal reflux disease	Events/At risk	<10/106[‖]	<10/85[‖]	<10/95[‖]	<10/116[‖]	<10/103[‖]
	**CumI[†]**	**2.83 (0.76-7.41)**	**1.18 (0.10-5.75)**	**6.32 (2.57-12.45)**	**4.31 (1.60-9.16)**	**5.83 (2.37-11.53)**
	IR[‡]	3.36 (0.69-9.81)	1.44 (0.04-8.03)	8.40 (3.08-18.28)	5.30 (1.72-12.36)	8.04 (2.95-17.49)
Peptic ulcer disease	Events/At risk	10/175	16/173	15/152	<10/147[‖]	10/156
	**CumI[†]**	**5.71 (2.92-9.85)**	**9.25 (5.51-14.15)**	**9.87 (5.77-15.25)**	**4.76 (2.10-9.08)**	**6.41 (3.27-11.01)**
	IR[‡]	8.26 (3.96-15.19)	13.63 (7.79-22.14)	15.76 (8.82-26.00)	6.95 (2.80-14.33)	9.14 (4.38-16.82)
Chronic kidney diseases	Events/At risk	96/5,756	86/6,265	84/6,474	93/6,850	107/6,540
	**CumI[†]**	**1.67 (1.36-2.02)**	**1.37 (1.11-1.68)**	**1.30 (1.04-1.60)**	**1.36 (1.10-1.65)**	**1.64 (1.35-1.97)**
	IR[‡]	2.37 (1.92-2.89)	1.94 (1.55-2.40)	1.80 (1.44-2.23)	1.88 (1.52-2.31)	2.30 (1.88-2.78)
Anaemia	Events/At risk	77/3,446	93/3,784	92/3,944	110/4,293	112/4,453
	**CumI[†]**	**2.23 (1.78-2.77)**	**2.46 (2.00-2.99)**	**2.33 (1.90-2.84)**	**2.56 (2.12-3.07)**	**2.52 (2.09-3.01)**
	IR[‡]	3.02 (2.39-3.78)	3.37 (2.72-4.12)	3.19 (2.58-3.92)	3.52 (2.89-4.24)	3.47 (2.86-4.18)
Coagulopathy	Events/At risk	14/729	11/1,011	13/962	18/1,027	17/1,134
	**CumI[†]**	**1.92 (1.10-3.12)**	**1.09 (0.58-1.89)**	**1.35 (0.76-2.25)**	**1.75 (1.08-2.70)**	**1.50 (0.91-2.34)**
	IR[‡]	2.72 (1.49-4.57)	1.51 (0.75-2.70)	1.95 (1.04-3.34)	2.59 (1.53-4.09)	2.22 (1.29-3.55)
Diabetes	Events/At risk	105/9,164	95/9,956	105/9,887	120/10,249	119/10,277
	**CumI[†]**	**1.15 (0.94-1.38)**	**0.95 (0.78-1.16)**	**1.06 (0.87-1.28)**	**1.17 (0.98-1.39)**	**1.16 (0.96-1.38)**
	IR[‡]	1.42 (1.16-1.71)	1.17 (0.95-1.43)	1.30 (1.07-1.58)	1.44 (1.20-1.73)	1.44 (1.19-1.72)
Thyroid disease	Events/At risk	11/1,397	16/1,493	<10/1,400[‖]	10/1,568	12/1,566
	**CumI[†]**	**0.79 (0.42-1.37)**	**1.07 (0.64-1.70)**	**0.64 (0.32-1.18)**	**0.64 (0.33-1.14)**	**0.77 (0.42-1.31)**
	IR[‡]	0.94 (0.47-1.68)	1.28 (0.73-2.07)	0.75 (0.34-1.42)	0.75 (0.36-1.37)	0.92 (0.48-1.61)
Ischemic stroke (history)[#]	Events/At risk	15/1,602	16/1,754	14/1,677	21/1,783	22/1,675
	**CumI[†]**	**0.94 (0.55-1.51)**	**0.91 (0.55-1.45)**	**0.83 (0.48-1.37)**	**1.18 (0.75-1.77)**	**1.31 (0.85-1.95)**
	IR[‡]	1.19 (0.66-1.96)	1.14 (0.65-1.85)	1.04 (0.57-1.75)	1.47 (0.91-2.25)	1.62 (1.01-2.45)
Transient ischemic attack[#]	Events/At risk	<10/789[‖]	<10/830[‖]	15/802	14/854	12/775
	**CumI[†]**	**0.76 (0.32-1.59)**	**0.96 (0.46-1.83)**	**1.87 (1.10-3.00)**	**1.64 (0.94-2.67)**	**1.55 (0.85-2.62)**
	IR[‡]	0.90 (0.33-1.96)	1.11 (0.48-2.18)	2.21 (1.23-3.64)	1.86 (1.02-3.13)	1.80 (0.93-3.14)
Other arterial thromboembolism[#]	Events/At risk	14/592	10/539	<10/504[‖]	<10/472[‖]	<10/395[‖]
	**CumI[†]**	**2.36 (1.36-3.84)**	**1.86 (0.95-3.28)**	**1.19 (0.50-2.47)**	**1.27 (0.53-2.63)**	**1.52 (0.63-3.13)**
	IR[‡]	3.03 (1.66-5.08)	2.35 (1.13-4.32)	1.47 (0.54-3.19)	1.63 (0.60-3.55)	1.95 (0.72-4.25)
Parkinson's disease	Events/At risk	<10/428[‖]	<10/513[‖]	<10/493[‖]	<10/554[‖]	<10/524[‖]
	**CumI[†]**	**0.47 (0.10-1.58)**	**0.97 (0.37-2.16)**	**1.62 (0.77-3.06)**	**0.54 (0.15-1.50)**	**0.95 (0.37-2.12)**
	IR[‡]	0.64 (0.08-2.30)	1.27 (0.41-2.97)	2.10 (0.91-4.15)	0.71 (0.15-2.07)	1.25 (0.40-2.91)
Alzheimer's disease	Events/At risk	<10/498[‖]	<10/565[‖]	<10/554[‖]	<10/569[‖]	<10/612[‖]
	**CumI[†]**	**0.80 (0.27-1.95)**	**0.88 (0.34-1.97)**	**0.54 (0.15-1.50)**	**0.88 (0.34-1.95)**	**1.14 (0.51-2.26)**
	IR[‡]	1.18 (0.32-3.01)	1.37 (0.44-3.20)	0.85 (0.18-2.48)	1.35 (0.44-3.15)	1.72 (0.69-3.54)
Autoimmune disease	Events/At risk	<10/106[‖]	<10/105[‖]	<10/117[‖]	<10/154[‖]	<10/139[‖]

© 2023 Chen Q et al. *JAMA Network Open.*

	**CumI[†]**	**0.94 (0.08-4.68)**	**0.95 (0.08-4.72)**	**0.85 (0.08-4.26)**	**1.95 (0.53-5.18)**	**0.72 (0.06-3.63)**
	IR[‡]	1.25 (0.03-6.95)	1.28 (0.03-7.12)	1.05 (0.03-5.86)	2.52 (0.52-7.38)	0.92 (0.02-5.10)
Systemic connective tissue disorders	Events/At risk	<10/463[‖]	<10/594[‖]	<10/535[‖]	<10/524[‖]	15/637
	**CumI[†]**	**1.08 (0.41-2.39)**	**1.01 (0.42-2.10)**	**0.75 (0.25-1.81)**	**0.95 (0.37-2.12)**	**2.35 (1.38-3.76)**
	IR[‡]	1.38 (0.45-3.22)	1.32 (0.49-2.88)	0.91 (0.25-2.32)	1.23 (0.40-2.88)	3.11 (1.74-5.13)
Venous thromboembolism[#]	Events/At risk	<10/838[‖]	11/828	10/850	15/991	10/911
	**CumI[†]**	**0.95 (0.45-1.81)**	**1.33 (0.71-2.30)**	**1.18 (0.61-2.09)**	**1.51 (0.89-2.43)**	**1.10 (0.57-1.95)**
	IR[‡]	1.32 (0.57-2.61)	1.77 (0.88-3.17)	1.61 (0.77-2.96)	2.08 (1.17-3.43)	1.50 (0.72-2.77)
Major bleeding[#]	Events/At risk	53/2,239	64/2,418	59/2,390	86/2,570	80/2,495
	**CumI[†]**	**2.37 (1.80-3.06)**	**2.65 (2.06-3.34)**	**2.47 (1.90-3.15)**	**3.35 (2.70-4.09)**	**3.21 (2.57-3.95)**
	IR[‡]	3.07 (2.30-4.01)	3.42 (2.64-4.37)	3.25 (2.47-4.19)	4.32 (3.46-5.34)	4.20 (3.33-5.23)
Malignant tumour	Events/At risk	55/4,342	65/4,638	46/4,651	72/4,805	62/4,950
	**CumI[†]**	**1.27 (0.97-1.63)**	**1.40 (1.09-1.77)**	**0.99 (0.74-1.31)**	**1.50 (1.18-1.87)**	**1.25 (0.97-1.59)**
	IR[‡]	2.03 (1.53-2.65)	2.21 (1.70-2.81)	1.57 (1.15-2.10)	2.37 (1.86-2.99)	2.01 (1.54-2.58)
≥1 hospitalization within the prior 4 years	Events/At risk	414/36,758	407/39,032	398/37,987	442/39,370	456/37,936
	**CumI[†]**	**1.13 (1.02-1.24)**	**1.04 (0.95-1.15)**	**1.05 (0.95-1.15)**	**1.12 (1.02-1.23)**	**1.20 (1.10-1.32)**
	IR[‡]	1.34 (1.21-1.47)	1.23 (1.11-1.35)	1.23 (1.11-1.35)	1.32 (1.20-1.44)	1.41 (1.29-1.55)
Without any hospitalization within the prior 4 years	Events/At risk	134/18,764	164/21,950	168/21,729	173/22,701	159/23,452
	**CumI[†]**	**0.71 (0.60-0.84)**	**0.75 (0.64-0.87)**	**0.77 (0.66-0.90)**	**0.76 (0.66-0.88)**	**0.68 (0.58-0.79)**
	IR[‡]	0.80 (0.67-0.95)	0.84 (0.72-0.98)	0.87 (0.74-1.01)	0.85 (0.73-0.99)	0.76 (0.65-0.89)
Survived until discharge from the index hospitalization	Events/At risk	540/53,129	559/58,381	553/57,179	597/59,377	603/58,543
	**CumI[†]**	**1.02 (0.93-1.10)**	**0.96 (0.88-1.04)**	**0.97 (0.89-1.05)**	**1.01 (0.93-1.09)**	**1.03 (0.95-1.11)**
	IR[‡]	1.13 (1.04-1.23)	1.06 (0.98-1.15)	1.07 (0.98-1.16)	1.11 (1.02-1.20)	1.14 (1.05-1.23)
Main reason for admission to the index hospitalization						
Atrial fibrillation	Events/At risk	137/19,496	134/23,257	140/23,047	161/23,782	134/22,877
	**CumI[†]**	**0.70 (0.59-0.83)**	**0.58 (0.49-0.68)**	**0.61 (0.51-0.71)**	**0.68 (0.58-0.79)**	**0.59 (0.49-0.69)**
	IR[‡]	0.73 (0.61-0.87)	0.60 (0.50-0.71)	0.63 (0.53-0.74)	0.70 (0.60-0.82)	0.61 (0.51-0.72)
Not primarily for atrial fibrillation	Events/At risk	411/36,026	437/37,725	426/36,669	454/38,289	481/38,511
	**CumI[†]**	**1.14 (1.04-1.25)**	**1.16 (1.05-1.27)**	**1.16 (1.06-1.28)**	**1.19 (1.08-1.30)**	**1.25 (1.14-1.36)**
	IR[‡]	1.42 (1.28-1.56)	1.44 (1.31-1.58)	1.44 (1.31-1.58)	1.47 (1.34-1.61)	1.55 (1.41-1.69)
Heart failure	Events/At risk	46/4,566	48/4,906	61/4,767	60/4,872	64/4,905
	**CumI[†]**	**1.01 (0.75-1.33)**	**0.98 (0.73-1.29)**	**1.28 (0.99-1.63)**	**1.23 (0.95-1.57)**	**1.30 (1.02-1.65)**
	IR[‡]	1.33 (0.98-1.78)	1.28 (0.95-1.70)	1.69 (1.29-2.17)	1.62 (1.24-2.09)	1.72 (1.33-2.20)
Ischemic heart diseases	Events/At risk	42/4,504	60/4,544	56/4,469	57/4,714	60/4,831
	**CumI[†]**	**0.93 (0.68-1.25)**	**1.32 (1.02-1.69)**	**1.25 (0.96-1.61)**	**1.21 (0.93-1.55)**	**1.24 (0.96-1.59)**
	IR[‡]	1.02 (0.74-1.38)	1.46 (1.12-1.88)	1.38 (1.04-1.79)	1.32 (1.00-1.72)	1.35 (1.03-1.74)

© 2023 Chen Q et al. *JAMA Network Open.*

Respiratory diseases	Events/At risk	33/3,880	40/4,863	38/4,641	44/5,038	60/5,243
	**CumI**[†]	**0.85 (0.60-1.18)**	**0.82 (0.60-1.11)**	**0.82 (0.59-1.11)**	**0.87 (0.64-1.16)**	**1.14 (0.88-1.46)**
	IR[‡]	1.17 (0.81-1.64)	1.13 (0.81-1.54)	1.12 (0.79-1.54)	1.20 (0.87-1.62)	1.58 (1.20-2.03)
Injury/poisoning	Events/At risk	33/2,972	25/3,120	41/3,138	34/3,208	44/3,464
	**CumI**[†]	**1.11 (0.78-1.54)**	**0.80 (0.53-1.17)**	**1.31 (0.95-1.75)**	**1.06 (0.75-1.46)**	**1.27 (0.94-1.69)**
	IR[‡]	1.45 (1.00-2.04)	1.04 (0.67-1.54)	1.70 (1.22-2.31)	1.37 (0.95-1.91)	1.65 (1.20-2.22)
Symptoms/signs/abnormal laboratory findings[**]	Events/At risk	32/2,922	38/2,997	24/2,761	22/2,655	31/2,483
	**CumI**[†]	**1.10 (0.77-1.53)**	**1.27 (0.91-1.72)**	**0.87 (0.57-1.27)**	**0.83 (0.54-1.23)**	**1.25 (0.87-1.75)**
	IR[‡]	1.30 (0.89-1.83)	1.47 (1.04-2.02)	1.00 (0.64-1.48)	0.93 (0.58-1.41)	1.40 (0.95-1.99)
Neoplasms	Events/At risk	40/2,748	41/2,793	30/2,722	43/2,869	33/2,929
	**CumI**[†]	**1.46 (1.06-1.96)**	**1.47 (1.07-1.97)**	**1.10 (0.76-1.55)**	**1.50 (1.10-2.00)**	**1.13 (0.79-1.56)**
	IR[‡]	2.20 (1.57-3.00)	2.20 (1.58-2.98)	1.68 (1.13-2.39)	2.27 (1.64-3.06)	1.70 (1.17-2.38)
Digestive diseases	Events/At risk	43/2,123	47/2,138	51/1,996	51/2,200	48/2,093
	**CumI**[†]	**2.03 (1.49-2.69)**	**2.20 (1.64-2.89)**	**2.56 (1.93-3.32)**	**2.32 (1.75-3.01)**	**2.29 (1.72-3.00)**
	IR[‡]	2.52 (1.82-3.40)	2.74 (2.01-3.64)	3.20 (2.38-4.21)	2.86 (2.13-3.76)	2.87 (2.12-3.81)
Other heart valve disorders[§§]	Events/At risk	18/1,477	16/1,498	14/1,630	17/1,623	12/1,534
	**CumI**[†]	**1.22 (0.75-1.89)**	**1.07 (0.64-1.70)**	**0.86 (0.49-1.41)**	**1.05 (0.64-1.64)**	**0.78 (0.43-1.33)**
	IR[‡]	1.31 (0.78-2.07)	1.15 (0.66-1.87)	0.92 (0.50-1.55)	1.13 (0.66-1.80)	0.83 (0.43-1.45)
Genitourinary diseases	Events/At risk	12/1,468	21/1,531	24/1,570	13/1,672	23/1,707
	**CumI**[†]	**0.82 (0.45-1.39)**	**1.37 (0.88-2.06)**	**1.53 (1.01-2.23)**	**0.78 (0.44-1.30)**	**1.35 (0.88-1.99)**
	IR[‡]	1.01 (0.52-1.76)	1.74 (1.08-2.66)	1.92 (1.23-2.85)	0.97 (0.52-1.66)	1.71 (1.09-2.57)
Infectious/parasitic diseases	Events/At risk	12/1,059	10/1,069	12/1,037	11/1,204	15/1,189
	**CumI**[†]	**1.13 (0.62-1.92)**	**0.94 (0.48-1.67)**	**1.16 (0.64-1.96)**	**0.91 (0.49-1.59)**	**1.26 (0.74-2.03)**
	IR[‡]	1.63 (0.84-2.84)	1.35 (0.65-2.47)	1.56 (0.81-2.72)	1.24 (0.62-2.22)	1.72 (0.96-2.83)
Endocrine/nutritional/metabolic diseases	Events/At risk	<10/680[‖]	11/670	<10/616[‖]	<10/634[‖]	11/721
	**CumI**[†]	**1.03 (0.46-2.03)**	**1.64 (0.87-2.84)**	**0.97 (0.41-2.03)**	**1.26 (0.60-2.39)**	**1.53 (0.81-2.64)**
	IR[‡]	1.27 (0.51-2.61)	2.14 (1.07-3.82)	1.29 (0.47-2.80)	1.60 (0.69-3.16)	1.99 (0.99-3.55)
Diseases of blood/blood-forming organs	Events/At risk	24/492	22/503	19/465	24/514	22/483
	**CumI**[†]	**4.88 (3.21-7.04)**	**4.37 (2.83-6.42)**	**4.09 (2.55-6.17)**	**4.67 (3.08-6.74)**	**4.55 (2.94-6.68)**
	IR[‡]	6.34 (4.06-9.44)	5.82 (3.65-8.81)	5.33 (3.21-8.33)	6.06 (3.89-9.02)	6.01 (3.76-9.09)
Neurological disorders[##]	Events/At risk	<10/491[‖]	<10/455[‖]	<10/402[‖]	<10/441[‖]	<10/405[‖]
	**CumI**[†]	**0.61 (0.17-1.68)**	**0.22 (0.02-1.18)**	**24.88 (20.76-29.20)**	**0.23 (0.02-1.21)**	**0.49 (0.10-1.67)**
	IR[‡]	0.72 (0.15-2.10)	0.28 (0.01-1.54)	0.00 (0.00-1.11)	0.27 (0.01-1.51)	0.61 (0.07-2.22)

† Cumulative incidence (%, 95% confidence interval), estimated by the cumulative incidence competing risk method

‡ Incidence rate (per 100-person-year, 95% confidence interval)

* Defined as ≥2 (outpatient) prescription records of antiplatelet agents within six months (*i.e.*, 183 days) before the index dates (exclusive)

§ Identified within the index hospitalizations unless otherwise stated

© 2023 Chen Q et al. *JAMA Network Open.*

|| Except for rheumatic mitral stenosis and mechanical heart valves
Identified within the prior four years (except for venous thromboembolism which was identified within the prior six months)
** Except for abnormalities of heart beat (ICD-10 code: R00) and cardiac murmurs and other cardiac sounds (ICD-10 code: R01)
§§ Except for endocarditis, valve unspecified, in diseases classified elsewhere (ICD-10 code: I398)
Except for transient cerebral ischaemic attacks and related syndromes (ICD-10 code: G45) and vascular syndromes of brain in cerebrovascular diseases (ICD-10 code: G46)
||| Exact result was masked to prevent potential individual or group disclosure.
Abbreviations: CumI, cumulative incidence; IR, incidence rate.

© 2023 Chen Q et al. *JAMA Network Open.*

eTable 24. Subgroup Analysis of Risk of All-Cause Mortality Within the 1-Year Follow-up After Incident Nonvalvular Atrial Fibrillation Diagnosis

Subgroup	Cohort	2014	2015	2016	2017	2018
Sex						
Male	Events/At risk	5,605/31,391	5,746/34,046	5,627/33,761	5,706/35,304	5,756/35,246
	**CumI**†	**17.86 (17.43-18.28)**	**16.88 (16.48-17.27)**	**16.67 (16.27-17.06)**	**16.16 (15.78-16.55)**	**16.33 (15.94-16.72)**
	IR‡	20.43 (19.90-20.97)	19.19 (18.70-19.69)	18.93 (18.44-19.44)	18.27 (17.80-18.75)	18.54 (18.07-19.03)
Female	Events/At risk	5,084/24,489	5,448/27,271	5,048/26,257	5,356/27,086	5,144/26,450
	**CumI**†	**20.76 (20.25-21.27)**	**19.98 (19.50-20.45)**	**19.23 (18.75-19.70)**	**19.77 (19.30-20.25)**	**19.45 (18.97-19.92)**
	IR‡	24.42 (23.75-25.10)	23.37 (22.75-24.00)	22.32 (21.71-22.94)	23.08 (22.46-23.70)	22.70 (22.08-23.32)
Immigration background						
Native Dutch	Events/At risk	9,447/49,197	9,937/53,983	9,405/52,449	9,725/54,543	9,641/53,914
	**CumI**†	**19.20 (18.85-19.55)**	**18.41 (18.08-18.73)**	**17.93 (17.60-18.26)**	**17.83 (17.51-18.15)**	**17.88 (17.56-18.21)**
	IR‡	22.26 (21.81-22.71)	21.22 (20.81-21.64)	20.60 (20.18-21.02)	20.45 (20.05-20.86)	20.59 (20.18-21.01)
First/Second-generation immigrants	Events/At risk	1,242/6,683	1,257/7,334	1,270/7,569	1,337/7,847	1,259/7,782
	**CumI**†	**18.58 (17.65-19.51)**	**17.14 (16.27-18.00)**	**16.78 (15.93-17.62)**	**17.04 (16.20-17.87)**	**16.18 (15.36-16.99)**
	IR‡	21.36 (20.19-22.58)	19.55 (18.48-20.66)	19.04 (18.00-20.11)	19.40 (18.38-20.47)	18.27 (17.28-19.31)
CHA_2DS_2-VASc score						
0	Events/At risk	163/4,038	183/4,787	180/4,790	145/4,760	149/4,589
	**CumI**†	**4.04 (3.43-4.64)**	**3.82 (3.28-4.36)**	**3.76 (3.22-4.29)**	**3.05 (2.56-3.53)**	**3.25 (2.73-3.76)**
	IR‡	4.15 (3.54-4.84)	3.93 (3.39-4.55)	3.86 (3.32-4.47)	3.11 (2.63-3.66)	3.33 (2.82-3.91)
1	Events/At risk	567/7,422	564/8,309	566/8,160	576/8,577	591/8,565
	**CumI**†	**7.64 (7.03-8.24)**	**6.79 (6.25-7.33)**	**6.94 (6.38-7.49)**	**6.72 (6.18-7.24)**	**6.90 (6.36-7.44)**
	IR‡	8.05 (7.40-8.74)	7.13 (6.55-7.74)	7.29 (6.70-7.92)	7.04 (6.48-7.64)	7.26 (6.69-7.87)
≥2	Events/At risk	9,959/44,420	10,447/48,221	9,929/47,068	1,0341/49,053	10,160/48,542
	**CumI**†	**22.42 (22.03-22.81)**	**21.66 (21.30-22.03)**	**21.10 (20.73-21.46)**	**21.08 (20.72-21.44)**	**20.93 (20.57-21.29)**
	IR‡	26.71 (26.19-27.24)	25.67 (25.18-26.17)	24.87 (24.39-25.37)	24.85 (24.38-25.34)	24.72 (24.24-25.21)
HAS-BLED score						
0	Events/At risk	202/5,034	250/6,083	225/6,227	219/6,506	229/6,531
	**CumI**†	**4.01 (3.47-4.55)**	**4.11 (3.61-4.61)**	**3.61 (3.15-4.08)**	**3.37 (2.93-3.80)**	**3.51 (3.06-3.95)**
	IR‡	4.13 (3.58-4.74)	4.24 (3.73-4.80)	3.71 (3.24-4.23)	3.45 (3.01-3.94)	3.60 (3.15-4.10)
1	Events/At risk	2,789/17,923	3,010/20,476	2,900/20,433	3,011/21,426	3,024/21,440
	**CumI**†	**15.56 (15.03-16.09)**	**14.70 (14.21-15.18)**	**14.19 (13.71-14.67)**	**14.05 (13.59-14.52)**	**14.10 (13.64-14.57)**
	IR‡	17.45 (16.81-18.11)	16.43 (15.85-17.03)	15.79 (15.22-16.38)	15.61 (15.05-16.17)	15.72 (15.17-16.29)
2	Events/At risk	4,039/20,011	4,196/21,706	4,006/20,811	4,125/21,490	4,137/21,309
	**CumI**†	**20.18 (19.63-20.74)**	**19.33 (18.80-19.85)**	**19.25 (18.71-19.78)**	**19.19 (18.67-19.72)**	**19.41 (18.88-19.94)**
	IR‡	23.59 (22.86-24.32)	22.42 (21.75-23.11)	22.30 (21.62-23.00)	22.23 (21.56-22.92)	22.63 (21.94-23.33)
≥3	Events/At risk	3,659/12,912	3,738/13,052	3,544/12,547	3,707/12,968	3,510/12,416

© 2023 Chen Q et al. *JAMA Network Open.*

	**CumI†**	**28.34 (27.56-29.11)**	**28.64 (27.86-29.41)**	**28.25 (27.45-29.03)**	**28.59 (27.80-29.36)**	**28.27 (27.47-29.06)**
	IR‡	35.69 (34.54-36.86)	36.20 (35.05-37.38)	35.62 (34.45-36.81)	36.18 (35.03-37.37)	35.71 (34.54-36.92)
Use of antithrombotic agents						
Pre-existing chronic use of antiplatelet agents*	Events/At risk	3,280/16,060	3,346/16,304	3,293/15,790	3,358/16,683	3,244/16,264
	**CumI†**	**20.42 (19.80-21.04)**	**20.52 (19.90-21.14)**	**20.85 (20.22-21.49)**	**20.13 (19.52-20.73)**	**19.95 (19.33-20.56)**
	IR‡	23.90 (23.08-24.73)	24.05 (23.25-24.88)	24.50 (23.67-25.35)	23.52 (22.73-24.33)	23.35 (22.55-24.16)
≥1 oral anticoagulant prescription within the 1-year follow-up	Events/At risk	3,976/37,905	4,334/44,426	4,219/43,767	4,542/45,144	4,442/44,881
	**CumI†**	**10.49 (10.18-10.80)**	**9.76 (9.48-10.03)**	**9.64 (9.36-9.92)**	**10.06 (9.78-10.34)**	**9.90 (9.62-10.17)**
	IR‡	11.13 (10.78-11.48)	10.31 (10.00-10.62)	10.18 (9.88-10.49)	10.65 (10.34-10.96)	10.49 (10.19-10.81)
Comorbidities (or medical history)§						
Asthma	Events/At risk	107/687	136/837	130/791	140/911	195/1,046
	**CumI†**	**15.57 (12.82-18.24)**	**16.25 (13.71-18.71)**	**16.43 (13.81-18.98)**	**15.37 (12.99-17.68)**	**18.64 (16.25-20.97)**
	IR‡	17.52 (14.36-21.17)	18.41 (15.44-21.77)	18.52 (15.48-22.00)	17.40 (14.64-20.53)	21.44 (18.53-24.67)
Chronic obstructive pulmonary disease	Events/At risk	1,865/5,837	1,993/6,073	1,791/5,713	1,837/5,718	1,755/5,684
	**CumI†**	**31.95 (30.74-33.14)**	**32.82 (31.63-33.99)**	**31.35 (30.14-32.54)**	**32.13 (30.91-33.33)**	**30.88 (29.66-32.07)**
	IR‡	41.54 (39.67-43.46)	43.06 (41.19-45.00)	40.68 (38.82-42.61)	42.11 (40.20-44.08)	40.16 (38.31-42.09)
Other chronic lung disease	Events/At risk	332/748	379/835	369/811	432/973	520/1,116
	**CumI†**	**44.39 (40.71-47.83)**	**45.39 (41.91-48.66)**	**45.50 (41.96-48.82)**	**44.40 (41.19-47.43)**	**46.59 (43.59-49.44)**
	IR‡	68.95 (61.73-76.78)	72.63 (65.50-80.33)	72.28 (65.09-80.04)	70.84 (64.32-77.84)	75.14 (68.82-81.88)
Heart failure	Events/At risk	3,519/9,984	3,626/10,705	3,563/10,407	3,616/10,882	3,683/11,043
	**CumI†**	**35.25 (34.30-36.18)**	**33.87 (32.97-34.76)**	**34.24 (33.32-35.14)**	**33.23 (32.34-34.11)**	**33.35 (32.47-34.23)**
	IR‡	47.58 (46.02-49.18)	45.23 (43.77-46.73)	45.83 (44.34-47.36)	44.31 (42.88-45.78)	44.57 (43.15-46.04)
Myocardial infarction (history)	Events/At risk	556/2,552	630/2,899	616/2,997	620/3,190	593/3,216
	**CumI†**	**21.79 (20.17-23.37)**	**21.73 (20.22-23.22)**	**20.55 (19.09-21.99)**	**19.44 (18.05-20.80)**	**18.44 (17.09-19.77)**
	IR‡	26.14 (24.02-28.41)	26.02 (24.03-28.14)	24.43 (22.54-26.44)	22.91 (21.14-24.78)	21.58 (19.88-23.39)
Hypertension	Events/At risk	3,298/16,265	3,498/17,925	3,187/17,379	3,610/18,579	3,672/19,113
	**CumI†**	**20.28 (19.66-20.89)**	**19.51 (18.93-20.09)**	**18.34 (17.76-18.91)**	**19.43 (18.86-20.00)**	**19.21 (18.65-19.77)**
	IR‡	23.68 (22.88-24.50)	22.70 (21.95-23.46)	21.12 (20.40-21.87)	22.60 (21.87-23.35)	22.36 (21.64-23.09)
Other valvular heart disease‖	Events/At risk	1,581/6,639	1,747/7,057	1,602/6,653	1,666/6,875	1,600/6,721
	**CumI†**	**23.81 (22.78-24.83)**	**24.76 (23.74-25.76)**	**24.08 (23.04-25.10)**	**24.23 (23.21-25.24)**	**23.81 (22.78-24.82)**
	IR‡	28.72 (27.32-30.17)	30.17 (28.78-31.62)	29.10 (27.70-30.56)	29.34 (27.95-30.78)	28.86 (27.47-30.31)
Peripheral artery disease	Events/At risk	286/1,025	404/1,212	294/994	305/1,023	301/986
	**CumI†**	**27.90 (25.10-30.60)**	**33.33 (30.63-35.94)**	**29.58 (26.68-32.36)**	**29.81 (26.95-32.56)**	**30.53 (27.59-33.34)**
	IR‡	34.78 (30.87-39.06)	44.44 (40.21-48.99)	37.94 (33.73-42.54)	38.61 (34.40-43.20)	40.21 (35.79-45.01)
Liver diseases	Events/At risk	394/1,052	415/1,058	390/1,037	419/1,051	450/1,036
	**CumI†**	**37.45 (34.46-40.31)**	**39.22 (36.21-42.10)**	**37.61 (34.59-40.49)**	**39.87 (36.83-42.76)**	**43.44 (40.34-46.38)**
	IR‡	53.63 (48.46-59.20)	56.75 (51.42-62.48)	54.49 (49.21-60.17)	58.61 (53.13-64.50)	67.11 (61.05-73.60)

© 2023 Chen Q et al. *JAMA Network Open.*

Gastroesophageal reflux disease	Events/At risk	24/117	24/92	31/114	27/127	37/111
	**CumI**†	**20.51 (12.85-27.50)**	**26.09 (16.55-34.54)**	**27.19 (18.55-34.92)**	**21.26 (13.81-28.06)**	**33.33 (23.96-41.55)**
	IR‡	23.91 (15.32-35.57)	31.96 (20.48-47.56)	33.69 (22.89-47.83)	25.21 (16.61-36.67)	43.47 (30.61-59.92)
Peptic ulcer disease	Events/At risk	78/239	80/244	75/212	70/226	73/235
	**CumI**†	**32.64 (26.42-38.33)**	**32.79 (26.63-38.43)**	**35.38 (28.61-41.50)**	**30.97 (24.67-36.75)**	**31.06 (24.89-36.73)**
	IR‡	44.15 (34.90-55.11)	43.89 (34.80-54.62)	50.36 (39.61-63.13)	40.97 (31.94-51.77)	40.60 (31.82-51.04)
Chronic kidney diseases	Events/At risk	2,244/5,808	2,388/6,304	2,402/6,516	2,500/6,896	2,453/6,585
	**CumI**†	**38.64 (37.37-39.88)**	**37.88 (36.67-39.07)**	**36.86 (35.68-38.02)**	**36.25 (35.11-37.38)**	**37.25 (36.07-38.41)**
	IR‡	54.32 (52.09-56.61)	53.10 (50.99-55.27)	50.76 (48.75-52.83)	49.81 (47.88-51.80)	51.85 (49.82-53.94)
Anaemia	Events/At risk	1,235/3,527	1,402/3,868	1,450/4,015	1,585/4,390	1,642/4,546
	**CumI**†	**35.02 (33.42-36.57)**	**36.25 (34.71-37.74)**	**36.11 (34.61-37.58)**	**36.10 (34.67-37.51)**	**36.12 (34.71-37.50)**
	IR‡	46.55 (43.99-49.22)	48.79 (46.27-51.41)	48.62 (46.15-51.19)	48.80 (46.43-51.27)	49.06 (46.71-51.49)
Coagulopathy	Events/At risk	279/749	378/1,036	377/982	420/1,044	473/1,179
	**CumI**†	**37.25 (33.69-40.62)**	**36.49 (33.49-39.35)**	**38.39 (35.27-41.36)**	**40.23 (37.18-43.13)**	**40.12 (37.25-42.85)**
	IR‡	52.11 (46.18-58.60)	50.20 (45.26-55.52)	54.91 (49.51-60.74)	58.92 (53.42-64.84)	58.52 (53.36-64.04)
Diabetes	Events/At risk	2,367/9,242	2,550/10,024	2,459/9,944	2,610/10,308	2,671/10,341
	**CumI**†	**25.61 (24.72-26.50)**	**25.44 (24.58-26.29)**	**24.73 (23.88-25.57)**	**25.32 (24.48-26.15)**	**25.83 (24.98-26.67)**
	IR‡	31.40 (30.15-32.70)	31.10 (29.90-32.33)	30.20 (29.01-31.41)	31.01 (29.83-32.22)	31.90 (30.70-33.13)
Thyroid disease	Events/At risk	300/1,408	331/1,504	260/1,409	314/1,580	349/1,570
	**CumI**†	**21.31 (19.14-23.42)**	**22.01 (19.89-24.07)**	**18.45 (16.40-20.45)**	**19.87 (17.88-21.82)**	**22.23 (20.15-24.26)**
	IR‡	25.20 (22.43-28.22)	26.10 (23.36-29.07)	21.39 (18.87-24.16)	23.17 (20.67-25.87)	26.66 (23.94-29.61)
Ischemic stroke (history)#	Events/At risk	466/1,613	470/1,759	474/1,687	486/1,792	436/1,685
	**CumI**†	**28.89 (26.64-31.07)**	**26.72 (24.62-28.76)**	**28.10 (25.92-30.21)**	**27.12 (25.03-29.15)**	**25.88 (23.75-27.94)**
	IR‡	36.42 (33.19-39.88)	33.10 (30.17-36.23)	35.15 (32.05-38.46)	33.65 (30.72-36.77)	31.68 (28.78-34.80)
Transient ischemic attack#	Events/At risk	167/793	150/832	159/804	136/860	148/778
	**CumI**†	**21.06 (18.17-23.85)**	**18.03 (15.37-20.60)**	**19.78 (16.98-22.48)**	**15.81 (13.34-18.22)**	**19.02 (16.22-21.73)**
	IR‡	24.78 (21.16-28.83)	20.57 (17.41-24.14)	23.08 (19.63-26.96)	17.76 (14.90-21.01)	21.98 (18.58-25.82)
Other arterial thromboembolism#	Events/At risk	177/593	155/541	130/509	140/474	121/398
	**CumI**†	**29.85 (26.07-33.44)**	**28.65 (24.74-32.36)**	**25.54 (21.65-29.23)**	**29.54 (25.31-33.53)**	**30.40 (25.73-34.78)**
	IR‡	37.65 (32.31-43.63)	35.84 (30.42-41.95)	31.17 (26.04-37.01)	37.64 (31.66-44.41)	38.78 (32.18-46.34)
Parkinson's disease	Events/At risk	149/429	159/514	150/496	175/557	163/528
	**CumI**†	**34.73 (30.07-39.09)**	**30.93 (26.82-34.82)**	**30.24 (26.08-34.17)**	**31.42 (27.45-35.17)**	**30.87 (26.82-34.70)**
	IR‡	47.06 (39.81-55.26)	40.14 (34.14-46.89)	38.89 (32.91-45.63)	41.02 (35.17-47.57)	40.10 (34.18-46.75)
Alzheimer's disease	Events/At risk	226/506	275/573	274/559	258/575	277/616
	**CumI**†	**44.66 (40.16-48.83)**	**47.99 (43.74-51.93)**	**49.02 (44.70-53.00)**	**44.87 (40.65-48.79)**	**44.97 (40.90-48.76)**
	IR‡	65.40 (57.15-74.50)	74.25 (65.74-83.57)	76.85 (68.02-86.50)	68.31 (60.23-77.18)	67.32 (59.62-75.73)
Autoimmune disease	Events/At risk	31/106	31/105	29/117	47/154	39/139

© 2023 Chen Q et al. *JAMA Network Open.*

	**CumI†**	**29.25 (20.03-37.40)**	**29.52 (20.24-37.73)**	**24.79 (16.54-32.22)**	**30.52 (22.85-37.42)**	**28.06 (20.19-35.15)**
	IR‡	38.21 (25.96-54.23)	39.16 (26.61-55.58)	30.21 (20.23-43.39)	38.97 (28.63-51.82)	35.67 (25.37-48.76)
Systemic connective tissue disorders	Events/At risk	138/466	181/601	133/538	152/528	199/640
	**CumI†**	**29.61 (25.34-33.64)**	**30.12 (26.35-33.69)**	**24.72 (20.99-28.28)**	**28.79 (24.82-32.55)**	**31.09 (27.41-34.59)**
	IR‡	37.55 (31.54-44.36)	39.16 (33.67-45.30)	29.91 (25.04-35.44)	37.06 (31.40-43.44)	40.27 (34.87-46.27)
Venous thromboembolism#	Events/At risk	296/839	263/833	300/854	357/998	313/918
	**CumI†**	**35.28 (31.96-38.43)**	**31.57 (28.34-34.66)**	**35.13 (31.85-38.25)**	**35.77 (32.73-38.68)**	**34.10 (30.96-37.09)**
	IR‡	48.71 (43.32-54.59)	41.66 (36.77-47.01)	47.92 (42.65-53.67)	48.79 (43.86-54.13)	46.39 (41.39-51.82)
Major bleeding#	Events/At risk	698/2,287	737/2,454	762/2,444	791/2,617	772/2,545
	**CumI†**	**30.52 (28.61-32.38)**	**30.03 (28.20-31.82)**	**31.18 (29.32-32.99)**	**30.23 (28.44-31.96)**	**30.33 (28.52-32.10)**
	IR‡	38.87 (36.04-41.86)	38.12 (35.42-40.98)	40.33 (37.52-43.30)	38.22 (35.60-40.97)	38.93 (36.23-41.78)
Malignant tumour	Events/At risk	2,203/4,364	2,273/4,664	2,325/4,682	2,377/4,830	2,478/4,979
	**CumI†**	**50.48 (48.98-51.94)**	**48.73 (47.28-50.15)**	**49.66 (48.21-51.07)**	**49.21 (47.78-50.60)**	**49.77 (48.36-51.14)**
	IR‡	80.39 (77.06-83.82)	75.97 (72.88-79.16)	78.52 (75.36-81.78)	77.23 (74.15-80.40)	79.29 (76.20-82.48)
≥1 hospitalization within the prior 4 years	Events/At risk	7,911/37,004	7,988/39,243	7,549/38,189	7,819/39,582	7,612/38,138
	**CumI†**	**21.38 (20.96-21.80)**	**20.36 (19.96-20.75)**	**19.77 (19.37-20.17)**	**19.75 (19.36-20.15)**	**19.96 (19.56-20.36)**
	IR‡	25.19 (24.64-25.75)	23.83 (23.31-24.35)	23.01 (22.49-23.53)	23.00 (22.49-23.52)	23.31 (22.79-23.84)
Without any hospitalization within the prior 4 years	Events/At risk	2,778/18,876	3,206/22,074	3,126/21,829	3,243/22,808	3,288/23,558
	**CumI†**	**14.72 (14.21-15.22)**	**14.52 (14.06-14.99)**	**14.32 (13.85-14.78)**	**14.22 (13.76-14.67)**	**13.96 (13.51-14.40)**
	IR‡	16.49 (15.88-17.11)	16.25 (15.69-16.82)	16.01 (15.45-16.58)	15.86 (15.32-16.41)	15.61 (15.08-16.16)
Survived until discharge from the index hospitalization	Events/At risk	8,277/53,468	8,572/58,695	8,118/57,461	8,353/59,681	8,035/58,831
	**CumI†**	**15.48 (15.17-15.79)**	**14.60 (14.32-14.89)**	**14.13 (13.84-14.41)**	**14.00 (13.72-14.27)**	**13.66 (13.38-13.93)**
	IR‡	17.18 (16.81-17.55)	16.12 (15.78-16.46)	15.53 (15.20-15.87)	15.36 (15.04-15.70)	14.98 (14.66-15.31)
Main reason for admission to the index hospitalization						
Atrial fibrillation	Events/At risk	1,163/19,496	1,250/23,257	1,206/23,047	1,257/23,782	1,107/22,877
	**CumI†**	**5.97 (5.63-6.30)**	**5.37 (5.08-5.66)**	**5.23 (4.94-5.52)**	**5.29 (5.00-5.57)**	**4.84 (4.56-5.12)**
	IR‡	6.19 (5.84-6.56)	5.56 (5.26-5.88)	5.40 (5.10-5.72)	5.46 (5.16-5.77)	4.99 (4.70-5.29)
Not primarily for atrial fibrillation	Events/At risk	9,526/36,384	9,944/38,060	9,469/36,971	9,805/38,608	9,793/38,819
	**CumI†**	**26.18 (25.73-26.63)**	**26.13 (25.68-26.57)**	**25.61 (25.17-26.06)**	**25.40 (24.96-25.83)**	**25.23 (24.79-25.66)**
	IR‡	32.33 (31.68-32.98)	32.31 (31.68-32.95)	31.55 (30.91-32.19)	31.22 (30.61-31.85)	31.08 (30.46-31.70)
Heart failure	Events/At risk	1,543/4,566	1,604/4,906	1,587/4,767	1,585/4,872	1,593/4,905
	**CumI†**	**33.79 (32.41-35.15)**	**32.69 (31.37-33.99)**	**33.29 (31.94-34.62)**	**32.53 (31.20-33.84)**	**32.48 (31.15-33.77)**
	IR‡	44.46 (42.27-46.74)	42.64 (40.58-44.78)	43.59 (41.47-45.79)	42.53 (40.46-44.68)	42.62 (40.56-44.77)
Ischemic heart diseases	Events/At risk	528/4,504	580/4,544	511/4,469	516/4,714	475/4,831
	**CumI†**	**11.72 (10.78-12.66)**	**12.76 (11.79-13.73)**	**11.43 (10.50-12.36)**	**10.95 (10.05-11.83)**	**9.83 (8.99-10.67)**
	IR‡	12.79 (11.73-13.93)	14.05 (12.93-15.24)	12.47 (11.41-13.60)	11.91 (10.90-12.98)	10.59 (9.66-11.59)

© 2023 Chen Q et al. *JAMA Network Open.*

Respiratory diseases	Events/At risk	1,418/3,880	1,738/4,863	1,618/4,641	1,795/5,038	1,827/5,244
	**CumI†**	**36.55 (35.01-38.04)**	**35.74 (34.38-37.07)**	**34.86 (33.48-36.22)**	**35.63 (34.29-36.94)**	**34.84 (33.54-36.12)**
	IR‡	50.14 (47.57-52.82)	48.88 (46.61-51.23)	47.49 (45.21-49.86)	48.85 (46.61-51.16)	47.72 (45.56-49.96)
Injury/poisoning	Events/At risk	929/2,972	949/3,120	950/3,138	960/3,209	1,035/3,464
	**CumI†**	**31.26 (29.57-32.90)**	**30.42 (28.78-32.01)**	**30.27 (28.65-31.86)**	**29.92 (28.31-31.48)**	**29.88 (28.34-31.39)**
	IR‡	40.62 (38.05-43.32)	39.36 (36.90-41.95)	39.08 (36.63-41.64)	38.27 (35.89-40.77)	38.57 (36.26-40.99)
Symptoms/signs/abnormal laboratory findings**	Events/At risk	644/2,922	585/2,997	524/2,761	447/2,655	411/2,483
	**CumI†**	**22.04 (20.52-23.53)**	**19.52 (18.09-20.93)**	**18.98 (17.50-20.43)**	**16.84 (15.40-18.25)**	**16.55 (15.08-18.00)**
	IR‡	25.95 (23.98-28.03)	22.50 (20.71-24.40)	21.68 (19.86-23.61)	18.86 (17.15-20.69)	18.54 (16.79-20.42)
Neoplasms	Events/At risk	1,231/2,749	1,233/2,794	1,247/2,722	1,268/2,869	1,291/2,929
	**CumI†**	**44.78 (42.89-46.61)**	**44.13 (42.26-45.94)**	**45.81 (43.91-47.65)**	**44.20 (42.35-45.98)**	**44.08 (42.25-45.85)**
	IR‡	67.03 (63.34-70.89)	65.31 (61.72-69.06)	69.11 (65.32-73.05)	66.20 (62.60-69.94)	65.83 (62.29-69.52)
Digestive diseases	Events/At risk	608/2,477	594/2,470	563/2,297	596/2,515	602/2,396
	**CumI†**	**24.55 (22.83-26.22)**	**24.05 (22.34-25.72)**	**24.51 (22.73-26.25)**	**23.70 (22.02-25.34)**	**25.13 (23.37-26.84)**
	IR‡	29.99 (27.65-32.47)	29.48 (27.16-31.95)	30.28 (27.83-32.89)	28.77 (26.51-31.18)	31.08 (28.65-33.66)
Other heart valve disorders§§	Events/At risk	138/1,477	140/1,498	147/1,630	156/1,623	118/1,534
	**CumI†**	**9.34 (7.85-10.82)**	**9.35 (7.86-10.81)**	**9.02 (7.62-10.40)**	**9.61 (8.17-11.03)**	**7.69 (6.35-9.02)**
	IR‡	9.99 (8.40-11.81)	10.03 (8.44-11.84)	9.64 (8.14-11.33)	10.26 (8.72-12.01)	8.11 (6.71-9.71)
Genitourinary diseases	Events/At risk	387/1,469	441/1,531	434/1,570	468/1,672	488/1,707
	**CumI†**	**26.34 (24.06-28.56)**	**28.80 (26.50-31.04)**	**27.64 (25.40-29.82)**	**27.99 (25.81-30.11)**	**28.59 (26.41-30.70)**
	IR‡	32.32 (29.18-35.71)	36.39 (33.07-39.95)	34.37 (31.21-37.76)	34.71 (31.63-38.00)	36.00 (32.87-39.34)
Infectious/parasitic diseases	Events/At risk	396/1,059	405/1,069	328/1,037	392/1,204	383/1,189
	**CumI†**	**37.39 (34.41-40.24)**	**37.89 (34.91-40.73)**	**31.63 (28.74-34.40)**	**32.56 (29.86-35.15)**	**32.21 (29.50-34.82)**
	IR‡	53.31 (48.19-58.83)	54.39 (49.23-59.96)	42.40 (37.93-47.24)	44.03 (39.78-48.61)	43.54 (39.29-48.12)
Endocrine/nutritional/metabolic diseases	Events/At risk	191/680	211/670	195/616	175/634	215/721
	**CumI†**	**28.09 (24.63-31.39)**	**31.49 (27.88-34.92)**	**31.66 (27.88-35.23)**	**27.60 (24.04-31.00)**	**29.82 (26.40-33.08)**
	IR‡	34.45 (29.73-39.69)	40.62 (35.32-46.49)	41.75 (36.09-48.03)	34.86 (29.88-40.42)	38.40 (33.44-43.89)
Diseases of blood/blood-forming organs	Events/At risk	142/492	167/503	149/465	169/514	154/483
	**CumI†**	**28.86 (24.74-32.76)**	**33.20 (28.96-37.19)**	**32.04 (27.67-36.15)**	**32.88 (28.69-36.82)**	**31.88 (27.60-35.92)**
	IR‡	36.24 (30.52-42.71)	42.88 (36.62-49.90)	40.28 (34.07-47.29)	41.78 (35.72-48.57)	40.87 (34.67-47.86)
Neurological disorders##	Events/At risk	103/491	136/455	100/402	98/441	105/405
	**CumI†**	**20.98 (17.29-24.50)**	**29.89 (25.56-33.97)**	**24.88 (20.53-28.98)**	**22.22 (18.24-26.01)**	**25.93 (21.53-30.07)**
	IR‡	24.63 (20.10-29.87)	37.47 (31.44-44.32)	30.13 (24.51-36.64)	26.47 (21.49-32.26)	32.22 (26.35-39.01)

† Cumulative incidence (%, 95% confidence interval), estimated by the Kaplan-Meier estimator

‡ Incidence rate (per 100-person-year, 95% confidence interval)

* Defined as ≥2 (outpatient) prescription records of antiplatelet agents within six months (*i.e.*, 183 days) before the index dates (exclusive)

§ Identified within the index hospitalizations unless otherwise stated

© 2023 Chen Q et al. *JAMA Network Open.*

|| Except for rheumatic mitral stenosis and mechanical heart valves
Identified within the prior four years (except for venous thromboembolism which was identified within the prior six months)
** Except for abnormalities of heart beat (ICD-10 code: R00) and cardiac murmurs and other cardiac sounds (ICD-10 code: R01)
§§ Except for endocarditis, valve unspecified, in diseases classified elsewhere (ICD-10 code: I398)
Except for transient cerebral ischaemic attacks and related syndromes (ICD-10 code: G45) and vascular syndromes of brain in cerebrovascular diseases (ICD-10 code: G46)
Abbreviations: CumI, cumulative incidence; IR, incidence rate.

© 2023 Chen Q et al. *JAMA Network Open.*

eTable 25. Overall Time Trends in Composition of CHA_2DS_2-VASc Score and HAS-BLED Score of Patients With Incident Nonvalvular Atrial Fibrillation in the Netherlands

	2014<br>(N=55,880)	2015<br>(N=61,317)	2016<br>(N=60,018)	2017<br>(N=62,390)	2018<br>(N=61,696)
CHA_2DS_2-VASc score, mean ± SD	2.95 ± 1.67	2.91 ± 1.68	2.89 ± 1.67	2.90 ± 1.67	2.90 ± 1.66
Heart failure, n (%)					
0	44,139 (79.0)	48,813 (79.6)	47,885 (79.8)	49,739 (79.7)	48,947 (79.3)
1	11,741 (21.0)	12,504 (20.4)	12,133 (20.2)	12,651 (20.3)	12,749 (20.7)
Hypertension, n (%)					
0	35,917 (64.3)	39,255 (64.0)	38,312 (63.8)	38,958 (62.4)	37,904 (61.4)
1	19,963 (35.7)	22,062 (36.0)	21,706 (36.2)	23,432 (37.6)	23,792 (38.6)
Age, n (%)					
0	11,013 (19.7)	12,268 (20.0)	12,335 (20.6)	12,695 (20.3)	12,488 (20.2)
1	15,590 (27.9)	17,403 (28.4)	17,326 (28.9)	18,286 (29.3)	18,357 (29.8)
2	29,277 (52.4)	31,646 (51.6)	30,357 (50.6)	31,409 (50.3)	30,851 (50.0)
Diabetes, n (%)					
0	44,768 (80.1)	49,451 (80.6)	48,308 (80.5)	50,149 (80.4)	49,704 (80.6)
1	11,112 (19.9)	11,866 (19.4)	11,710 (19.5)	12,241 (19.6)	11,992 (19.4)
Stroke/TIA/thromboembolism, n (%)					
0	51,412 (92.0)	56,715 (92.5)	55,696 (92.8)	57,969 (92.9)	57,551 (93.3)
2	4,468 (8.0)	4,602 (7.5)	4,322 (7.2)	4,421 (7.1)	4,145 (6.7)
Vascular disease, n (%)					
0	41,443 (74.2)	46,425 (75.7)	45,282 (75.4)	46,522 (74.6)	45,953 (74.5)
1	14,437 (25.8)	14,892 (24.3)	14,736 (24.6)	15,868 (25.4)	15,743 (25.5)
Sex, n (%)					
0	31,391 (56.2)	34,046 (55.5)	33,761 (56.3)	35,304 (56.6)	35,246 (57.1)
1	24,489 (43.8)	27,271 (44.5)	26,257 (43.7)	27,086 (43.4)	26,450 (42.9)
HAS-BLED score, mean ± SD	1.79 ± 1.02	1.73 ± 1.02	1.71 ± 1.02	1.70 ± 1.02	1.69 ± 1.01
Uncontrolled hypertension, n (%)					
0	40,231 (72.0)	44,888 (73.2)	44,308 (73.8)	46,421 (74.4)	46,087 (74.7)
1	15,649 (28.0)	16,429 (26.8)	15,710 (26.2)	15,969 (25.6)	15,609 (25.3)
Abnormal renal function, n (%)					
0	49,007 (87.7)	54,028 (88.1)	52,480 (87.4)	54,450 (87.3)	54,169 (87.8)
1	6,873 (12.3)	7,289 (11.9)	7,538 (12.6)	7,940 (12.7)	7,527 (12.2)
Abnormal liver function, n (%)					
0	54,533 (97.6)	59,999 (97.9)	58,711 (97.8)	61,073 (97.9)	60,411 (97.9)
1	1,347 (2.4)	1,318 (2.1)	1,307 (2.2)	1,317 (2.1)	1,285 (2.1)
Ischemic stroke/TIA (history), n (%)					

© 2023 Chen Q et al. *JAMA Network Open.*

0	53,245 (95.3)	58,757 (95.8)	57,581 (95.9)	59,967 (96.1)	59,332 (96.2)
1	2,635 (4.7)	2,560 (4.2)	2,437 (4.1)	2,423 (3.9)	2,364 (3.8)
Major bleeding, n (%)					
0	54,110 (96.8)	59,570 (97.2)	58,469 (97.4)	60,761 (97.4)	60,097 (97.4)
1	1,770 (3.2)	1,747 (2.8)	1,549 (2.6)	1,629 (2.6)	1,599 (2.6)
Elderly, n (%)					
0	11,013 (19.7)	12,268 (20.0)	12,335 (20.6)	12,695 (20.3)	12,488 (20.2)
1	44,867 (80.3)	49,049 (80.0)	47,683 (79.4)	49,695 (79.7)	49,208 (79.8)
Alcohol abuse, n (%)					
0	54,974 (98.4)	60,416 (98.5)	59,010 (98.3)	61,220 (98.1)	60,568 (98.2)
1	906 (1.6)	901 (1.5)	1,008 (1.7)	1,170 (1.9)	1,128 (1.8)
Antiplatelet agents or NSIADs, n (%)					
0	30,124 (53.9)	34,448 (56.2)	34,457 (57.4)	36,171 (58.0)	36,180 (58.6)
1	25,756 (46.1)	26,869 (43.8)	25,561 (42.6)	26,219 (42.0)	25,516 (41.4)

Abbreviations: TIA, transient ischemic attack; NSAID, nonsteroidal anti-inflammatory drugs.

© 2023 Chen Q et al. *JAMA Network Open.*

eTable 26. Time Trends in Composition of CHA_2DS_2-VASc Score and HAS-BLED Score of Male Patients With Incident Nonvalvular Atrial Fibrillation in the Netherlands

	2014	2015	2016	2017	2018
	(N=31,391)	(N=34,046)	(N=33,761)	(N=35,304)	(N=35,246)
CHA_2DS_2-VASc score, mean ± SD	2.35 ± 1.56	2.31 ± 1.57	2.31 ± 1.57	2.34 ± 1.58	2.35 ± 1.56
Heart failure, n (%)					
0	25,625 (81.6)	27,911 (82.0)	27,649 (81.9)	28,813 (81.6)	28,583 (81.1)
1	5,766 (18.4)	6,135 (18.0)	6,112 (18.1)	6,491 (18.4)	6,663 (18.9)
Hypertension, n (%)					
0	21,183 (67.5)	22,907 (67.3)	22,519 (66.7)	23,023 (65.2)	22,574 (64.0)
1	10,208 (32.5)	11,139 (32.7)	11,242 (33.3)	12,281 (34.8)	12,672 (36.0)
Age, n (%)					
0	7,915 (25.2)	8,622 (25.3)	8,680 (25.7)	8,974 (25.4)	8,775 (24.9)
1	9,896 (31.5)	10,823 (31.8)	10,770 (31.9)	11,293 (32.0)	11,524 (32.7)
2	13,580 (43.3)	14,601 (42.9)	14,311 (42.4)	15,037 (42.6)	14,947 (42.4)
Diabetes, n (%)					
0	25,281 (80.5)	27,570 (81.0)	27,282 (80.8)	28,405 (80.5)	28,444 (80.7)
1	6,110 (19.5)	6,476 (19.0)	6,479 (19.2)	6,899 (19.5)	6,802 (19.3)
Stroke/TIA/thromboembolism, n (%)					
0	28,959 (92.3)	31,560 (92.7)	31,418 (93.1)	32,907 (93.2)	32,974 (93.6)
2	2,432 (7.7)	2,486 (7.3)	2,343 (6.9)	2,397 (6.8)	2,272 (6.4)
Vascular disease, n (%)					
0	21,610 (68.8)	24,101 (70.8)	23,808 (70.5)	24,558 (69.6)	24,426 (69.3)
1	9,781 (31.2)	9,945 (29.2)	9,953 (29.5)	10,746 (30.4)	10,820 (30.7)
Sex, n (%)					
0	31,391 (100.0)	34,046 (100.0)	33,761 (100.0)	35,304 (100.0)	35,246 (100.0)
1	-	-	-	-	-
HAS-BLED score, mean ± SD	1.71 ± 1.05	1.66 ± 1.04	1.65 ± 1.05	1.64 ± 1.05	1.63 ± 1.04
Uncontrolled hypertension, n (%)					
0	23,500 (74.9)	25,858 (76.0)	25,684 (76.1)	27,017 (76.5)	26,948 (76.5)
1	7,891 (25.1)	8,188 (24.0)	8,077 (23.9)	8,287 (23.5)	8,298 (23.5)
Abnormal renal function, n (%)					
0	27,651 (88.1)	30,182 (88.7)	29,658 (87.8)	30,961 (87.7)	31,130 (88.3)
1	3,740 (11.9)	3,864 (11.3)	4,103 (12.2)	4,343 (12.3)	4,116 (11.7)
Abnormal liver function, n (%)					
0	30,633 (97.6)	33,324 (97.9)	32,969 (97.7)	3,4516 (97.8)	34,483 (97.8)
1	758 (2.4)	722 (2.1)	792 (2.3)	788 (2.2)	763 (2.2)
Ischemic stroke/TIA (history), n (%)					

© 2023 Chen Q et al. *JAMA Network Open.*

0	29,968 (95.5)	32,674 (96.0)	32,461 (96.1)	3,4045 (96.4)	33,968 (96.4)
1	1,423 (4.5)	1,372 (4.0)	1,300 (3.9)	1,259 (3.6)	1,278 (3.6)
Major bleeding, n (%)					
0	30,391 (96.8)	33,021 (97.0)	32,858 (97.3)	34,367 (97.3)	34,292 (97.3)
1	1,000 (3.2)	1,025 (3.0)	903 (2.7)	937 (2.7)	954 (2.7)
Elderly, n (%)					
0	7,915 (25.2)	8,622 (25.3)	8,680 (25.7)	8,974 (25.4)	8,775 (24.9)
1	23,476 (74.8)	25,424 (74.7)	25,081 (74.3)	26,330 (74.6)	26,471 (75.1)
Alcohol abuse, n (%)					
0	30,683 (97.7)	33,334 (97.9)	32,967 (97.6)	34,379 (97.4)	34,362 (97.5)
1	708 (2.3)	712 (2.1)	794 (2.4)	925 (2.6)	884 (2.5)
Antiplatelet agents or NSIADs, n (%)					
0	16,703 (53.2)	18,927 (55.6)	19,116 (56.6)	20,128 (57.0)	20,389 (57.8)
1	14,688 (46.8)	15,119 (44.4)	14,645 (43.4)	15,176 (43.0)	14,857 (42.2)

Abbreviations: TIA, transient ischemic attack; NSAID, nonsteroidal anti-inflammatory drugs.

© 2023 Chen Q et al. *JAMA Network Open.*

eTable 27. Time Trends in Composition of CHA_2DS_2-VASc Score and HAS-BLED Score of Female Patients With Incident Nonvalvular Atrial Fibrillation in the Netherlands

	2014<br>(N=24,489)	2015<br>(N=27,271)	2016<br>(N=26,257)	2017<br>(N=27,086)	2018<br>(N=26,450)
CHA_2DS_2-VASc score, mean ± SD	3.72 ± 1.48	3.66 ± 1.49	3.63 ± 1.50	3.64 ± 1.49	3.64 ± 1.49
Heart failure, n (%)					
0	18,514 (75.6)	20,902 (76.6)	20,236 (77.1)	20,926 (77.3)	20,364 (77.0)
1	5,975 (24.4)	6,369 (23.4)	6,021 (22.9)	6,160 (22.7)	6,086 (23.0)
Hypertension, n (%)					
0	14,734 (60.2)	16,348 (59.9)	15,793 (60.1)	15,935 (58.8)	15,330 (58.0)
1	9,755 (39.8)	10,923 (40.1)	10,464 (39.9)	11,151 (41.2)	11,120 (42.0)
Age, n (%)					
0	3,098 (12.7)	3,646 (13.4)	3,655 (13.9)	3,721 (13.7)	3,713 (14.0)
1	5,694 (23.3)	6,580 (24.1)	6,556 (25.0)	6,993 (25.8)	6,833 (25.8)
2	15,697 (64.1)	17,045 (62.5)	16,046 (61.1)	16,372 (60.4)	15,904 (60.1)
Diabetes, n (%)					
0	19,487 (79.6)	21,881 (80.2)	21,026 (80.1)	21,744 (80.3)	21,260 (80.4)
1	5,002 (20.4)	5,390 (19.8)	5,231 (19.9)	5,342 (19.7)	5,190 (19.6)
Stroke/TIA/thromboembolism, n (%)					
0	22,453 (91.7)	25,155 (92.2)	24,278 (92.5)	25,062 (92.5)	24,577 (92.9)
2	2,036 (8.3)	2,116 (7.8)	1,979 (7.5)	2,024 (7.5)	1,873 (7.1)
Vascular disease, n (%)					
0	19,833 (81.0)	22,324 (81.9)	21,474 (81.8)	21,964 (81.1)	21,527 (81.4)
1	4,656 (19.0)	4,947 (18.1)	4,783 (18.2)	5,122 (18.9)	4,923 (18.6)
Sex, n (%)					
0	-	-	-	-	-
1	24,489 (100.0)	27,271 (100.0)	26,257 (100.0)	27,086 (100.0)	26,450 (100.0)
HAS-BLED score, mean ± SD	1.88 ± 0.98	1.82 ± 0.98	1.79 ± 0.98	1.78 ± 0.98	1.76 ± 0.97
Uncontrolled hypertension, n (%)					
0	16,731 (68.3)	19,030 (69.8)	18,624 (70.9)	19,404 (71.6)	19,139 (72.4)
1	7,758 (31.7)	8,241 (30.2)	7,633 (29.1)	7,682 (28.4)	7,311 (27.6)
Abnormal renal function, n (%)					
0	21,356 (87.2)	23,846 (87.4)	22,822 (86.9)	23,489 (86.7)	23,039 (87.1)
1	3,133 (12.8)	3,425 (12.6)	3,435 (13.1)	3,597 (13.3)	3,411 (12.9)
Abnormal liver function, n (%)					
0	23,900 (97.6)	26,675 (97.8)	25,742 (98.0)	26,557 (98.0)	25,928 (98.0)
1	589 (2.4)	596 (2.2)	515 (2.0)	529 (2.0)	522 (2.0)
Ischemic stroke/TIA (history), n (%)					

© 2023 Chen Q et al. *JAMA Network Open.*

0	23,277 (95.1)	26,083 (95.6)	25,120 (95.7)	25,922 (95.7)	25,364 (95.9)
1	1,212 (4.9)	1,188 (4.4)	1,137 (4.3)	1,164 (4.3)	1,086 (4.1)
Major bleeding, n (%)					
0	23,719 (96.9)	26,549 (97.4)	25,611 (97.5)	26,394 (97.4)	25,805 (97.6)
1	770 (3.1)	722 (2.6)	646 (2.5)	692 (2.6)	645 (2.4)
Elderly, n (%)					
0	3,098 (12.7)	3,646 (13.4)	3,655 (13.9)	3,721 (13.7)	3,713 (14.0)
1	21,391 (87.3)	23,625 (86.6)	22,602 (86.1)	23,365 (86.3)	22,737 (86.0)
Alcohol abuse, n (%)					
0	24,291 (99.2)	27,082 (99.3)	26,043 (99.2)	26,841 (99.1)	26,206 (99.1)
1	198 (0.8)	189 (0.7)	214 (0.8)	245 (0.9)	244 (0.9)
Antiplatelet agents or NSIADs, n (%)					
0	13,421 (54.8)	15,521 (56.9)	15,341 (58.4)	16,043 (59.2)	15,791 (59.7)
1	11,068 (45.2)	11,750 (43.1)	10,916 (41.6)	11,043 (40.8)	10,659 (40.3)

Abbreviations: TIA, transient ischemic attack; NSAID, nonsteroidal anti-inflammatory drugs.

© 2023 Chen Q et al. *JAMA Network Open*.

eTable 28. Time Trends in Composition of CHA_2DS_2-VASc Score and HAS-BLED Score of Incident Nonvalvular Atrial Fibrillation Patients in the Netherlands, Excluding Patients With Preexisting Chronic Oral Anticoagulant Treatment

	2014 (N=40,663)	2015 (N=44,149)	2016 (N=43,353)	2017 (N=44,417)	2018 (N=42,586)
CHA_2DS_2-VASc score, mean ± SD	2.95 ± 1.67	2.92 ± 1.68	2.90 ± 1.68	2.92 ± 1.68	2.91 ± 1.67
Heart failure, n (%)					
0	32,162 (79.1)	34,943 (79.1)	34,408 (79.4)	35,327 (79.5)	33,600 (78.9)
1	8,501 (20.9)	9,206 (20.9)	8,945 (20.6)	9,090 (20.5)	8,986 (21.1)
Hypertension, n (%)					
0	26,183 (64.4)	28,083 (63.6)	27,513 (63.5)	27,653 (62.3)	26,178 (61.5)
1	14,480 (35.6)	16,066 (36.4)	15,840 (36.5)	16,764 (37.7)	16,408 (38.5)
Age, n (%)					
0	8,542 (21.0)	9,465 (21.4)	9,518 (22.0)	9,751 (22.0)	9,428 (22.1)
1	11,120 (27.3)	12,100 (27.4)	12,144 (28.0)	12,680 (28.5)	12,439 (29.2)
2	21,001 (51.6)	22,584 (51.2)	21,691 (50.0)	21,986 (49.5)	20,719 (48.7)
Diabetes, n (%)					
0	32,777 (80.6)	35,775 (81.0)	34,897 (80.5)	35,737 (80.5)	34,251 (80.4)
1	7,886 (19.4)	8,374 (19.0)	8,456 (19.5)	8,680 (19.5)	8,335 (19.6)
Stroke/TIA/thromboembolism, n (%)					
0	37,339 (91.8)	40,756 (92.3)	40,124 (92.6)	41,125 (92.6)	39,646 (93.1)
2	3,324 (8.2)	3,393 (7.7)	3,229 (7.4)	3,292 (7.4)	2,940 (6.9)
Vascular disease, n (%)					
0	29,728 (73.1)	32,935 (74.6)	32,174 (74.2)	32,507 (73.2)	30,931 (72.6)
1	10,935 (26.9)	11,214 (25.4)	11,179 (25.8)	11,910 (26.8)	11,655 (27.4)
Sex, n (%)					
0	22,407 (55.1)	23,997 (54.4)	23,838 (55.0)	24,609 (55.4)	23,775 (55.8)
1	18,256 (44.9)	20,152 (45.6)	19,515 (45.0)	19,808 (44.6)	18,811 (44.2)
HAS-BLED score, mean ± SD	1.79 ± 1.04	1.73 ± 1.04	1.72 ± 1.05	1.71 ± 1.04	1.69 ± 1.04
Uncontrolled hypertension, n (%)					
0	30,825 (75.8)	33,843 (76.7)	33,467 (77.2)	34,621 (77.9)	33,396 (78.4)
1	9,838 (24.2)	10,306 (23.3)	9,886 (22.8)	9,796 (22.1)	9,190 (21.6)
Abnormal renal function, n (%)					
0	35,595 (87.5)	38,676 (87.6)	37,701 (87.0)	38,594 (86.9)	37,159 (87.3)
1	5,068 (12.5)	5,473 (12.4)	5,652 (13.0)	5,823 (13.1)	5,427 (12.7)
Abnormal liver function, n (%)					
0	39,588 (97.4)	43,107 (97.6)	42,267 (97.5)	43,349 (97.6)	41,579 (97.6)
1	1,075 (2.6)	1,042 (2.4)	1,086 (2.5)	1,068 (2.4)	1,007 (2.4)

© 2023 Chen Q et al. *JAMA Network Open.*

Ischemic stroke/TIA (history), n (%)					
0	38,673 (95.1)	42,220 (95.6)	41,483 (95.7)	42,578 (95.9)	40,858 (95.9)
1	1,990 (4.9)	1,929 (4.4)	1,870 (4.3)	1,839 (4.1)	1,728 (4.1)
Major bleeding, n (%)					
0	39,523 (97.2)	43,018 (97.4)	42,368 (97.7)	43,361 (97.6)	41,616 (97.7)
1	1,140 (2.8)	1,131 (2.6)	985 (2.3)	1,056 (2.4)	970 (2.3)
Elderly, n (%)					
0	8,542 (21.0)	9,465 (21.4)	9,518 (22.0)	9,751 (22.0)	9,428 (22.1)
1	32,121 (79.0)	34,684 (78.6)	33,835 (78.0)	34,666 (78.0)	33,158 (77.9)
Alcohol abuse, n (%)					
0	39,908 (98.1)	43,416 (98.3)	42,501 (98.0)	43,461 (97.8)	41,699 (97.9)
1	755 (1.9)	733 (1.7)	852 (2.0)	956 (2.2)	887 (2.1)
Antiplatelet agents or NSIADs, n (%)					
0	20,056 (49.3)	22,938 (52.0)	23,075 (53.2)	23,867 (53.7)	22,895 (53.8)
1	20,607 (50.7)	21,211 (48.0)	20,278 (46.8)	20,550 (46.3)	19,691 (46.2)

Abbreviations: TIA, transient ischemic attack; NSAID, nonsteroidal anti-inflammatory drugs.

© 2023 Chen Q et al. *JAMA Network Open.*

eTable 29. Time Trends in Composition of CHA_2DS_2-VASc Score and HAS-BLED Score of Male Patients With Incident Nonvalvular Atrial Fibrillation in the Netherlands, Excluding Patients With Preexisting Chronic Oral Anticoagulant Treatment

	2014 (N=22,407)	2015 (N=23,997)	2016 (N=23,838)	2017 (N=24,609)	2018 (N=23,775)
CHA_2DS_2-VASc score, mean ± SD	2.34 ± 1.56	2.31 ± 1.58	2.31 ± 1.58	2.33 ± 1.58	2.35 ± 1.57
Heart failure, n (%)					
0	18,313 (81.7)	19,546 (81.5)	19,432 (81.5)	20,044 (81.4)	19,172 (80.6)
1	4,094 (18.3)	4,451 (18.5)	4,406 (18.5)	4,565 (18.6)	4,603 (19.4)
Hypertension, n (%)					
0	15,100 (67.4)	16,059 (66.9)	15,823 (66.4)	15,990 (65.0)	15,176 (63.8)
1	7,307 (32.6)	7,938 (33.1)	8,015 (33.6)	8,619 (35.0)	8,599 (36.2)
Age, n (%)					
0	6,005 (26.8)	6,528 (27.2)	6,546 (27.5)	6,793 (27.6)	6,474 (27.2)
1	6,949 (31.0)	7,318 (30.5)	7,386 (31.0)	7,663 (31.1)	7,599 (32.0)
2	9,453 (42.2)	10,151 (42.3)	9,906 (41.6)	10,153 (41.3)	9,702 (40.8)
Diabetes, n (%)					
0	18,158 (81.0)	19,480 (81.2)	19,234 (80.7)	19,806 (80.5)	19,115 (80.4)
1	4,249 (19.0)	4,517 (18.8)	4,604 (19.3)	4,803 (19.5)	4,660 (19.6)
Stroke/TIA/thromboembolism, n (%)					
0	20,608 (92.0)	22,216 (92.6)	22,131 (92.8)	22,853 (92.9)	22,208 (93.4)
2	1,799 (8.0)	1,781 (7.4)	1,707 (7.2)	1,756 (7.1)	1,567 (6.6)
Vascular disease, n (%)					
0	15,051 (67.2)	16,592 (69.1)	16,375 (68.7)	16,641 (67.6)	15,854 (66.7)
1	7,356 (32.8)	7,405 (30.9)	7,463 (31.3)	7,968 (32.4)	7,921 (33.3)
Sex, n (%)					
0	22,407 (100.0)	23,997 (100.0)	23,838 (100.0)	24,609 (100.0)	23,775 (100.0)
1	-	-	-	-	-
HAS-BLED score, mean ± SD	1.71 ± 1.07	1.66 ± 1.07	1.66 ± 1.08	1.64 ± 1.07	1.64 ± 1.07
Uncontrolled hypertension, n (%)					
0	17,612 (78.6)	19,065 (79.4)	18,939 (79.4)	19,678 (80.0)	19,073 (80.2)
1	4,795 (21.4)	4,932 (20.6)	4,899 (20.6)	4,931 (20.0)	4,702 (19.8)
Abnormal renal function, n (%)					
0	19,695 (87.9)	21,129 (88.0)	20,790 (87.2)	21,460 (87.2)	20,854 (87.7)
1	2,712 (12.1)	2,868 (12.0)	3,048 (12.8)	3,149 (12.8)	2,921 (12.3)
Abnormal liver function, n (%)					
0	21,797 (97.3)	23,422 (97.6)	23,180 (97.2)	23,983 (97.5)	23,179 (97.5)
1	610 (2.7)	575 (2.4)	658 (2.8)	626 (2.5)	596 (2.5)

© 2023 Chen Q et al. *JAMA Network Open.*

Ischemic stroke/TIA (history), n (%)					
0	21,339 (95.2)	22,982 (95.8)	22,858 (95.9)	23,667 (96.2)	22,873 (96.2)
1	1,068 (4.8)	1,015 (4.2)	980 (4.1)	942 (3.8)	902 (3.8)
Major bleeding, n (%)					
0	21,763 (97.1)	23,343 (97.3)	23,274 (97.6)	23,997 (97.5)	23,190 (97.5)
1	644 (2.9)	654 (2.7)	564 (2.4)	612 (2.5)	585 (2.5)
Elderly, n (%)					
0	6,005 (26.8)	6,528 (27.2)	6,546 (27.5)	6,793 (27.6)	6,474 (27.2)
1	16,402 (73.2)	17,469 (72.8)	17,292 (72.5)	17,816 (72.4)	17,301 (72.8)
Alcohol abuse, n (%)					
0	21,829 (97.4)	23,424 (97.6)	23,167 (97.2)	23,855 (96.9)	23,091 (97.1)
1	578 (2.6)	573 (2.4)	671 (2.8)	754 (3.1)	684 (2.9)
Antiplatelet agents or NSIADs, n (%)					
0	10,951 (48.9)	12,290 (51.2)	12,495 (52.4)	12,989 (52.8)	12,517 (52.6)
1	11,456 (51.1)	11,707 (48.8)	11,343 (47.6)	11,620 (47.2)	11,258 (47.4)

Abbreviations: TIA, transient ischemic attack; NSAID, nonsteroidal anti-inflammatory drugs.

© 2023 Chen Q et al. *JAMA Network Open.*

eTable 30. Time Trends in Composition of CHA_2DS_2-VASc Score and HAS-BLED Score of Female Patients With Incident Nonvalvular Atrial Fibrillation in the Netherlands, Excluding Patients With Preexisting Chronic Oral Anticoagulant Treatment

	2014 (N=18,256)	2015 (N=20,152)	2016 (N=19,515)	2017 (N=19,808)	2018 (N=18,811)
CHA_2DS_2-VASc score, mean ± SD	3.69 ± 1.49	3.65 ± 1.50	3.63 ± 1.51	3.64 ± 1.50	3.62 ± 1.51
Heart failure, n (%)					
0	13,849 (75.9)	15,397 (76.4)	14,976 (76.7)	15,283 (77.2)	14,428 (76.7)
1	4,407 (24.1)	4,755 (23.6)	4,539 (23.3)	4,525 (22.8)	4,383 (23.3)
Hypertension, n (%)					
0	11,083 (60.7)	12,024 (59.7)	11,690 (59.9)	11,663 (58.9)	11,002 (58.5)
1	7,173 (39.3)	8,128 (40.3)	7,825 (40.1)	8,145 (41.1)	7,809 (41.5)
Age, n (%)					
0	2,537 (13.9)	2,937 (14.6)	2,972 (15.2)	2,958 (14.9)	2,954 (15.7)
1	4,171 (22.8)	4,782 (23.7)	4,758 (24.4)	5,017 (25.3)	4,840 (25.7)
2	11,548 (63.3)	12,433 (61.7)	11,785 (60.4)	11,833 (59.7)	11,017 (58.6)
Diabetes, n (%)					
0	14,619 (80.1)	16,295 (80.9)	15,663 (80.3)	15,931 (80.4)	15,136 (80.5)
1	3,637 (19.9)	3,857 (19.1)	3,852 (19.7)	3,877 (19.6)	3,675 (19.5)
Stroke/TIA/thromboembolism, n (%)					
0	16,731 (91.6)	18,540 (92.0)	17,993 (92.2)	18,272 (92.2)	17,438 (92.7)
2	1,525 (8.4)	1,612 (8.0)	1,522 (7.8)	1,536 (7.8)	1,373 (7.3)
Vascular disease, n (%)					
0	14,677 (80.4)	16,343 (81.1)	15,799 (81.0)	15,866 (80.1)	15,077 (80.1)
1	3,579 (19.6)	3,809 (18.9)	3,716 (19.0)	3,942 (19.9)	3,734 (19.9)
Sex, n (%)					
0	-	-	-	-	-
1	18,256 (100.0)	20,152 (100.0)	19,515 (100.0)	19,808 (100.0)	18,811 (100.0)
HAS-BLED score, mean ± SD	1.88 ± 1.00	1.82 ± 1.00	1.79 ± 1.01	1.78 ± 1.00	1.76 ± 1.00
Uncontrolled hypertension, n (%)					
0	13,213 (72.4)	14,778 (73.3)	14,528 (74.4)	14,943 (75.4)	14,323 (76.1)
1	5,043 (27.6)	5,374 (26.7)	4,987 (25.6)	4,865 (24.6)	4,488 (23.9)
Abnormal renal function, n (%)					
0	15,900 (87.1)	17,547 (87.1)	16,911 (86.7)	17,134 (86.5)	16,305 (86.7)
1	2,356 (12.9)	2,605 (12.9)	2,604 (13.3)	2,674 (13.5)	2,506 (13.3)
Abnormal liver function, n (%)					
0	17,791 (97.5)	19,685 (97.7)	19,087 (97.8)	19,366 (97.8)	18,400 (97.8)
1	465 (2.5)	467 (2.3)	428 (2.2)	442 (2.2)	411 (2.2)

© 2023 Chen Q et al. *JAMA Network Open.*

Ischemic stroke/TIA (history), n (%)					
0	17,334 (94.9)	19,238 (95.5)	18,625 (95.4)	18,911 (95.5)	17,985 (95.6)
1	922 (5.1)	914 (4.5)	890 (4.6)	897 (4.5)	826 (4.4)
Major bleeding, n (%)					
0	17,760 (97.3)	19,675 (97.6)	19,094 (97.8)	19,364 (97.8)	18,426 (98.0)
1	496 (2.7)	477 (2.4)	421 (2.2)	444 (2.2)	385 (2.0)
Elderly, n (%)					
0	2,537 (13.9)	2,937 (14.6)	2,972 (15.2)	2,958 (14.9)	2,954 (15.7)
1	15,719 (86.1)	17,215 (85.4)	16,543 (84.8)	16,850 (85.1)	15,857 (84.3)
Alcohol abuse, n (%)					
0	18,079 (99.0)	19,992 (99.2)	19,334 (99.1)	19,606 (99.0)	18,608 (98.9)
1	177 (1.0)	160 (0.8)	181 (0.9)	202 (1.0)	203 (1.1)
Antiplatelet agents or NSIADs, n (%)					
0	9,105 (49.9)	10,648 (52.8)	10,580 (54.2)	10,878 (54.9)	10,378 (55.2)
1	9,151 (50.1)	9,504 (47.2)	8,935 (45.8)	8,930 (45.1)	8,433 (44.8)

Abbreviations: TIA, transient ischemic attack; NSAID, nonsteroidal anti-inflammatory drugs.

© 2023 Chen Q et al. *JAMA Network Open.*

eTable 31. Association Between Baseline CHA_2DS_2-VASc Score and 1-Year Risk of Ischemic Stroke

CHA_2DS_2-VASc score (all study population)							**Literature (Lip et al*)**
Score	No. at risk†	Observation time (PYs)	No. events	Cumulative incidence (%, 95%CI)‡	Incidence rate (per 100 PYs, 95%CI)	Crude hazard ratio (95%CI)	One-year TE event rate (%, 95%CI)*
0	22,964	22,317	91	0.40 (0.32-0.48)	0.41 (0.33-0.50)	1 (Reference)	-
1	41,033	38,910	242	0.59 (0.52-0.67)	0.62 (0.55-0.71)	1.52 (1.19-1.93)	
2	60,250	54,251	586	0.97 (0.90-1.05)	1.08 (0.99-1.17)	2.61 (2.09-3.25)	
3	70,297	60,336	964	1.37 (1.29-1.46)	1.60 (1.50-1.70)	3.81 (3.08-4.73)	
4	56,033	45,768	1,041	1.86 (1.75-1.97)	2.27 (2.14-2.42)	5.37 (4.33-6.65)	
5	31,495	24,642	836	2.65 (2.48-2.84)	3.39 (3.17-3.63)	7.93 (6.39-9.84)	
6	13,389	9,931	524	3.91 (3.59-4.25)	5.28 (4.83-5.75)	12.20 (9.77-15.25)	
≥7	5,840	4,084	314	5.38 (4.82-5.98)	7.69 (6.86-8.59)	17.49 (13.85-22.08)	
CHA_2DS_2-VASc score (excluding patients with pre-existing chronic oral anticoagulant treatment)							
0	16,471	15,899	58	0.35 (0.27-0.45)	0.36 (0.28-0.47)	1 (Reference)	0 (0-0)
1	29,161	27,343	175	0.60 (0.52-0.69)	0.64 (0.55-0.74)	1.74 (1.30-2.35)	0.6 (0.0-3.4)
2	42,432	37,747	428	1.01 (0.92-1.11)	1.13 (1.03-1.25)	3.05 (2.32-4.01)	1.6 (0.3-4.7)
3	50,270	42,690	708	1.41 (1.31-1.51)	1.66 (1.54-1.79)	4.41 (3.38-5.77)	3.9 (1.7-7.6)
4	40,212	32,553	774	1.92 (1.79-2.06)	2.38 (2.21-2.55)	6.26 (4.79-8.17)	1.9 (0.5-4.9)
5	22,661	17,553	627	2.77 (2.56-2.99)	3.57 (3.30-3.86)	9.30 (7.11-12.17)	3.2 (0.7-9.0)
6	9,657	7,045	391	4.05 (3.67-4.46)	5.55 (5.01-6.13)	14.28 (10.84-18.81)	3.6 (0.4-12.3)
≥7	4,304	2,965	245	5.69 (5.03-6.41)	8.26 (7.26-9.37)	20.89 (15.69-27.82)	7: 8.0 (1.0-26.0)<br>8: 11.1 (0.3-48.3)<br>9: 100 (2.5-100)

† Individuals who were primarily admitted to the index hospitalization for ischemic stroke were excluded.

‡ Estimated by the cumulative incidence competing risk method

* The results were excerpted from Table 6 of the literature by Lip et al (Chest. 2010;137(2):263-272), which was based on a cohort of non-valvular atrial fibrillation patients who were not receiving anticoagulants at baseline. According to the literature, TE included ischemic stroke, transient ischemic attack, and peripheral embolism, and the 95%CI of the one-year TE event rate was estimated by binomial approximation.

Abbreviations: PY, person-year; CI, confidence interval; TE, thromboembolism.

© 2023 Chen Q et al. *JAMA Network Open.*

eTable 32. Total Numbers of Different Diagnosis Records in the Data Source by Calendar Years

Calendar year	Any diagnosis (proportion to total population)	No. Dutch population†	No. diagnosis records‡ (proportion to total diagnosis records)						
			Atrial fibrillation	Rheumatic mitral stenosis/ Mechanical heart valves	Ischemic stroke	Major bleeding	Intracranial haemorrhage	Gastrointestinal bleeding	
The data source was the National Medical Registration (LMR)*.									
2010	5,823,701 (35.14%)	16,574,989	77,581 (1.33%)	7,370 (0.13%)	21,947 (0.38%)	38,516 (0.66%)	7,149 (0.12%)	18,154 (0.31%)	
2011	6,144,195 (36.89%)	16,655,799	85,506 (1.39%)	8,299 (0.14%)	22,252 (0.36%)	39,817 (0.65%)	7,008 (0.11%)	19,108 (0.31%)	
2012	6,645,533 (39.72%)	16,730,348	96,532 (1.45%)	9,942 (0.15%)	24,332 (0.37%)	43,903 (0.66%)	7,391 (0.11%)	20,109 (0.30%)	
The data source was started to switch from the LMR to the National Basic Register Hospital Care (LBZ)*.									
2013	8,615,772 (51.35%)	16,779,575	129,179 (1.50%)	12,094 (0.14%)	24,003 (0.28%)	62,450 (0.72%)	9,063 (0.11%)	27,670 (0.32%)	
All hospitals were switched to the LBZ*.									
2014	8,362,555 (49.69%)	16,829,289	122,470 (1.46%)	12,248 (0.15%)	28,309 (0.34%)	65,808 (0.79%)	9,519 (0.11%)	29,453 (0.35%)	
2015	8,543,025 (50.55%)	16,900,726	145,594 (1.70%)	13,374 (0.16%)	30,504 (0.36%)	69,905 (0.82%)	9,679 (0.11%)	30,127 (0.35%)	
2016	8,552,436 (50.37%)	16,979,120	145,367 (1.70%)	13,437 (0.16%)	32,723 (0.38%)	72,319 (0.85%)	9,668 (0.11%)	31,537 (0.37%)	
2017	8,523,159 (49.90%)	17,081,507	150,325 (1.76%)	12,915 (0.15%)	33,592 (0.39%)	71,934 (0.84%)	9,783 (0.11%)	31,018 (0.36%)	
2018	8,501,551 (49.48%)	17,181,084	152,225 (1.79%)	12,919 (0.15%)	34,012 (0.40%)	73,783 (0.87%)	9,368 (0.11%)	32,506 (0.38%)	
2019	9,059,363 (52.42%)	17,282,163	158,386 (1.75%)	13,320 (0.15%)	34,378 (0.38%)	75,323 (0.83%)	9,802 (0.11%)	32,667 (0.36%)	

† Numbers of Dutch population were accessed from the StatLine open data provided by Statistics Netherlands (https://opendata.cbs.nl/#/CBS/en/dataset/83474ENG/table, accessed on March 18, 2022).
‡ Regardless of primary/main diagnosis
* See eMethods for details.

© 2023 Chen Q et al. *JAMA Network Open.*

eFigure 1. Illustration of the Calculation of Proportion of Days Covered by Antithrombotic Agents

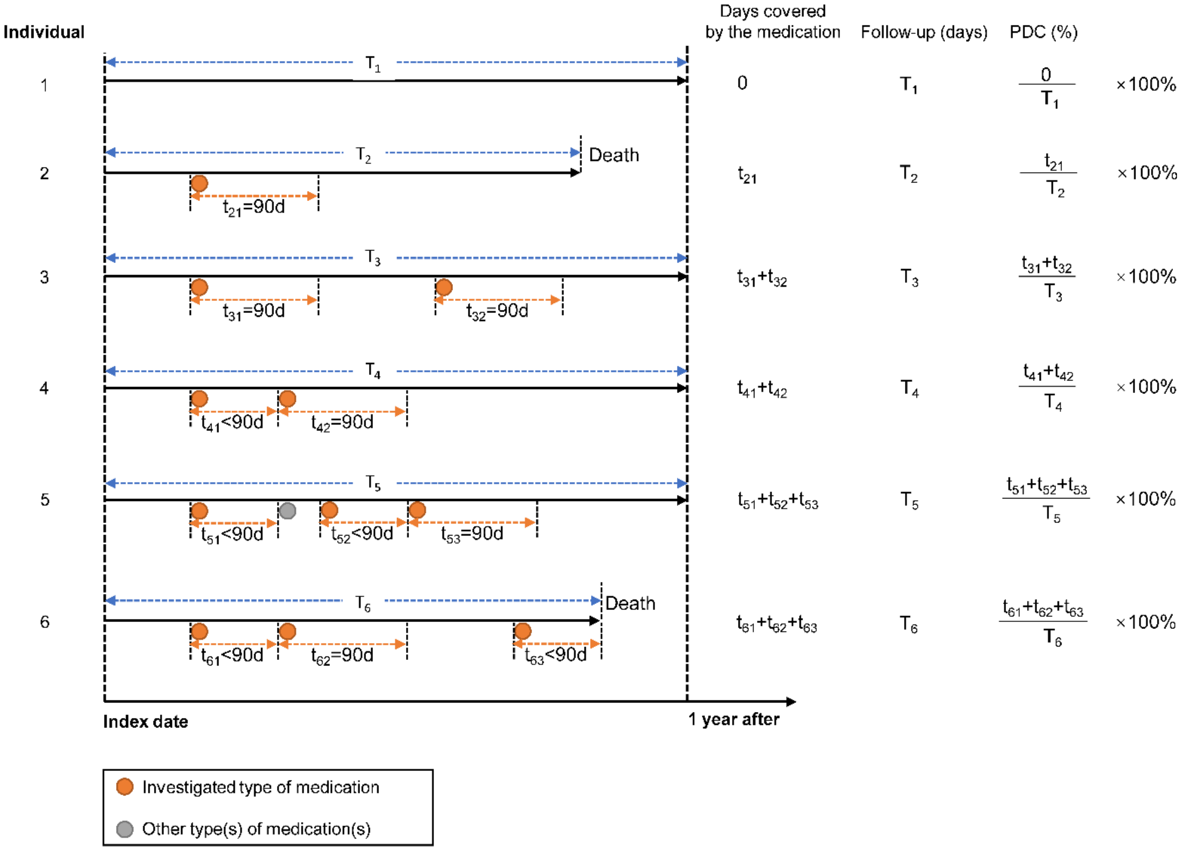

When determining the PDCs by oral anticoagulants (OACs), heparin group was considered as the other type of medication; when determining the PDCs by vitamin K antagonists (VKAs), heparin group and direct oral anticoagulants (DOACs) were considered as the other types of medications; when determining the PDCs by DOACs, heparin group and VKAs were considered as the other types of medications. Antiplatelet agent prescriptions were not taken into account when determining PDCs by OACs/heparin group or OACs only, and vice versa.

© 2023 Chen Q et al. *JAMA Network Open.*

eFigure 2. Cumulative Incidence of Ischemic Stroke Within 1 Year After Incident Nonvalvular Atrial Fibrillation Diagnosis†

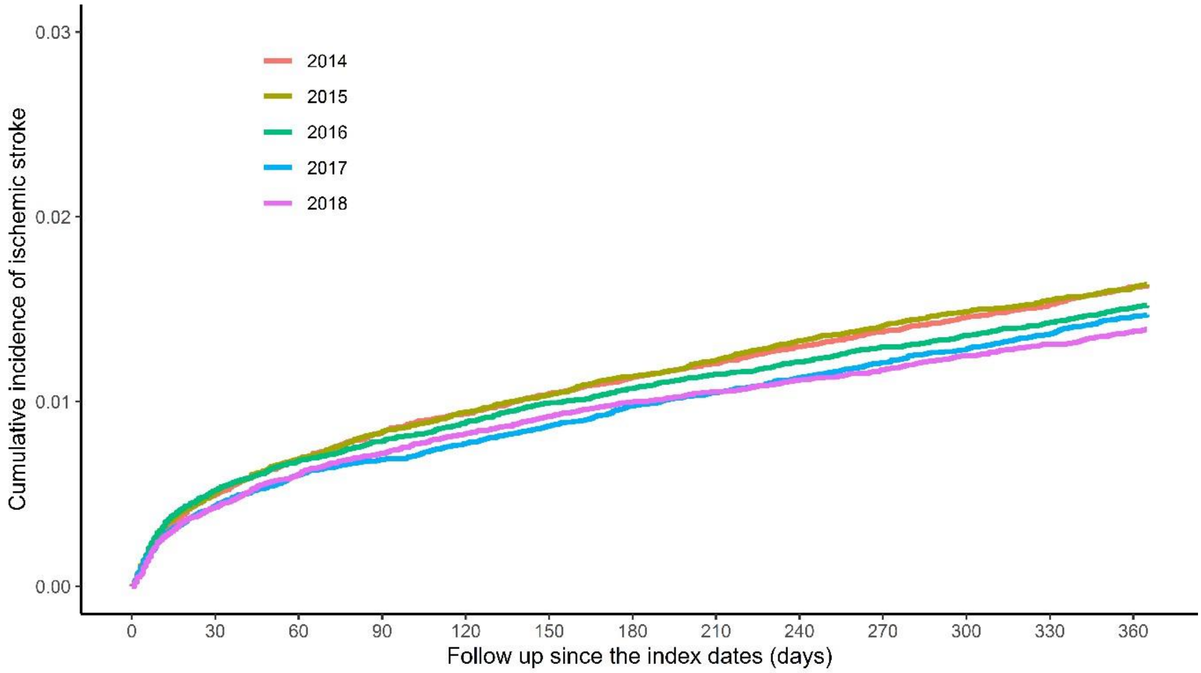

Number at risk

	55880	51988	50436	49420	48674	48030	47467	46982	46503	46081	45656	45248	44779
	61317	57050	55465	54506	53665	53005	52414	51881	51380	50907	50481	50053	49668
	60018	55924	54412	53395	52711	52084	51546	51039	50594	50154	49728	49304	48896
	62390	58128	56604	55622	54849	54259	53697	53172	52716	52227	51753	51307	50857
	61696	57262	55709	54806	54101	53499	52904	52419	51964	51550	51148	50752	50366

† Estimated by the cumulative incidence competing risk method

© 2023 Chen Q et al. *JAMA Network Open.*

eFigure 3. Cumulative Incidence of Major Bleeding Within 1 Year After Incident Nonvalvular Atrial Fibrillation Diagnosis†

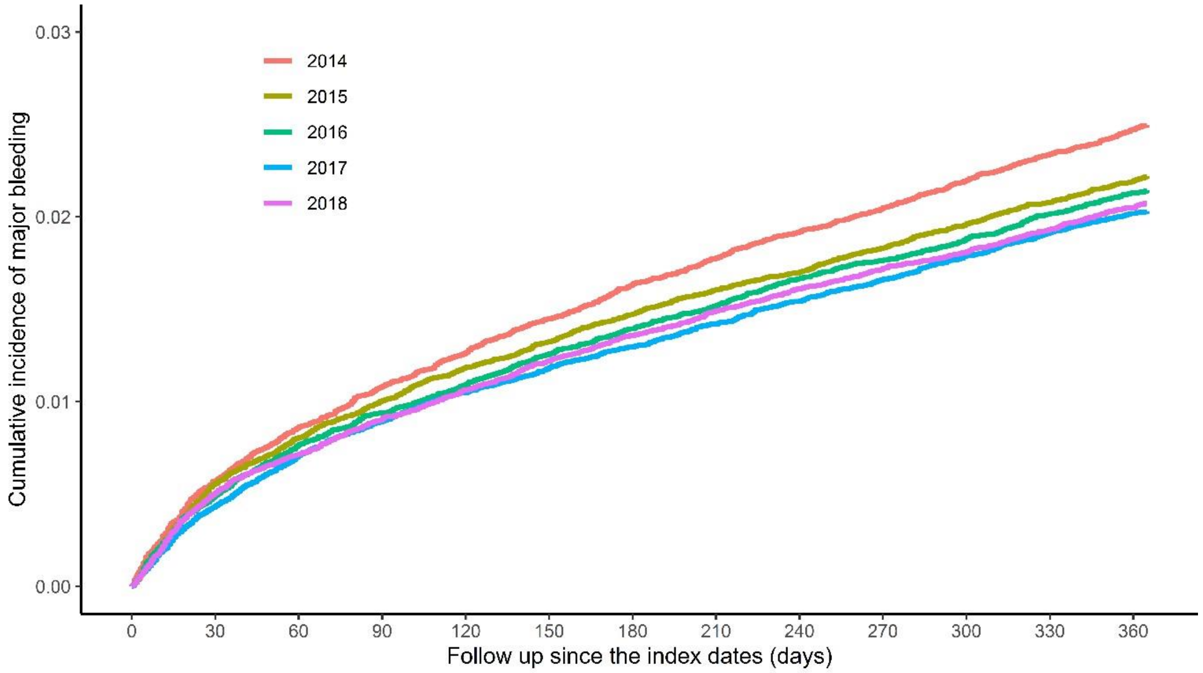

Number at risk

54773	50995	49423	48386	47620	46977	46392	45885	45403	44972	44535	44115	43643
60221	56080	54486	53509	52653	51988	51386	50854	50374	49889	49446	48996	48591
59078	55168	53597	52569	51867	51224	50683	50169	49690	49264	48827	48378	47957
61372	57242	55689	54666	53878	53284	52734	52203	51739	51242	50742	50297	49862
60701	56371	54823	53908	53189	52572	51955	51441	50972	50553	50159	49736	49329

† Estimated by the cumulative incidence competing risk method

© 2023 Chen Q et al. *JAMA Network Open.*

eFigure 4. Cumulative Incidence of Intracranial Hemorrhage Within 1 Year After Incident Nonvalvular Atrial Fibrillation Diagnosis†

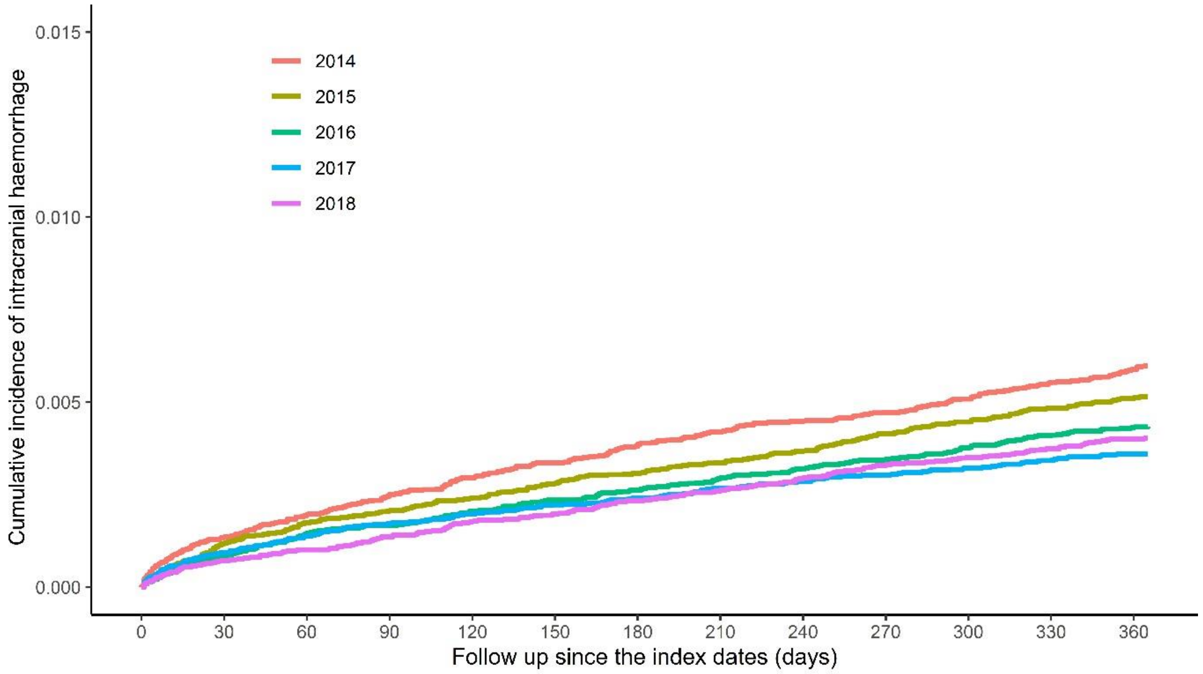

Number at risk

55468	51817	50321	49341	48618	48013	47472	46997	46545	46143	45739	45346	44906
60907	56911	55377	54471	53660	53025	52468	51967	51499	51036	50631	50211	49833
59655	55859	54383	53407	52748	52157	51645	51155	50731	50314	49906	49490	49101
62005	57993	56533	55565	54824	54266	53751	53252	52827	52367	51909	51496	51086
61339	57151	55658	54793	54123	53553	52973	52498	52062	51658	51286	50904	50544

† Estimated by the cumulative incidence competing risk method

© 2023 Chen Q et al. *JAMA Network Open.*

eFigure 5. Cumulative Incidence of Gastrointestinal Bleeding Within 1 Year After Incident Nonvalvular Atrial Fibrillation Diagnosis†

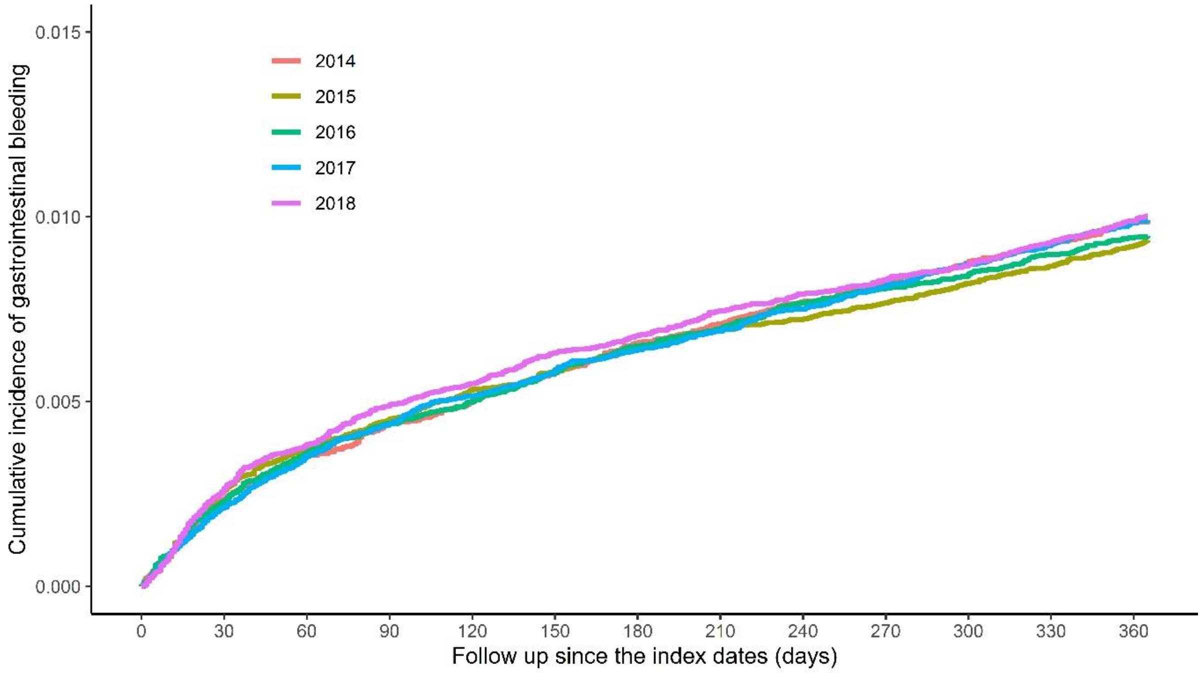

Number at risk

55522	51702	50168	49165	48432	47803	47239	46757	46289	45876	45461	45060	44603
60982	56819	55249	54305	53471	52830	52239	51723	51258	50789	50361	49939	49546
59716	55759	54233	53223	52548	51934	51403	50912	50458	50032	49621	49197	48797
62071	57894	56367	55367	54597	54022	53484	52970	52515	52033	51556	51137	50706
61388	57029	55485	54589	53898	53308	52725	52227	51774	51374	50994	50589	50201

† Estimated by the cumulative incidence competing risk method

© 2023 Chen Q et al. *JAMA Network Open.*

eFigure 6. Survival Probability Within 1 Year After Incident Nonvalvular Atrial Fibrillation Diagnosis†

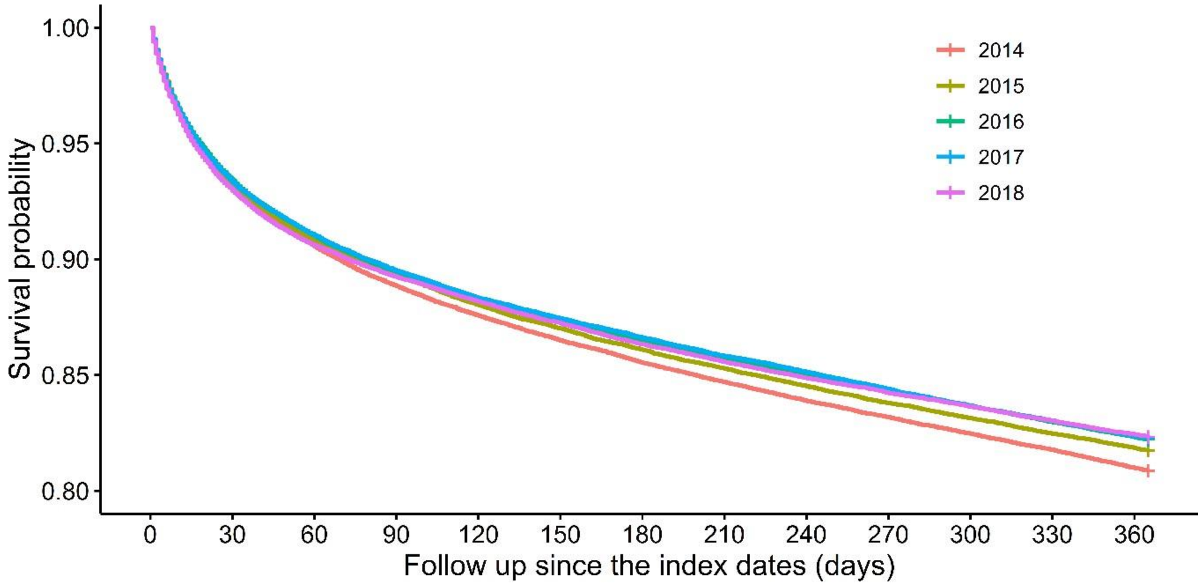

Number at risk

0	30	60	90	120	150	180	210	240	270	300	330	360
55880	52135	50642	49667	48950	48345	47809	47341	46892	46491	46091	45703	45268
61317	57247	55714	54803	53993	53365	52801	52302	51839	51389	50985	50575	50201
60018	56125	54659	53676	53024	52440	51932	51447	51029	50611	50211	49803	49418
62390	58300	56833	55869	55127	54569	54052	53557	53126	52668	52214	51807	51397
61696	57431	55926	55072	54409	53844	53275	52806	52372	51977	51610	51232	50871

Follow up since the index dates (days)

† Estimated by the Kaplan-Meier estimator

© 2023 Chen Q et al. *JAMA Network Open.*

eFigure 7. Cumulative Incidence of Ischemic Stroke Within 1 Year After Incident Nonvalvular Atrial Fibrillation Diagnosis, Excluding Patients With Preexisting Chronic Oral Anticoagulant Treatment†

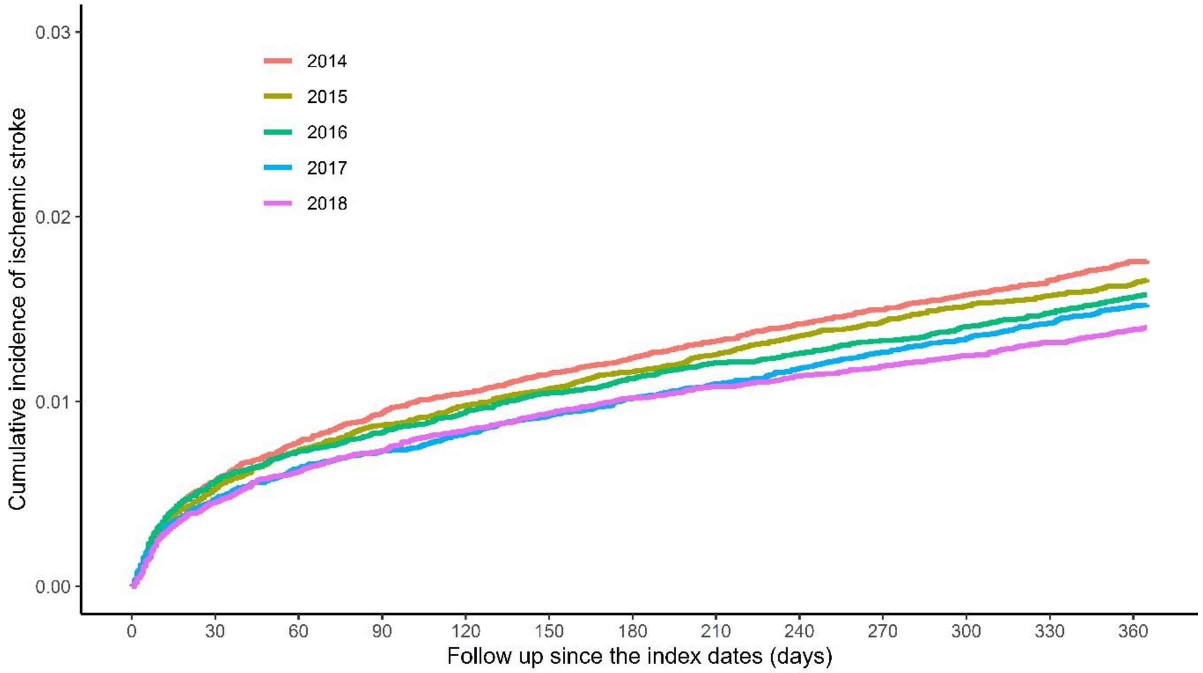

Number at risk

	40663	37640	36402	35648	35096	34623	34190	33835	33489	33166	32848	32551	32196
	44149	40849	39598	38850	38231	37732	37310	36898	36558	36190	35859	35535	35272
	43353	40112	38950	38193	37670	37201	36798	36425	36095	35772	35458	35137	34830
	44417	41084	39926	39188	38604	38167	37759	37375	37029	36661	36309	35994	35674
	42586	39231	38062	37386	36858	36439	36012	35657	35322	35040	34772	34482	34208

† Estimated by the cumulative incidence competing risk method

© 2023 Chen Q et al. *JAMA Network Open.*

eFigure 8. Cumulative Incidence of Major Bleeding Within 1 Year After Incident Nonvalvular Atrial Fibrillation Diagnosis, Excluding Patients With Preexisting Chronic Oral Anticoagulant Treatment†

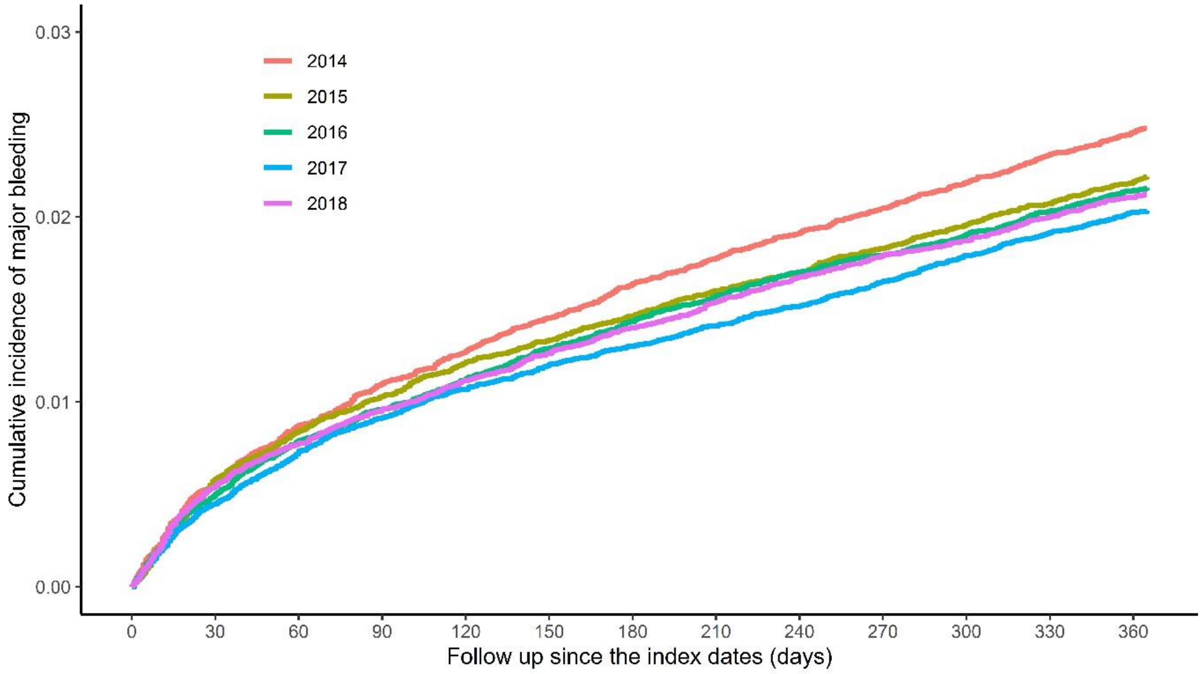

Number at risk

40002	37047	35780	35007	34443	33971	33523	33157	32805	32472	32149	31842	31491
43474	40246	38997	38234	37607	37110	36685	36277	35952	35573	35235	34893	34607
42799	39679	38462	37690	37154	36671	36263	35886	35531	35217	34898	34563	34242
43801	40548	39356	38590	38001	37564	37161	36779	36435	36057	35676	35365	35048
42036	38728	37554	36865	36328	35899	35454	35073	34724	34429	34156	33848	33561

† Estimated by the cumulative incidence competing risk method

© 2023 Chen Q et al. *JAMA Network Open.*

eFigure 9. Cumulative Incidence of Intracranial Hemorrhage Within 1 Year After Incident Nonvalvular Atrial Fibrillation Diagnosis, Excluding Patients With Preexisting Chronic Oral Anticoagulant Treatment†

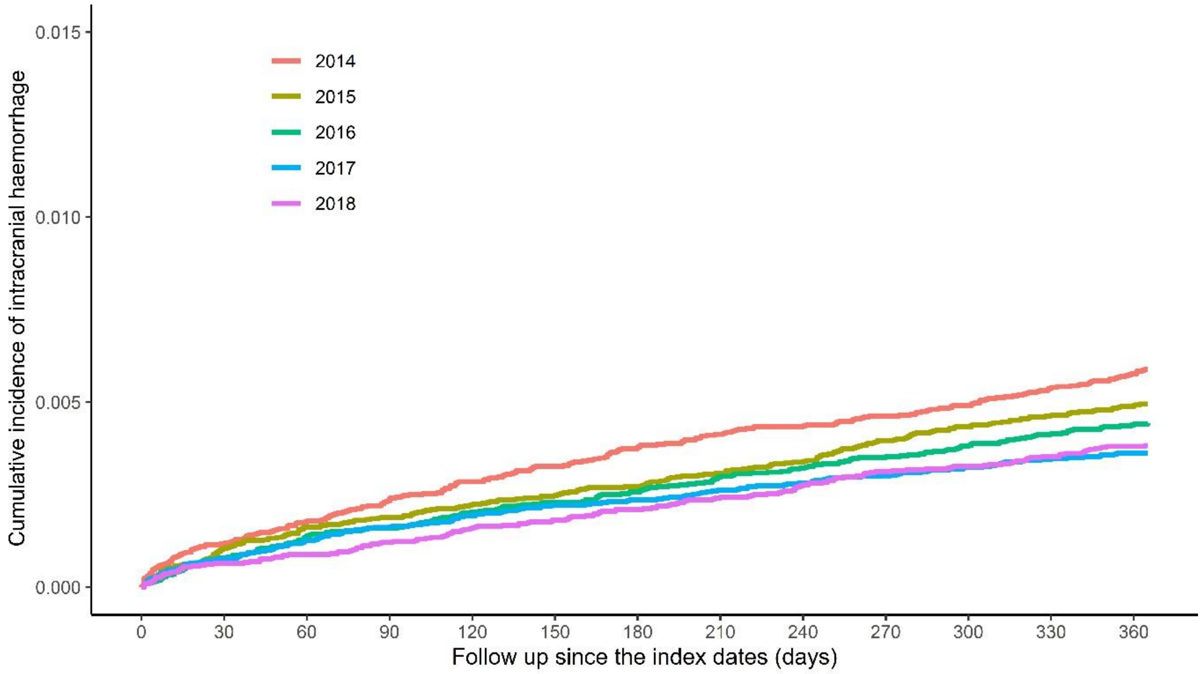

Number at risk

40419	37565	36360	35634	35097	34651	34231	33887	33562	33252	32952	32669	32334
43897	40784	39577	38861	38263	37783	37387	37001	36684	36320	36007	35688	35426
43132	40100	38960	38231	37727	37285	36900	36537	36216	35902	35605	35291	34994
44173	41026	39906	39179	38616	38202	37824	37461	37139	36791	36448	36160	35865
42392	39197	38069	37415	36912	36517	36100	35751	35430	35152	34898	34620	34361

† Estimated by the cumulative incidence competing risk method

© 2023 Chen Q et al. *JAMA Network Open.*

eFigure 10. Cumulative Incidence of Gastrointestinal Bleeding Within 1 Year After Incident Nonvalvular Atrial Fibrillation Diagnosis, Excluding Patients With Preexisting Chronic Oral Anticoagulant Treatment†

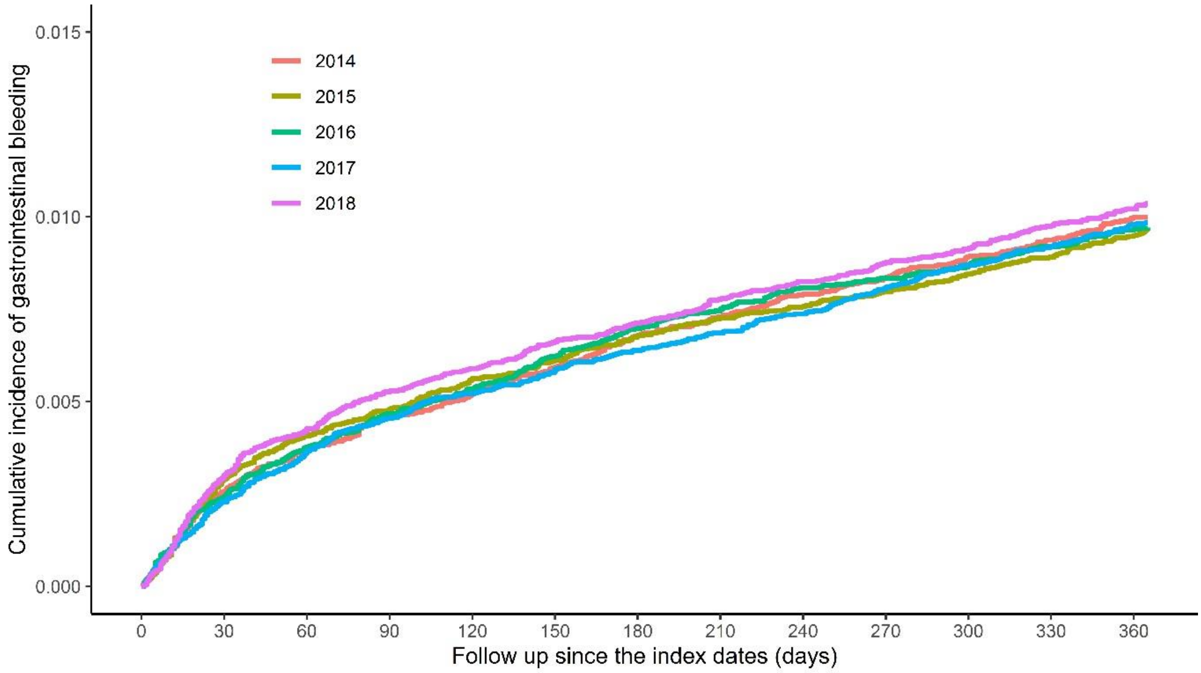

Number at risk

	40442	37475	36250	35508	34968	34509	34070	33722	33382	33068	32759	32469	32123
	43931	40708	39476	38739	38124	37635	37213	36814	36500	36136	35807	35484	35212
	43172	40038	38862	38098	37580	37118	36721	36363	36025	35718	35421	35102	34797
	44234	40967	39803	39052	38479	38056	37656	37283	36942	36578	36217	35922	35613
	42415	39107	37941	37264	36747	36336	35915	35553	35220	34940	34678	34388	34113

† Estimated by the cumulative incidence competing risk method

© 2023 Chen Q et al. *JAMA Network Open.*

eFigure 11. Survival Probability Within 1 Year After Incident Nonvalvular Atrial Fibrillation Diagnosis, Excluding Patients With Preexisting Chronic Oral Anticoagulant Treatment†

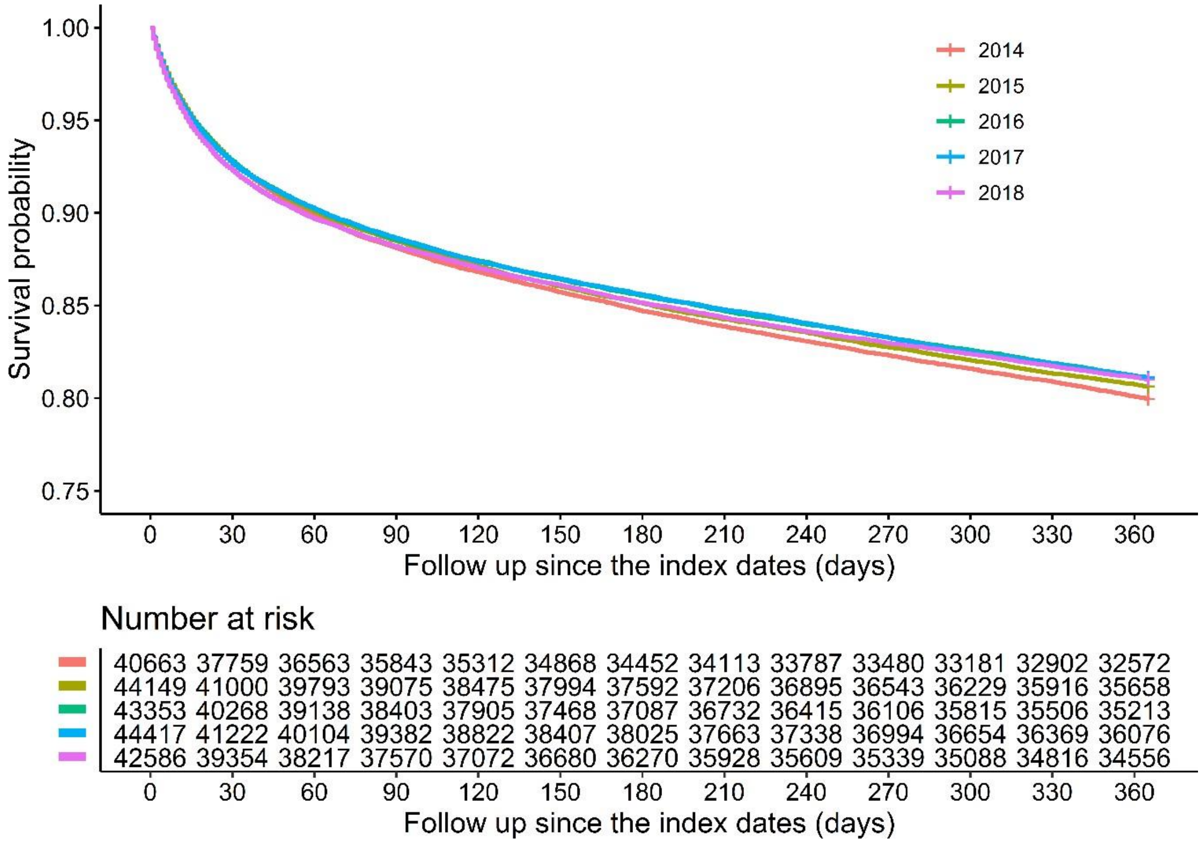

† Estimated by the Kaplan-Meier estimator

© 2023 Chen Q et al. *JAMA Network Open.*
