# Supplementary material for: Time Trends in Patient Characteristics, Anticoagulation Treatment, and Prognosis of Incident Nonvalvular Atrial Fibrillation in the Netherlands
Source: JAMA Netw Open. 2023 Apr 25;6(4):e239973. doi: 10.1001/jamanetworkopen.2023.9973 (PMC10130953; doi:10.1001/jamanetworkopen.2023.9973)
Supplement: Supplement 2. — Data Sharing Statement [file jamanetwopen-e239973-s002.pdf]

Data Sharing Statement

Chen. Time Trends in Patient Characteristics, Anticoagulation Treatment, and Prognosis of Incident Nonvalvular Atrial Fibrillation in the Netherlands. *JAMA Netw Open*. Published April 25, 2023. doi:10.1001/jamanetworkopen.2023.9973

Data
Data available: No

Additional Information
Explanation for why data not available: The study used non-public microdata from Statistics Netherlands, but these data cannot be shared directly by the authors. Under certain conditions, these microdata are accessible for statistical and scientific research. For further information: microdata@cbs.nl.
